# Cobalt-catalyzed C(sp^3^)–H/C(sp^2^)–H oxidative coupling between alkanes and benzamides†

**DOI:** 10.1039/c8ra01377b

**Published:** 2018-04-10

**Authors:** Shuangjie Li, Bao Wang, Guangyu Dong, Chunpu Li, Hong Liu

**Affiliations:** School of Pharmacy, China Pharmaceutical University Jiangsu Nanjing 210009 China; State Key Laboratory of Drug Research, Shanghai Institute of Materia Medica, Chinese Academy of Sciences 555 Zu Chong Zhi Road Shanghai 201203 China; Key Laboratory of Receptor Research, Shanghai Institute of Materia Medica, Chinese Academy of Sciences 555 Zu Chong Zhi Road Shanghai 201203 China; School of Life Science and Technology, ShanghaiTech University 100 Haike Road Shanghai 201210 China hliu@simm.ac.cn

## Abstract

A direct cobalt-catalyzed oxidative coupling between C(sp^2^)–H in unactivated benzamides and C(sp^3^)–H in simple alkanes, ethers and toluene derivatives was explored. This protocol achieves direct C–C formation without using alkyl or aryl halide surrogates and exhibits high practicality with ample substrate scope. The method provides a new way to construct linear and five- or six-membered ring moieties in bioactive molecules.

## Abstract 2

A direct cobalt-catalyzed oxidative coupling between C(sp^2^)–H in unactivated benzamides and C(sp^3^)–H in simple alkanes, ethers and toluene derivatives was explored.

## Graphical abstract

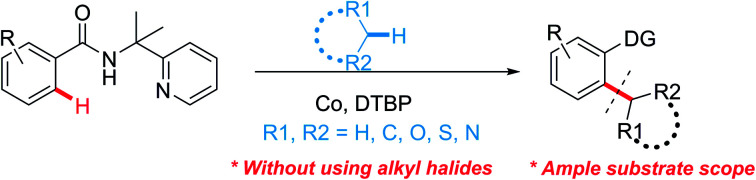

## Introduction

Five- or six-membered ring moieties such as cyclohexane, tetrahydrofuranare (THF), tetrahydropyrans (THP), morpholine, and 1,4-dioxane are fundamental structural motifs in numerous natural products, bioactive molecules and important synthetically useful compounds.^1^ These groups are essential to improve the aqueous solubility and good pharmacokinetic profiles of small molecule drugs.^2^ However, direct functionalization of these hydrophilic groups to complex organic compounds is challenging.

**Scheme 1:**
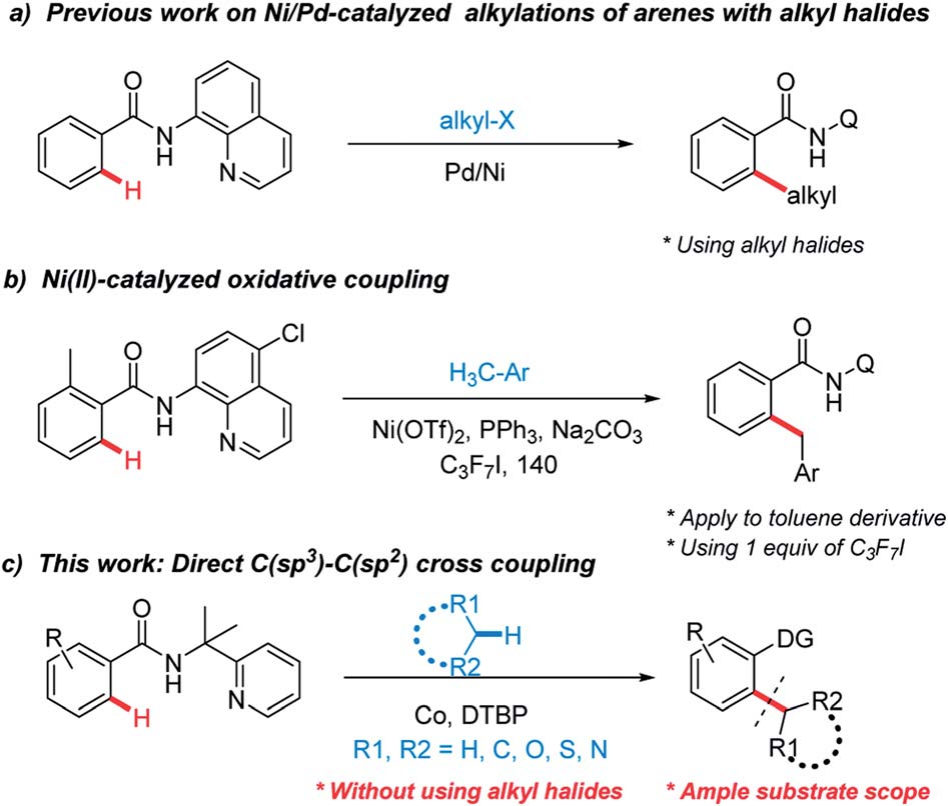
C(sp^3^)–C(sp^2^) cross coupling.

Much research effort has been expended into forging linkages between sp^2^ and sp^3^ hybridized carbons.^3^ Among them, selective C–H functionalization assisted by directing-groups has received considerable attention and emerged as an appealing strategy to construct C–C bonds.^4^ To date, alkyl halides species as sp^3^ components can be found from these excellent works (Scheme 1a).^5^

However, using alkyl halides make these methods less economical and their tendency toward β-hydride eliminations instead of oxidative additions hinder feasible application. Therefore, the development of direct methods for activating the inactive C–H bonds of simple alkanes to generate C–C bonds is more attractive but challenging due to the higher bond dissociation energy (BDE). In recent years, Li's group and others have reported C(sp^3^)–H bond functionalization reactions for the C(sp^2^)–C(sp^3^) bond formation between heteroaromatics and cycloalkanes.^6^ Despite the great progress that has been achieved through those radical-based processes, huge challenges still remain in the lack of selectivity. In addition, most of these examples involve the coupling of C(sp^2^)–H bonds in electron-rich aromatic compounds or acidic C–H bonds.

Recently, Chatani^7^ and other groups^8^ have combined these radical-based processes with direct C–H functionalization and cross-electrophile coupling. Chatani have successfully explored Ni(ii)-catalyzed oxidative coupling between C(sp^2^)–H in benzamides and C(sp^3^)–H in toluene derivatives (Scheme 1b). However, these approaches suffer from their own limitations on the substrate scope. Combined with the fact that radicals with a single electron have a strong tendency to form chemical bonds, we envisioned that auxiliary-assisted strategy might provide a solution for the selective aryl–alkyl oxidative coupling with an appropriate transition metal in a higher oxidation state. Recently, Co-catalyzed direct C–H functionalization of benzamide derivatives has received much interest owing to its unique reactivity and functional-group tolerance.^9^ Herein, using the auxiliary-assisted strategy, we report a cobalt-catalyzed oxidative coupling between C(sp^2^)–H bonds in benzamides and C(sp^3^)–H bonds in various alkanes, esters and toluene derivatives (Scheme 1c).^10^

## Results and Discussion

**Scheme 2:**
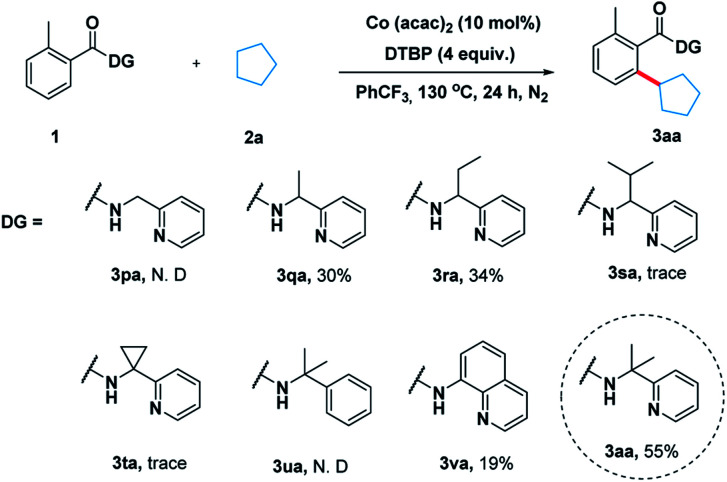
Effect of the directing groups.

To illustrate the feasibility of the aryl–alkyl oxidative coupling strategy, a series of benzamides containing bidentate directing groups were examined with cyclopentane 2a in the presence of a catalytic amount of Co(acac)_2_ at 130 °C under N_2_ protection (Scheme 2). Different directing groups have exhibited very different reactivity suggesting that the choice of directing group was extremely crucial for the reaction. Among them, the classical 2-(pyridin-2-yl)-propan-2-amine (PIP) seemed to be very encouraging, producing 55% yield. Subsequently, we carried out an extensive survey of reaction conditions using 1a as the model substrate.

After extensive optimization, Co(acac)_2_ was found to be a useful metal catalyst. Other metals like Cu or Ni were hardly effective (for the detailed optimization see ESI Table 1†). Next, a series of oxidants were then examined under the optimized conditions. The use of THBP, DCP, BPO and K_2_S_2_O_8_ as the oxidants was very disappointed. Thereafter, we elevated the temperature and found that 140 °C was turned out to be optimal temperature, producing the desired product 3aa in 72% yield (entry 10). When 10 equivalents of 2a were used the reaction yield decreased significantly (Table 1, entry 14).

**Table 1:** Optimization of reaction^a^

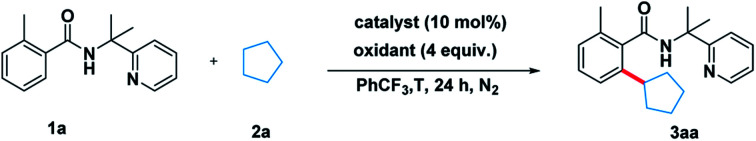				
Entry	Catalyst	Oxidant	Temp. (°C)	Yields^b^ (%)
1	Co(acac)_2_	DTBP	130	55
2	Co(OAc)_2_	DTBP	130	30
3	CoBr_2_	DTBP	130	N. D.
4	CoC_2_O_4_	DTBP	130	34
5	Co(acac)_2_	THBP	130	N. D.
6	Co(acac)_2_	DCP	130	Trace
7	Co(acac)_2_	BPO	130	N. D.
8	Co(acac)_2_	K_2_S_2_O_8_	130	N. D.
9	Co(acac)_2_	DDQ	130	N. D.
**10**	**Co(acac)**_**2**_	**DTBP**	**140**	**72**
11	Co(acac)_2_	DTBP	150	70
12^c^	Co(acac)_2_	DTBP	140	57
13^d^	Co(acac)_2_	DTBP	140	62
14^e^	Co(acac)_2_	DTBP	140	25

a Reaction conditions: 1a (0.2 mmol), 2a (1 mL), Co(acac)_2_ (0.02 mmol), DTBP (0.8 mmol) in PhCF_3_ (1 mL) under N_2_ at 140 °C for 24 h.

b Isolated yields.

c The reaction was carried under air.

d The catalyst (0.01 mmol) was used.

e 10 Equiv. of 2a was used.

**Scheme 3:**
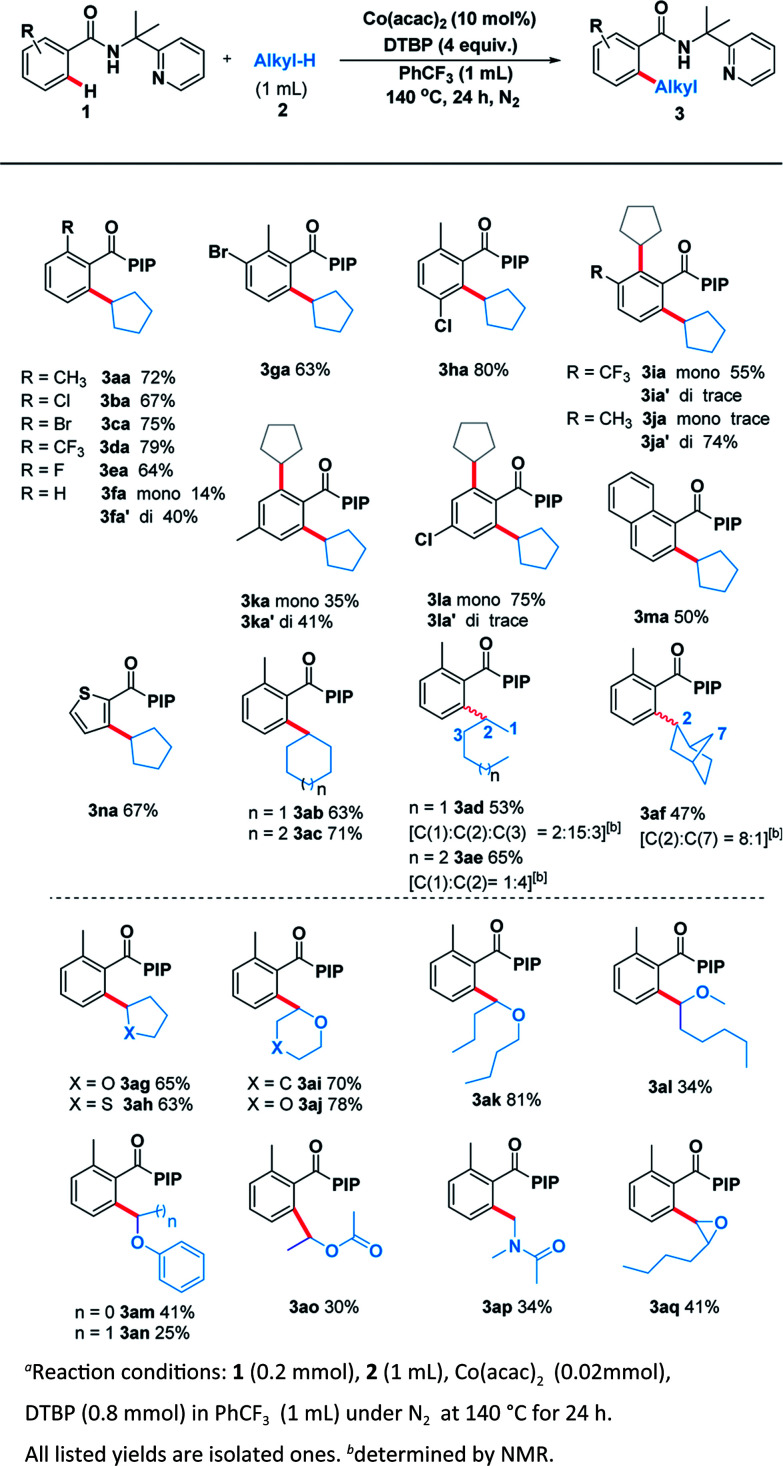
Scope of C(sp^3^)–H and C(sp^2^)–H partners^a^.

With the optimal system in hand, we examined the scope of benzamide substrates with various C(sp^3^)–H bonds as illustrated in Scheme 3. At first, the reaction of cyclopentane with diversely decorated benzamides proceeded smoothly to afford the corresponding arylation products in 30% to 81% yields. To our delight, the *o*-substituted benzamides gave the alkylated products (3aa–3ea) in moderate to good yields. Halogen substituents, such as bromine (3ca, 3ga) and chlorine (3ba, 3ha, 3la) were tolerated well. The reactions of *p*-substituted or non-substituted benzamides resulted in a mixture of mono- and di- alkylated products (3fa, 3ka), with the latter preferred. When the *m*-position was occupied by a trifluoromethyl group, only the mono-alkylated product (3ia) was isolated in 55% yield. At the same time, we probed its scope in the C–H functionalization of unactivated alkanes including cycloalkanes and open chain alkanes with benzamides. The ring size had slightly effect on the yield of the corresponding products (3ab, 3ac). However, linear alkanes including *n*-hexane and *n*-heptane afforded a mixture of regioisomers. For example, *n*-hexane reacted well to form the corresponding product 3ad as a mixture of positional isomers in the ratio C1/C2/C3 = 2 : 15 : 3 and a combined yield of 53%. Interestingly, when norbornane was used, 3af was isolated in a yield of 47% with a ratio of C2/C7 = 8 : 1. No reaction at the tertiary C(sp^3^)–H position (C3) of norbornane was observed, which was possibly due to the steric hindrance (Scheme 3).

In order to thoroughly outline the utility of this present method we probed the scope of this oxidative coupling protocol with a range of ethers which are easier to react because of their lower bond dissociation energy (BDE). Interestingly, the optimal conditions were compatible with cyclic ethers including tetrahydrofuran (3ag), tetrahydrothiophene (3ah), tetrahydropyran (3ai) and 1,4-dioxane (3aj). Besides cyclic ethers, acyclic ethers performed well under the same conditions, delivering both secondary (3ak, 3al, 3am, 3ao) and primary (3an) C–H functionalization products. To our delight, desired product (3ap) was obtained when using *N*,*N*-dimethylacetamide as the sp^3^ component. It is noteworthy that the asymmetric 2-butyloxirane (3aq) also underwent the target reaction only at methylene sites on account of steric effect.

**Scheme 4:**
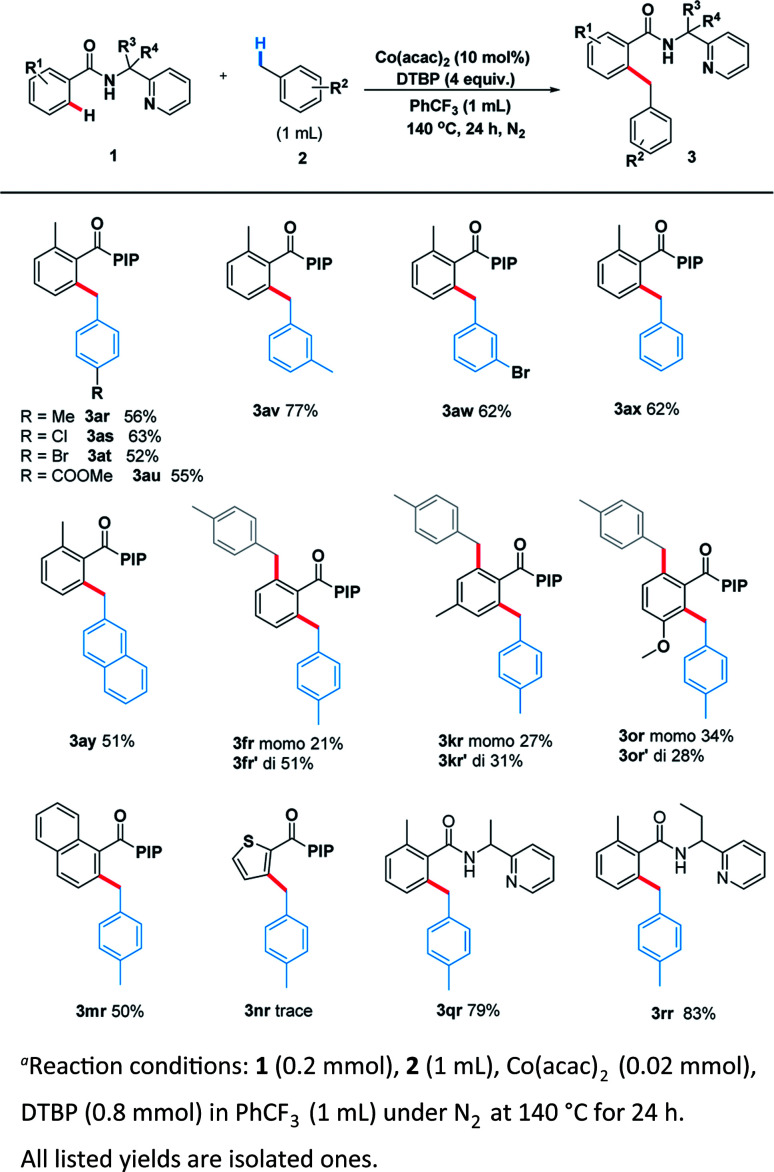
Scope of C(sp^3^)–H in toluene derivatives^a^.

Encouraged by these pleased results, we examined the scope of benzylic substrates with substrate 1 and the current catalytic system was suitable for a wide range of arylmethanes either electron-donating (3ar), electron-withdrawing (3as, 3at, 3au, 3aw) or sterically hindered group (3av) all delivered the desired products in synthetically useful yields (Scheme 4). In addition, 2-methylnaphthalene smoothly underwent the coupling process to afford the desired product (3ay). Naphthalene (3mr) also proceeded smoothly under the standard reaction condition. However, heterocycle (3nr) was not compatible with the reaction. Surprisingly, when the other benzamides containing different directing groups were used we found that *a*-ethyl-substituted substrates gave the best yield (3rr, 83%), suggesting the conformation of directing group has a great impact on the scope of substrates.

**Scheme 5:**
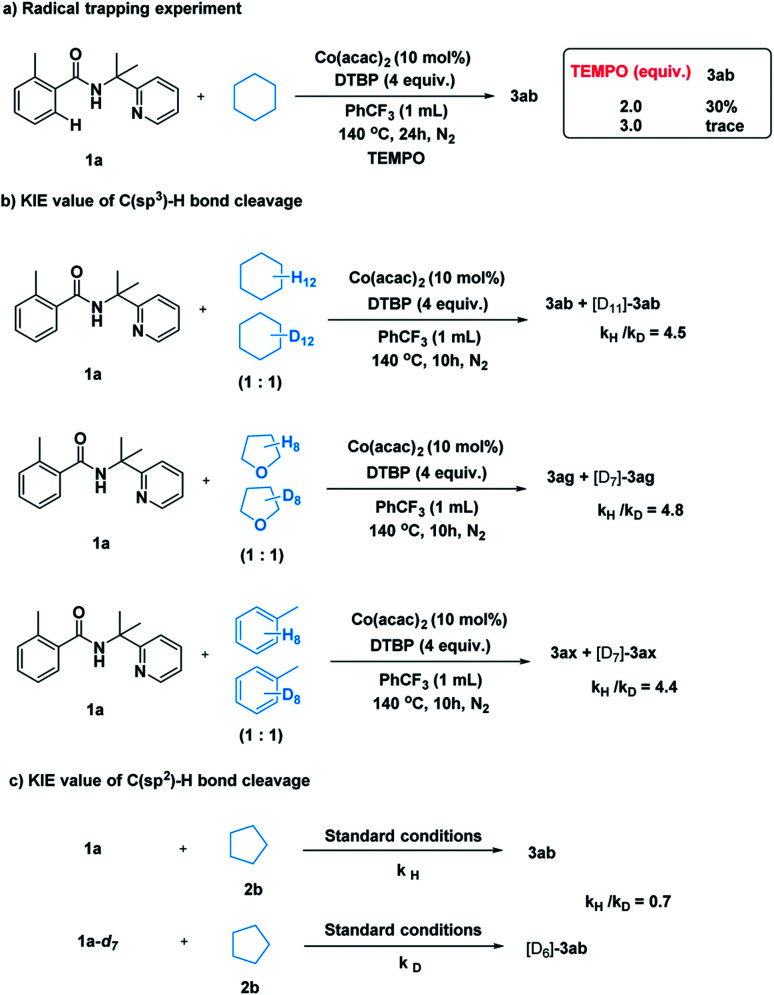
Mechanistic study.

After establishing the method, we tried to gain some insight into the catalytic pathway. A radical-trapping experiment was carried out using a commonly used radical trapping reagent TEMPO (Scheme 5a). The formation of the desired product was totally suppressed. This result suggested that a radical process is most likely to be involved in the C–H cleavage of simple alkane. Next, kinetic isotopic effect studies with separate kinetic experiments were performed to gain insights into the rate-determining step for this cross-coupling reaction. Both the C(sp^3^)–H bond cleavage of 2 and the C(sp^2^)–H bond cleavage of 1 were studied. A primary kinetic isotopic effect was observed for C(sp^3^)–H bond cleavage.

(Scheme 5b, *k*_H_/*k*_D_ = 4.5, 4.8, 4.4), while no obvious kinetic isotopic effect was observed for the C(sp^2^)–H cleavage (Scheme 5c, *k*_H_/*k*_D_ = 0.7), suggesting that C(sp^3^)–H bond cleavage was probably the rate-determining step in this transformation.

**Scheme 6:**
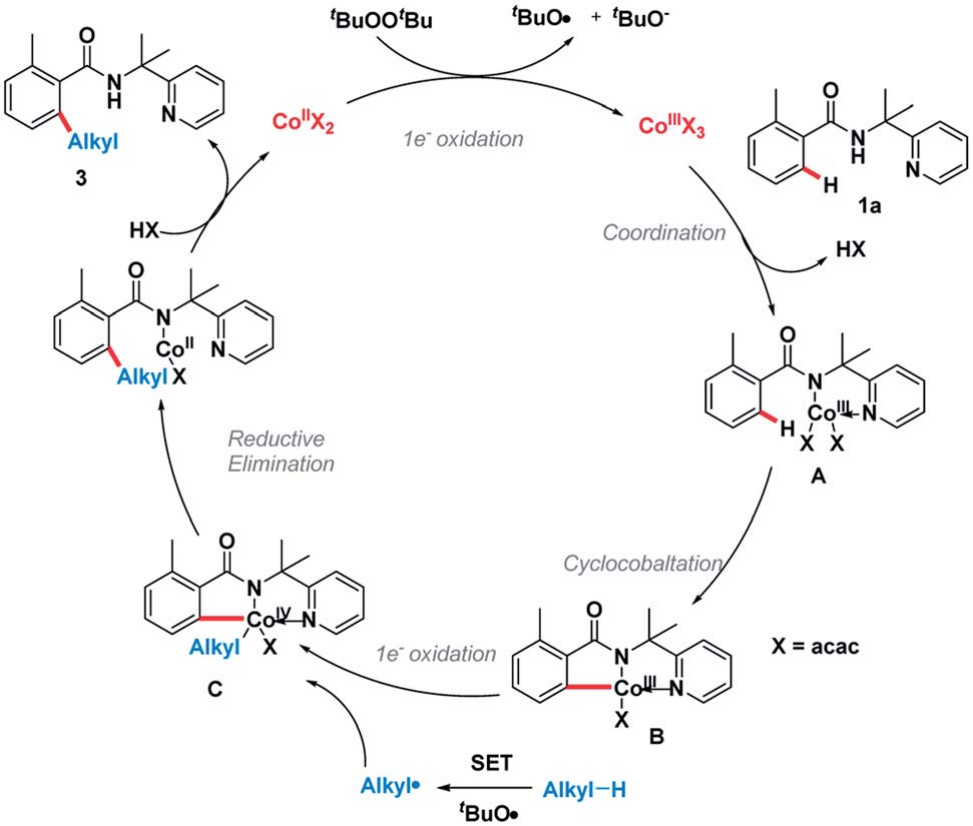
Plausible mechanism.

On the basis of the above observations, the plausible catalytic path is proposed (Scheme 6). Initially, the Co(ii) species is oxidized by DTBP through a single-electron transfer (SET) process giving the Co(iii) species and an alkoxy radical (^*t*^BuO). Next, amide 1a was coordinated to the Co(iii) species followed by ligand exchange forming intermediate A.^10,11^ The C(sp^2^)–H bond of A is broken, generating intermediate B. Meanwhile, an alkyl radical can be formed through hydrogen abstraction by *in situ* generated *tert*-butoxyl radical and the subsequent oxidative addition of this alkyl radical into intermediate B giving the complex C.^12^ Finally, the catalytic cycle is completed by reductive elimination of the cobalt complex C followed by protonation giving product 3 and Co(ii) species.

To further demonstrate the synthetic versatility of this new method, we successfully removed the DG under acidic conditions as other groups have demonstrated (see the ESI† for details).

## Conclusions

In summary, we developed a coordinating activation strategy to allow the radical oxidative simple alkanes, ethers and toluene derivatives with unactivated benzamides, utilizing a low-cost and easily available cobalt catalyst. This reaction represents the first example of cobalt-catalyzed radical oxidation of C(sp^3^)–H/C(sp^2^)–H without using alkyl halides or alkyl metal species. The success of this reaction hinges on the combination of the 2-(pyridin-2-yl)propan-2-amine (PIP) moiety and cobalt catalyst. The protocol outlined a straightforward approach to synthesize complex molecules of biological relevance.

## Conflicts of interest

There are no conflicts to declare.

## Supplementary Material

RA-008-C8RA01377B-s001
