# Supplementary material for: Cobalt-catalyzed C(sp3)–H/C(sp2)–H oxidative coupling between alkanes and benzamides
Source: RSC Adv. 2018 Apr 10;8(24):13454–8. doi: 10.1039/c8ra01377b (PMC9079668; doi:10.1039/c8ra01377b)
Supplement: RA-008-C8RA01377B-s001 [file RA-008-C8RA01377B-s001.pdf]

Electronic Supplementary Material (ESI) for RSC Advances.
This journal is © The Royal Society of Chemistry 2018

Supporting Information

Cobalt-Catalyzed C(sp^3)–H/C(sp^2)–H Oxidative Coupling between Alkanes and Benzamides

Shuangjie Li,[a,b,c,‡] Bao Wang,[b,c,d,‡] Guangyu Dong,[b,c,d] Chunpu Li,[b,c] and Hong Liu[b,c,*]

[a]School of Pharmacy, China Pharmaceutical University, Jiangsu, Nanjing 210009, China
[b]State Key Laboratory of Drug Research, Shanghai Institute of Materia Medica, Chinese Academy of Sciences, 555 Zu Chong Zhi Road, Shanghai, 201203, China.
[c]Key Laboratory of Receptor Research, Shanghai Institute of Materia Medica, Chinese Academy of Sciences, 555 Zu Chong Zhi Road, Shanghai, 201203, China
[d]School of Life Science and Technology, ShanghaiTech University, 100 Haike Road, Shanghai 201210, China.
E-mail: hliu@simm.ac.cn

Table of Contents

(I) General Methods 2

(II) Typical Synthesis Procedure and Characterization of 1 3

(III) Typical Synthesis Procedure and Characterization of 3 6

(IV) Optimization of the reaction conditions 31

(V) Kinetic Isotope Effect (KIE) Study 31

(VI) Cleavage of the PIP Directing Group 34

(VII) Copies of ^{1}H NMR and ^{13}C NMR Spectra 36

(VIII) References 92

(I) General Methods

Analytical thin layer chromatography (TLC) was HSGF 254 (0.15-0.2 mm thickness). Preparative thin layer chromatography (PTLC) was HSGF 254 (0.4-0.5 mm thickness). All products were characterized by their NMR and MS spectra. ^{1}H and ^{13}C NMR spectra were recorded on a 400 MHz, 500 MHz or 600 MHz instrument. Chemical shifts were reported in parts per million (ppm, δ) downfield from tetramethylsilane. Proton coupling patterns are described as singlet (s), doublet (d), triplet (t), quartet (q), multiplet (m), doublet of doublets (dd) and broad (br). High-resolution mass spectra (HRMS) were measured on Micromass Ultra Q-TOF spectrometer. All benzamides were prepared by following the same procedure as described in the literature[1]. Other reagents (chemicals) were purchased from Alfa Aesar, Across organics, TCI, J&K Chemicals, Energy Chemical and Adamas and used without further purification.

(II) Typical Synthesis Procedure and Characterization of 1

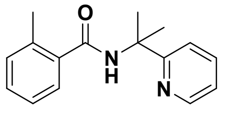
1a
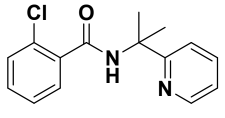
1b
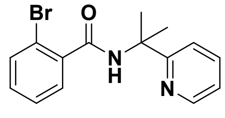
1c
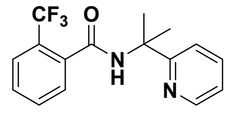
1d

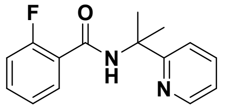
1e
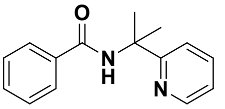
1f
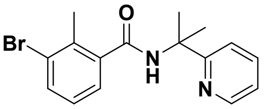
1g
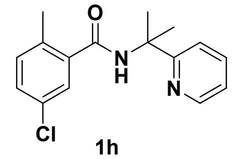
1h

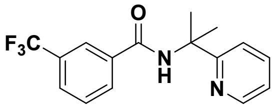
1i
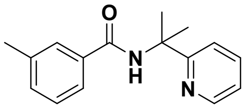
1j
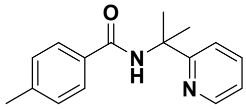
1k
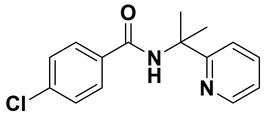
1l

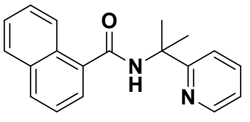
1m
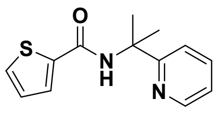
1n
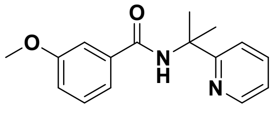
1o
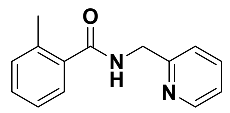
1p

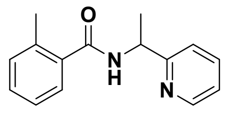
1q
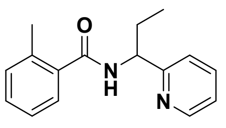
1r
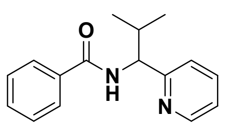
1s
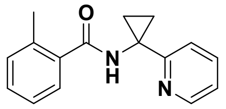
1t

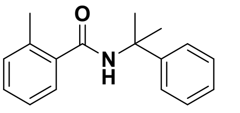
1u
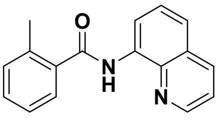
1v

1. Typical Synthesis Procedure of 1a-1v

Compounds **1a-1f, 1h-1p, 1u, 1v** were known compounds and were prepared according to literature.[1] **1g, 1q-1t** were prepared according to the following procedure.

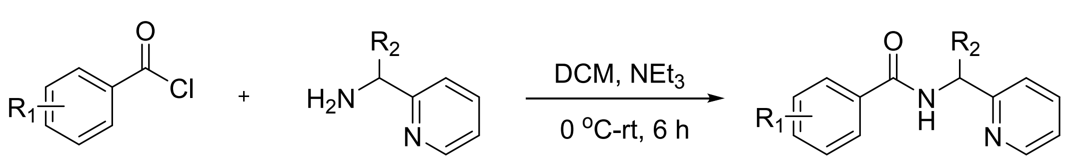

1

The acid chloride was dissolved in 5 mL anhydrous CH_2Cl_2 and added dropwise to a

20 mL anhydrous CH_2Cl_2 solution containing amine (5 mmol) and Et_3N (10 mmol) at 0 °C. After stirring for 6h at ambient temperature, the resulting mixture was washed with brine, dried over $MgSO_4$, filtered and concentrated under reduced pressure. The residue was purified by flash chromatography to give the white solid substrates.

2. Characterization of Starting Materials 1g, 1q-1t

3-bromo-2-methyl-*N*-(2-(pyridin-2-yl)propan-2-yl)benzamide (1g)

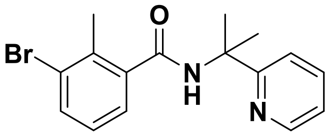

White solid (75%). R_f 0.54 (PE/EtOAc = 3/1). ^1H NMR (500 MHz, $CDCl_3$) δ 8.44 (dd, J = 4.9, 0.8 Hz, 1H), 8.16 (s, 1H), 7.64 (td, J = 7.9, 1.7 Hz, 1H), 7.51 (t, J = 8.2 Hz, 1H), 7.43 (dd, J = 8.0, 0.8 Hz, 1H), 7.38 (d, J = 8.1 Hz, 1H), 7.25 (dd, J = 7.5, 0.7 Hz, 1H), 7.11 (ddd, J = 7.4, 5.0, 0.8 Hz, 1H), 2.36 (s, 3H), 1.78 (s, 6H). ^{13}C NMR (126 MHz, $CDCl_3$) δ 168.9, 163.9, 147.4, 139.6, 137.7, 135.2, 133.4, 129.2, 127.0, 125.9, 122.2, 119.8, 57.1, 27.6, 20.6, 20.0; HRMS (ESI) m/z: calculated for $C_{16}H_{18}BrN_2O$ $[M + H]^+$: 333.0597, found: 333.0598.

2-methyl-*N*-(1-(pyridin-2-yl)ethyl)benzamide (1q)

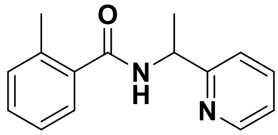

White solid (55%). R_f 0.23 (PE/EtOAc = 3/1). ^1H NMR (500 MHz, $CDCl_3$) δ 8.52 (d, J = 4.5 Hz, 1H), 7.68 (t, J = 7.7 Hz, 1H), 7.43 (d, J = 7.6 Hz, 1H), 7.29 (t, J = 7.6 Hz, 3H), 7.20 (t, J = 7.2 Hz, 3H), 5.33 (p, J = 6.8 Hz, 1H), 2.45 (s, 3H), 1.58 (d, J = 6.8 Hz, 3H). ^{13}C NMR (126 MHz, $CDCl_3$) δ 169.2, 160.8, 149.0, 136.9, 136.6, 136.1, 130.9, 129.7, 126.9, 125.7, 122.4, 121.6, 50.0, 22.9, 19.8; HRMS (ESI) m/z: calculated for $C_{15}H_{17}N_2O$ $[M + H]^+$: 241.1335, found: 241.1331.

2-methyl-*N*-(1-(pyridin-2-yl)propyl)benzamide (1r)

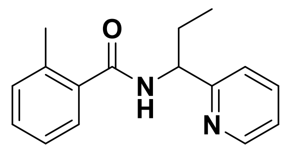

White solid (67%). R_f 0.43 (PE/EtOAc = 3/1). ^1H NMR (500 MHz, $CDCl_3$) δ 8.52 (d, *J* = 4.5 Hz, 1H), 7.70 – 7.61 (m, 1H), 7.41 (d, *J* = 7.5 Hz, 1H), 7.28 (t, *J* = 8.9 Hz, 2H), 7.22 – 7.13 (m, 4H), 5.20 (dd, *J* = 14.2, 6.9 Hz, 1H), 2.43 (s, 3H), 2.05 – 1.86 (m, 2H), 0.90 (t, *J* = 7.4 Hz, 3H). ^{13}C NMR (126 MHz, $CDCl_3$) δ 169.2, 160.8, 149.0, 136.9, 136.6, 136.1, 130.9, 129.7; HRMS (ESI) m/z: calculated for $C_{16}H_{19}N_2O$ $[M + H]^+$: 255.1492, found: 255.1491.

_N_-(2-methyl-1-(pyridin-2-yl)propyl)benzamide (1s)

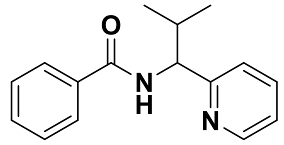

White solid (65%). R_f 0.41 (PE/EtOAc = 3/1). ^1H NMR (500 MHz, $CDCl_3$) δ 8.56 (d, *J* = 4.8 Hz, 1H), 7.87 – 7.84 (m, 2H), 7.67 – 7.62 (m, 1H), 7.59 (d, *J* = 8.0 Hz, 1H), 7.51 – 7.40 (m, 1H), 7.46 – 7.40 (m, 2H), 7.25 (d, *J* = 6.6 Hz, 1H), 7.19 (dd, *J* = 7.4, 4.9 Hz, 1H), 5.11 (dd, *J* = 8.5, 6.8 Hz, 1H), 2.25 (dq, *J* = 13.6, 6.8 Hz, 1H), 1.00 (d, *J* = 6.8 Hz, 3H), 0.87 (d, *J* = 6.8 Hz, 3H). ^{13}C NMR (126 MHz, $CDCl_3$) δ 166.9, 159.2, 149.0, 136.3, 134.8, 131.3, 128.5, 127.1, 123.2, 122.3, 59.2, 34.3, 19.2, 18.7; HRMS (ESI) m/z: calculated for $C_{16}H_{19}N_2O$ $[M + H]^+$: 255.1492, found: 255.1484.

2-methyl-_N_-(1-(pyridin-2-yl)cyclopropyl)benzamide (1t)

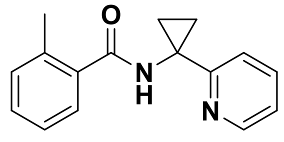

White solid (46%). R_f 0.25 (PE/EtOAc = 3/1). ^1H NMR (500 MHz, $CDCl_3$) δ 8.48 (d, *J* = 4.6 Hz, 1H), 7.63 (td, *J* = 7.6, 1.5 Hz, 1H), 7.46 (dd, *J* = 7.7, 3.0 Hz, 2H), 7.33 (dd, *J* = 10.7, 4.3 Hz, 1H), 7.26 – 7.20 (m, 2H), 7.09 (dd, *J* = 7.1, 5.2 Hz, 1H), 6.62 (s, 1H),

2.49 (s, 3H), 1.74 (q, J = 4.7 Hz, 2H), 1.41 (q, J = 4.7 Hz, 2H). ^{13}C NMR (126 MHz, $CDCl_3$) δ 170.7, 160.6, 149.1, 136.5, 136.4, 135.9, 131.3, 130.2, 126.7, 125.8, 120.9, 119.3, 36.7, 20.0, 19.3; HRMS (ESI) m/z: calculated for $C_{16}H_{17}N_2O$ [M + H]$^+$: 253.1335, found: 253.1335.

(III) Typical Synthesis Procedure and Characterization of 3

1. The Co-Catalyzed Synthesis of 3

To a reaction tube was add **1a** (51 mg, 0.2 mmol), cyclopentane **2a** (1.0 mL), di-tert-butyl peroxide DTBP (0.146 mL, 0.8 mmol), $Co(acac)_2$ (7.0 mg, 10 mmol%) and benzotrifluoride (1.0 mL), then was evacuated and purged with N_2 three times. The solution was kept at 140 ^oC for 24h. After the solution was cooled to room temperature, the crude mixture was purified by silica gel column chromatography [ethyl acetate/petroleum ether (v/v, 1:6) as eluent] to give a corresponding product **3aa**.

2-cyclopentyl-6-methyl-*N*-(2-(pyridin-2-yl)propan-2-yl)benzamide (3aa)

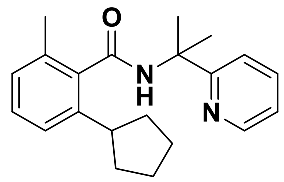

Yellow oil (48.5 mg, 72%). R_f 0.43 (PE/EtOAc = 3/1). ^{1}H NMR (400 MHz, $CDCl_3$) δ 8.45 – 8.41 (m, 1H), 8.03 (s, 1H), 7.73 (td, J = 8.0, 1.8 Hz, 1H), 7.46 (d, J = 8.1 Hz, 1H), 7.25 – 7.15 (m, 3H), 7.05 – 7.00 (m, 1H), 3.25 – 3.10 (m, 1H), 2.37 (s, 3H), 2.13 – 2.06 (m, 2H), 1.92 (s, 6H), 1.82 – 1.72 (m, 2H), 1.67 – 1.53 (m, 4H). ^{13}C NMR (126 MHz, $CDCl_3$) δ 169.5, 164.3, 147.5, 143.0, 138.7, 137.1, 134.0, 128.5, 127.4, 123.3, 121.9, 119.5, 57.1, 42.5, 27.4, 25.9, 19.3; HRMS (ESI) m/z: calculated for $C_{22}H_{29}N_2O$ [M + H]$^+$: 337.2274, found: 337.2272.

2-chloro-6-cyclopentyl-*N*-(2-(pyridin-2-yl)propan-2-yl)benzamide (3ba)

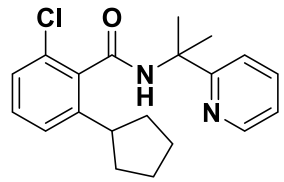

Yellow oil (46.0 mg, 67%). R_f 0.21 (PE/EtOAc = 3/1). ^{1}H NMR (400 MHz, $CDCl_3$) δ 8.46 (d, *J* = 4.6 Hz, 1H), 8.13 (s, 1H), 7.75 (td, *J* = 7.9, 1.6 Hz, 1H), 7.50 (d, *J* = 8.1 Hz, 1H), 7.29 – 7.24 (m, 2H), 7.24 – 7.17 (m, 2H), 3.30 – 3.13 (m, 1H), 2.15 – 2.05 (m, 2H), 1.93 (s, 6H), 1.80 (s, 2H), 1.67 – 1.55 (m, 4H). ^{13}C NMR (126 MHz, $CDCl_3$) δ 166.4, 164.1, 147.4, 145.9, 137.6, 137.2, 130.5, 129.6, 126.7, 124.5, 121.9, 119.5, 57.4, 42.7, 35.4, 27.3, 25.8; HRMS (ESI) m/z: calculated for $C_{20}H_{24}ClN_2O$ $[M + H]^+$: 343.1572, found: 343.1575.

2-bromo-6-cyclopentyl-*N*-(2-(pyridin-2-yl)propan-2-yl)benzamide (3ca)

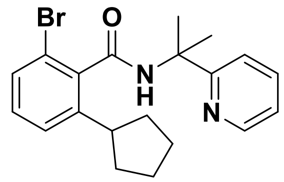

Yellow oil (58.0 mg, 75%). R_f 0.23 (PE/EtOAc = 3/1). ^{1}H NMR (400 MHz, $CDCl_3$) δ 8.44 (dd, *J* = 4.9, 0.8 Hz, 1H), 8.11 (s, 1H), 7.73 (td, *J* = 8.1, 1.6 Hz, 1H), 7.48 (d, *J* = 8.1 Hz, 1H), 7.41 – 7.35 (m, 1H), 7.28 (d, *J* = 7.9 Hz, 1H), 7.21 – 7.15 (m, 2H), 3.27 – 3.11 (m, 1H), 2.13 – 2.03 (m, 2H), 1.92 (s, 6H), 1.78 (d, *J* = 2.8 Hz, 2H), 1.60 (s, 4H). ^{13}C NMR (126 MHz, $CDCl_3$) δ 167.1, 164.1, 147.4, 146.0, 139.5, 137.2, 129.9, 129.9, 125.0, 121.9, 119.5, 57.4, 42.9, 27.3, 25.8; HRMS (ESI) m/z: calculated for $C_{20}H_{24}BrN_2O$ $[M + H]^+$: 387.1067, found: 387.1072.

2-cyclopentyl-*N*-(2-(pyridin-2-yl)propan-2-yl)-6-(trifluoromethyl)benzamide (3da)

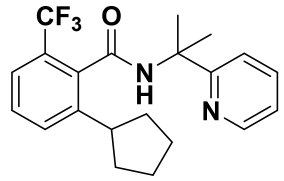

Yellow oil (59.5 mg, 79%). R_f 0.32 (PE/EtOAc = 3/1). ^1H NMR (400 MHz, $CDCl_3$) δ 8.41 (dd, *J* = 4.1, 0.8 Hz, 1H), 8.27 (s, 1H), 7.72 (td, *J* = 7.9, 1.7 Hz, 1H), 7.55 (d, *J* = 7.8 Hz, 1H), 7.49 (d, *J* = 7.3 Hz, 1H), 7.47 – 7.40 (m, 2H), 7.19 – 7.14 (m, 1H), 3.34 – 3.19 (m, 1H), 2.15 – 2.03 (m, 2H), 1.89 (d, *J* = 18.2 Hz, 6H), 1.80 (s, 2H), 1.68 – 1.51 (m, 4H). ^{13}C NMR (126 MHz, $CDCl_3$) δ 166.3, 164.0, 147.3, 145.5, 137.2, 136.2, 130.1, 128.8, 126.7 (q, *J* = 30.07, 33.50), 124.0 (q, *J* = 275.19, 245.41), 123.4 (q, *J* = 5.32, 6.23), 122.0, 119.5, 57.3, 42.0, 36.1, 35.2, 27.0, 26.9, 26.0, 25.8; HRMS (ESI) m/z: calculated for $C_{21}H_{24}F_3N_2O$ $[M + H]^+$: 377.1835, found: 377.1827.

2-cyclopentyl-6-fluoro-N-(2-(pyridin-2-yl)propan-2-yl)benzamide (3ea)

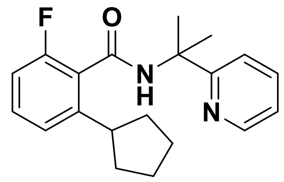

Yellow oil (41.8 mg, 64%). R_f 0.53 (PE/EtOAc = 3/1). ^1H NMR (400 MHz, $CDCl_3$) δ 8.45 (d, *J* = 4.8 Hz, 1H), 8.07 (s, 1H), 7.73 (td, *J* = 7.9, 1.7 Hz, 1H), 7.48 (d, *J* = 8.1 Hz, 1H), 7.33 – 7.24 (m, 1H), 7.18 (dd, *J* = 7.3, 5.0 Hz, 1H), 7.12 (d, *J* = 7.8 Hz, 1H), 6.91 (t, *J* = 8.6 Hz, 1H), 3.31 – 3.17 (m, 1H), 2.12 – 2.01 (m, 2H), 1.89 (s, 6H), 1.82 – 1.74 (m, 2H), 1.66 – 1.55 (m, 4H). ^{13}C NMR (126 MHz, $CDCl_3$) δ 164.6, 164.1, 159.0 (d, *J* = 252.14), 147.5, 146.8, 146.7, 137.2, 130.1, 130.0, 126.5 (d, *J* = 18.05), 121.9, 121.7 (d, *J* = 32.24), 119.5, 112.7 (d, *J* = 22.76), 57.4, 42.1, 42.1, 35.1, 27.5, 25.8; HRMS (ESI) m/z: calculated for $C_{20}H_{25}FN_2O$ $[M + H]^+$: 327.1867, found: 327.1870.

2-cyclopentyl-*N*-(2-(pyridin-2-yl)propan-2-yl)benzamide (3fa)

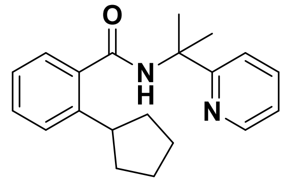

Yellow oil (41.8 mg, 64%). R_f 0.53 (PE/EtOAc = 3/1). ^1H NMR (600 MHz, $CDCl_3$) δ 8.46 (d, *J* = 4.5 Hz, 1H), 8.03 (s, 1H), 7.73 (td, *J* = 7.9, 1.7 Hz, 1H), 7.46 (d, *J* = 8.1

Hz, 1H), 7.40 (d, *J* = 7.6 Hz, 1H), 7.38 – 7.33 (m, 2H), 7.22 – 7.15 (m, 2H), 3.49 – 3.39 (m, 1H), 2.15 – 2.08 (m, 2H), 1.89 (s, 6H), 1.82 – 1.75 (m, 2H), 1.67 – 1.57 (m, 4H) ; HRMS (ESI) m/z: calculated for $C_{20}H_{25}N_2O$ [M + H]$^+$: 309.1961, found: 309.1955.

2,6-dicyclopentyl-*N*-(2-(pyridin-2-yl)propan-2-yl)benzamide (3fa')

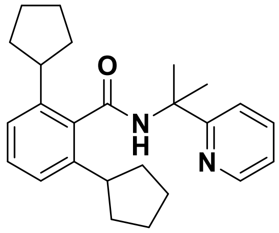

Yellow oil (41.8 mg, 64%). R_f 0.63 (PE/EtOAc = 3/1). ^{1}H NMR (400 MHz, $CDCl_3$) δ 8.43 (d, *J* = 4.5 Hz, 1H), 8.00 (s, 1H), 7.73 (t, *J* = 7.1 Hz, 1H), 7.47 (d, *J* = 8.1 Hz, 1H), 7.34 – 7.27 (m, 1H), 7.18 (t, *J* = 8.7 Hz, 3H), 3.16 (p, *J* = 8.8 Hz, 2H), 2.13 – 2.02 (m, 4H), 1.91 (s, 6H), 1.82 – 1.73 (m, 4H), 1.68 – 1.54 (m, 8H). ^{13}C NMR (126 MHz, $CDCl_3$) δ 169.7, 164.2, 147.4, 142.8, 138.5, 137.1, 128.8, 123.3, 121.9, 119.5, 57.1, 42.6, 36.1, 35.0, 27.3, 26.0, 25.8 ; HRMS (ESI) m/z: calculated for $C_{25}H_{33}N_2O$ [M + H]$^+$: 377.2587, found: 377.2581.

3-bromo-6-cyclopentyl-2-methyl-*N*-(2-(pyridin-2-yl)propan-2-yl)benzamide (3ga)

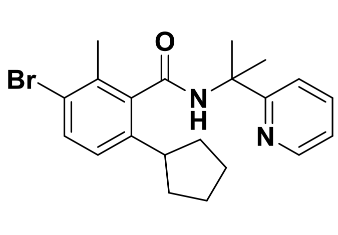

Yellow oil (50.4 mg, 63%). R_f 0.5 (PE/EtOAc = 3/1). ^{1}H NMR (400 MHz, $CDCl_3$) δ 8.44 (d, *J* = 4.4 Hz, 1H), 8.12 (s, 1H), 7.77 (t, *J* = 7.7 Hz, 1H), 7.48 (t, *J* = 7.8 Hz, 2H), 7.25 – 7.18 (m, 1H), 7.04 (d, *J* = 8.5 Hz, 1H), 3.09 (dd, *J* = 16.8, 9.0 Hz, 1H), 2.41 (s, 3H), 2.09 – 1.99 (m, 2H), 1.91 (d, *J* = 7.2 Hz, 6H), 1.77 (s, 2H), 1.63 – 1.49 (m, 4H). ^{13}C NMR (126 MHz, $CDCl_3$) δ 168.6, 163.9, 147.2, 142.5, 140.0, 137.5, 133.6, 132.6,

125.1, 122.6, 122.1, 119.6, 57.1, 42.4, 35.9, 35.0, 27.4, 27.2, 25.9, 25.8, 19.9; HRMS (ESI) m/z: calculated for $C_{21}H_{26}BrN_2O$ [M + H]$^+$: 401.1223, found: 401.1228.

3-chloro-2-cyclopentyl-6-methyl-*N*-(2-(pyridin-2-yl)propan-2-yl)benzamide (3ha)

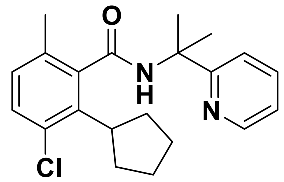

Yellow oil (57.0 mg, 80%). R_f 0.52 (PE/EtOAc = 3/1). ^{1}H NMR (500 MHz, $CDCl_3$) δ 8.45 – 8.42 (m, 1H), 8.06 (s, 1H), 7.73 (td, *J* = 7.9, 1.8 Hz, 1H), 7.45 (d, *J* = 8.1 Hz, 1H), 7.22 (d, *J* = 8.1 Hz, 1H), 7.21 – 7.16 (m, 1H), 6.96 (dd, *J* = 8.1, 0.6 Hz, 1H), 3.44 – 3.26 (m, 1H), 2.32 (s, 3H), 2.25 – 2.16 (m, 2H), 1.89 (d, *J* = 8.0 Hz, 10H), 1.67 – 1.50 (m, 2H). ^{13}C NMR (126 MHz, $CDCl_3$) δ 168.7, 164.0, 147.5, 141.2, 138.4, 137.2, 133.1, 131.1, 131.0, 128.9, 122.0, 119.4, 57.1, 42.9, 31.3, 30.5, 27.3, 27.1, 26.8, 19.0; HRMS (ESI) m/z: calculated for $C_{21}H_{26}ClN_2O$ [M + H]$^+$: 357.1728, found: 357.1723.

2-cyclopentyl-*N*-(2-(pyridin-2-yl)propan-2-yl)-5-(trifluoromethyl)benzamide (3ia)

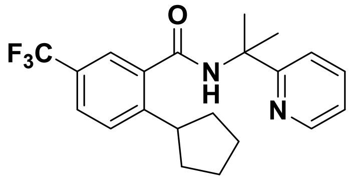

Yellow oil (41.5 mg, 55%). R_f 0.33 (PE/EtOAc = 3/1). ^{1}H NMR (400 MHz, $CDCl_3$) δ 8.45 (dd, *J* = 4.8, 0.7 Hz, 1H), 8.25 (s, 1H), 7.77 – 7.70 (m, 1H), 7.64 (s, 1H), 7.59 (d, *J* = 8.3 Hz, 1H), 7.46 (dd, *J* = 8.1, 5.5 Hz, 2H), 7.23 – 7.15 (m, 1H), 3.53 – 3.37 (m, 1H), 2.21 – 2.07 (m, 2H), 1.89 (s, 6H), 1.84 – 1.76 (m, 2H), 1.68 – 1.56 (m, 4H). ^{13}C NMR (126 MHz, $CDCl_3$) δ 168.4, 163.9, 148.4, 147.5, 138.7, 137.2, 127.7 (q, *J* = 32.05, 35.53), 127.1, 126.0 (d, *J* = 3.64), 125.1, 123.6 (d, *J* = 3.63), 122.9, 122.0, 119.4, 57.2, 41.9, 35.1, 27.3, 25.8; HRMS (ESI) m/z: calculated for $C_{21}H_{24}F_3N_2O$ [M + H]$^+$: 377.1835, found: 377.1837.

2,6-dicyclopentyl-3-methyl-*N*-(2-(pyridin-2-yl)propan-2-yl)benzamide (3ja')

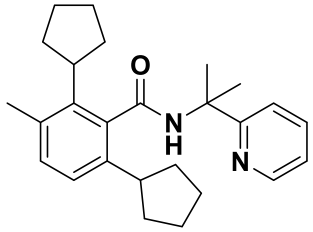

Yellow oil (115.6 mg, 74%). R_f 0.55 (PE/EtOAc = 3/1). ^{1}H NMR (600 MHz, $CDCl_3$) δ 8.45 – 8.40 (m, 1H), 7.94 (s, 1H), 7.72 (td, *J* = 8.0, 1.5 Hz, 1H), 7.46 (d, *J* = 8.1 Hz, 1H), 7.18 (dd, *J* = 6.9, 5.1 Hz, 1H), 7.13 – 7.07 (m, 2H), 3.34 (p, *J* = 9.6 Hz, 1H), 3.17 – 3.07 (m, 1H), 2.37 (s, 3H), 2.06 – 2.01 (m, 2H), 1.98 – 1.90 (m, 4H), 1.89 (d, *J* = 5.0 Hz, 6H), 1.85 – 1.80 (m, 2H), 1.79 – 1.73 (m, 3H), 1.68 – 1.59 (m, 4H), 1.59 – 1.52 (m, 3H). ^{13}C NMR (151 MHz, $CDCl_3$) δ 170.49, 164.22, 147.46, 140.53, 139.75, 138.73, 137.03, 133.81, 132.32, 123.42, 121.85, 119.43, 57.06, 43.11, 42.32, 36.16, 34.88, 32.22, 31.18, 27.20, 27.13, 27.02, 26.76, 25.88, 25.69, 20.58. HRMS (ESI) m/z: calculated for $C_{26}H_{35}N_2O$ $[M + H]^+$: 391.2744, found: 391.2731.

2-cyclopentyl-4-methyl-*N*-(2-(pyridin-2-yl)propan-2-yl)benzamide (3ka)

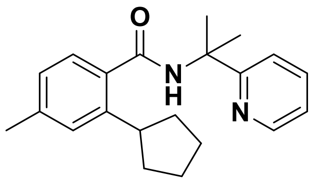

Yellow oil (22.6 mg, 35%). R_f 0.43 (PE/EtOAc = 3/1). ^{1}H NMR (400 MHz, $CDCl_3$) δ 8.46 (d, *J* = 4.2 Hz, 1H), 7.98 (s, 1H), 7.72 (td, *J* = 7.9, 1.6 Hz, 1H), 7.46 (d, *J* = 8.1 Hz, 1H), 7.32 (d, *J* = 7.7 Hz, 1H), 7.21 – 7.14 (m, 2H), 7.00 (d, *J* = 7.6 Hz, 1H), 3.51 – 3.38 (m, 1H), 2.35 (s, 3H), 2.16 – 2.03 (m, 2H), 1.88 (s, 6H), 1.83 – 1.74 (m, 2H), 1.67 – 1.56 (m, 4H). ^{13}C NMR (126 MHz, $CDCl_3$) δ 170.0, 164.4, 147.5, 144.2, 139.2, 137.1, 135.6, 127.1, 126.8, 126.1, 121.9, 119.5, 57.0, 41.7, 35.1, 27.5, 25.8, 21.5; HRMS (ESI) m/z: calculated for $C_{21}H_{27}N_2O$ $[M + H]^+$: 323.2118, found: 323.2110 .

2,6-dicyclopentyl-4-methyl-*N*-(2-(pyridin-2-yl)propan-2-yl)benzamide (3ka')

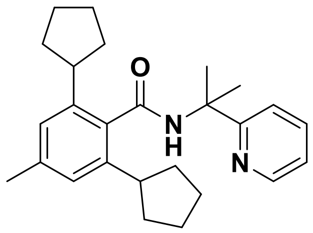

Yellow oil (26.4 mg, 41%). R_f 0.2 (PE/EtOAc = 3/1). ^{1}H NMR (500 MHz, $CDCl_3$) δ 8.42 (d, *J* = 3.6 Hz, 1H), 7.94 (s, 1H), 7.73 (s, 1H), 7.46 (d, *J* = 7.2 Hz, 1H), 7.18 (s, 1H), 6.97 (s, 2H), 3.19 – 3.09 (m, 2H), 2.33 (s, 3H), 2.05 (s, 4H), 1.89 (s, 6H), 1.77 (s, 4H), 1.64 – 1.54 (m, 8H). ^{13}C NMR (126 MHz, $CDCl_3$) δ 170.0, 164.3, 147.5, 142.7, 138.2, 136.9, 136.1, 124.0, 121.8, 119.4, 57.1, 42.5, 36.1, 34.9, 27.3, 25.9, 25.8, 21.7; HRMS (ESI) m/z: calculated for $C_{26}H_{35}N_2O$ $[M + H]^+$: 391.2744, found: 391.2732.

4-chloro-2-cyclopentyl-*N*-(2-(pyridin-2-yl)propan-2-yl)benzamide (3la)

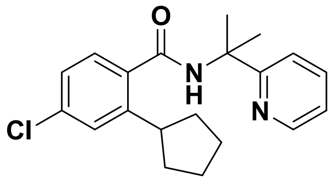

Yellow oil (51.4 mg, 75%). R_f 0.21 (PE/EtOAc = 3/1). ^{1}H NMR (500 MHz, $CDCl_3$) δ 8.48 – 8.44 (m, 1H), 8.11 (s, 1H), 7.75 (td, *J* = 7.9, 1.7 Hz, 1H), 7.50 (d, *J* = 8.1 Hz, 1H), 7.30 – 7.25 (m, 2H), 7.24 – 7.17 (m, 2H), 3.27 – 3.15 (m, 1H), 2.16 – 2.04 (m, 2H), 1.93 (s, 6H), 1.84 – 1.76 (m, 2H), 1.67 – 1.56 (m, 4H). ^{13}C NMR (126 MHz, $CDCl_3$) δ 166.4, 164.1, 147.4, 145.9, 137.6, 137.2, 130.5, 129.6, 126.7, 124.5, 121.9, 119.5, 57.4, 42.7, 35.4, 27.3, 25.8; HRMS (ESI) m/z: calculated for $C_{20}H_{24}ClN_2O$ $[M + H]^+$: 343.1572, found: 343.1565.

2-cyclopentyl-*N*-(2-(pyridin-2-yl)propan-2-yl)-1-naphthamide (3ma)

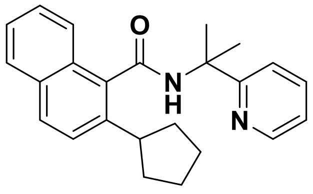

Yellow oil (36.0 mg, 50%). R_f 0.43 (PE/EtOAc = 3/1). ^{1}H NMR (400 MHz, $CDCl_3$) δ 8.38 (dd, J = 4.8, 0.7 Hz, 1H), 8.26 (s, 1H), 7.99 (d, J = 8.2 Hz, 1H), 7.83 (dd, J = 14.1, 8.3 Hz, 2H), 7.75 – 7.67 (m, 1H), 7.54 – 7.40 (m, 4H), 7.18 – 7.11 (m, 1H), 3.42 (dd, J = 16.6, 8.3 Hz, 1H), 2.16 (s, 2H), 2.03 (d, J = 21.4 Hz, 6H), 1.88 (d, J = 3.5 Hz, 2H), 1.72 (d, J = 12.8 Hz, 4H). ^{13}C NMR (126 MHz, $CDCl_3$) δ 169.1, 164.1, 147.5, 140.1, 137.1, 134.7, 131.8, 130.2, 128.9, 127.7, 126.6, 125.3, 125.1, 124.2, 121.9, 119.4, 57.3, 42.8, 35.8, 34.9, 27.6, 27.4, 26.3; HRMS (ESI) m/z: calculated for $C_{24}H_{27}N_2O$ $[M+H]^+$: 359.2118, found: 359.2110.

3-cyclopentyl-*N*-(2-(pyridin-2-yl)propan-2-yl)thiophene-2-carboxamide (3na)

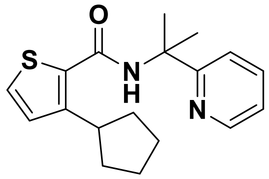

Yellow oil (42.2 mg, 67%). R_f 0.33 (PE/EtOAc = 3/1). ^{1}H NMR (400 MHz, $CDCl_3$) δ 8.51 (d, J = 3.8 Hz, 2H), 7.75 (td, J = 7.8, 1.6 Hz, 1H), 7.45 (d, J = 8.1 Hz, 1H), 7.27 (d, J = 4.5 Hz, 1H), 7.21 (dd, J = 7.3, 4.9 Hz, 1H), 7.01 (d, J = 5.2 Hz, 1H), 3.88 – 3.71 (m, 1H), 2.21 – 2.11 (m, 2H), 1.86 (s, 6H), 1.82 – 1.77 (m, 2H), 1.71 – 1.59 (m, 4H). ^{13}C NMR (126 MHz, $CDCl_3$) δ 165.9, 163.9, 150.2, 148.8, 138.7, 134.9, 129.2, 127.8, 123.4, 120.9, 58.7, 40.9, 36.1, 29.0, 27.1; HRMS (ESI) m/z: calculated for $C_{18}H_{23}N_2OS$ $[M+H]^+$: 315.1526, found: 315.1522.

2-cyclohexyl-6-methyl-*N*-(2-(pyridin-2-yl)propan-2-yl)benzamide (3ab)

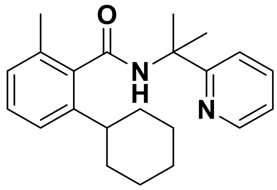

Yellow oil (42.5 mg, 63%). R_f 0.63 (PE/EtOAc = 3/1). ^{1}H NMR (400 MHz, MeOD) δ 8.82 (s, 1H), 8.55 – 8.46 (m, 1H), 7.83 (td, J = 8.0, 1.7 Hz, 1H), 7.67 (d, J = 8.1 Hz, 1H), 7.32 – 7.25 (m, 1H), 7.22 (t, J = 7.6 Hz, 1H), 7.16 (d, J = 7.7 Hz, 1H), 7.03 (d, J

= 7.3 Hz, 1H), 2.77 – 2.65 (m, 1H), 2.34 (s, 3H), 2.05 – 1.87 (m, 2H), 1.80 (s, 9H), 1.57 – 1.41 (m, 3H), 1.28 – 1.35 (m, 2H). ^{13}C NMR (126 MHz, MeOD) δ 171.0, 164.6, 147.6, 144.1, 136.9, 136.9, 133.8, 128.5, 127.1, 123.3, 121.7, 120.1, 57.2, 41.0, 34.2, 26.8, 26.4, 25.8, 18.0; HRMS (ESI) m/z: calculated for $C_{22}H_{29}N_2O$ [M + H]$^+$: 337.2274, found: 337.2279.

2-cycloheptyl-6-methyl-*N*-(2-(pyridin-2-yl)propan-2-yl)benzamide(3ac)

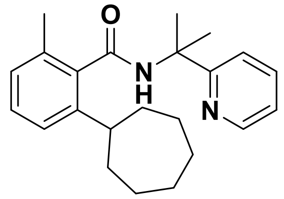

Yellow oil (49.8 mg, 71%). R_f 0.2 (PE/EtOAc = 3/1). ^{1}H NMR (400 MHz, $CDCl_3$) δ 8.46 – 8.37 (m, 1H), 8.04 (s, 1H), 7.73 (td, *J* = 8.0, 1.8 Hz, 1H), 7.47 (d, *J* = 8.1 Hz, 1H), 7.25 – 7.16 (m, 2H), 7.12 (d, *J* = 7.7 Hz, 1H), 7.01 (d, *J* = 7.4 Hz, 1H), 2.97 – 2.85 (m, 1H), 2.36 (s, 3H), 1.93 (s, 8H), 1.80 – 1.39 (m, 10H). ^{13}C NMR (126 MHz, $CDCl_3$) δ 169.3, 164.3, 147.5, 146.3, 137.2, 137.1, 134.0, 128.5, 127.1, 123.6, 121.9, 119.5, 57.0, 42.8, 27.8, 27.5, 19.3; HRMS (ESI) m/z: calculated for $C_{23}H_{31}N_2O$ [M + H]$^+$: 351.2431, found: 351.2428.

2-(hexan-2-yl)-6-methyl-*N*-(2-(pyridin-2-yl)propan-2-yl)benzamide (3ad)

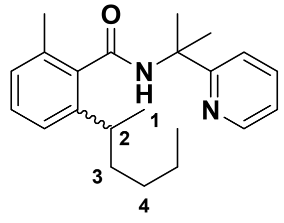

Yellow oil (37.2 mg, 53%). R_f 0.3 (PE/EtOAc = 3/1). ^{1}H NMR (400 MHz, $CDCl_3$) δ 8.41 (d, *J* = 4.1 Hz, 1H), 8.08 (d, *J* = 11.5 Hz, 1H), 7.79 – 7.67 (m, 1H), 7.46 (d, *J* = 8.1 Hz, 1H), 7.22 (d, *J* = 7.6 Hz, 1H), 7.18 (dd, *J* = 6.7, 4.9 Hz, 1H), 7.09 (dd, *J* = 18.0, 7.5 Hz, 1H), 7.03 (d, *J* = 7.4 Hz, 1H), 3.00 – 2.60 (m, 1H), 2.37 (s, 3H), 1.92 (s, 6H), 1.67 – 1.48 (m, 2H), 1.27 – 1.14 (m, 7H), 0.83 – 0.73 (m, 3H). ^{13}C NMR (126

MHz, $CDCl_3$) δ 169.3, 169.2, 164.3, 147.4, 147.4, 144.4, 139.3, 138.5, 138.4, 137.1, 134.3, 134.0, 128.5, 128.3, 128.2, 127.4, 127.3, 127.3, 126.5, 123.2, 123.1, 121.9, 119.5, 119.4, 57.0, 38.1, 36.0, 33.4, 32.0, 31.7, 30.2, 29.5, 27.4, 27.4, 27.3, 23.0, 22.8, 22.5, 19.4, 19.3, 19.2, 14.0, 14.0; HRMS (ESI) m/z: calculated for $C_{23}H_{31}N_2O$ $[M + H]^+$: 351.2431, found: 351.2428.

2-(heptan-2-yl)-6-methyl-*N*-(2-(pyridin-2-yl)propan-2-yl)benzamide (3ae)

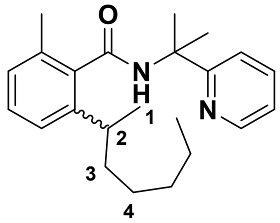

Yellow oil (45.9 mg, 65%). R_f 0.42 (PE/EtOAc = 3/1). ^1H NMR (400 MHz, $CDCl_3$) δ 8.40 (d, J = 4.6 Hz, 1H), 8.11 (d, J = 14.1 Hz, 1H), 7.73 (td, J = 7.9, 1.8 Hz, 1H), 7.46 (d, J = 8.1 Hz, 1H), 7.24 (dd, J = 12.8, 5.1 Hz, 1H), 7.20 – 7.15 (m, 1H), 7.12 (d, J = 7.8 Hz, 1H), 7.03 (d, J = 7.5 Hz, 1H), 2.99 – 2.61 (m, 1H), 2.37 (s, 3H), 1.92 (s, 6H), 1.70 – 1.60 (m, 1H), 1.56 – 1.46 (m, 1H), 1.30 – 1.09 (m, 9H), 0.83 – 0.67 (m, 3H). ^{13}C NMR (126 MHz, $CDCl_3$) δ 169.3, 169.3, 164.3, 147.4, 144.4, 139.3, 138.4, 137.1, 134.3, 134.0, 128.5, 128.2, 127.4, 127.3, 126.5, 123.1, 121.9, 121.9, 119.5, 119.4, 57.0, 38.3, 36.0, 33.5, 32.0, 32.0, 31.8, 29.8, 29.1, 27.4, 27.4, 27.3, 22.9, 22.6, 22.5, 19.3, 19.2, 14.0, 14.0; HRMS (ESI) m/z: calculated for $C_{23}H_{33}N_2O$ $[M + H]^+$: 353.2587, found: 353.2578.

2-((1R,2R,4S)-bicyclo[2.2.1]heptan-2-yl)-6-methyl-*N*-(2-(pyridin-2-yl)propan-2-yl)benzamide (3af)

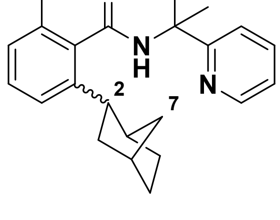

Yellow oil (32.8 mg, 47%). R_f 0.22 (PE/EtOAc = 3/1). ^{1}H NMR (400 MHz, $CDCl_3$) δ 8.46 – 8.39 (m, 1H), 8.07 (s, 1H), 7.74 (td, *J* = 7.8, 1.8 Hz, 1H), 7.47 (d, *J* = 8.1 Hz, 1H), 7.25 – 7.13 (m, 3H), 7.03 (dd, *J* = 12.1, 7.5 Hz, 1H), 2.95 (s, 1H), 2.36 (s, 3H), 2.31 (s, 2H), 1.93 (s, 6H), 1.68 (dd, *J* = 27.1, 10.1 Hz, 3H), 1.48 (s, 2H), 1.27 – 1.15 (m, 3H). ^{13}C NMR (126 MHz, $CDCl_3$) δ 168.7, 164.1, 147.5, 140.4, 137.2, 136.6, 134.1, 128.9, 128.7, 122.6, 122.0, 119.5, 78.3, 68.8, 57.0, 35.3, 27.4, 27.3, 26.3, 19.2; HRMS (ESI) m/z: calculated for $C_{23}H_{29}N_2O$ $[M + H]^+$: 349.2274, found: 349.2284.

2-methyl-*N*-(2-(pyridin-2-yl)propan-2-yl)-6-(tetrahydrofuran-2-yl)benzamide (3ag)

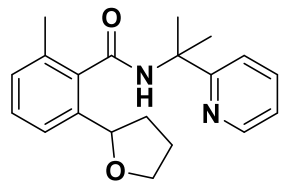

Yellow oil (42.2 mg, 65%). R_f 0.13(PE/EtOAc = 3/1). ^{1}H NMR (400 MHz, $CDCl_3$) δ 8.45 – 8.42 (m, 1H), 8.05 (s, 1H), 7.73 (td, *J* = 8.0, 1.8 Hz, 1H), 7.46 (d, *J* = 8.1 Hz, 1H), 7.38 – 7.32 (m, 1H), 7.26 (t, *J* = 7.7 Hz, 1H), 7.21 – 7.16 (m, 1H), 7.10 (d, *J* = 7.4 Hz, 1H), 5.07 (t, *J* = 7.2 Hz, 1H), 4.10 (dd, *J* = 14.2, 7.5 Hz, 1H), 3.92 – 3.85 (m, 1H), 2.37 (s, 3H), 2.36 – 2.29 (m, 1H), 2.04 – 1.91 (m, 2H), 1.90 (s, 6H), 1.84 – 1.76 (m, 1H). ^{13}C NMR (126 MHz, $CDCl_3$) δ 168.7, 164.1, 147.5, 140.4, 137.2, 136.6, 134.1, 128.9, 128.7, 122.6, 122.0, 119.5, 78.3, 68.8, 57.0, 35.3, 27.4, 27.3, 26.3, 19.2; HRMS (ESI) m/z: calculated for $C_{20}H_{25}N_2O_2$ $[M + H]^+$: 325.1911, found: 325.1905.

2-methyl-*N*-(2-(pyridin-2-yl)propan-2-yl)-6-(tetrahydrothiophen-2-yl)benzamide (3ah)

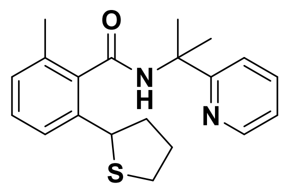

Yellow oil (43.0 mg, 63%). R_f 0.23 (PE/EtOAc = 3/1). ^1H NMR (400 MHz, $CDCl_3$) δ 8.44 (d, *J* = 4.4 Hz, 1H), 8.04 (s, 1H), 7.72 (td, *J* = 7.9, 1.6 Hz, 1H), 7.57 (d, *J* = 7.9 Hz, 1H), 7.46 (d, *J* = 8.1 Hz, 1H), 7.26 (t, *J* = 7.7 Hz, 1H), 7.18 (dd, *J* = 7.0, 5.2 Hz, 1H), 7.06 (d, *J* = 7.5 Hz, 1H), 4.73 (t, *J* = 6.8 Hz, 1H), 3.14 (dd, *J* = 16.1, 8.6 Hz, 1H), 2.97 (dd, *J* = 8.7, 4.9 Hz, 1H), 2.43 (dd, *J* = 10.8, 6.3 Hz, 1H), 2.36 (s, 3H), 2.27 – 2.19 (m, 1H), 1.91 (d, *J* = 9.5 Hz, 8H). ^{13}C NMR (151 MHz, $CDCl_3$) δ 168.8, 164.1, 147.5, 139.8, 138.3, 137.2, 134.1, 128.7, 128.6, 124.9, 122.0, 119.5, 57.2, 49.2, 41.1, 33.7, 31.3, 27.6, 27.3, 19.2; HRMS (ESI) m/z: calculated for $C_{20}H_{25}N_2OS$ $[M + H]^+$: 341.1682, found: 341.1675.

2-methyl-*N*-(2-(pyridin-2-yl)propan-2-yl)-6-(tetrahydro-2H-pyran-2-yl)benzamide (3ai)

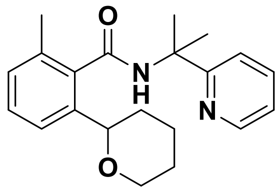

Yellow oil (47.7 mg, 70%). R_f 0.43 (PE/EtOAc = 3/1). ^1H NMR (400 MHz, $CDCl_3$) δ 8.44 (d, *J* = 4.3 Hz, 1H), 8.04 (s, 1H), 7.72 (td, *J* = 7.9, 1.6 Hz, 1H), 7.46 (d, *J* = 8.1 Hz, 1H), 7.37 (d, *J* = 7.8 Hz, 1H), 7.26 (dd, *J* = 8.8, 6.6 Hz, 1H), 7.17 (dd, *J* = 6.9, 5.1 Hz, 1H), 7.11 (d, *J* = 7.5 Hz, 1H), 4.57 – 4.46 (m, 1H), 4.10 – 3.99 (m, 1H), 3.54 (t, *J* = 11.6 Hz, 1H), 2.37 (s, 3H), 1.90 (t, *J* = 5.8 Hz, 7H), 1.71 – 1.46 (m, 5H). ^{13}C NMR (151 MHz, $CDCl_3$) δ 168.6, 164.2, 147.6, 139.5, 137.1, 136.9, 134.2, 129.3, 128.8, 123.3, 121.9, 119.5, 77.7, 69.2, 57.1, 33.7, 27.5, 27.4, 25.9, 24.1, 19.3; HRMS (ESI) m/z: calculated for $C_{21}H_{27}N_2O_2$ $[M + H]^+$: 339.2067, found: 339.2063.

2-(1,4-dioxan-2-yl)-6-methyl-*N*-(2-(pyridin-2-yl)propan-2-yl)benzamide (3aj)

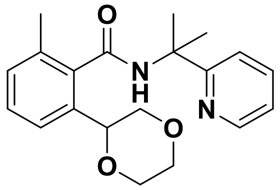

Yellow oil (53.2 mg, 78%). R_f 0.42 (PE/EtOAc = 3/1). ^{1}H NMR (400 MHz, $CDCl_3$) δ 8.42 (d, *J* = 4.2 Hz, 1H), 8.14 (s, 1H), 7.72 (td, *J* = 8.0, 1.7 Hz, 1H), 7.45 (d, *J* = 8.1 Hz, 1H), 7.39 (d, *J* = 7.8 Hz, 1H), 7.28 (t, *J* = 8.1 Hz, 1H), 7.20 – 7.11 (m, 2H), 4.81 (dd, *J* = 10.0, 2.4 Hz, 1H), 4.01 (dd, *J* = 11.5, 2.5 Hz, 1H), 3.91 – 3.77 (m, 2H), 3.70 (dd, *J* = 9.4, 3.1 Hz, 2H), 3.46 (dd, *J* = 11.4, 10.3 Hz, 1H), 2.37 (s, 3H), 1.92 (d, *J* = 5.9 Hz, 6H). ^{13}C NMR (126 MHz, $CDCl_3$) δ 168.2, 164.0, 147.5, 137.4, 137.2, 134.9, 134.2, 129.9, 128.7, 124.0, 122.0, 119.5, 75.8, 72.5, 67.2, 66.3, 57.1, 27.5, 27.3, 19.2; HRMS (ESI) m/z: calculated for $C_{20}H_{25}N_2O_3$ $[M + H]^+$: 341.1860, found: 341.1852.

2-(1-butoxybutyl)-6-methyl-*N*-(2-(pyridin-2-yl)propan-2-yl)benzamide (3ak)

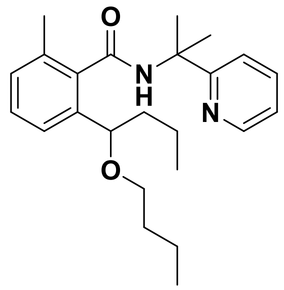

Yellow oil (62.0 mg, 81%). R_f 0.53 (PE/EtOAc = 3/1). ^{1}H NMR (400 MHz, $CDCl_3$) δ 8.43 – 8.33 (m, 1H), 8.19 (s, 1H), 7.73 (td, *J* = 8.0, 1.7 Hz, 1H), 7.45 (d, *J* = 8.1 Hz, 1H), 7.34 (d, *J* = 7.6 Hz, 1H), 7.27 (t, *J* = 7.6 Hz, 1H), 7.20 – 7.14 (m, 1H), 7.10 (d, *J* = 7.4 Hz, 1H), 4.48 (dd, *J* = 8.6, 4.4 Hz, 1H), 3.38 – 3.28 (m, 1H), 3.27 – 3.19 (m, 1H), 2.37 (s, 3H), 1.92 (d, *J* = 1.9 Hz, 6H), 1.78 – 1.59 (m, 2H), 1.54 – 1.41 (m, 3H), 1.32 – 1.23 (m, 3H), 0.86 – 0.77 (m, 6H). ^{13}C NMR (126 MHz, $CDCl_3$) δ 168.5, 164.1, 147.3, 140.4, 137.9, 137.2, 133.8, 128.8, 128.6, 123.5, 122.0, 119.5, 78.3, 68.7, 57.0, 41.0, 32.0, 27.4, 27.3, 19.5, 19.4, 19.1, 14.0, 13.9; HRMS (ESI) m/z: calculated for $C_{24}H_{35}N_2O_2$ $[M + H]^+$: 383.2693, found: 383.2688.

2-(1-methoxybutyl)-6-methyl-*N*-(2-(pyridin-2-yl)propan-2-yl)benzamide (3al)

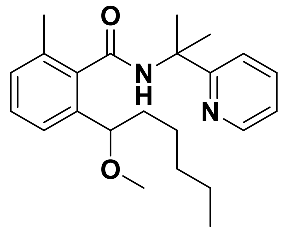

Yellow oil (25.1 mg, 34%). R_f 0.53 (PE/EtOAc = 3/1). ^1H NMR (400 MHz, $CDCl_3$) δ 8.40 (d, *J* = 4.5 Hz, 1H), 8.20 (s, 1H), 7.75 (t, *J* = 7.3 Hz, 1H), 7.46 (d, *J* = 8.1 Hz, 1H), 7.33 – 7.27 (m, 2H), 7.23 – 7.16 (m, 1H), 7.13 (dd, *J* = 5.9, 2.7 Hz, 1H), 4.37 (dd, *J* = 7.9, 5.0 Hz, 1H), 3.20 (s, 3H), 2.38 (s, 3H), 1.93 (s, 6H), 1.75 – 1.64 (m, 2H), 1.51 – 1.41 (m, 1H), 1.19 (s, 5H), 0.76 (s, 3H). ^{13}C NMR (126 MHz, $CDCl_3$) δ 168.5, 164.1, 147.3, 139.6, 138.2, 137.2, 133.9, 129.0, 128.7, 123.3, 122.0, 119.5, 80.5, 57.0, 56.8, 38.6, 31.8, 27.4, 27.3, 25.9, 22.5, 19.1, 14.0; HRMS (ESI) m/z: calculated for $C_{23}H_{33}N_2O_2$ $[M + H]^+$: 369.2537, found: 369.2526.

2-methyl-6-(phenoxymethyl)-*N*-(2-(pyridin-2-yl)propan-2-yl)benzamide (3am)

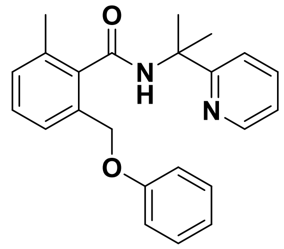

Yellow oil (29.6 mg, 41%). R_f 0.63 (PE/EtOAc = 3/1). ^1H NMR (400 MHz, $CDCl_3$) δ 8.45 (d, *J* = 4.3 Hz, 1H), 8.08 (s, 1H), 7.68 – 7.59 (m, 1H), 7.42 – 7.34 (m, 2H), 7.30 (t, *J* = 7.6 Hz, 1H), 7.21 (dd, *J* = 10.1, 5.7 Hz, 3H), 7.16 (dd, *J* = 7.0, 5.2 Hz, 1H), 6.91 (t, *J* = 8.8 Hz, 3H), 5.12 (s, 2H), 2.43 (s, 3H), 1.80 (s, 6H). ^{13}C NMR (126 MHz, $CDCl_3$) δ 168.1, 164.1, 158.6, 147.6, 138.0, 137.0, 135.0, 133.2, 130.2, 129.4, 129.3, 128.8, 126.4, 121.9, 120.8, 119.4, 114.7, 67.8, 57.2, 27.4, 19.3; HRMS (ESI) m/z: calculated for $C_{23}H_{25}N_2O_2$ $[M + H]^+$: 361.1911 , found: 361.1903.

2-methyl-6-(1-phenoxyethyl)-*N*-(2-(pyridin-2-yl)propan-2-yl)benzamide (3an)

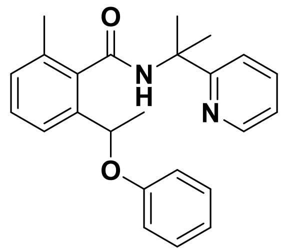

Yellow oil (18.7 mg, 25%). R_f 0.63 (PE/EtOAc = 3/1). ^{1}H NMR (400 MHz, $CDCl_3$) δ 8.46 (d, *J* = 4.8 Hz, 1H), 8.37 (s, 1H), 7.75 (td, *J* = 7.9, 1.7 Hz, 1H), 7.47 (d, *J* = 8.1 Hz, 1H), 7.40 (d, *J* = 7.8 Hz, 1H), 7.26 – 7.19 (m, 2H), 7.18 – 7.10 (m, 3H), 6.97 (d, *J* = 7.9 Hz, 2H), 6.82 (t, *J* = 7.3 Hz, 1H), 5.60 (q, *J* = 6.3 Hz, 1H), 2.42 (s, 3H), 1.96 (s, 6H), 1.68 (d, *J* = 6.3 Hz, 3H). ^{13}C NMR (151 MHz, $CDCl_3$) δ 168.4, 164.0, 157.7, 147.5, 140.1, 137.4, 136.6, 134.0, 129.4, 129.3, 129.1, 122.7, 122.1, 120.4, 119.5, 115.7, 72.6, 57.2, 27.5, 27.3, 24.8, 19.2; HRMS (ESI) m/z: calculated for $C_{24}H_{27}N_2O_2$ $[M + H]^+$: 375.2067, found: 375.2070.

1-(3-methyl-2-((2-(pyridin-2-yl)propan-2-yl)carbamoyl)phenyl)ethyl acetate (3ao)

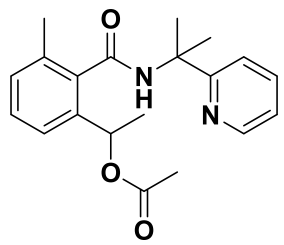

Yellow oil (20.4 mg, 30%). R_f 0.43 (PE/EtOAc = 3/1). ^{1}H NMR (400 MHz, $CDCl_3$) δ 8.45 (dd, *J* = 5.3, 1.8 Hz, 1H), 8.03 (s, 1H), 7.69 (t, *J* = 7.1 Hz, 1H), 7.48 (d, *J* = 8.0 Hz, 1H), 7.28 – 7.22 (m, 2H), 7.18 – 7.06 (m, 2H), 6.08 (q, *J* = 6.5 Hz, 1H), 2.35 (s, 3H), 1.98 (s, 3H), 1.87 (d, *J* = 5.8 Hz, 6H), 1.50 (d, *J* = 6.5 Hz, 3H). ^{13}C NMR (126 MHz, $CDCl_3$) δ 170.4, 168.1, 164.3, 147.6, 138.8, 137.0, 136.7, 134.7, 129.7, 128.9, 122.8, 121.8, 119.7, 70.5, 57.2, 27.6, 27.4, 22.7, 21.3, 19.3; HRMS (ESI) m/z: calculated for $C_{20}H_{25}N_2O_3$ $[M + H]^+$: 341.1860, found: 341.1860.

2-methyl-6-((*N*-methylacetamido)methyl)-*N*-(2-(pyridin-2-yl)propan-2-yl)benzamide (3ap)

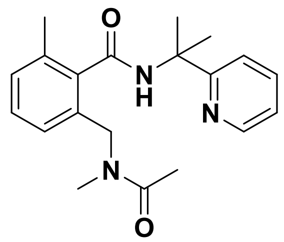

Yellow oil (21.0 mg, 34%). R_f 0.37 (PE/EtOAc = 3/1). ^{1}H NMR (400 MHz, $CDCl_3$) δ 8.43 (t, *J* = 5.2 Hz, 1H), 8.20 (d, *J* = 23.4 Hz, 1H), 7.83 – 7.69 (m, 1H), 7.51 – 7.42 (m, 1H), 7.31 – 7.15 (m, 3H), 7.13 – 7.06 (m, 1H), 4.65 (d, *J* = 26.3 Hz, 2H), 2.93 (s, 3H), 2.40 (d, *J* = 14.0 Hz, 3H), 2.12 (d, *J* = 13.6 Hz, 3H), 1.91 (d, *J* = 6.8 Hz, 6H). ^{13}C NMR (151 MHz, MeOD) δ 172.7, 172.5, 169.8, 169.5, 164.4, 164.4, 147.7, 147.6, 137.2, 137.1, 136.9, 136.5, 135.0, 134.5, 133.6, 133.2, 129.1, 129.0, 128.7, 124.1, 122.4, 121.8, 120.1, 120.0, 57.2, 51.2, 35.0, 33.0, 26.6, 20.3, 19.9, 17.9, 17.9; HRMS (ESI) m/z: calculated for $C_{24}H_{27}N_2O$ [M + H]$^+$: 340.2020, found: 340.2017.

2-(3-butyloxiran-2-yl)-6-methyl-*N*-(2-(pyridin-2-yl)propan-2-yl)benzamide (3aq)

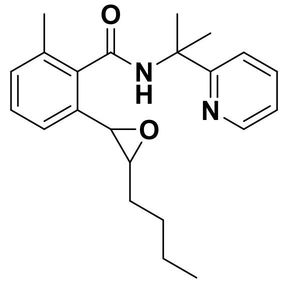

Yellow oil (21.0 mg, 34%). R_f 0.37 (PE/EtOAc = 3/1). ^{1}H NMR (400 MHz, $CDCl_3$) δ 8.41 (d, *J* = 4.5 Hz, 1H), 8.12 (s, 1H), 7.74 (t, *J* = 7.0 Hz, 1H), 7.45 (d, *J* = 8.1 Hz, 1H), 7.23 (d, *J* = 7.7 Hz, 1H), 7.18 (dd, *J* = 7.0, 5.1 Hz, 1H), 7.12 (d, *J* = 7.8 Hz, 1H), 7.04 (d, *J* = 7.4 Hz, 1H), 2.80 (s, 1H), 2.63 (s, 1H), 2.36 (s, 3H), 1.91 (s, 6H), 1.80 – 1.65 (m, 2H), 1.25 (dd, *J* = 6.8, 2.3 Hz, 4H), 0.96 – 0.78 (m, 3H). ^{13}C NMR (126 MHz, $CDCl_3$) δ 169.2, 169.2, 164.2, 147.4, 143.4, 143.3, 138.5, 138.5, 137.1, 134.2, 134.1, 128.6, 127.6, 122.9, 122.9, 121.9, 119.4, 57.0, 52.4, 47.0, 36.0, 34.4, 34.0, 27.4, 23.2, 23.0, 19.3; HRMS (ESI) m/z: calculated for $C_{22}H_{29}N_2O_2$ [M + H]$^+$: 353.2224, found: 353.2217.

2-methyl-6-(4-methylbenzyl)-*N*-(2-(pyridin-2-yl)propan-2-yl)benzamide (3ar)

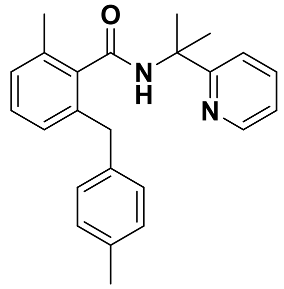

Yellow oil (40.2 mg, 56%). R_f 0.46 (PE/EtOAc = 3/1). ^{1}H NMR (400 MHz, $CDCl_3$) δ 8.40 (d, *J* = 4.3 Hz, 1H), 7.97 (s, 1H), 7.71 (td, *J* = 7.9, 1.7 Hz, 1H), 7.41 (d, *J* = 8.1 Hz, 1H), 7.21 – 7.14 (m, 2H), 7.07 (dt, *J* = 14.2, 7.9 Hz, 5H), 6.96 (d, *J* = 7.6 Hz, 1H), 4.04 (s, 2H), 2.41 (s, 3H), 2.27 (s, 3H), 1.87 (s, 6H). ^{13}C NMR (126 MHz, $CDCl_3$) δ 169.1, 164.2, 147.5, 138.6, 137.9, 137.4, 137.1, 135.3, 134.5, 129.1, 129.0, 128.9, 128.4, 128.0, 127.4, 121.8, 119.4, 57.1, 38.2, 27.3, 21.0, 19.3; HRMS (ESI) m/z: calculated for $C_{24}H_{27}N_2O$ $[M + H]^+$: 359.2118, found: 359.2116.

2-(4-chlorobenzyl)-6-methyl-*N*-(2-(pyridin-2-yl)propan-2-yl)benzamide (3as)

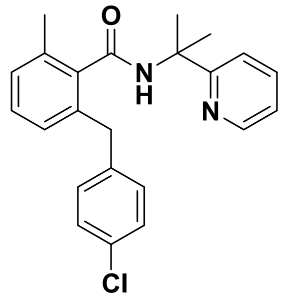

Yellow oil (47.8 mg, 63%). R_f 0.41 (PE/EtOAc = 3/1). ^{1}H NMR (500 MHz, $CDCl_3$) δ 8.37 (ddd, *J* = 4.9, 1.6, 0.9 Hz, 1H), 8.00 (s, 1H), 7.70 (td, *J* = 7.8, 1.8 Hz, 1H), 7.40 (d, *J* = 8.1 Hz, 1H), 7.22 – 7.07 (m, 7H), 6.95 (d, *J* = 7.7 Hz, 1H), 4.04 (s, 2H), 2.41 (s, 3H), 1.86 (s, 6H). ^{13}C NMR (126 MHz, $CDCl_3$) δ 168.9, 164.0, 147.4, 139.5, 138.7, 137.1, 136.5, 134.7, 131.7, 130.3, 128.5, 128.4, 128.3, 127.5, 121.9, 119.3, 57.1, 38.1, 27.3, 19.4; HRMS (ESI) m/z: calculated for $C_{23}H_{24}ClN_2O$ $[M + H]^+$: 379.1572, found: 379.1578.

2-(4-bromobenzyl)-6-methyl-*N*-(2-(pyridin-2-yl)propan-2-yl)benzamide (3at)

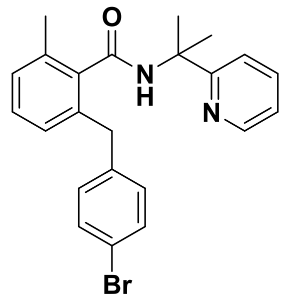

Yellow oil (44.0 mg, 52%). R_f 0.27 (PE/EtOAc = 3/1). ^1H NMR (400 MHz, $CDCl_3$) δ 8.37 (ddd, J = 4.9, 1.7, 0.9 Hz, 1H), 8.00 (s, 1H), 7.71 (td, J = 8.0, 1.8 Hz, 1H), 7.40 (d, J = 8.1 Hz, 1H), 7.31 (d, J = 8.4 Hz, 2H), 7.21 – 7.15 (m, 2H), 7.08 (t, J = 8.9 Hz, 3H), 6.94 (d, J = 7.6 Hz, 1H), 4.01 (s, 2H), 2.40 (s, 3H), 1.86 (s, 6H). ^{13}C NMR (126 MHz, $CDCl_3$) δ 168.8, 163.9, 147.4, 140.1, 138.7, 137.2, 136.4, 134.8, 131.4, 130.7, 128.5, 128.3, 127.5, 122.0, 119.8, 119.4, 57.0, 38.1, 27.3, 19.4; HRMS (ESI) m/z: calculated for $C_{23}H_{24}BrN_2O$ $[M + H]^+$: 423.1067, found: 423.1056.

4-(3-methyl-2-((2-(pyridin-2-yl)propan-2-yl)carbamoyl)benzyl)benzoate (3au)

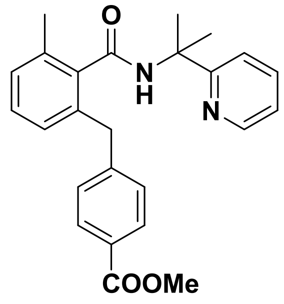

Yellow oil (52.3 mg, 65%). R_f 0.28 (PE/EtOAc = 3/1). ^1H NMR (400 MHz, $CDCl_3$) δ 8.36 – 8.34 (m, 1H), 7.99 (s, 1H), 7.87 (d, J = 8.4 Hz, 2H), 7.73 – 7.64 (m, 1H), 7.38 (d, J = 8.1 Hz, 1H), 7.26 (s, 1H), 7.24 (s, 1H), 7.19 (t, J = 7.6 Hz, 1H), 7.16 – 7.12 (m, 1H), 7.09 (d, J = 7.3 Hz, 1H), 6.95 (d, J = 7.3 Hz, 1H), 4.12 (s, 2H), 3.86 (s, 3H), 2.40 (s, 3H), 1.84 (s, 6H). ^{13}C NMR (126 MHz, $CDCl_3$) δ 168.8, 167.0, 163.9, 147.4, 146.5, 138.7, 137.1, 136.1, 134.8, 129.7, 129.0, 128.6, 128.4, 127.9, 127.6, 121.9, 119.3, 57.0, 51.9, 38.7, 27.3, 19.4; HRMS (ESI) m/z: calculated for $C_{25}H_{27}N_2O_3$ $[M + H]^+$: 403.2016, found: 403.2020.

2-methyl-6-(3-methylbenzyl)-*N*-(2-(pyridin-2-yl)propan-2-yl)benzamide (3av)

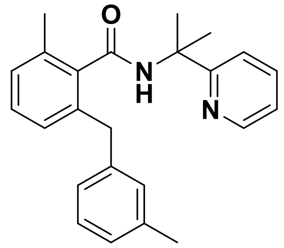

Yellow oil (55.2 mg, 77%). R_f 0.29 (PE/EtOAc = 3/1). ^1H NMR (400 MHz, $CDCl_3$) δ 8.40 (ddd, *J* = 4.9, 1.7, 0.9 Hz, 1H), 7.97 (s, 1H), 7.71 (td, *J* = 8.0, 1.8 Hz, 1H), 7.41 (d, *J* = 8.1 Hz, 1H), 7.22 – 7.15 (m, 2H), 7.15 – 7.06 (m, 2H), 7.01 (d, *J* = 5.8 Hz, 2H), 6.96 (dd, *J* = 11.2, 7.7 Hz, 2H), 4.05 (s, 2H), 2.41 (s, 3H), 2.25 (s, 3H), 1.86 (s, 6H). ^{13}C NMR (126 MHz, $CDCl_3$) δ 169.1, 164.2, 147.4, 140.9, 138.6, 137.9, 137.2, 137.1, 134.6, 129.8, 128.4, 128.3, 128.0, 127.5, 126.7, 126.1, 121.9, 119.4, 57.1, 38.6, 27.3, 21.4, 19.4; HRMS (ESI) m/z: calculated for $C_{24}H_{27}N_2O$ $[M + H]^+$: 359.2118, found: 359.2110.

2-(3-bromobenzyl)-6-methyl-*N*-(2-(pyridin-2-yl)propan-2-yl)benzamide (3aw)

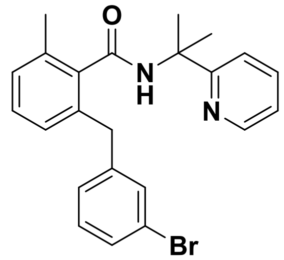

Yellow oil (52.4 mg, 62%). R_f 0.43 (PE/EtOAc = 3/1). ^1H NMR (400 MHz, $CDCl_3$) δ 8.42 – 8.39 (m, 1H), 8.01 (s, 1H), 7.75 – 7.65 (m, 1H), 7.39 (d, *J* = 8.1 Hz, 1H), 7.34 (s, 1H), 7.25 – 7.03 (m, 6H), 6.96 (d, *J* = 7.6 Hz, 1H), 4.04 (s, 2H), 2.40 (s, 3H), 1.85 (s, 6H). ^{13}C NMR (126 MHz, $CDCl_3$) δ 168.8, 164.0, 147.5, 143.4, 138.7, 137.2, 136.2, 134.8, 131.9, 129.9, 129.1, 128.6, 128.4, 127.7, 127.6, 122.5, 121.9, 119.3, 57.0, 38.3, 27.3, 19.4; HRMS (ESI) m/z: calculated for $C_{23}H_{24}BrN_2O$ $[M + H]^+$: 423.1067, found: 423.1066.

2-benzyl-6-methyl-*N*-(2-(pyridin-2-yl)propan-2-yl)benzamide (3ax)

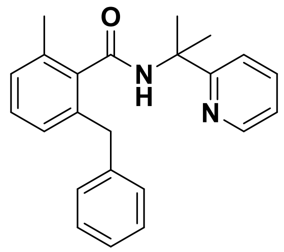

Yellow oil (85.4 mg, 62%). R_f 0.33 (PE/EtOAc = 3/1).^{1}H NMR (400 MHz, $CDCl_3$) δ 8.47 – 8.40 (m, 1H), 8.02 (s, 1H), 7.77 – 7.70 (m, 1H), 7.44 (d, *J* = 8.1 Hz, 1H), 7.31 – 7.25 (m, 4H), 7.24 – 7.15 (m, 3H), 7.12 (d, *J* = 7.5 Hz, 1H), 7.01 (d, *J* = 7.6 Hz, 1H), 4.14 (s, 2H), 2.46 (s, 3H), 1.90 (s, 6H). ^{13}C NMR (126 MHz, CDCl3) δ 169.0, 164.1, 147.5, 141.0, 138.7, 137.1, 137.1, 134.6, 129.0, 128.5, 128.4, 128.1, 127.5, 126.0, 121.9, 119.4, 57.1, 38.7, 27.3, 19.4; HRMS (ESI) m/z: calculated for $C_{23}H_{25}N_2O$ [M + H]$^+$: 345.1961, found: 345.1956.

2-methyl-6-(naphthalen-2-ylmethyl)-*N*-(2-(pyridin-2-yl)propan-2-yl)benzamide (3ay)

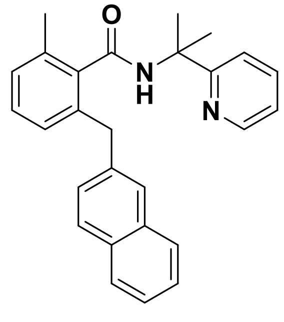

Yellow oil (40.2 mg, 51%). R_f 0.53 (PE/EtOAc = 3/1). ^{1}H NMR (400 MHz, $CDCl_3$) δ 8.14 – 8.10 (m, 1H), 8.01 (s, 1H), 7.77 – 7.66 (m, 3H), 7.66 – 7.60 (m, 1H), 7.59 (s, 1H), 7.43 – 7.31 (m, 4H), 7.21 (t, *J* = 7.6 Hz, 1H), 7.11 (d, *J* = 7.4 Hz, 1H), 7.07 – 7.01 (m, 2H), 4.25 (s, 2H), 2.44 (s, 3H), 1.85 (s, 6H). ^{13}C NMR (126 MHz, $CDCl_3$) δ 169.0, 163.9, 147.3, 138.8, 138.7, 137.0, 136.8, 134.8, 133.6, 132.1, 128.5, 128.2, 127.9, 127.7, 127.6, 127.6, 127.1, 125.8, 125.2, 121.8, 119.2, 57.0, 38.9, 27.3, 19.4; HRMS (ESI) m/z: calculated for $C_{27}H_{27}N_2O$ [M + H]$^+$: 395.2118, found: 395.2110.

2-(4-methylbenzyl)-*N*-(2-(pyridin-2-yl)propan-2-yl)benzamide (3fr)

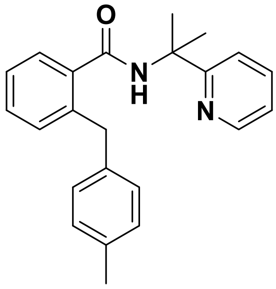

Yellow oil (21.0 mg, 21%). R_f 0.56 (PE/EtOAc = 3/1). ^{1}H NMR (500 MHz, $CDCl_3$) δ 8.45 (d, *J* = 4.3 Hz, 1H), 7.89 (s, 1H), 7.72 (t, *J* = 7.2 Hz, 1H), 7.52 (d, *J* = 7.3 Hz, 1H), 7.42 (d, *J* = 8.1 Hz, 1H), 7.32 (td, *J* = 7.5, 1.4 Hz, 1H), 7.27 – 7.23 (m, 1H), 7.21 – 7.15 (m, 2H), 7.08 (d, *J* = 8.0 Hz, 2H), 7.03 (d, *J* = 8.0 Hz, 2H), 4.19 (s, 2H), 2.27 (s, 3H), 1.80 (s, 6H). ^{13}C NMR (126 MHz, $CDCl_3$) δ 169.3, 164.4, 147.7, 139.0, 138.0, 137.9, 137.0, 135.3, 130.8, 129.6, 129.0, 129.0, 127.3, 126.2, 121.8, 119.4, 57.1, 38.2, 27.4, 21.0; HRMS (ESI) m/z: calculated for $C_{23}H_{25}N_2O$ $[M + H]^+$: 345.1961, found: 345.1959.

2,6-bis(4-methylbenzyl)-*N*-(2-(pyridin-2-yl)propan-2-yl)benzamide (3fr')

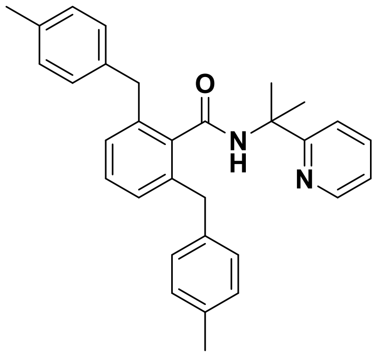

Yellow oil (21.0 mg, 51%). R_f 0.58 (PE/EtOAc = 3/1). ^{1}H NMR (500 MHz, $CDCl_3$) δ 8.36 (d, *J* = 4.5 Hz, 1H), 7.87 (s, 1H), 7.68 (t, *J* = 7.2 Hz, 1H), 7.36 (d, *J* = 8.0 Hz, 1H), 7.16 (t, *J* = 7.7 Hz, 2H), 7.10 (d, *J* = 8.0 Hz, 4H), 7.03 (d, *J* = 7.9 Hz, 4H), 6.95 (d, *J* = 7.7 Hz, 2H), 4.05 (s, 4H), 2.26 (s, 6H), 1.80 (s, 6H). ^{13}C NMR (126 MHz, $CDCl_3$) δ 168.9, 164.0, 147.4, 138.3, 137.8, 137.7, 136.9, 135.4, 129.1, 129.0, 128.6, 127.8, 121.7, 119.3, 57.1, 38.2, 27.2, 21.0; HRMS (ESI) m/z: calculated for $C_{31}H_{33}N_2O$ $[M + H]^+$: 449.2587, found: 449.2591.

4-methyl-2-(4-methylbenzyl)-*N*-(2-(pyridin-2-yl)propan-2-yl)benzamide (3kr)

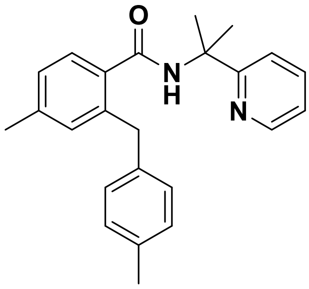

Yellow oil (38.7 mg, 27%). R_f 0.45 (PE/EtOAc = 3/1). ^{1}H NMR (500 MHz, $CDCl_3$) δ 8.46 – 8.42 (m, 1H), 7.88 (s, 1H), 7.69 (td, *J* = 7.8, 1.8 Hz, 1H), 7.41 (dd, *J* = 10.2, 7.9 Hz, 2H), 7.18 – 7.14 (m, 1H), 7.10 (d, *J* = 8.0 Hz, 2H), 7.05 (t, *J* = 8.6 Hz, 3H), 7.00 (s, 1H), 4.18 (s, 2H), 2.31 (s, 3H), 2.28 (s, 3H), 1.79 (s, 6H). ^{13}C NMR (126 MHz, $CDCl_3$) δ 169.0, 164.0, 147.2, 139.2, 138.6, 137.8, 136.7, 134.8, 134.7, 131.1, 128.6, 128.5, 127.0, 126.4, 121.4, 119.0, 56.6, 37.8, 27.0, 20.9, 20.6; HRMS (ESI) m/z: calculated for $C_{24}H_{27}N_2O$ $[M + H]^+$: 359.2118, found: 359.2113.

4-methyl-2,6-bis(4-methylbenzyl)-*N*-(2-(pyridin-2-yl)propan-2-yl)benzamide (3kr')

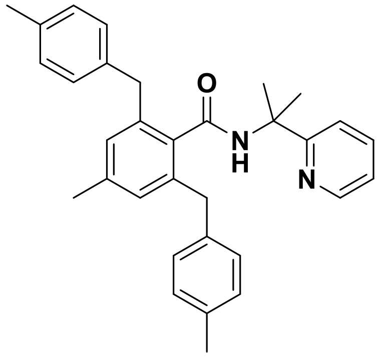

Yellow oil (57.4 mg, 31%). R_f 0.55 (PE/EtOAc = 3/1). ^{1}H NMR (500 MHz, $CDCl_3$) δ 8.36 (d, *J* = 4.8 Hz, 1H), 7.84 (s, 1H), 7.66 (t, *J* = 7.7 Hz, 1H), 7.34 (d, *J* = 8.1 Hz, 1H), 7.11 (d, *J* = 7.9 Hz, 5H), 7.04 (d, *J* = 7.8 Hz, 4H), 6.78 (s, 2H), 4.02 (s, 4H), 2.27 (s, 6H), 2.20 (s, 3H), 1.79 (s, 6H). ^{13}C NMR (126 MHz, $CDCl_3$) δ 169.1, 164.1, 147.4, 138.2, 138.0, 137.6, 135.3, 129.3, 129.1, 129.0, 128.5, 121.7, 121.5, 119.3, 57.1, 38.2, 27.2, 21.3, 21.0; HRMS (ESI) m/z: calculated for $C_{32}H_{35}N_2O$ $[M + H]^+$: 463.2744, found: 463.2749.

5-methoxy-2-(4-methylbenzyl)-*N*-(2-(pyridin-2-yl)propan-2-yl)benzamide (3or)

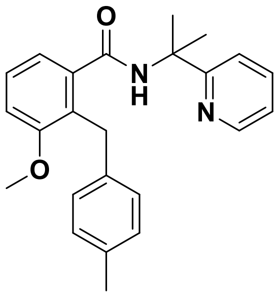

Yellow oil (50.9 mg, 34%). R_f 0.55 (PE/EtOAc = 3/1). ^{1}H NMR (500 MHz, $CDCl_3$) δ 8.44 – 8.39 (m, 1H), 7.90 (s, 1H), 7.69 (td, *J* = 7.8, 1.7 Hz, 1H), 7.38 (d, *J* = 8.1 Hz, 1H), 7.16 (dd, *J* = 7.4, 5.0 Hz, 1H), 7.10 – 7.00 (m, 6H), 6.87 (dd, *J* = 8.5, 2.8 Hz, 1H), 4.12 (s, 2H), 3.81 (s, 3H), 2.26 (s, 3H), 1.78 (s, 6H). ^{13}C NMR (126 MHz, $CDCl_3$) δ 169.0, 164.3, 157.8, 147.6, 138.8, 138.4, 137.0, 135.3, 132.0, 130.7, 129.0, 128.8, 121.8, 119.4, 115.3, 112.8, 60.4, 57.1, 55.5, 37.5, 27.4, 21.0, 21.0, 14.2; HRMS (ESI) m/z: calculated for $C_{24}H_{27}N_2O_2$ $[M + H]^+$: 375.2067, found: 375.2065.

3-methoxy-2,6-bis(4-methylbenzyl)-*N*-(2-(pyridin-2-yl)propan-2-yl)benzamide (3or')

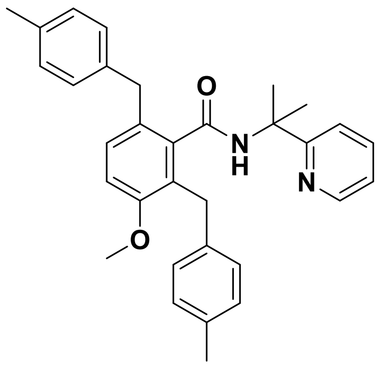

Yellow oil (53.6 mg, 28%). R_f 0.40 (PE/EtOAc = 3/1). ^{1}H NMR (500 MHz, $CDCl_3$) δ 8.31 (d, *J* = 4.4 Hz, 1H), 7.71 – 7.59 (m, 2H), 7.29 (d, *J* = 8.1 Hz, 1H), 7.09 (dd, *J* = 10.9, 8.0 Hz, 5H), 7.04 (d, *J* = 7.9 Hz, 2H), 6.95 (d, *J* = 8.3 Hz, 3H), 6.80 (d, *J* = 8.5 Hz, 1H), 4.05 (s, 2H), 3.98 (s, 2H), 3.72 (s, 3H), 2.27 (s, 3H), 2.22 (s, 3H), 1.73 (s, 6H). ^{13}C NMR (126 MHz, $CDCl_3$) δ 168.1, 163.5, 155.9, 146.9, 139.3, 137.8, 137.5, 136.5, 134.8, 134.2, 129.3, 128.7, 128.6, 128.5, 128.3, 127.9, 125.1, 121.2, 118.9,

110.7; HRMS (ESI) m/z: calculated for $C_{32}H_{35}N_2O_2$ $[M + H]^+$: 479.2693, found: 479.2687.

2-(4-methylbenzyl)-*N*-(2-(pyridin-2-yl)propan-2-yl)-1-naphthamide (3mr)

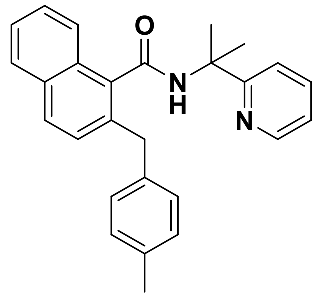

Yellow oil (39.5 mg, 50%). R_f 0.23 (PE/EtOAc = 3/1). ^1H NMR (500 MHz, $CDCl_3$) δ 8.36 (d, *J* = 4.4 Hz, 1H), 8.17 (s, 1H), 8.01 (d, *J* = 8.3 Hz, 1H), 7.80 (d, *J* = 7.8 Hz, 1H), 7.78 – 7.68 (m, 2H), 7.52 – 7.48 (m, 1H), 7.45 (dd, *J* = 7.2, 6.5 Hz, 2H), 7.27 (d, *J* = 9.3 Hz, 1H), 7.15 (d, *J* = 8.0 Hz, 3H), 7.04 (d, *J* = 7.9 Hz, 2H), 4.20 (s, 2H), 2.27 (s, 3H), 1.98 (s, 6H). ^{13}C NMR (126 MHz, $CDCl_3$) δ 168.7, 164.0, 147.4, 137.7, 137.2, 135.5, 135.0, 134.7, 132.0, 130.4, 129.1, 129.0, 128.8, 127.9, 127.9, 126.8, 125.6, 125.2, 121.9, 119.5, 57.4, 38.6, 27.5, 21.0; HRMS (ESI) m/z: calculated for $C_{27}H_{27}N_2O$ $[M + H]^+$: 395.2118, found: 395.2111.

2-methyl-6-(4-methylbenzyl)-*N*-(1-(pyridin-2-yl)ethyl)benzamide (3qr)

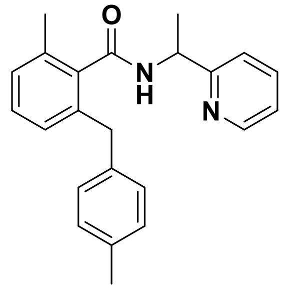

Yellow oil (54.5 mg, 79%). R_f 0.45 (PE/EtOAc = 3/1). ^1H NMR (400 MHz, $CDCl_3$) δ 8.43 (d, *J* = 4.9 Hz, 1H), 7.66 (td, *J* = 7.7, 1.8 Hz, 1H), 7.25 (d, *J* = 7.6 Hz, 1H), 7.20 – 7.14 (m, 2H), 7.07 – 6.93 (m, 7H), 5.31 (p, *J* = 6.8 Hz, 1H), 3.94 (s, 2H), 2.31 (s, 3H), 2.23 (s, 3H), 1.50 (d, *J* = 6.8 Hz, 3H). ^{13}C NMR (151 MHz, $CDCl_3$) δ 169.1, 160.4, 148.9, 137.6, 137.5, 136.8, 135.3, 134.5, 129.0, 128.7, 128.7, 128.0, 127.4,

122.3, 121.5, 49.8, 38.3, 22.6, 21.0, 19.2; HRMS (ESI) m/z: calculated for $C_{23}H_{25}N_2O$ $[M + H]^+$: 345.1961, found: 345.1966.

2-methyl-6-(4-methylbenzyl)-*N*-(1-(pyridin-2-yl)propyl)benzamide (3rr)

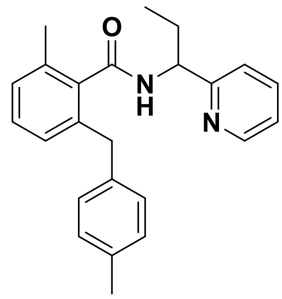

Yellow oil (59.5 mg, 83%). R_f 0.56 (PE/EtOAc = 3/1). ^1H NMR (400 MHz, $CDCl_3$) δ 8.49 – 8.42 (m, 1H), 7.65 (td, *J* = 7.7, 1.8 Hz, 1H), 7.23 (d, *J* = 7.8 Hz, 1H), 7.19 – 7.14 (m, 2H), 7.07 – 7.02 (m, 2H), 6.99 – 6.86 (m, 5H), 5.17 (dd, *J* = 14.5, 6.6 Hz, 1H), 3.93 (s, 2H), 2.31 (s, 3H), 2.23 (s, 3H), 1.90 (p, *J* = 7.2 Hz, 2H), 0.83 (t, *J* = 7.2 Hz, 3H). ^{13}C NMR (126 MHz, $CDCl_3$) δ 169.1, 162.4, 149.1, 138.7, 138.1, 138.1, 137.1, 135.2, 134.2, 129.3, 129.2, 128.5, 127.8, 127.2, 122.6, 121.5, 56.6, 38.0, 28.3, 21.1, 19.6, 11.5; HRMS (ESI) m/z: calculated for $C_{24}H_{27}N_2O$ $[M + H]^+$: 359.2118, found: 359.2110.

(IV) Optimization of the reaction conditions

Supplementary Table 1: Optimization of the reaction conditions.[a]

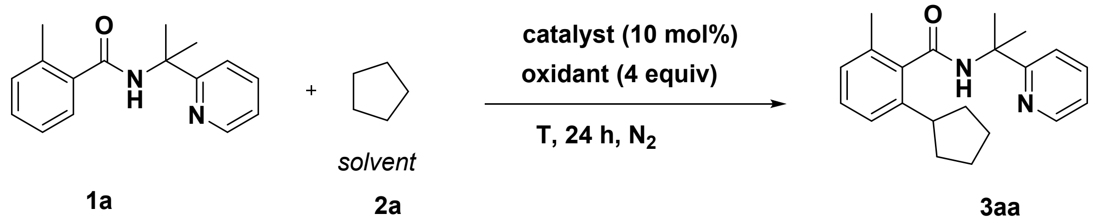

Entry	Catalyst	Oxidant	Temp (°C)	Yields (%)[b]
1	$Co(acac)_2$	DTBP	130	55
2	$Co(OAc)_2\,4H_2O$	DTBP	130	30
3	$CoBr_2$	DTBP	130	N. D.
4	CoC_2O_4	DTBP	130	34
5	Co_2CO_3	THBP	130	25
6	$FeCl_3$	DTBP	130	N. D.
7	$Ni(OAc)_2$	DTBP	130	N. D.
8	$Ni(acac)_2$	DTBP	130	N. D.
9	$Cu(OAc)_2$	DTBP	130	N. D.
10	$Co(acac)_2$	THBP	130	N. D.
11	$Co(acac)_2$	DCP	130	trace
12	$Co(acac)_2$	BPO	130	N. D.
13	$Co(acac)_2$	$K_2S_2O_8$	130	N. D.
14	$Co(acac)_2$	DDQ	130	N. D.
15	$Co(acac)_2$	DTBP	140	72
16	$Co(acac)_2$	DTBP	150	70
17[c]	$Co(acac)_2$	DTBP	140	57
18[d]	$Co(acac)_2$	DTBP	140	62
19[e]	$Co(acac)_2$	DTBP	140	25

[a] Reaction conditions: **1a** (0.2 mmol), **2a** (1 mL), $Co(acac)_2$ (0.02 mmol), DTBP (0.8 mmol) in $PhCF_3$ (1 mL) under N_2 for 24 h. [b] isolated yields.[c] The reaction was carried under air. [d] The catalyst (0.01 mmol) was used. [e] 10 equiv. of **2a** was used.

(V) Kinetic Isotope Effect (KIE) Study

1. Method of the radical trapping experiment.

To a reaction tube was added **1a** (51 mg, 0.2 mmol), cyclopentane **2a** (1.0 mL), di-*tert*-butyl peroxide DTBP (0.146 mL, 0.8 mmol), di-tert-butyl peroxide DTBP (0.146 mL, 0.8 mmol), $Co(acac)_2$ (7.0 mg, 10 mmol%), benzotrifluoride and TEMPO (1.0 mL), then was evacuated and purged with N_2 three times. The solution was kept at 140 °C for 24h. After the solution was cooled to room temperature, the crude mixture

was purified by silica gel column chromatography [ethyl acetate/petroleum ether (v/v, 1:6) as eluent] to give a corresponding product **3aa**.

2. Method of the kinetic isotope effect experiments

To a reaction tube was added **1a** (51 mg, 0.2 mmol), cyclopentane **2a** (1.0 mL), di-*tert*-butyl peroxide DTBP (0.146 mL, 0.8 mmol), $Co(acac)_2$ (7.0 mg, 10 mmol%) and benzotrifluoride (1.0 mL), then was evacuated and purged with N_2 three times. The solution was kept at 140 ^{o}C for 24h. After the solution was cooled to room temperature, the crude mixture was purified by silica gel column chromatography [ethyl acetate/petroleum ether (v/v, 1:6) as eluent] to give a corresponding product **3aa**.

By using above procedure, the similar sets of experiments were conducted by using [D_7]-**1a** instead of **1a** and using [D_{12}]-**2b** instead of **2b**, [D_8]-**2g** instead of **2g**, [D_8]-**2x** instead of **2x**. The KIE value for C(sp^3)-H bond cleavage of unactivated alkane was k_H/k_D = (4.5, 4.8, 4.4) and the KIE value for C(sp^2)-H bond cleavage of terminal alkyne was k_H /k_D = 0.7.

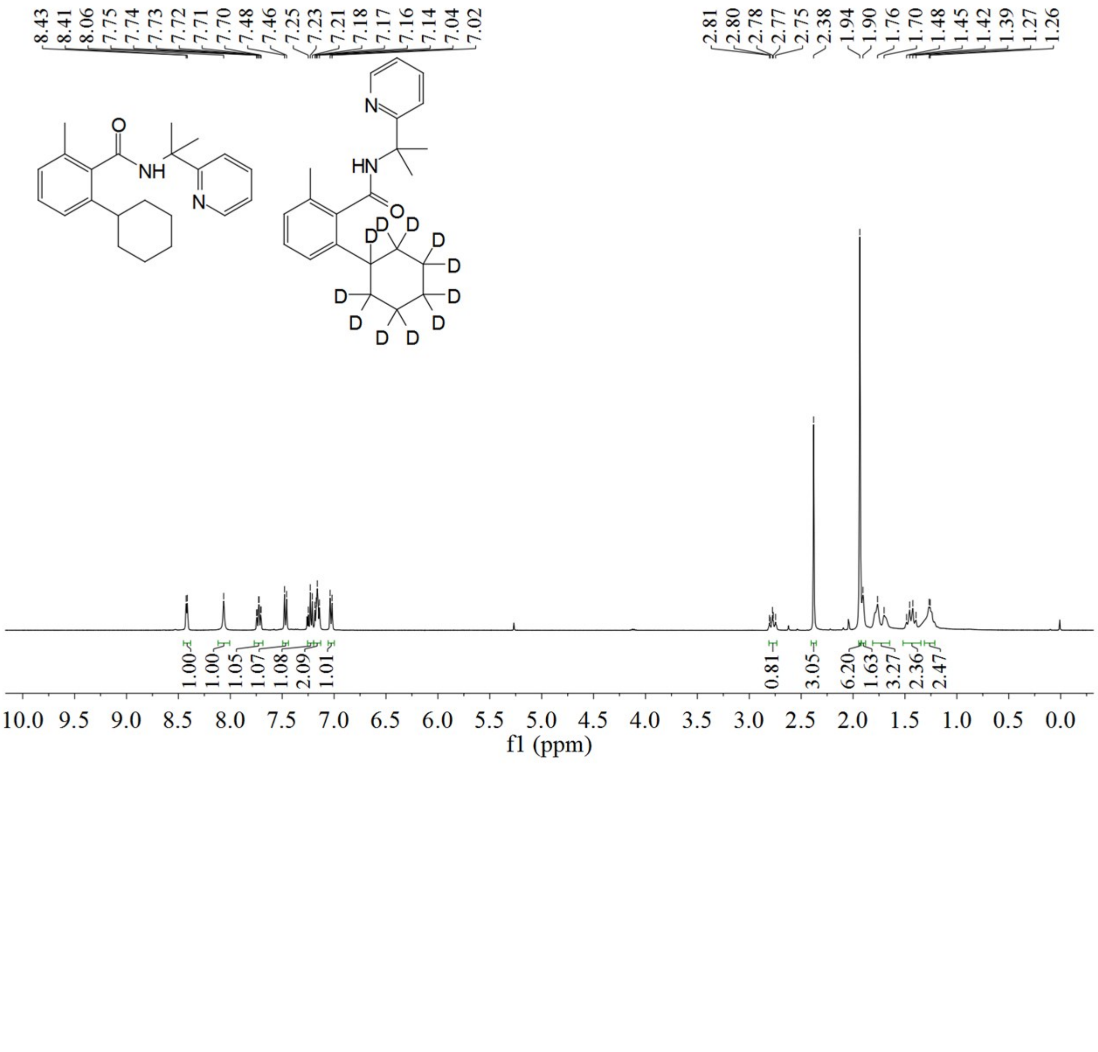

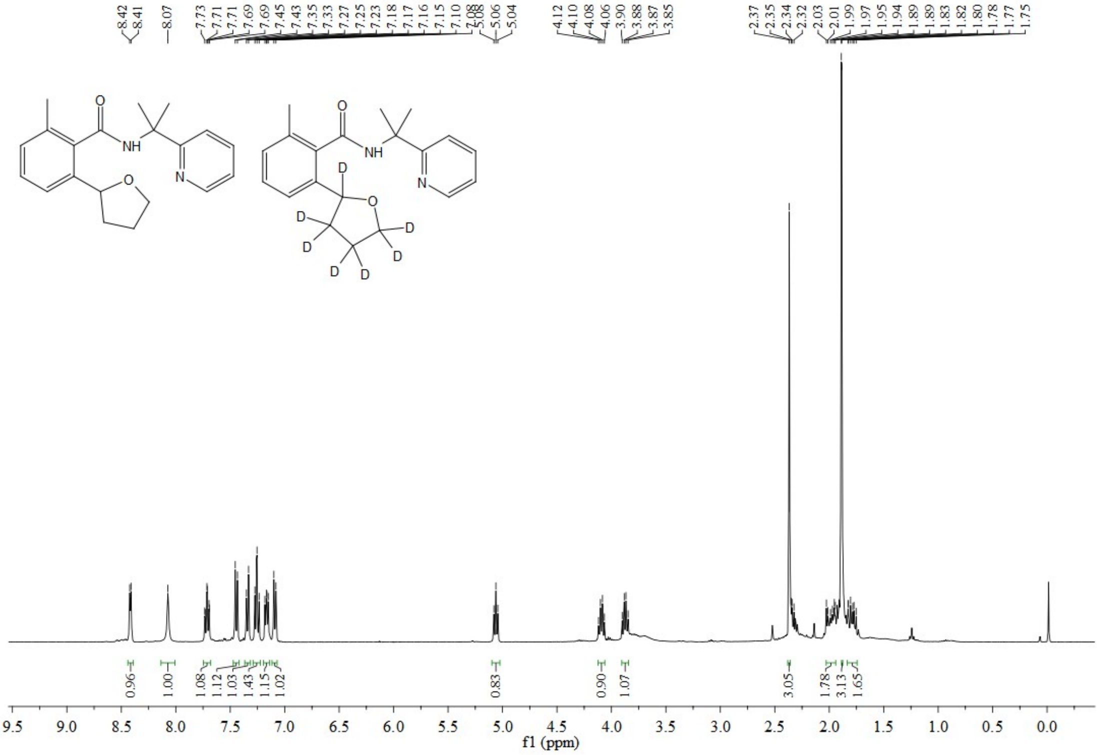
8.42
8.41
8.07
7.73
7.71
7.71
7.69
7.69
7.45
7.43
7.35
7.33
7.27
7.25
7.23
7.18
7.17
7.16
7.15
7.10
7.08
5.08
5.06
5.04
4.12
4.10
4.08
4.06
3.90
3.88
3.87
3.85
2.37
2.35
2.34
2.32
2.03
2.01
1.99
1.97
1.95
1.94
1.89
1.89
1.83
1.82
1.80
1.78
1.77
1.75
O
NH
N
O
D
0.96
1.00
1.08
1.12
1.03
1.43
1.15
1.02
0.83
0.90
1.07
3.05
1.78
3.13
1.65
9.5 9.0 8.5 8.0 7.5 7.0 6.5 6.0 5.5 5.0 4.5 4.0 3.5 3.0 2.5 2.0 1.5 1.0 0.5 0.0
f1 (ppm)

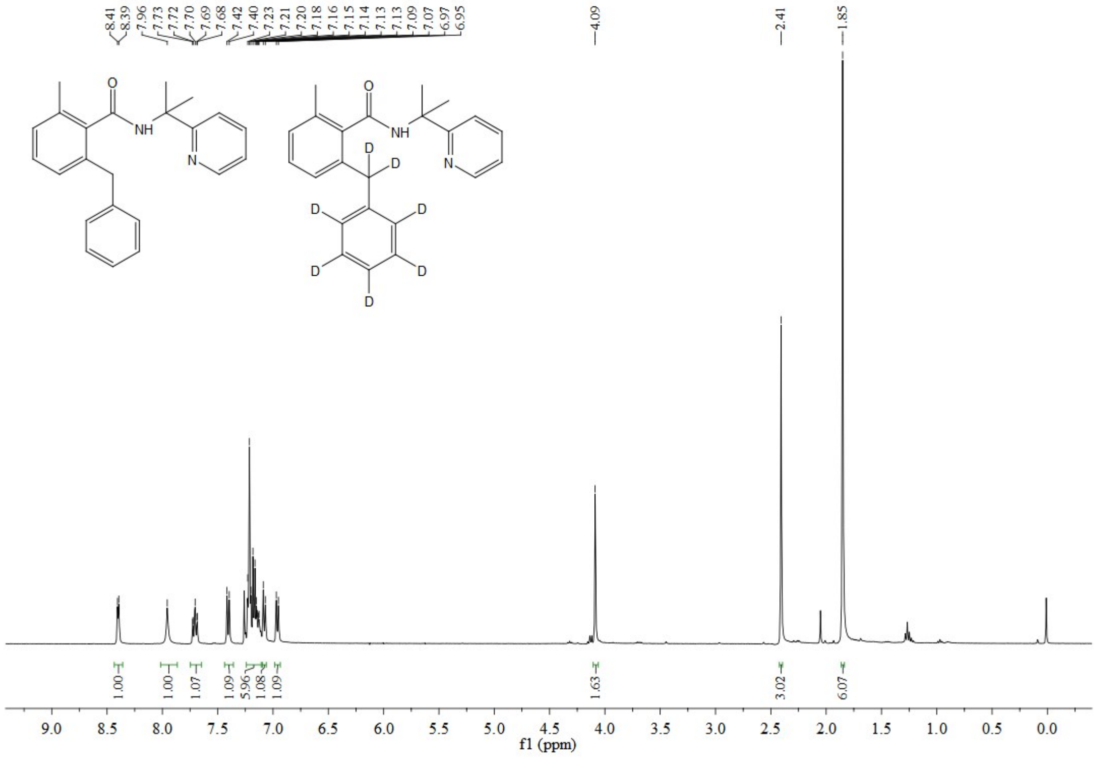
8.41
8.39
7.96
7.73
7.72
7.70
7.69
7.68
7.42
7.40
7.23
7.21
7.20
7.18
7.16
7.15
7.14
7.13
7.09
7.07
6.97
6.95
4.09
2.41
1.85
O
NH
N
D
1.00
1.00
1.07
1.09
5.96
1.08
1.09
1.63
3.02
6.07
9.0 8.5 8.0 7.5 7.0 6.5 6.0 5.5 5.0 4.5 4.0 3.5 3.0 2.5 2.0 1.5 1.0 0.5 0.0
f1 (ppm)

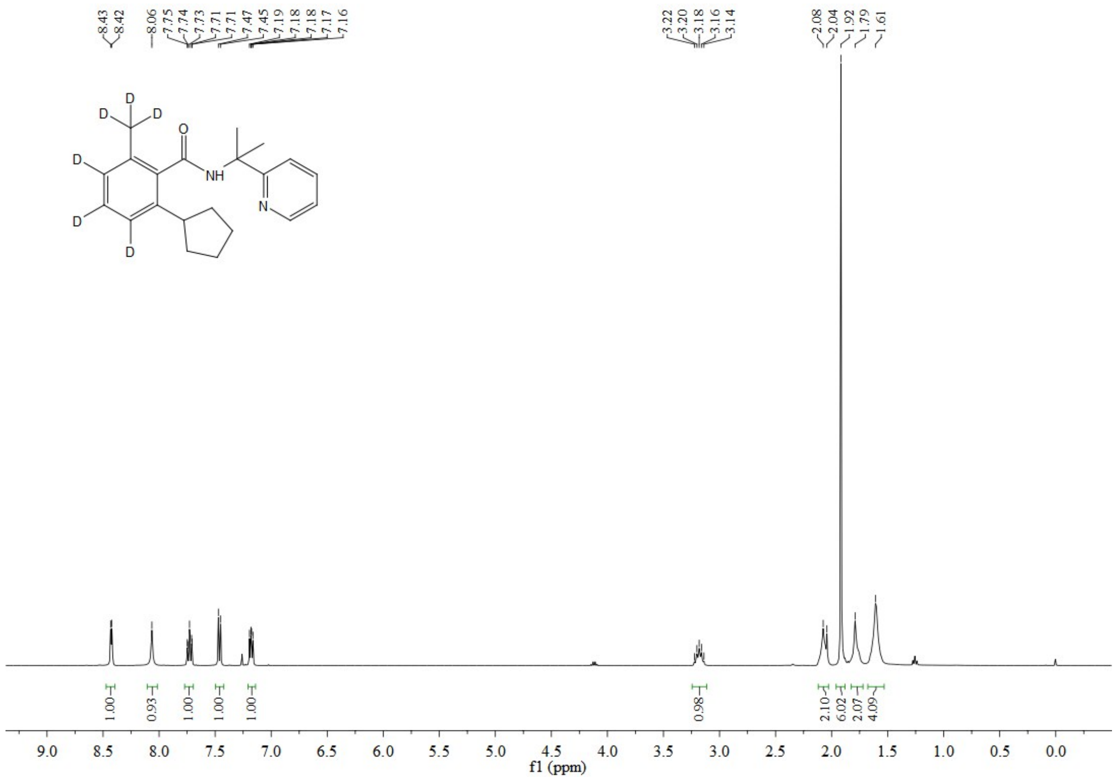

(VI) Cleavage of the PIP Directing Group

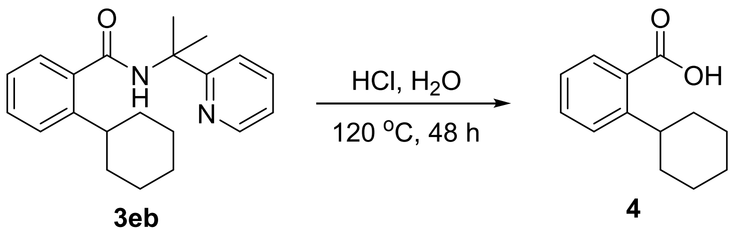

To a 50 mL Schlenk tube was added **3eb** (0.2 mmol), and conc. HCl (2 mL). The mixture was then heated at 120 °C for 48 hours. The reaction mixture was cooled to room temperature and the aqueous phase was extracted with dichloromethane (3×10 mL). The combined organic phase was dried with anhydrous magnesium sulfate. After concentration, the mixture was purified by flash chromatography to give target product **4** as a white solid (25mg, 61%).

2-cyclohexyl-N-(2-(pyridin-2-yl)propan-2-yl)benzamide(3eb)

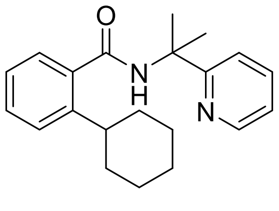

Yellow oil. R_f 0.40 (PE/EtOAc = 3/1). ^{1}H NMR (500 MHz, $CDCl_3$) δ 8.51 – 8.43 (m, 1H), 7.98 (s, 1H), 7.73 (td, J = 7.9, 1.7 Hz, 1H), 7.48 (d, J = 8.1 Hz, 1H), 7.40 (d, J = 7.3 Hz, 1H), 7.35 – 7.31 (m, 2H), 7.21 – 7.17 (m, 2H), 3.01 (tt, J = 11.9, 3.2 Hz, 1H), 1.90 (s, 6H), 1.80 (ddd, J = 53.3, 23.6, 7.3 Hz, 4H), 1.48 – 1.23 (m, 6H). ^{13}C NMR (126 MHz, $CDCl_3$) δ 169.9, 164.4, 147.6, 145.2, 137.7, 137.2, 129.4, 126.8, 126.5, 125.5, 121.9, 119.5, 57.1, 40.4, 34.5, 27.5, 27.0, 26.2.; HRMS (ESI) m/z: calculated for $C_{22}H_{29}N_2O$ $[M + H]^+$: 337.2274, found: 337.2268.

2-cyclohexylbenzoic acid(4)

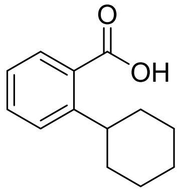

Yellow oil (25 mg, 61%). R_f 0.15 (PE/EtOAc = 1/1). ^{1}H NMR (500 MHz, DMSO) δ 12.82 (s, 1H), 7.62 (dd, J = 7.7, 1.2 Hz, 1H), 7.48 – 7.43 (m, 1H), 7.40 (d, J = 7.1 Hz, 1H), 7.24 (td, J = 7.6, 1.2 Hz, 1H), 3.35 – 3.24 (m, 1H), 1.83 – 1.66 (m, 5H), 1.48 – 1.21 (m, 5H). ^{13}C NMR (126 MHz, DMSO) δ 170.1, 147.5, 131.8, 131.6, 129.6, 127.0, 126.0, 34.3, 27.1, 26.1.; HRMS (ESI) m/z: calculated for $C_{13}H_{15}O_2$ $[M + H]^+$: 203.1078, found: 203.1073.

(VII) Copies of ^{1}H NMR and ^{13}C NMR Spectra

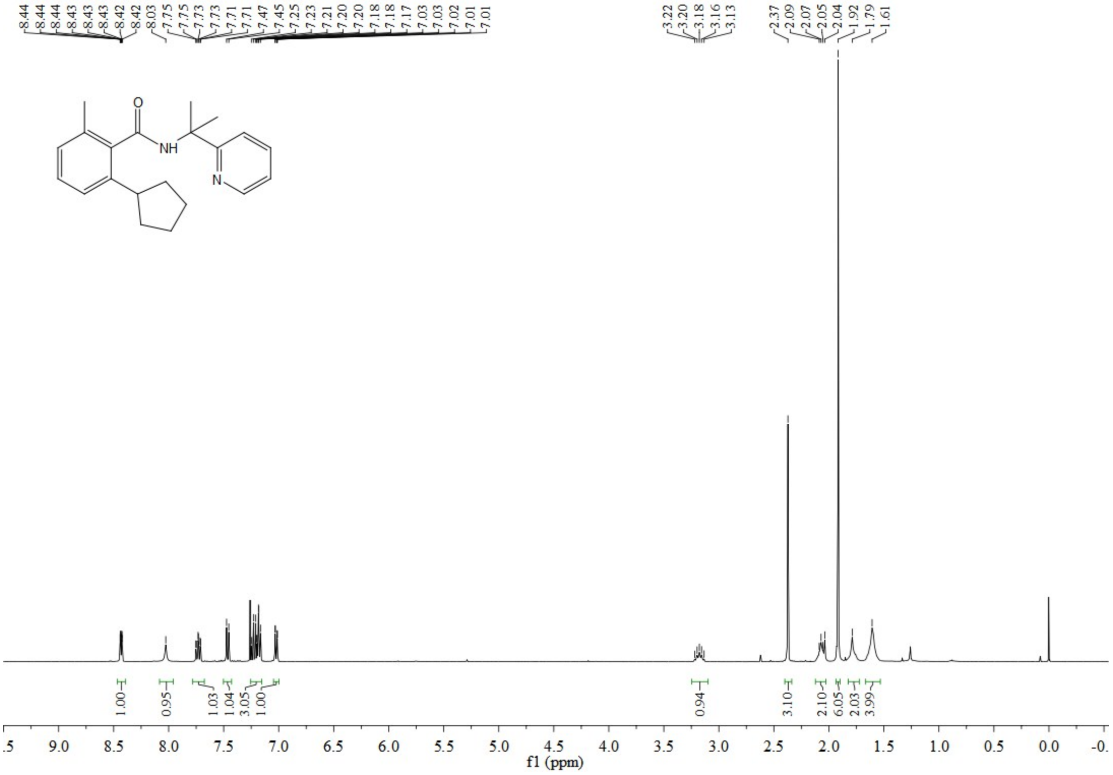

^{1}H NMR spectrum of compound **3aa**

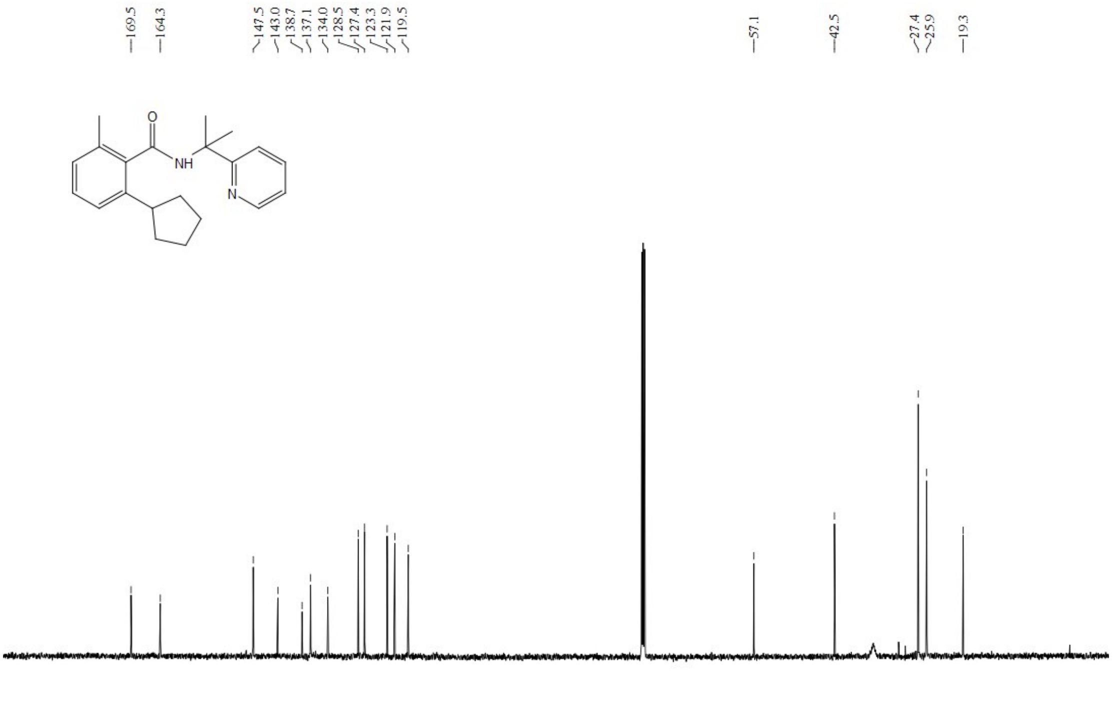

^{13}C NMR spectrum of compound **3aa**

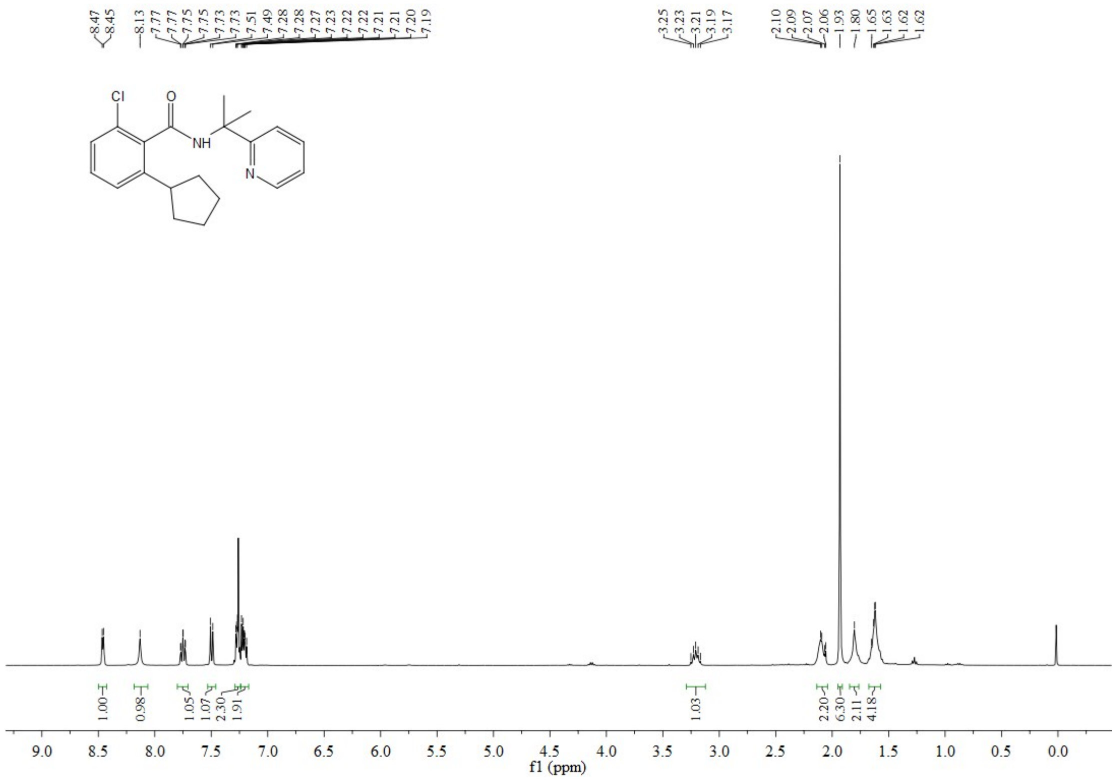

^{1}H NMR spectrum of compound **3ba**

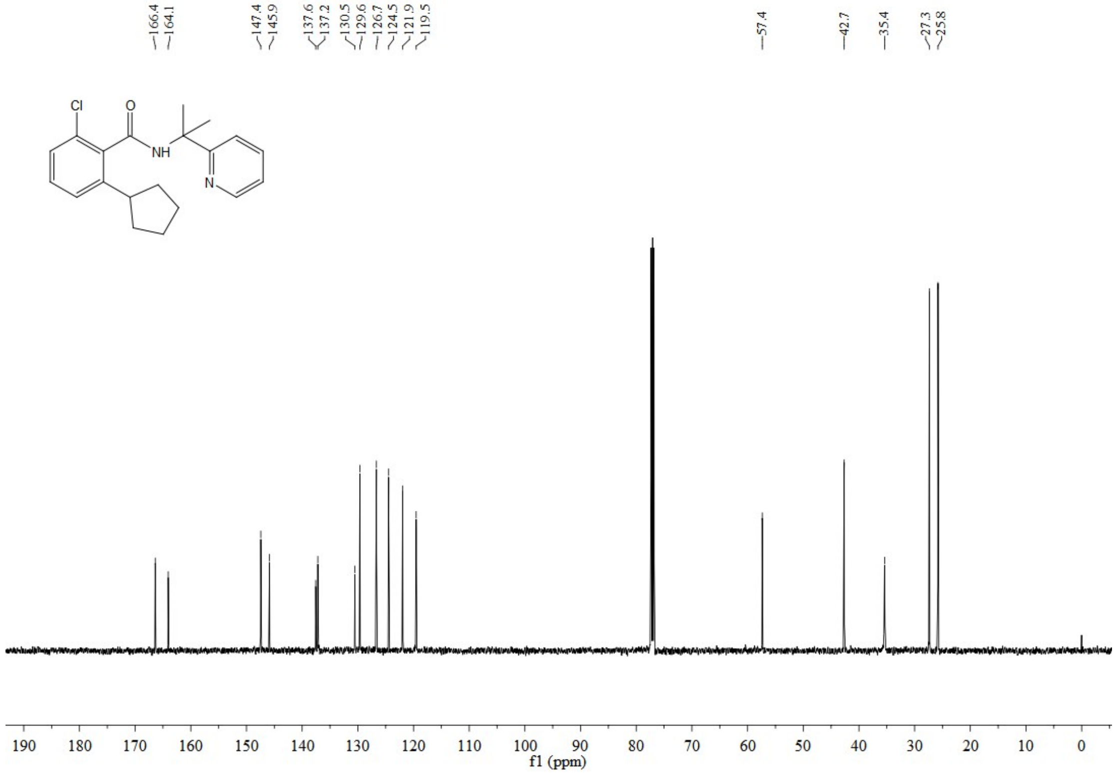

^{13}C NMR spectrum of compound **3ba**

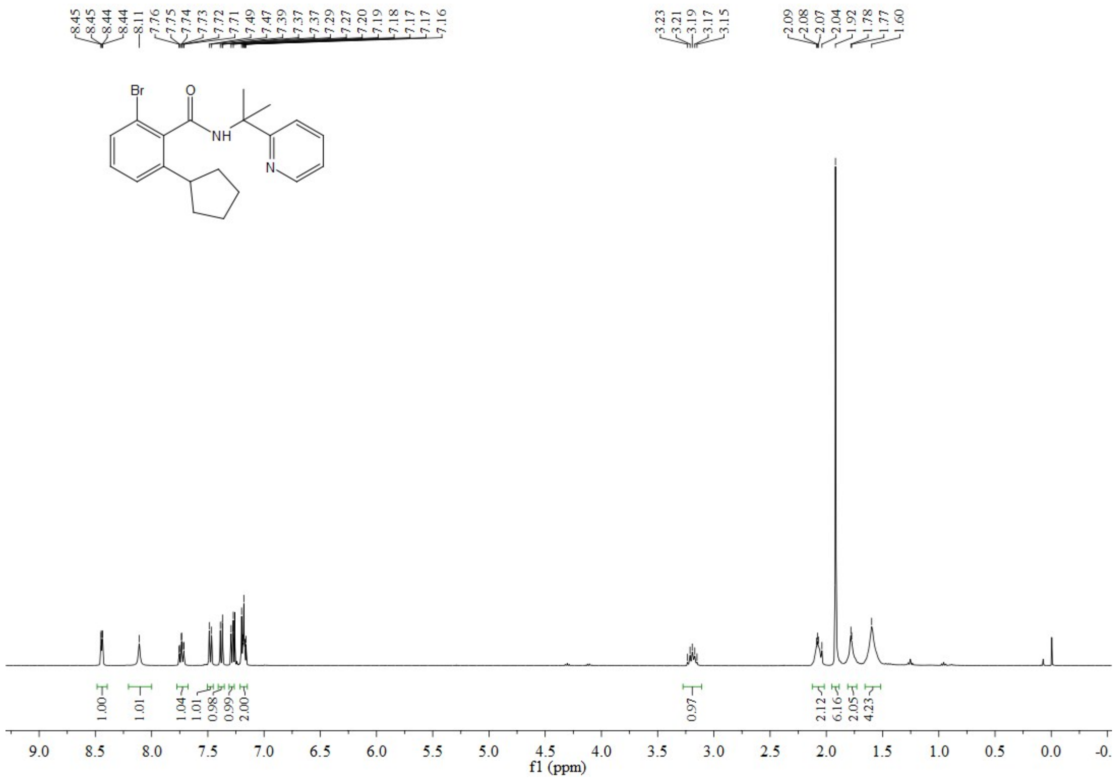

^{1}H NMR spectrum of compound **3ca**

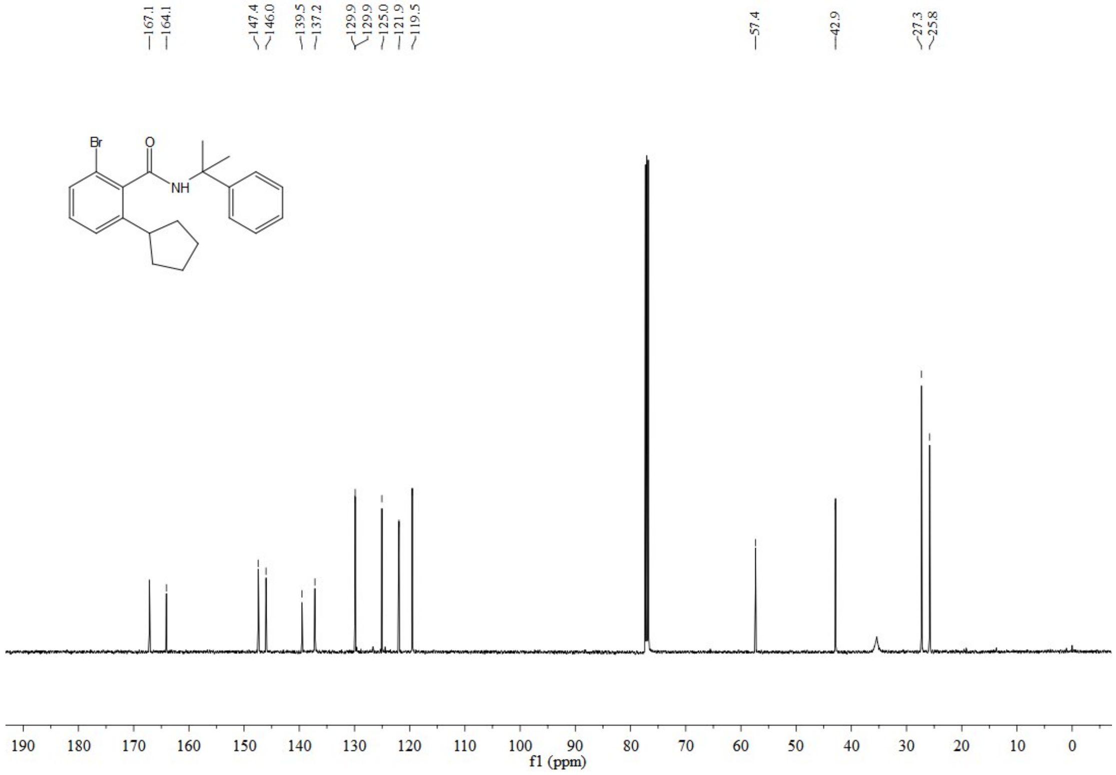

^{13}C NMR spectrum of compound **3ca**

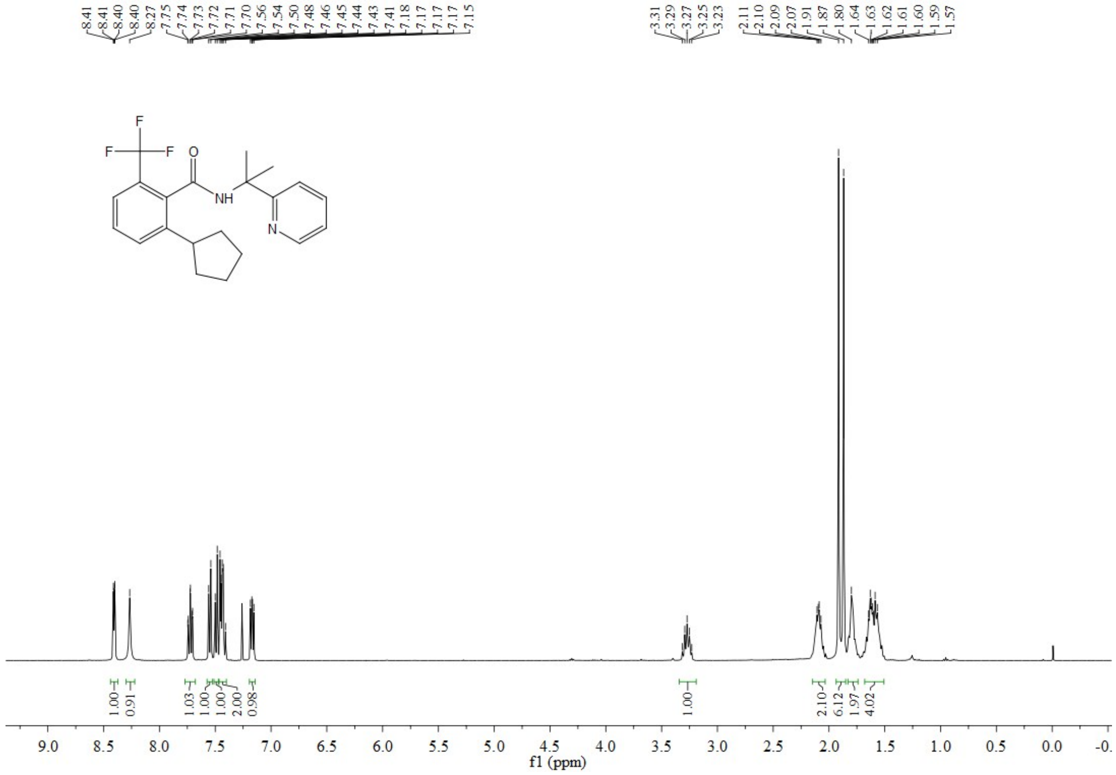

^{1}H NMR spectrum of compound **3da**

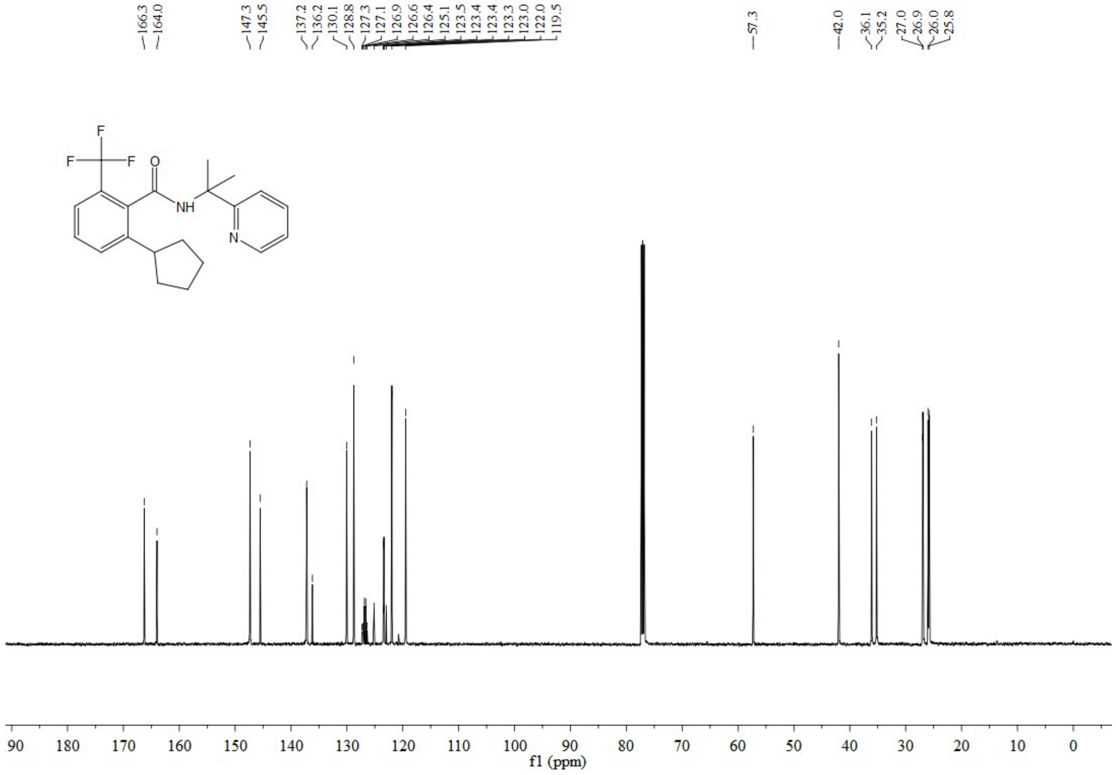

^{13}C NMR spectrum of compound **3da**

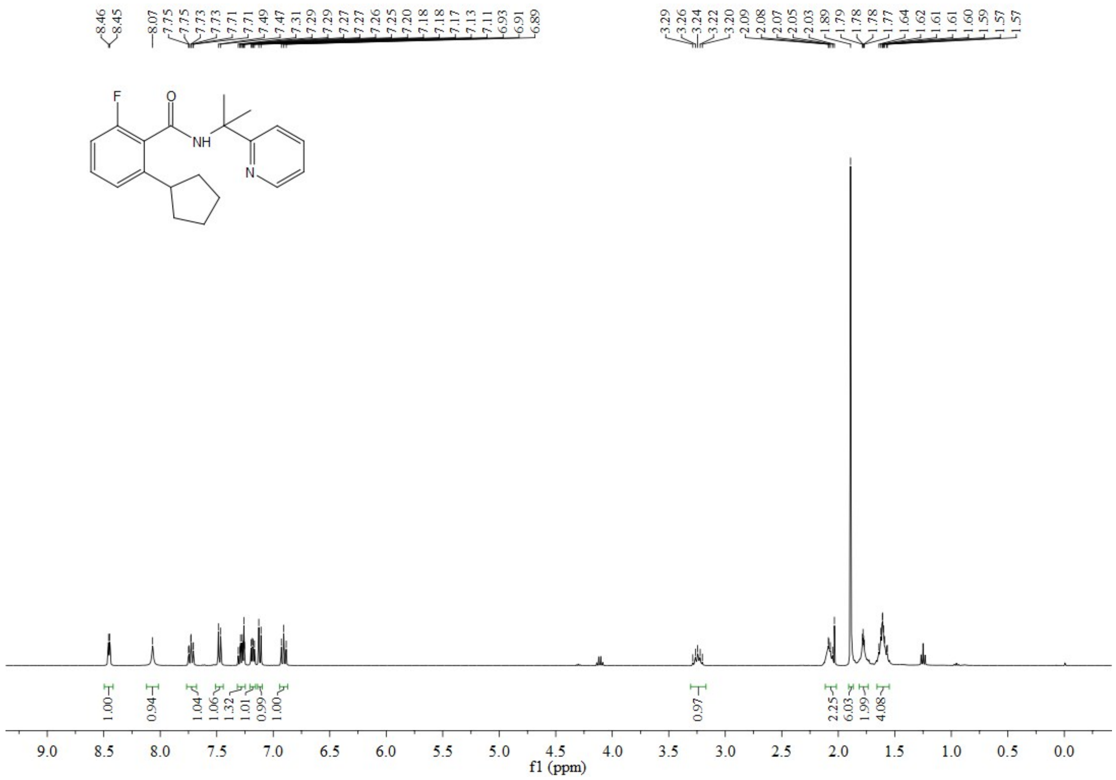

^{1}H NMR spectrum of compound **3ea**

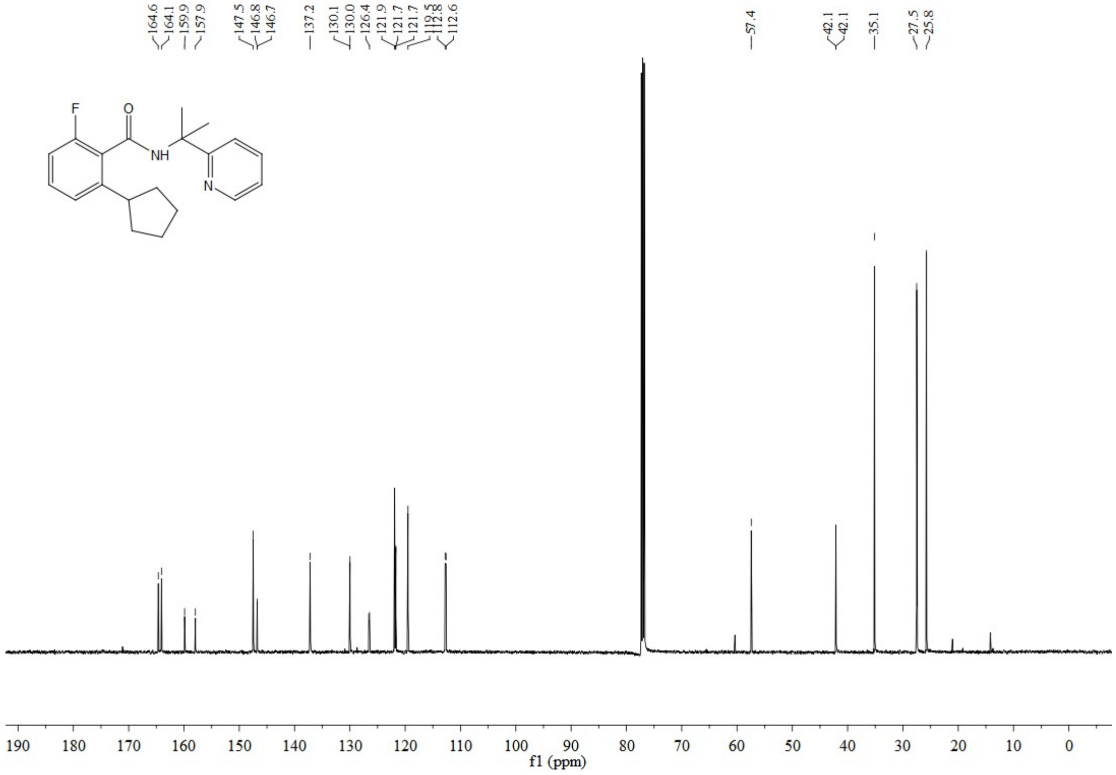

^{13}C NMR spectrum of compound **3ea**

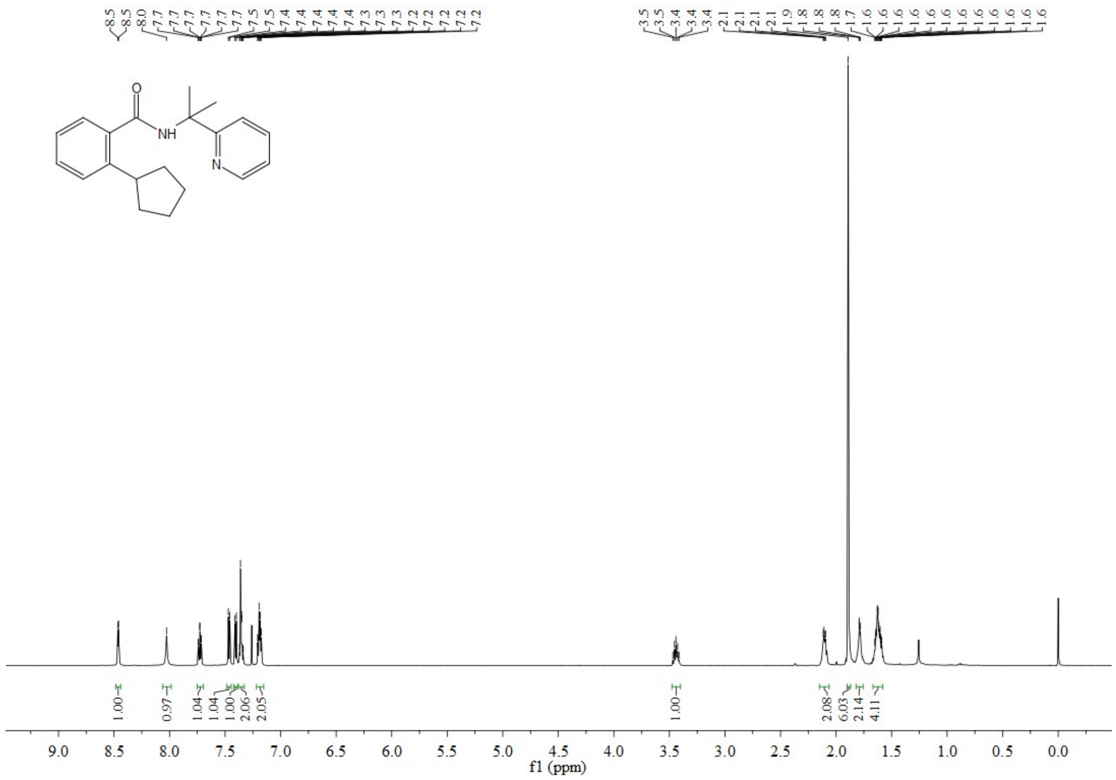

^{1}H NMR spectrum of compound **3fa**

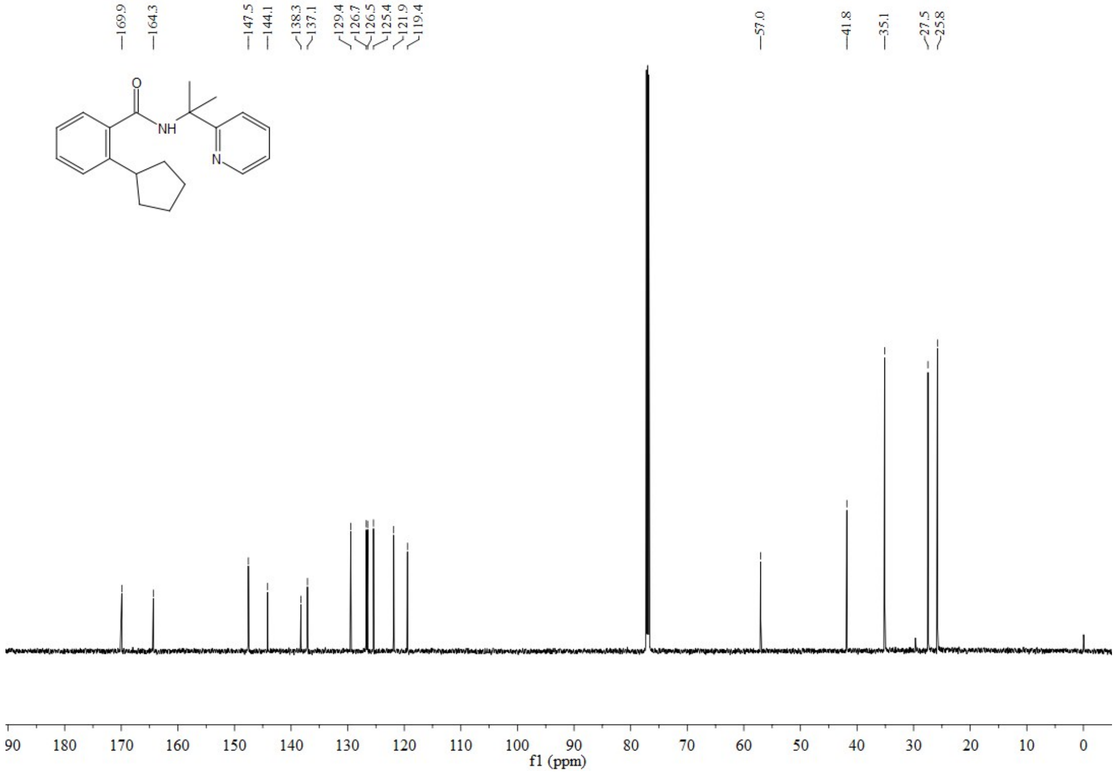

^{13}C NMR spectrum of compound **3fa**

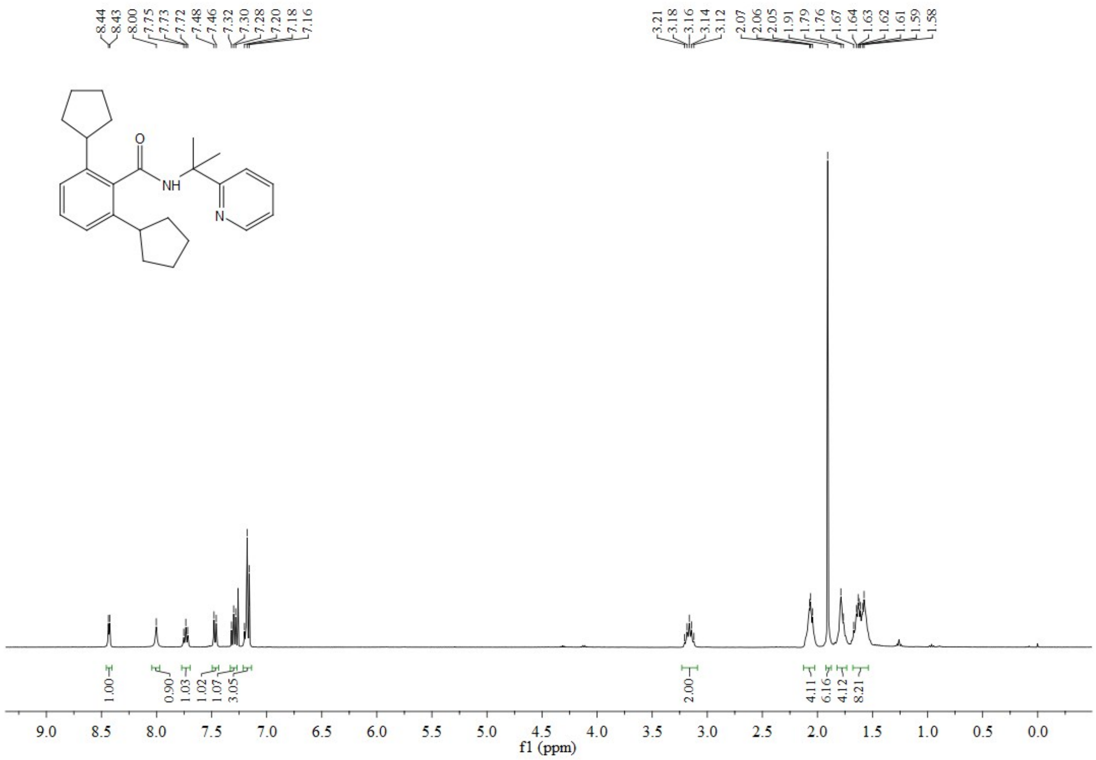

^{1}H NMR spectrum of compound **3fa'**

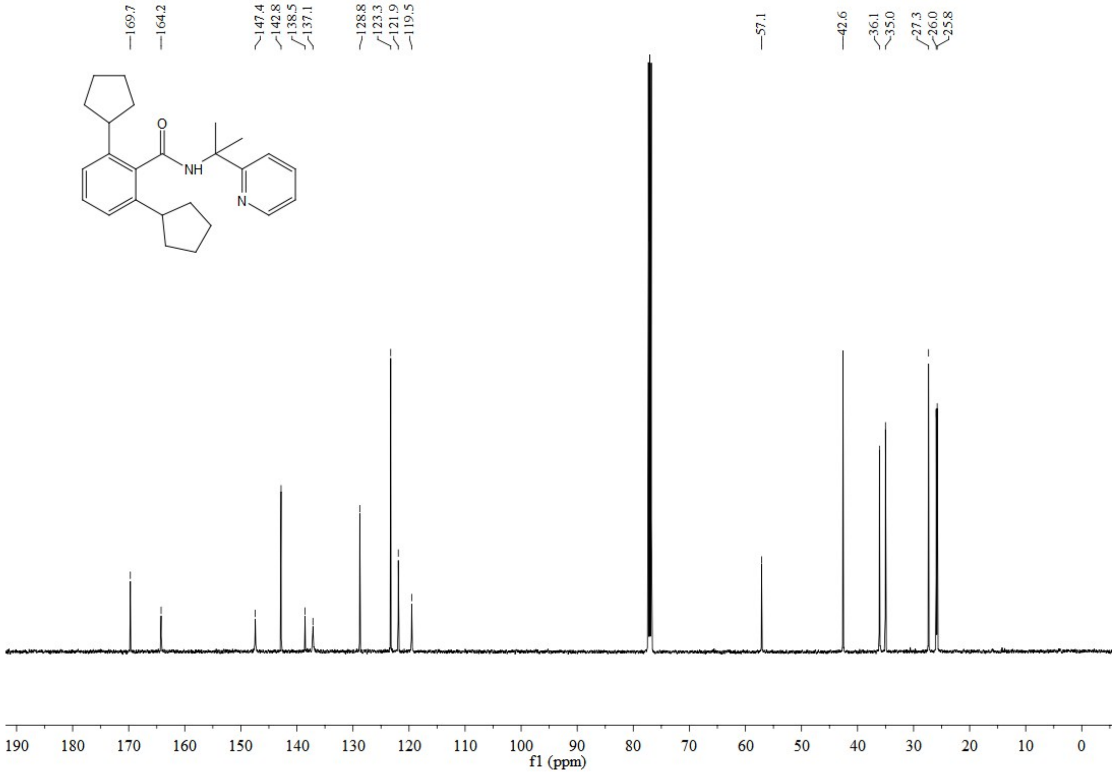

^{13}C NMR spectrum of compound **3fa'**

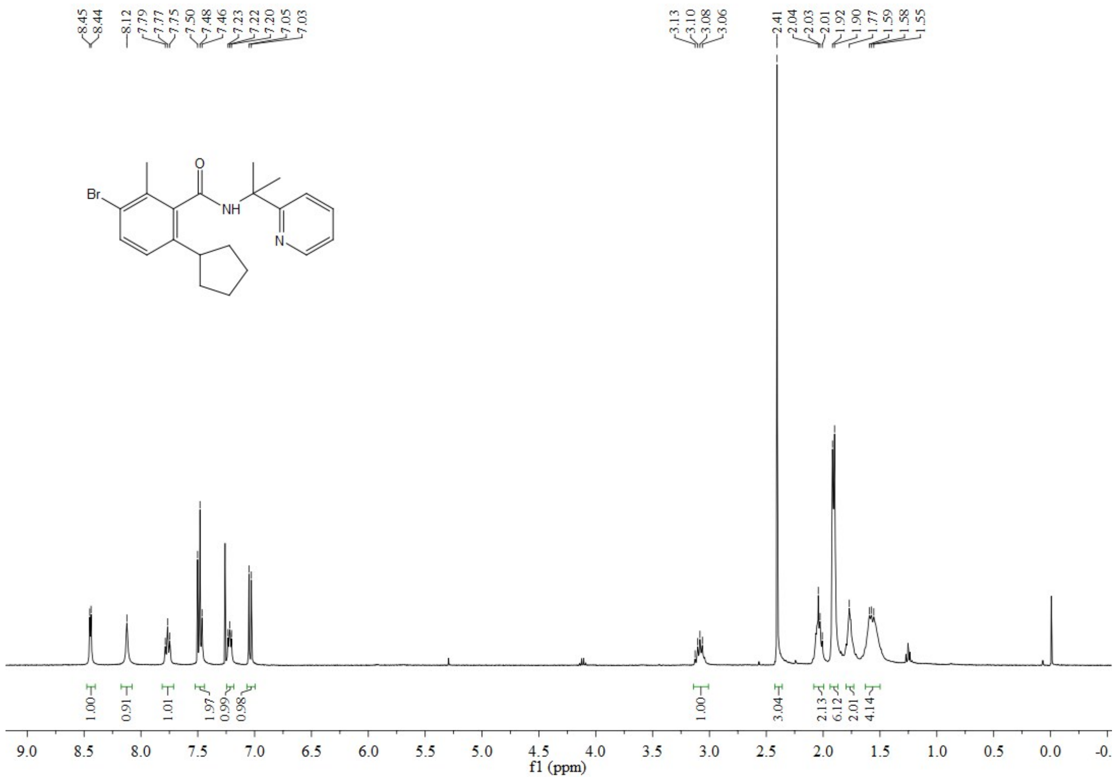

^{1}H NMR spectrum of compound **3ga**

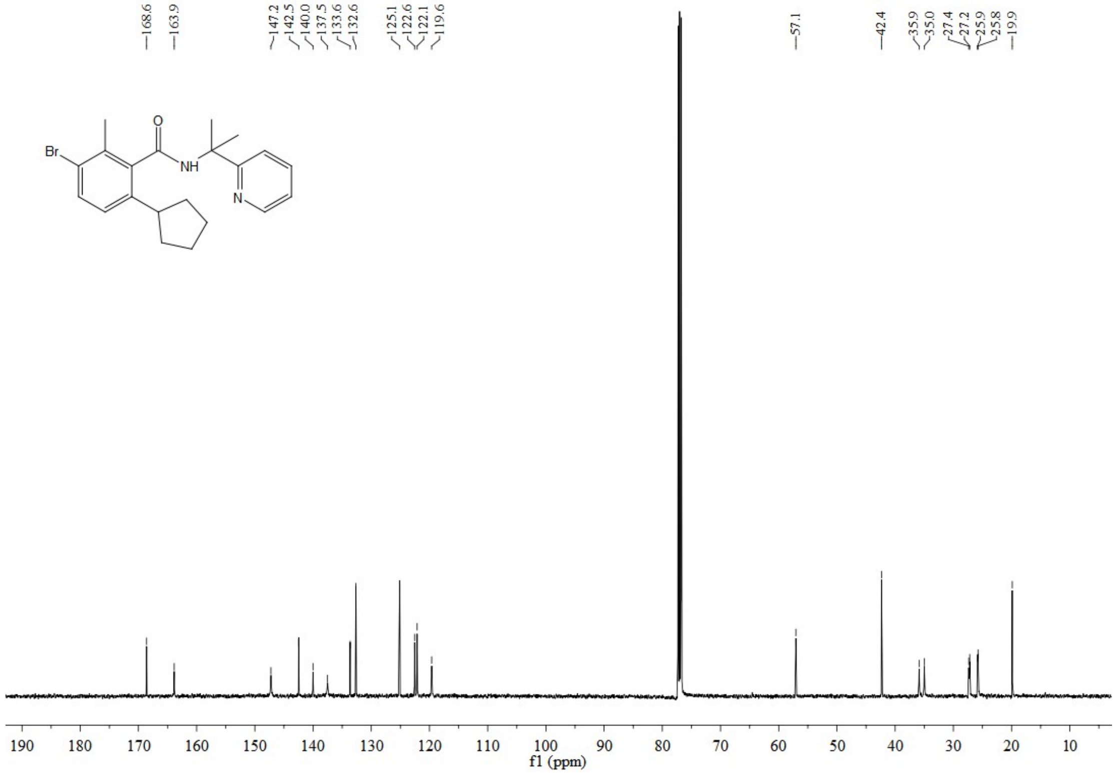

^{13}C NMR spectrum of compound **3ga**

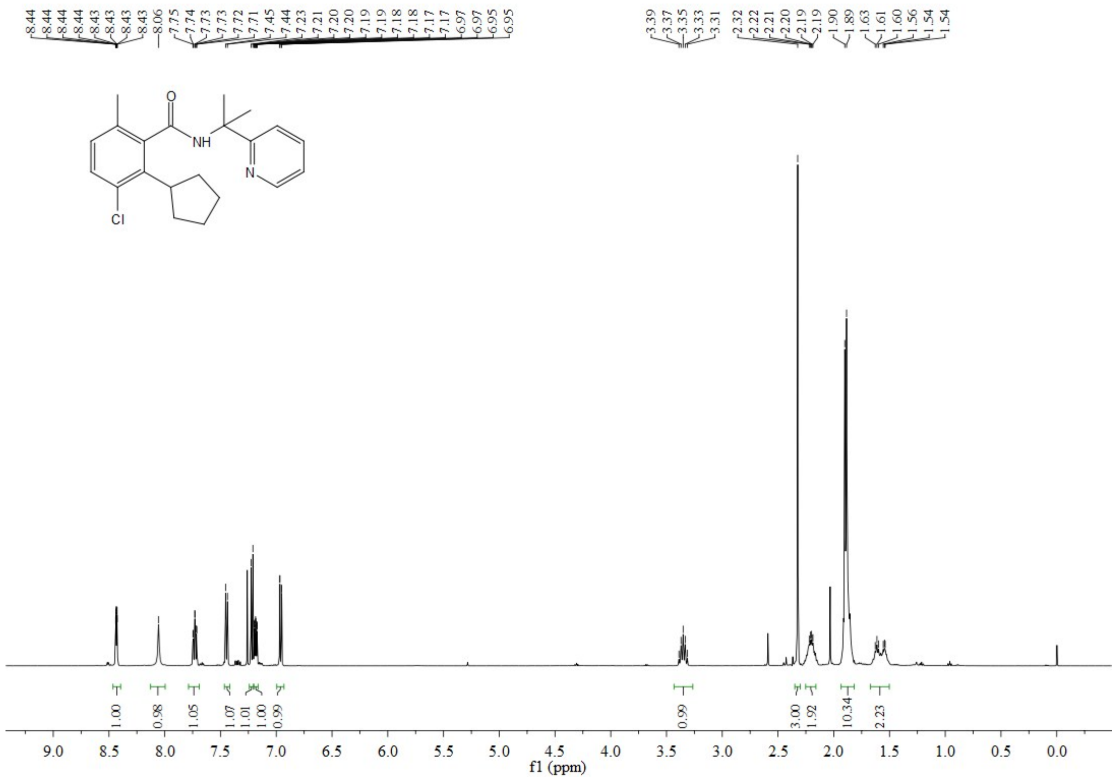

^{1}H NMR spectrum of compound **3ha**

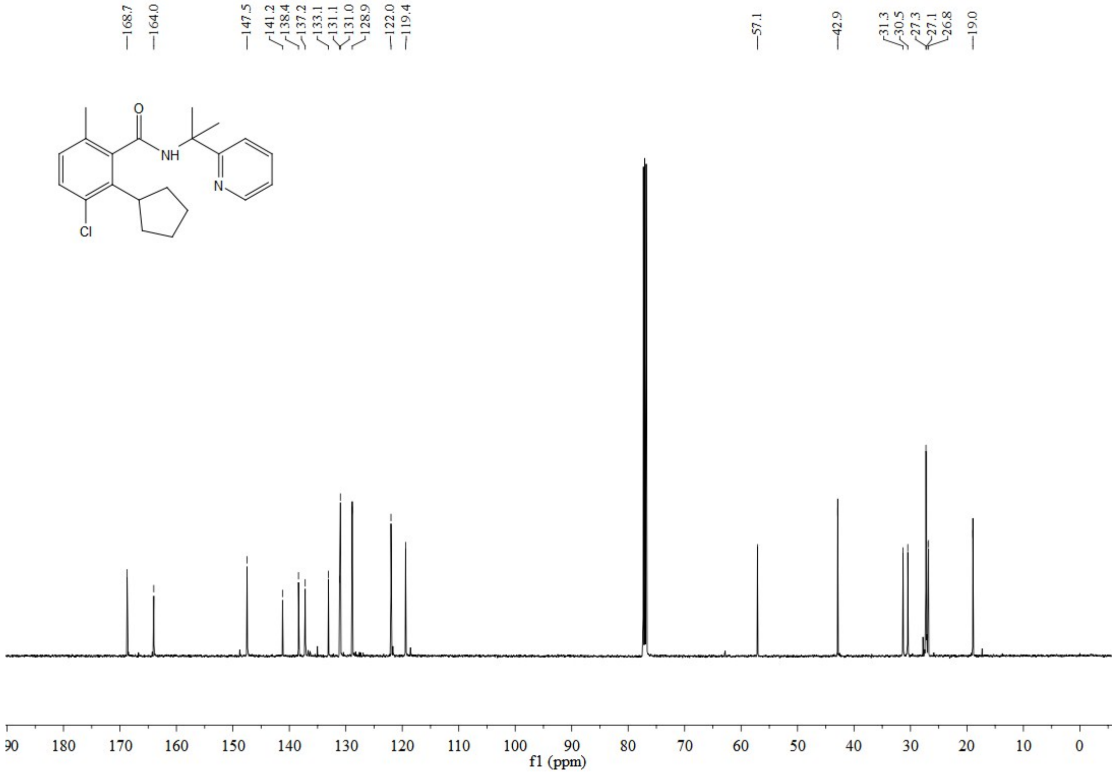

^{13}C NMR spectrum of compound **3ha**

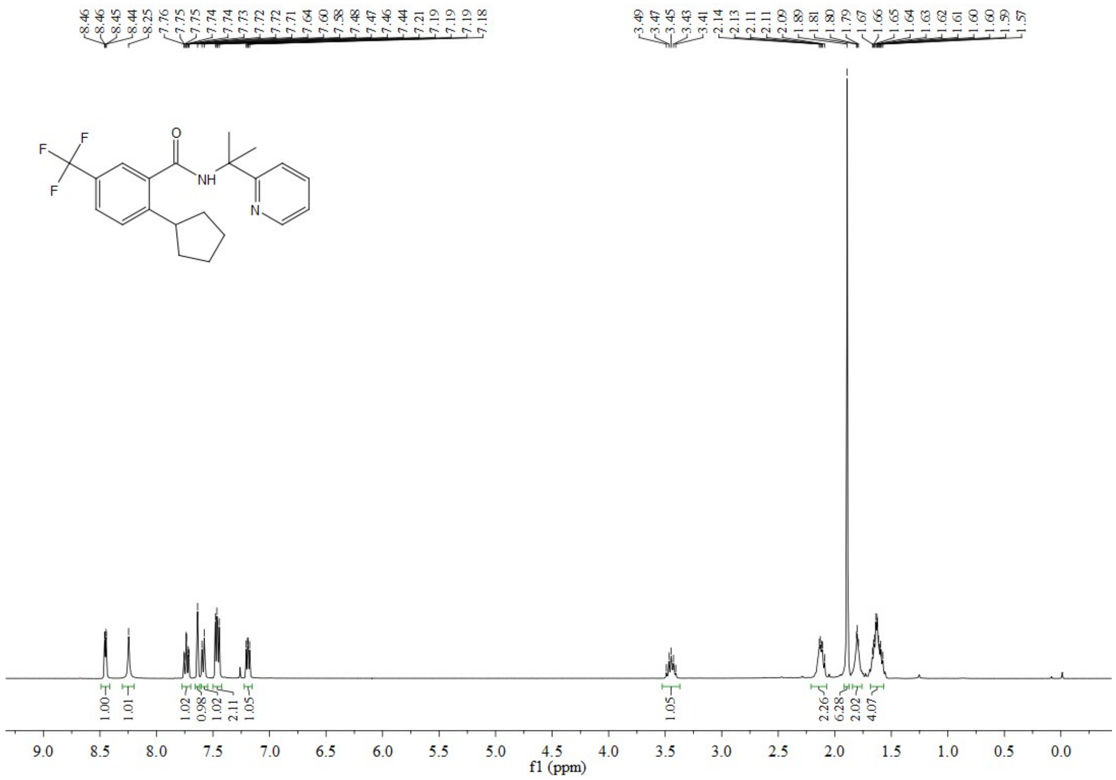

^{1}H NMR spectrum of compound **3ia**

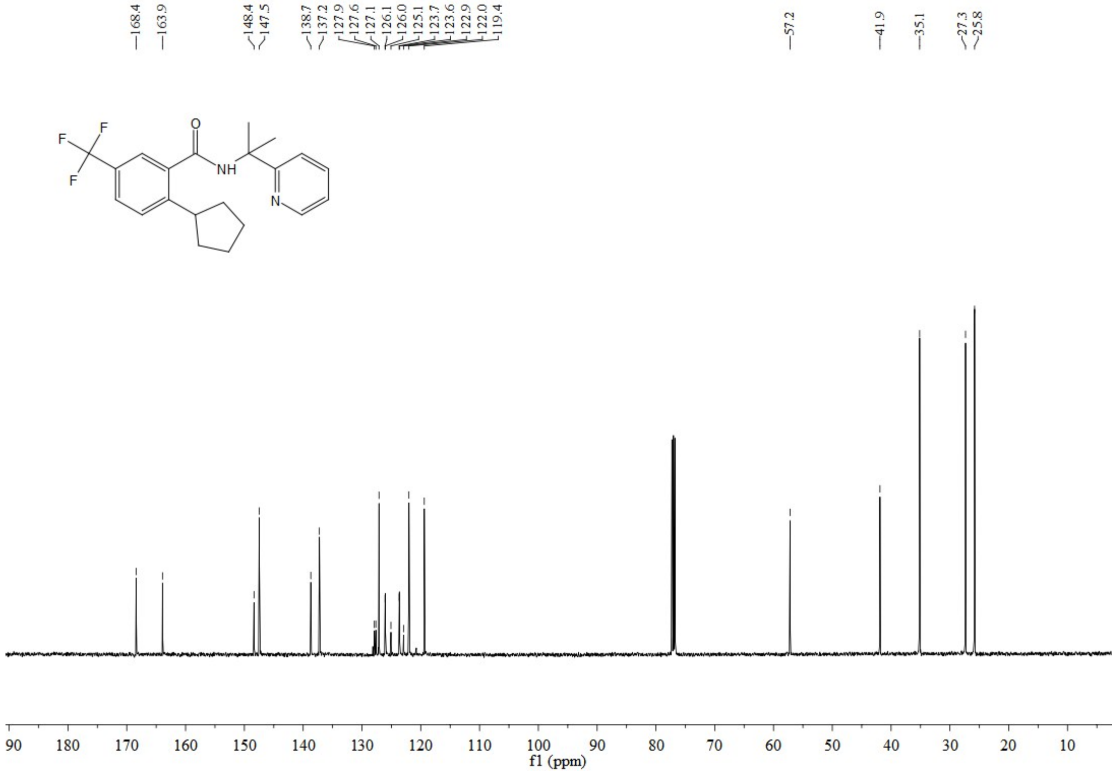

^{13}C NMR spectrum of compound **3ia**

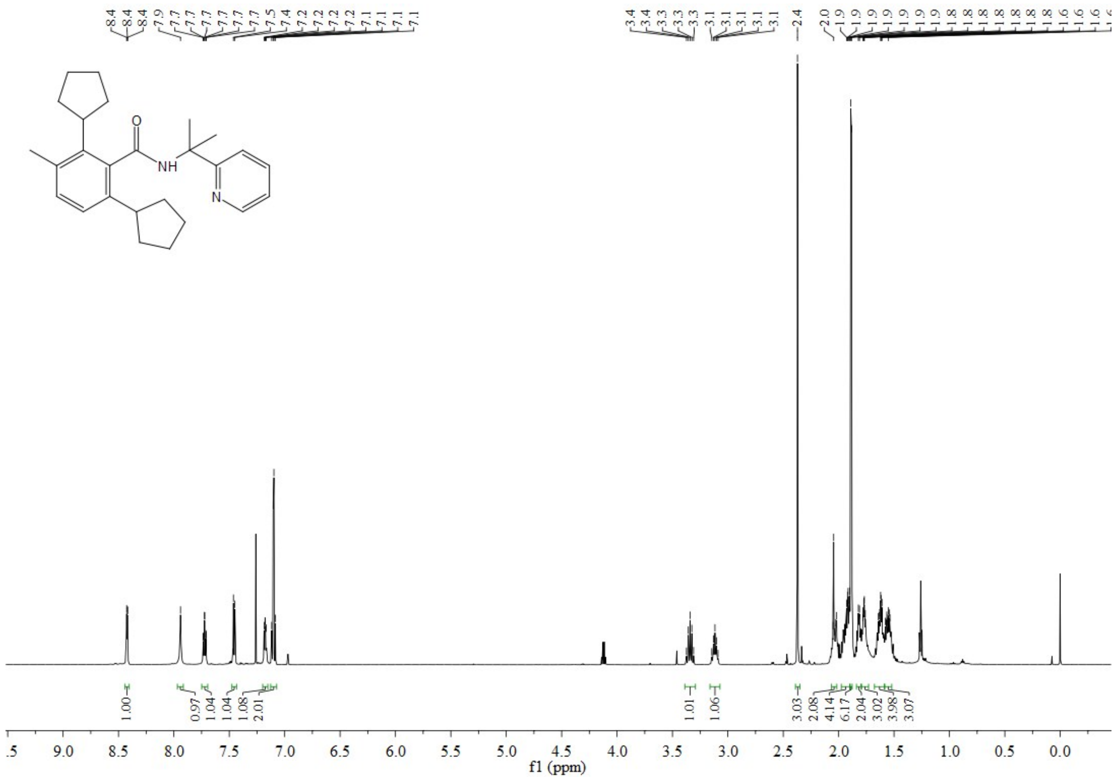

^{1}H NMR spectrum of compound **3ja'**

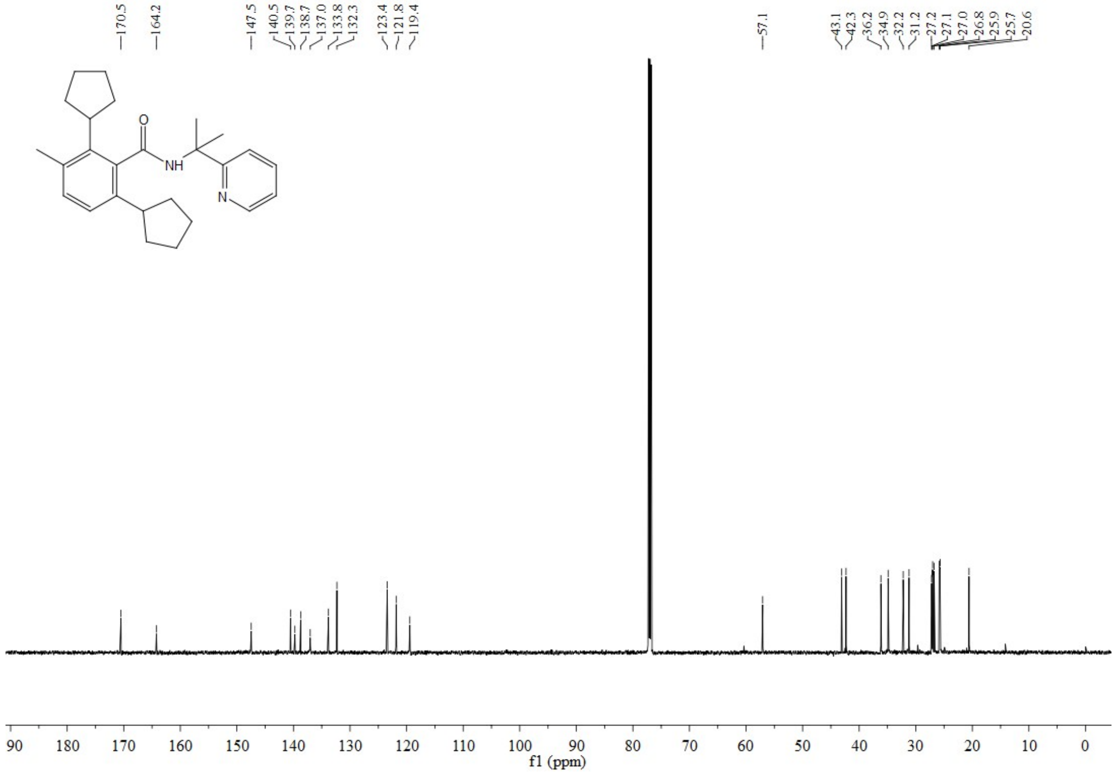

^{13}C NMR spectrum of compound **3ja'**

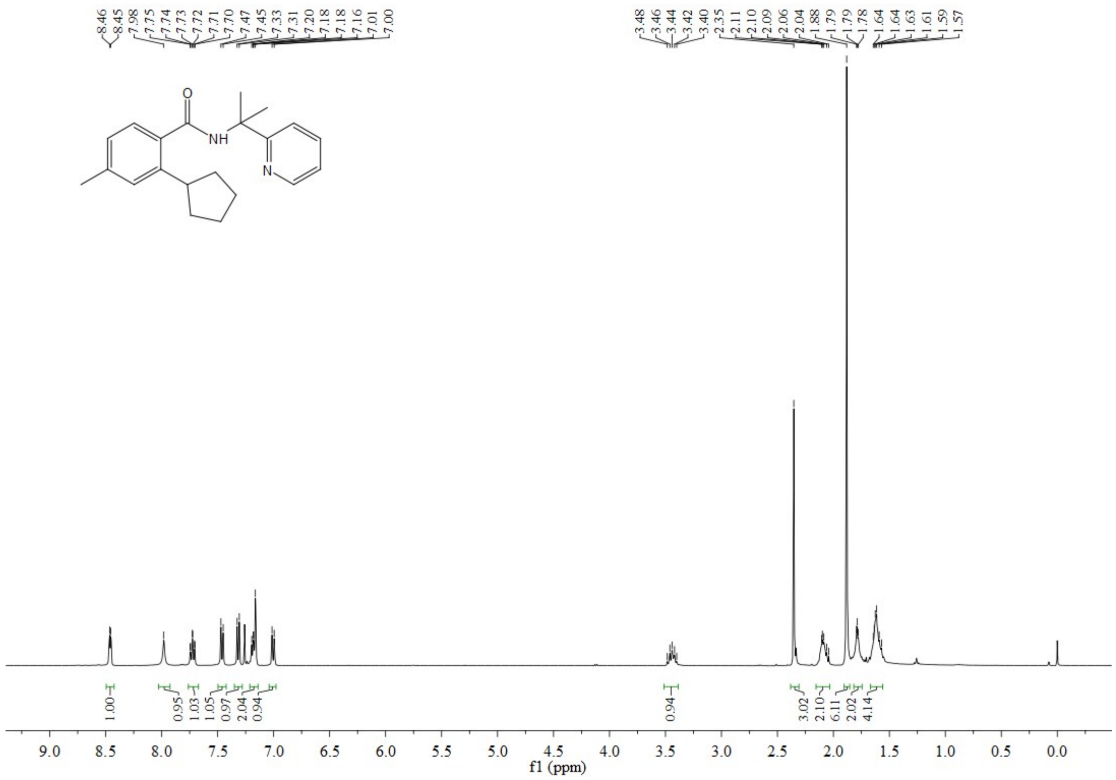

^{1}H NMR spectrum of compound **3ka**

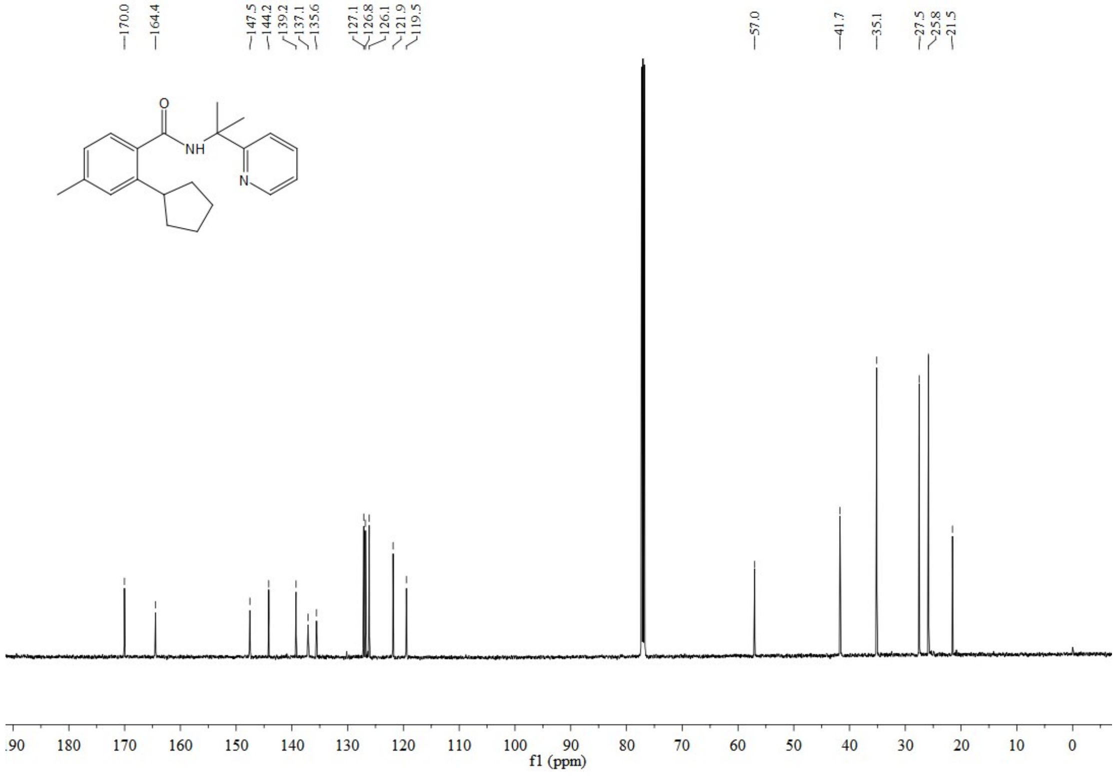

^{13}C NMR spectrum of compound **3ka**

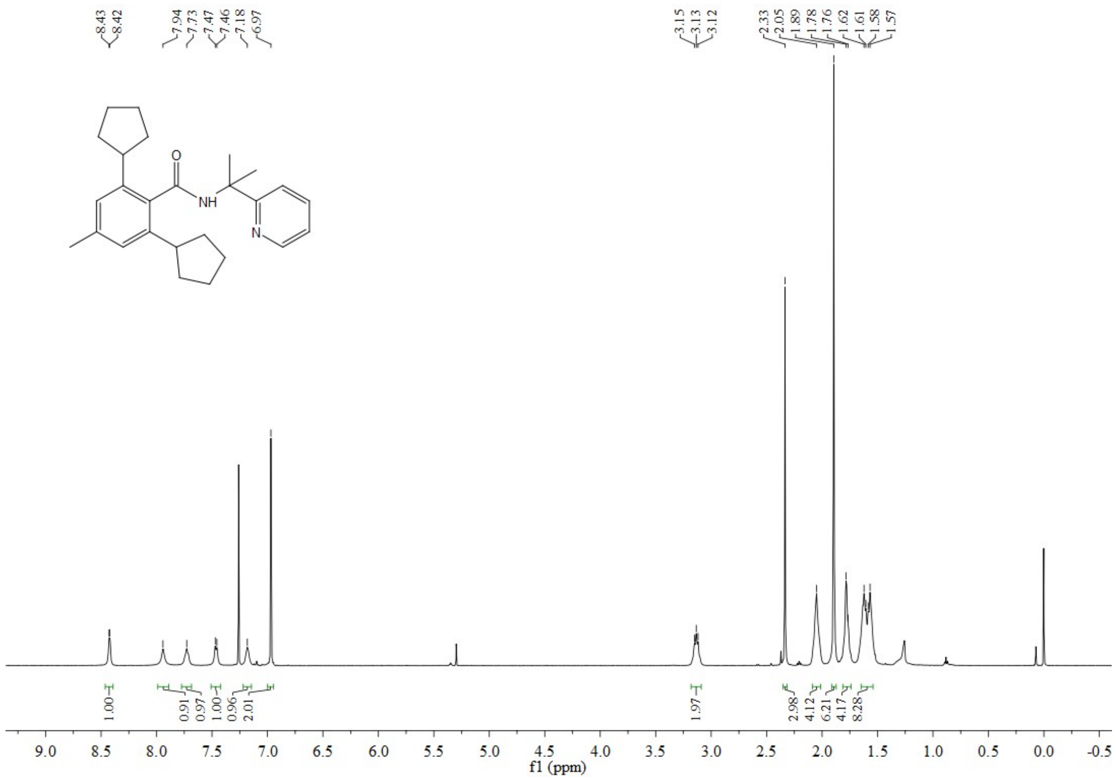

^{1}H NMR spectrum of compound **3ka'**

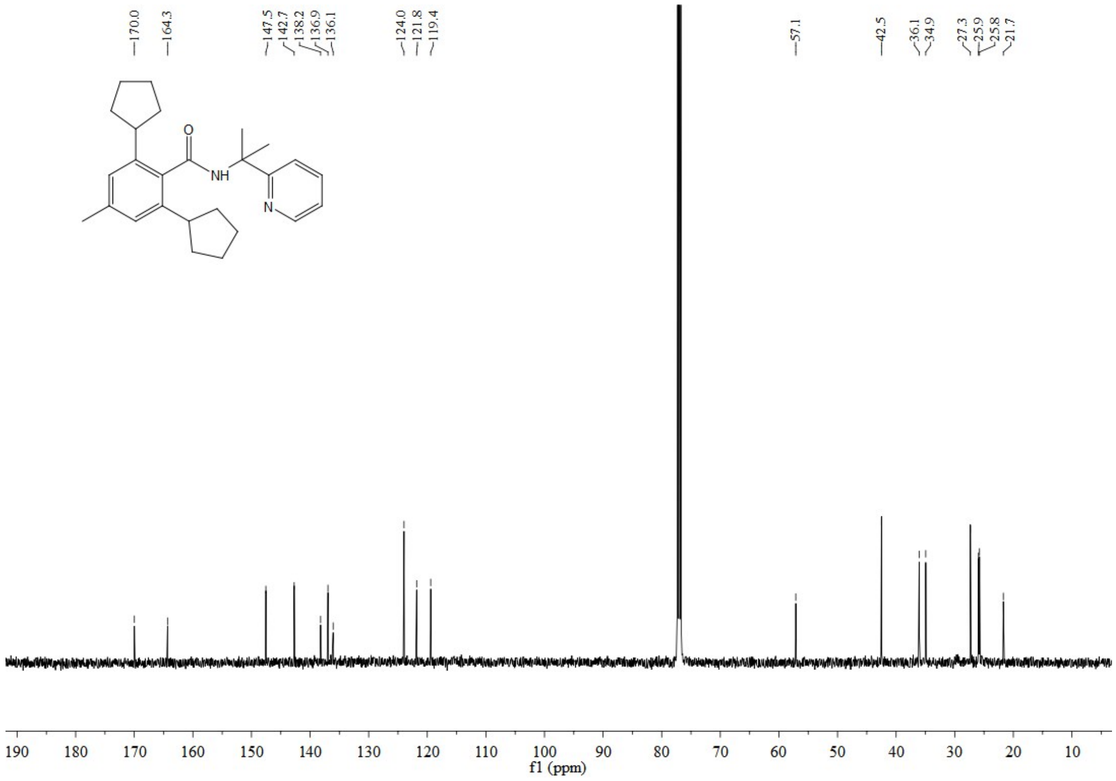

^{13}C NMR spectrum of compound **3ka'**

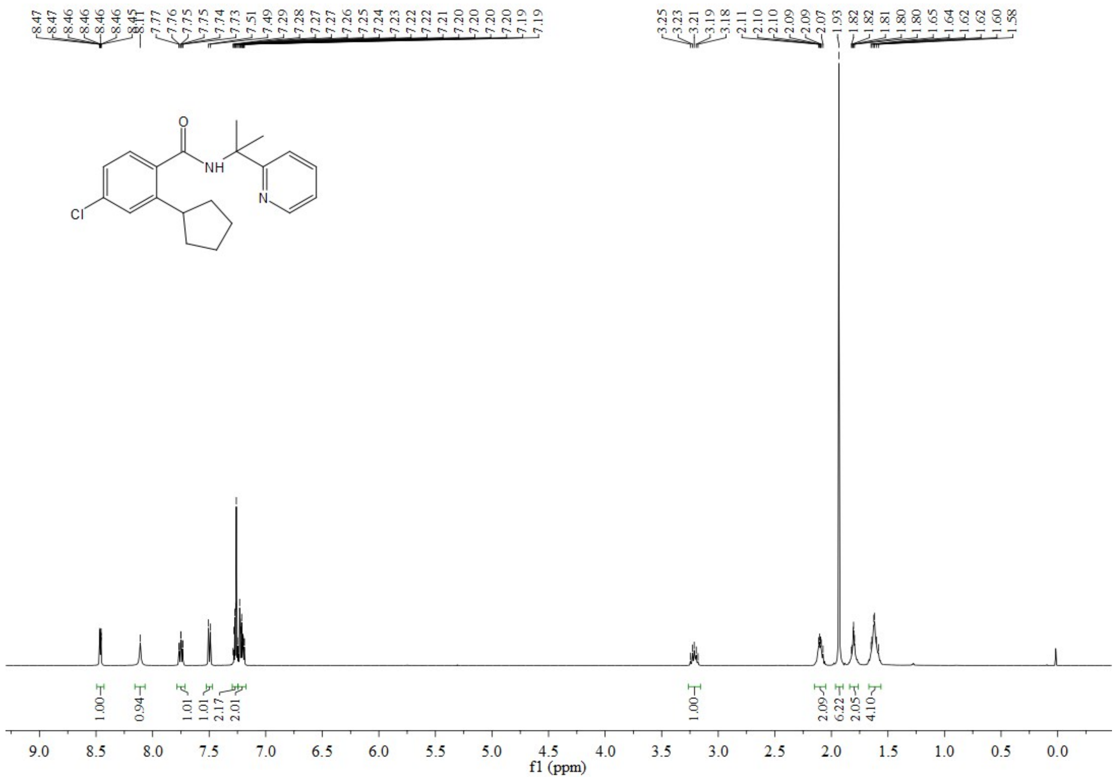

^{1}H NMR spectrum of compound **3la**

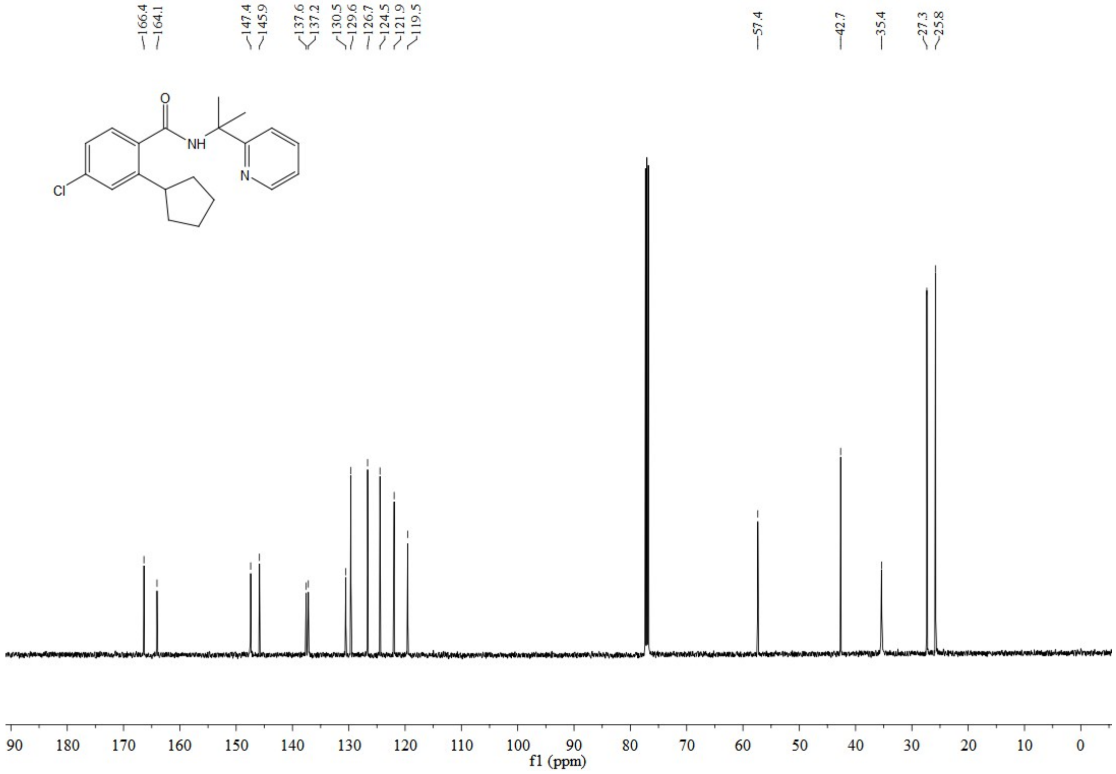

^{13}C NMR spectrum of compound **3la**

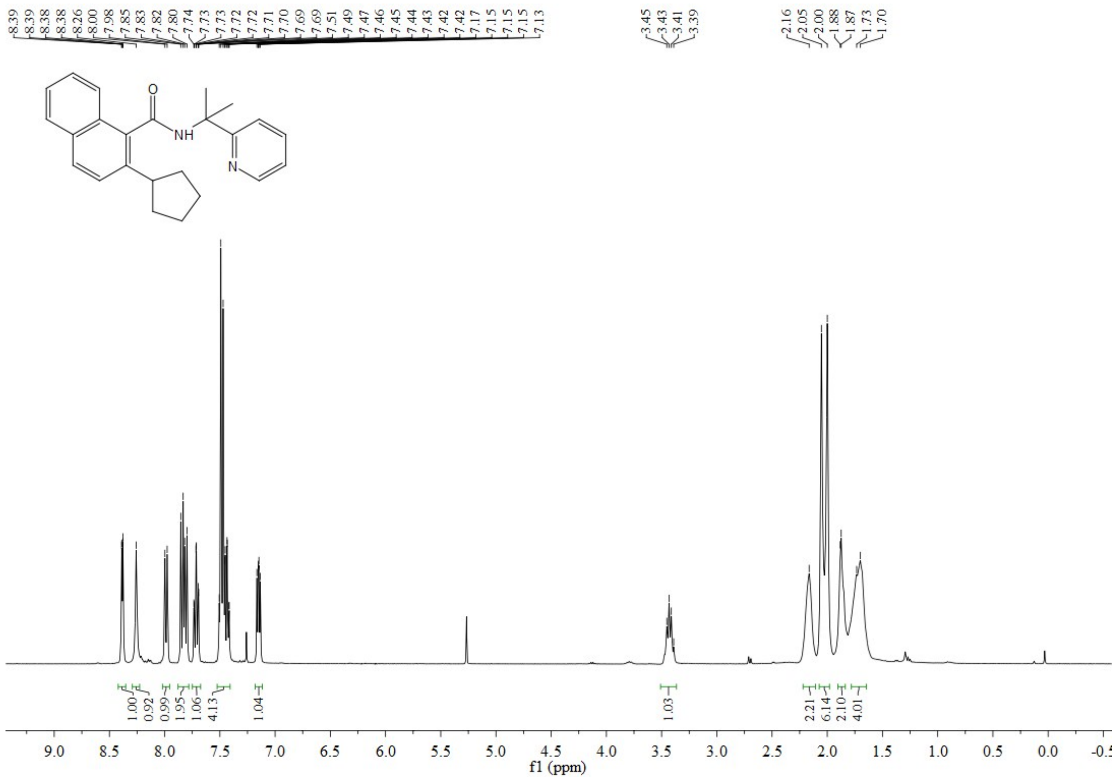

^{1}H NMR spectrum of compound **3ma**

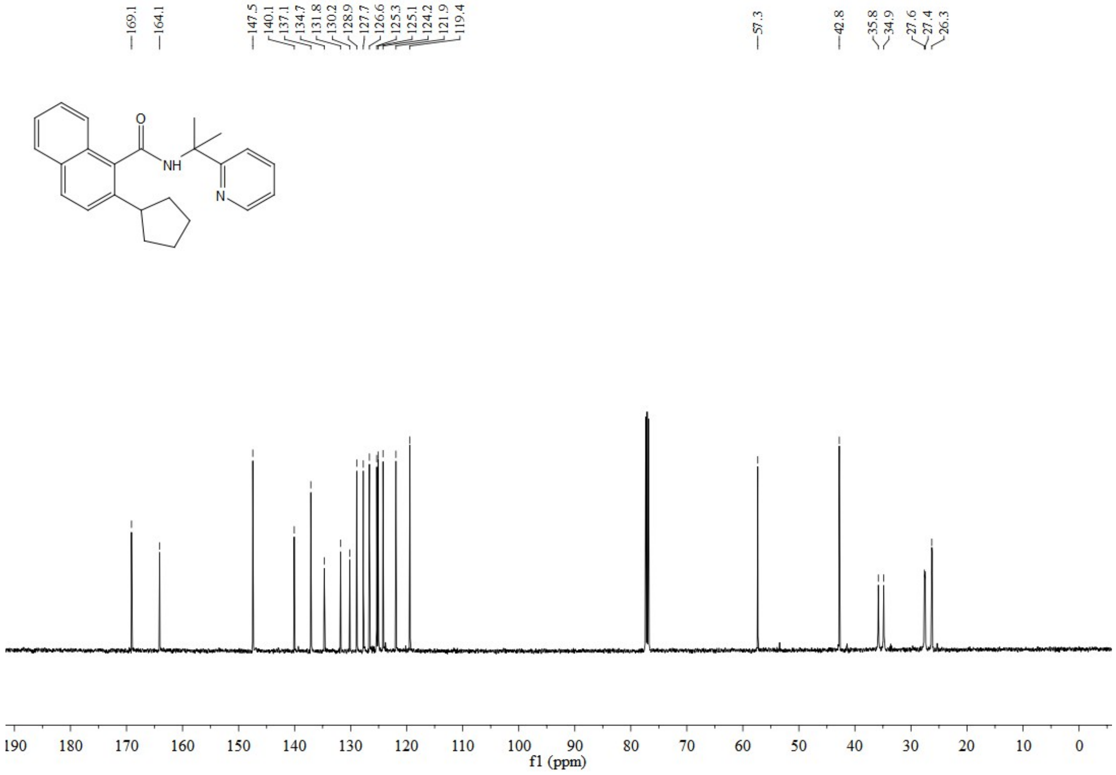

^{13}C NMR spectrum of compound **3ma**

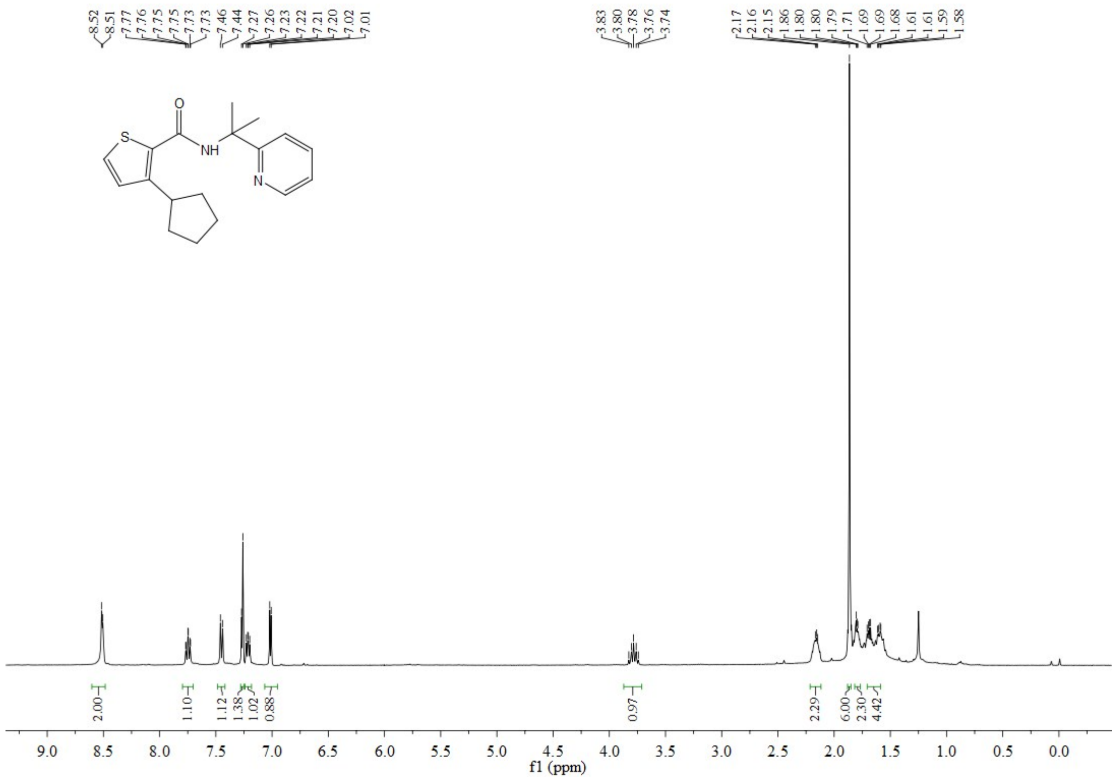

^{1}H NMR spectrum of compound **3na**

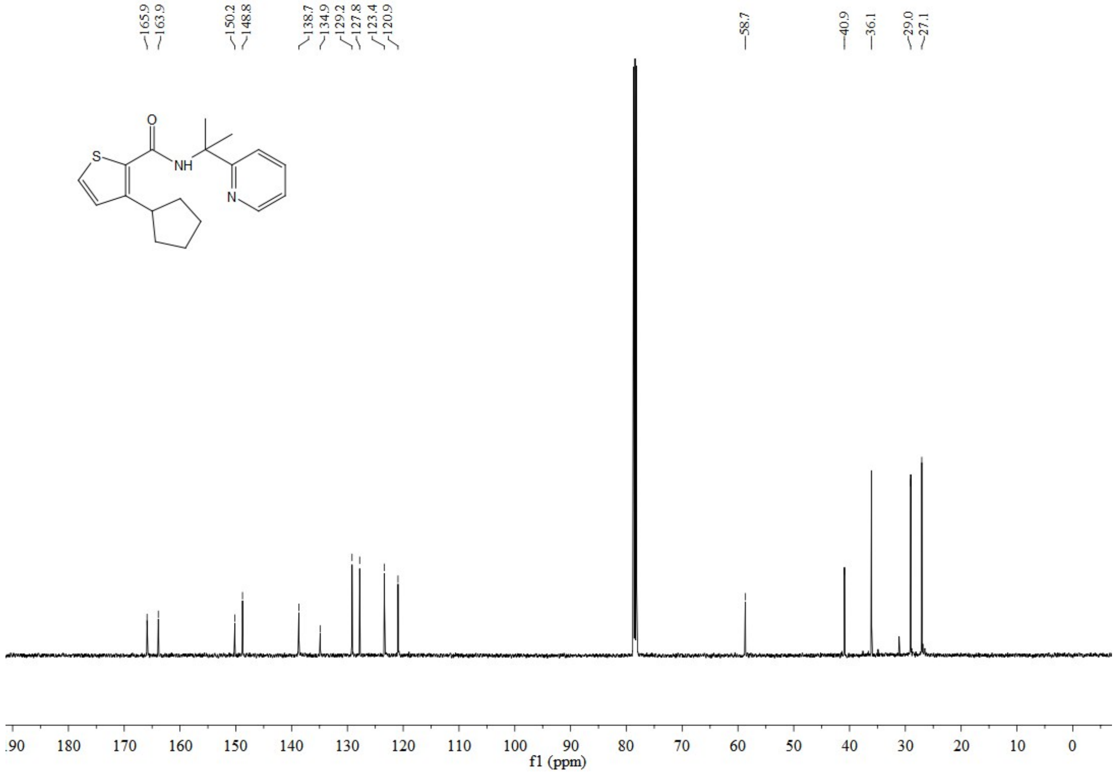

^{13}C NMR spectrum of compound **3na**

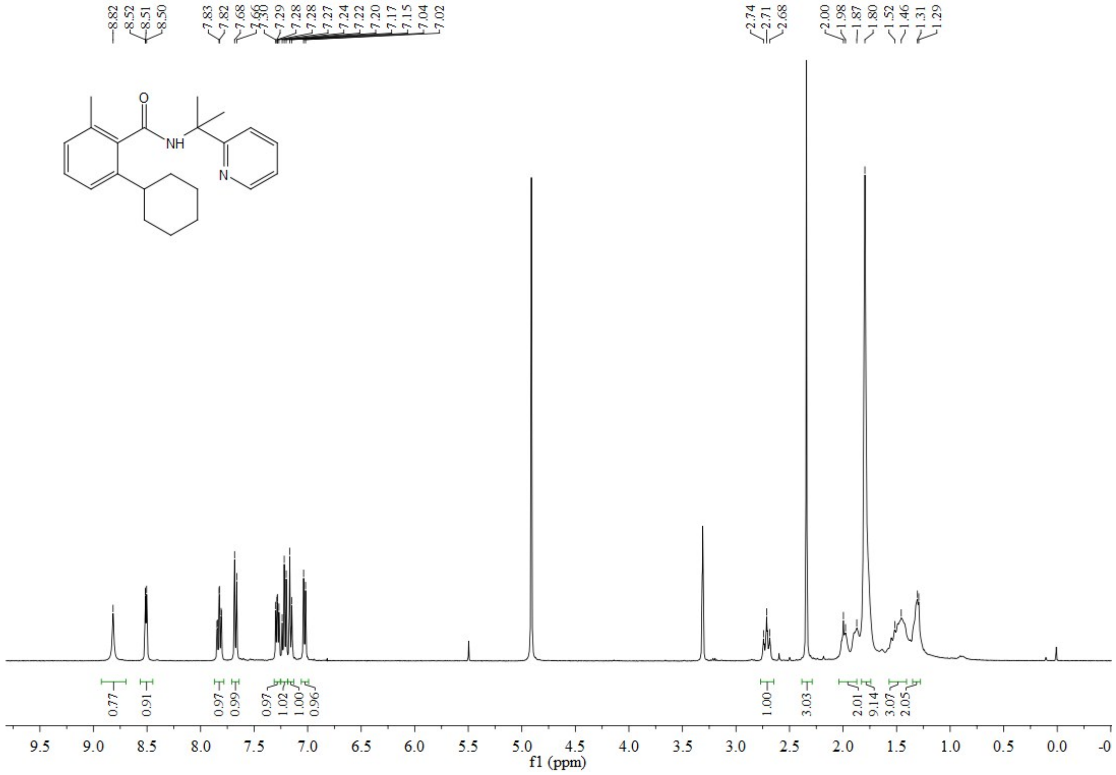

^{1}H NMR spectrum of compound **3ab**

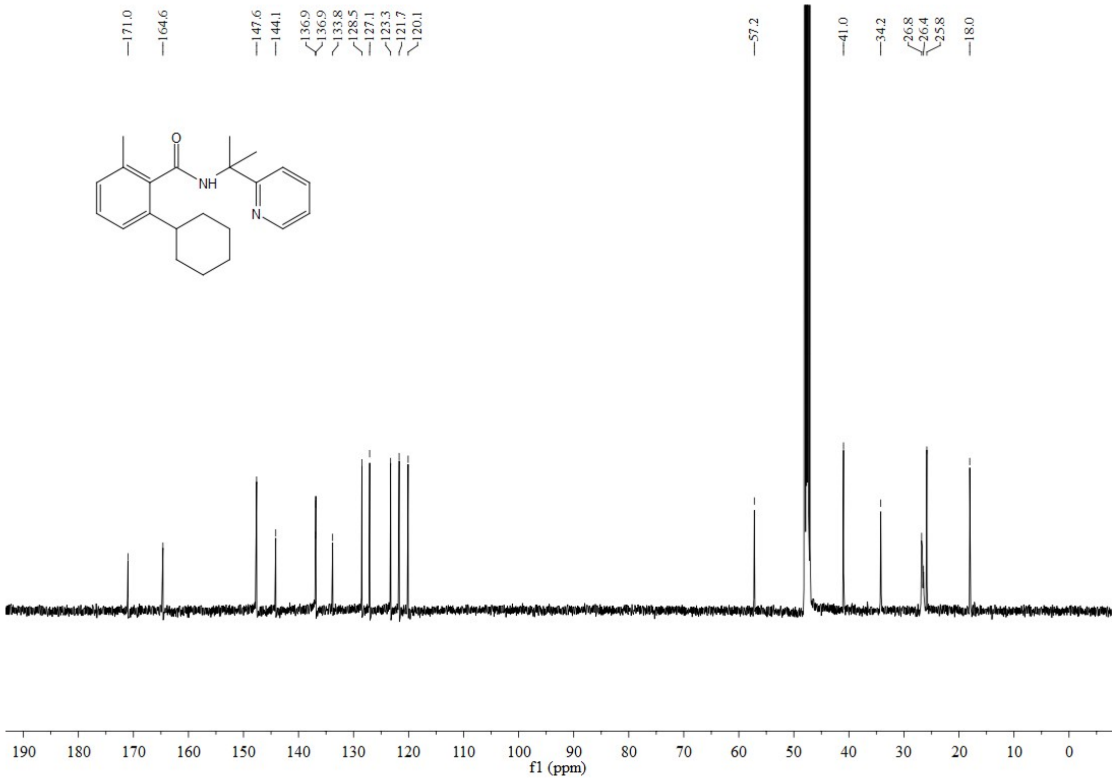

^{13}C NMR spectrum of compound **3ab**

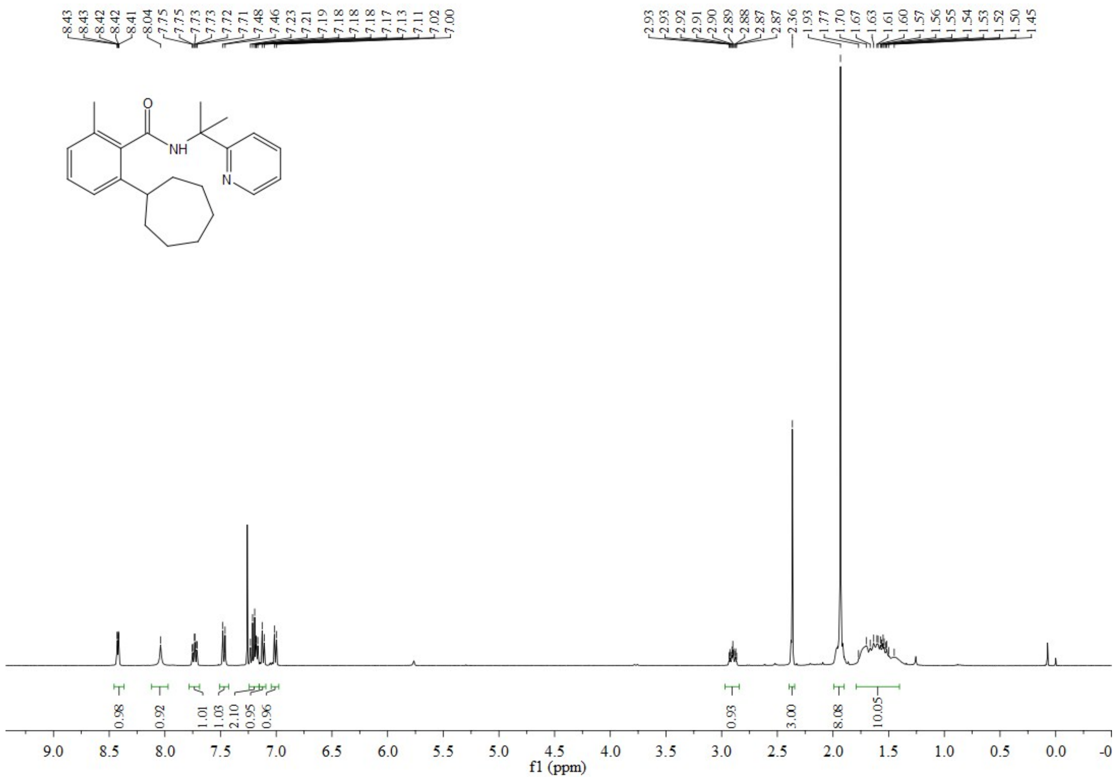

^{1}H NMR spectrum of compound **3ac**

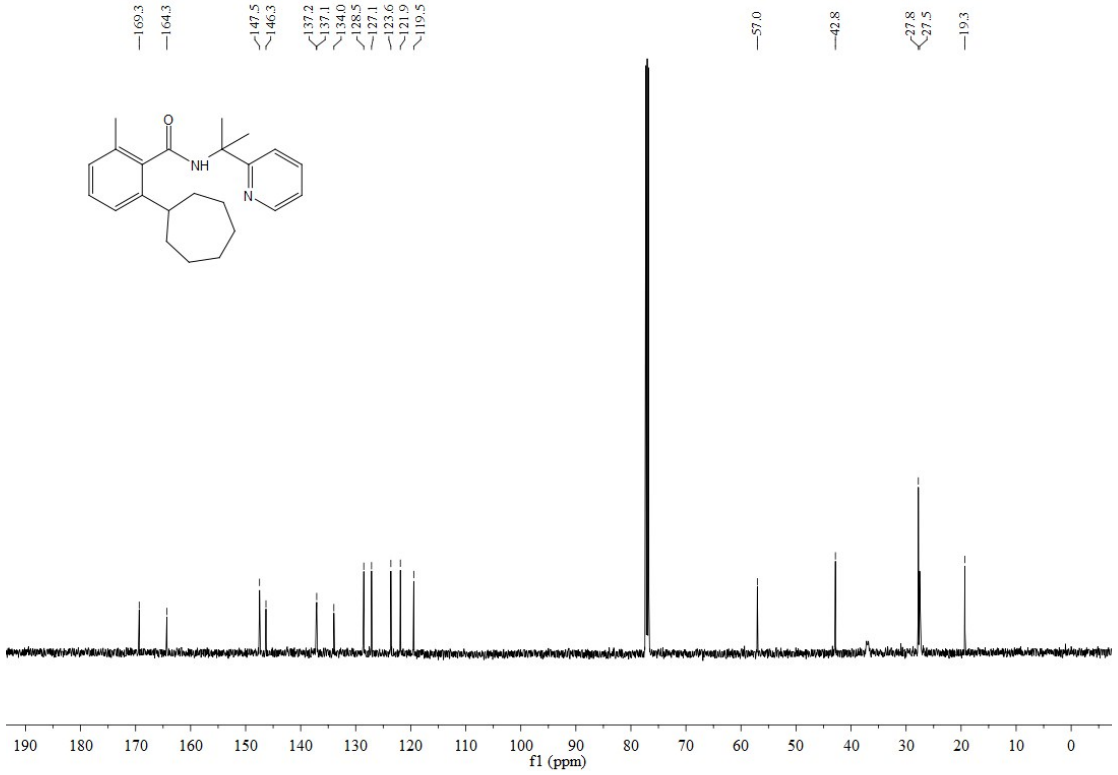

^{13}C NMR spectrum of compound **3ac**

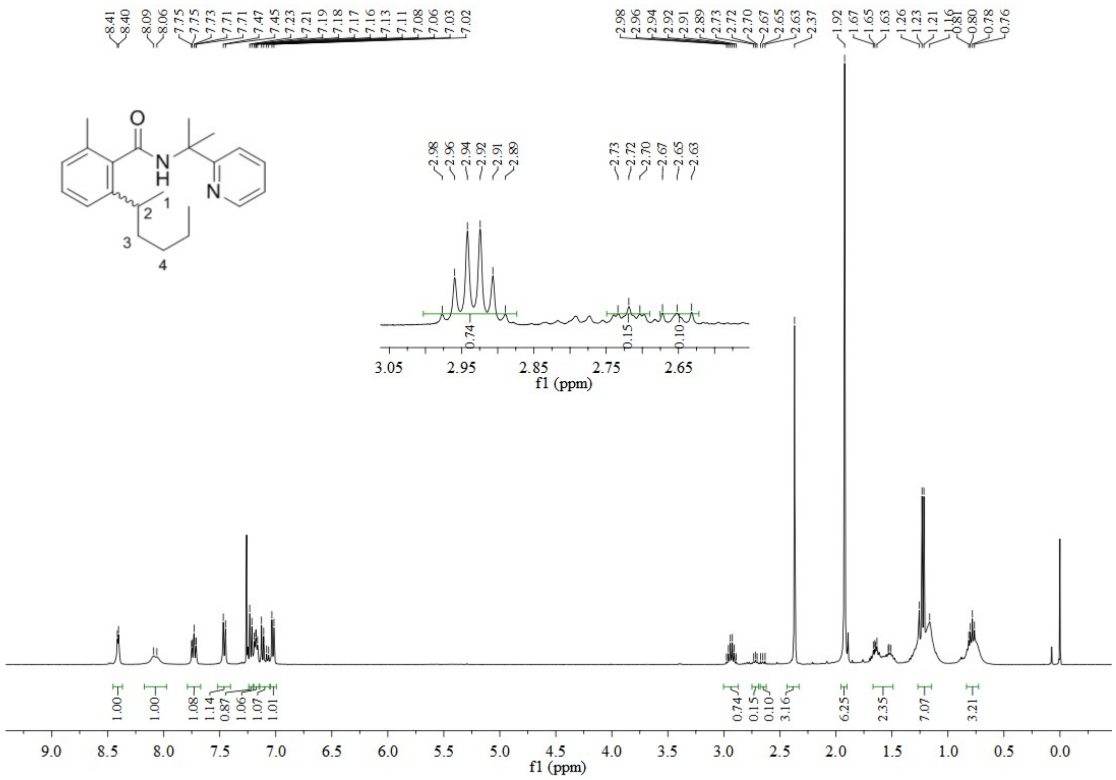

^{1}H NMR spectrum of compound **3ad**

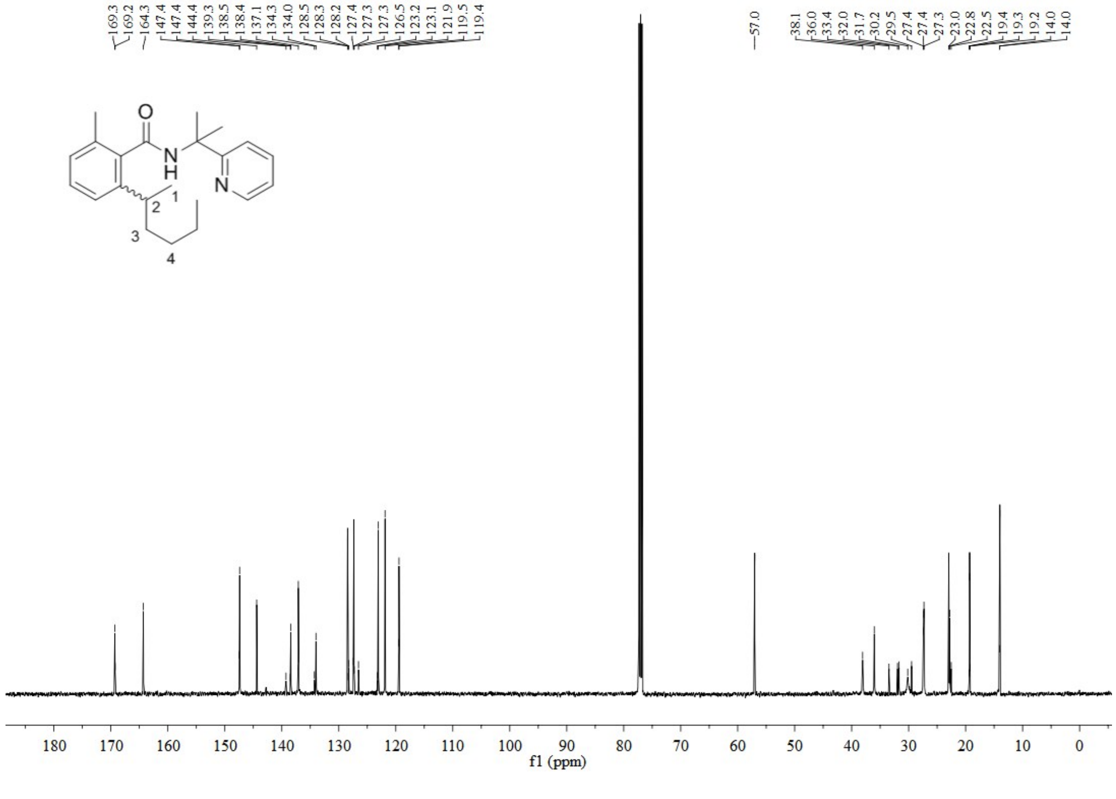

^{13}C NMR spectrum of compound **3ad**

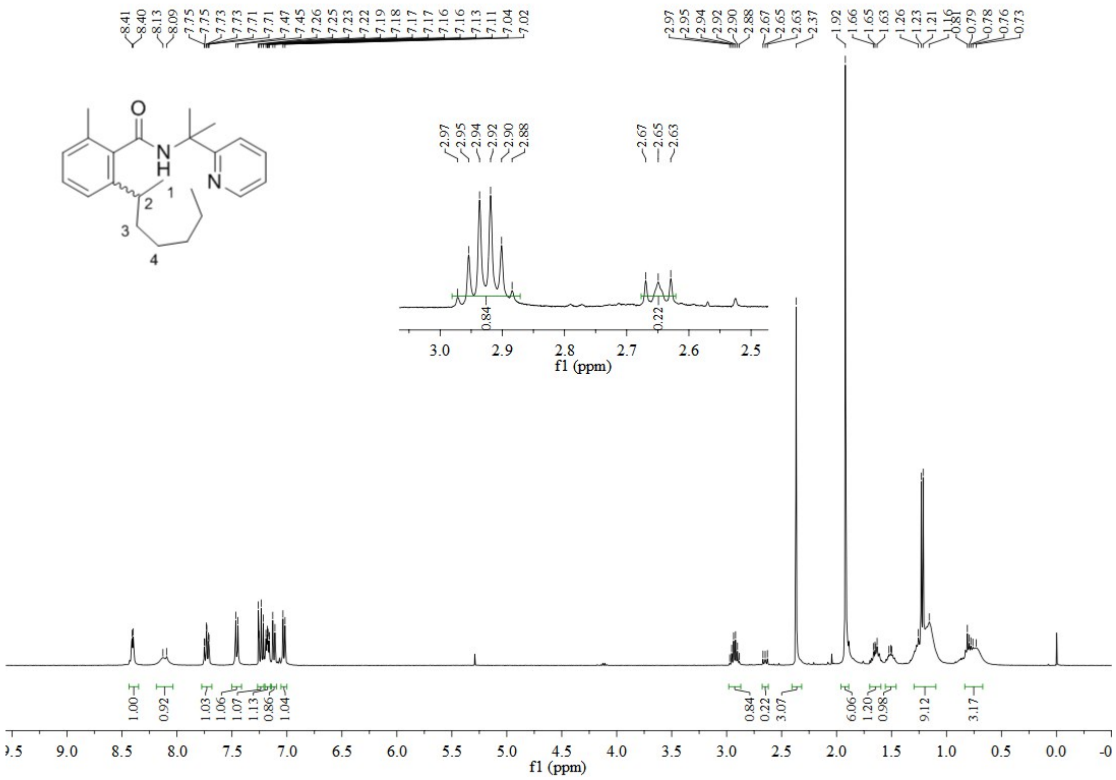

^{1}H NMR spectrum of compound **3ae**

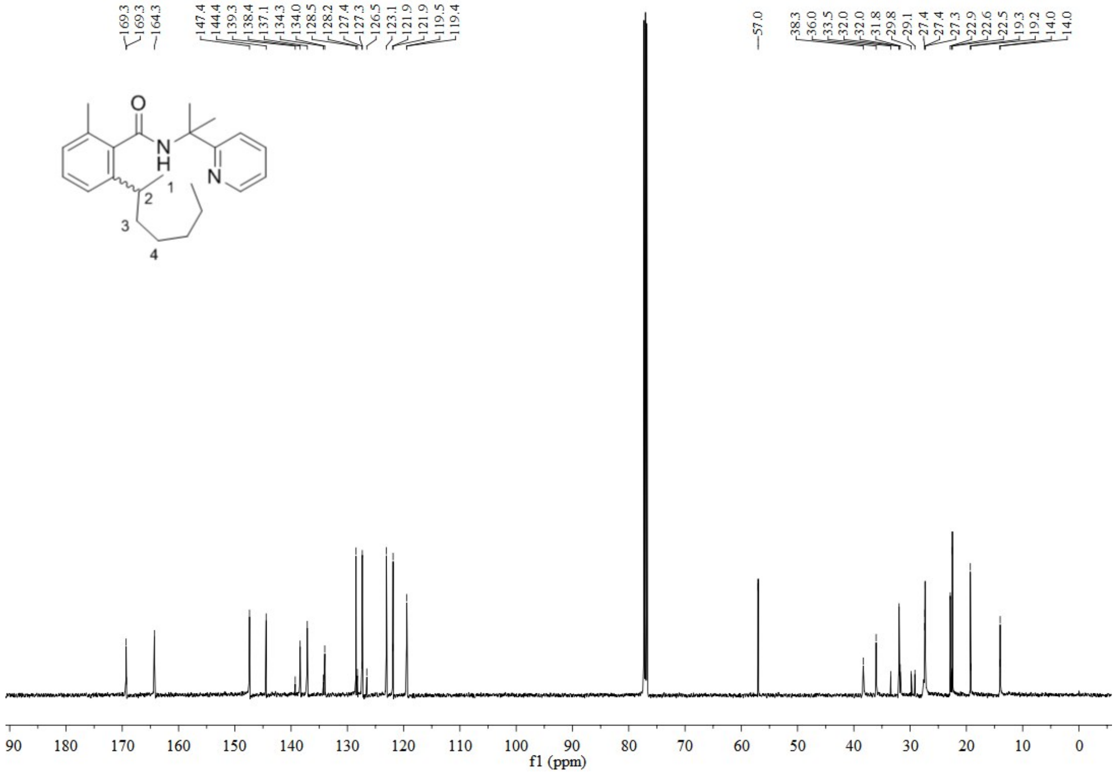

^{13}C NMR spectrum of compound **3ae**

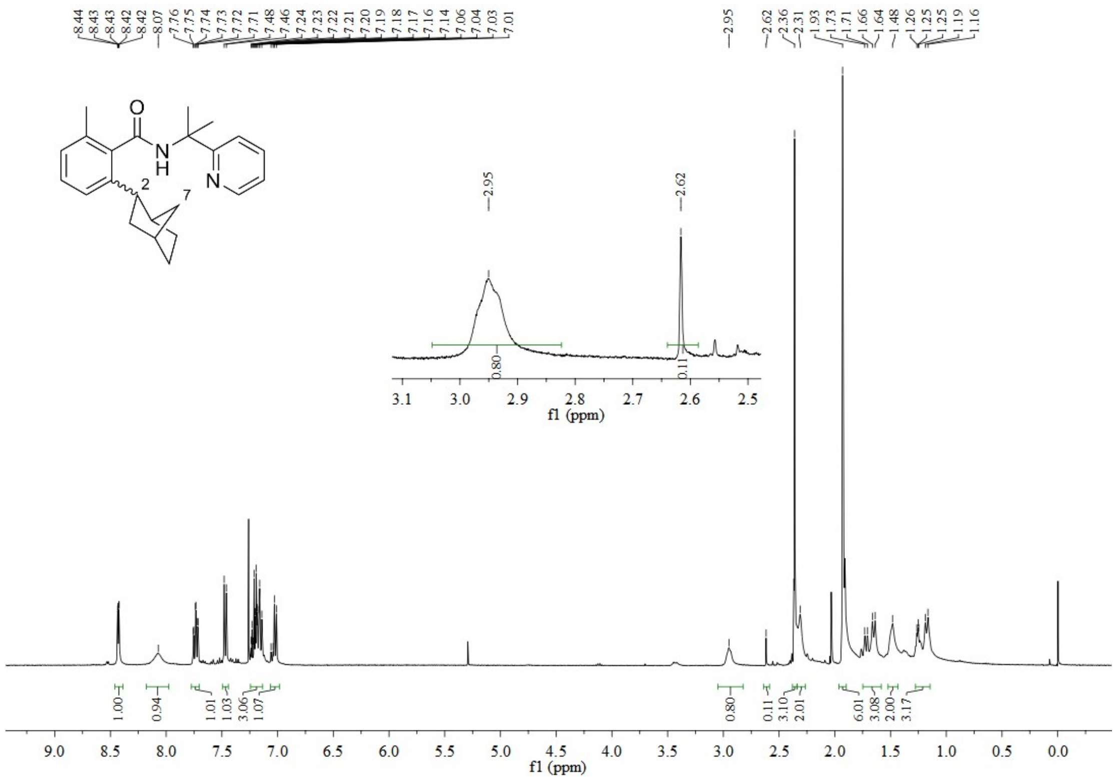

^{1}H NMR spectrum of compound **3af**

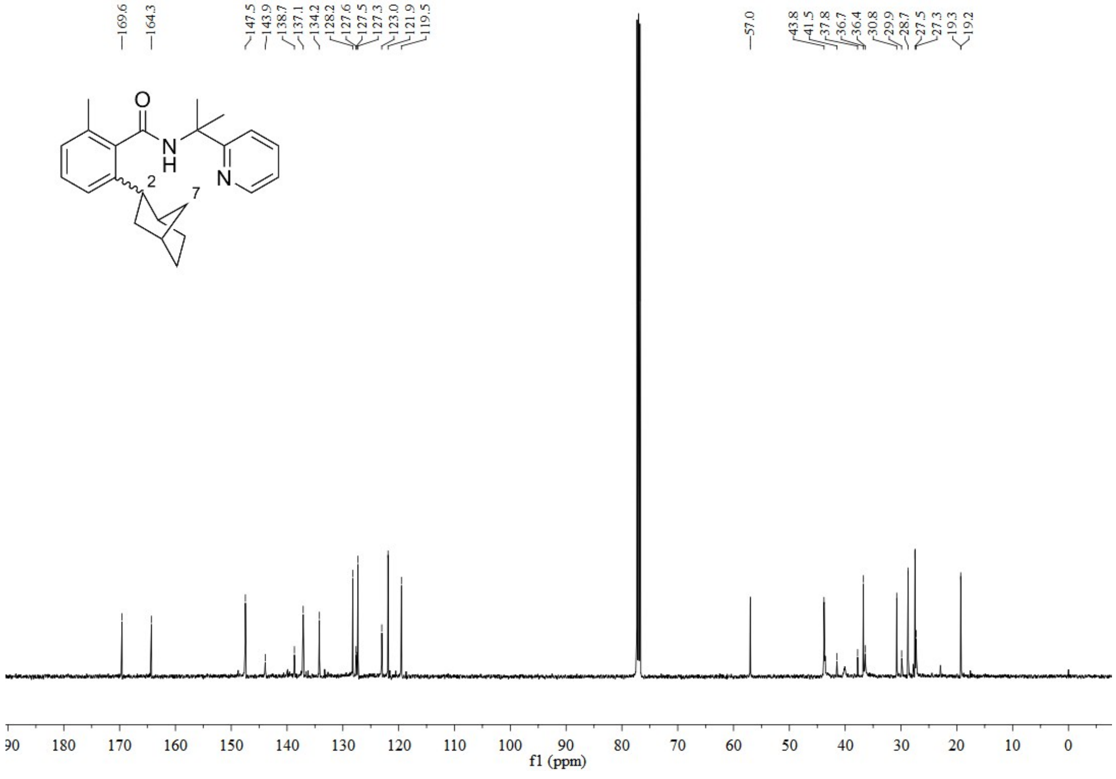

^{13}C NMR spectrum of compound **3af**

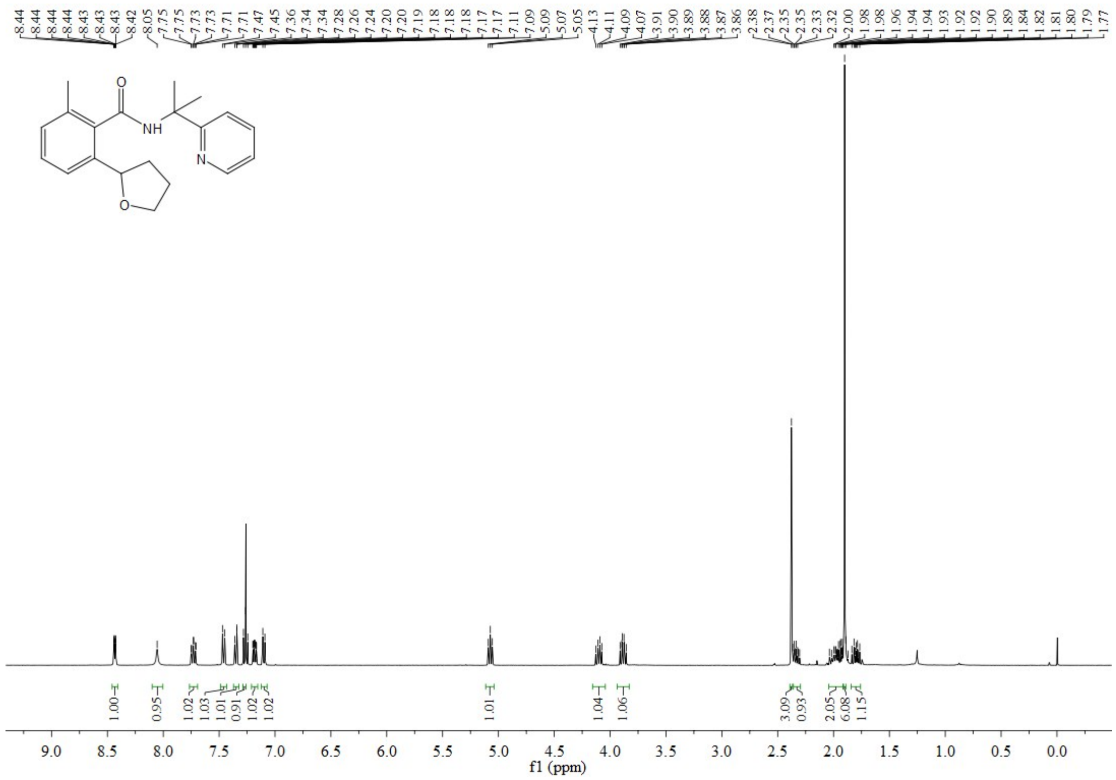

^{1}H NMR spectrum of compound **3ag**

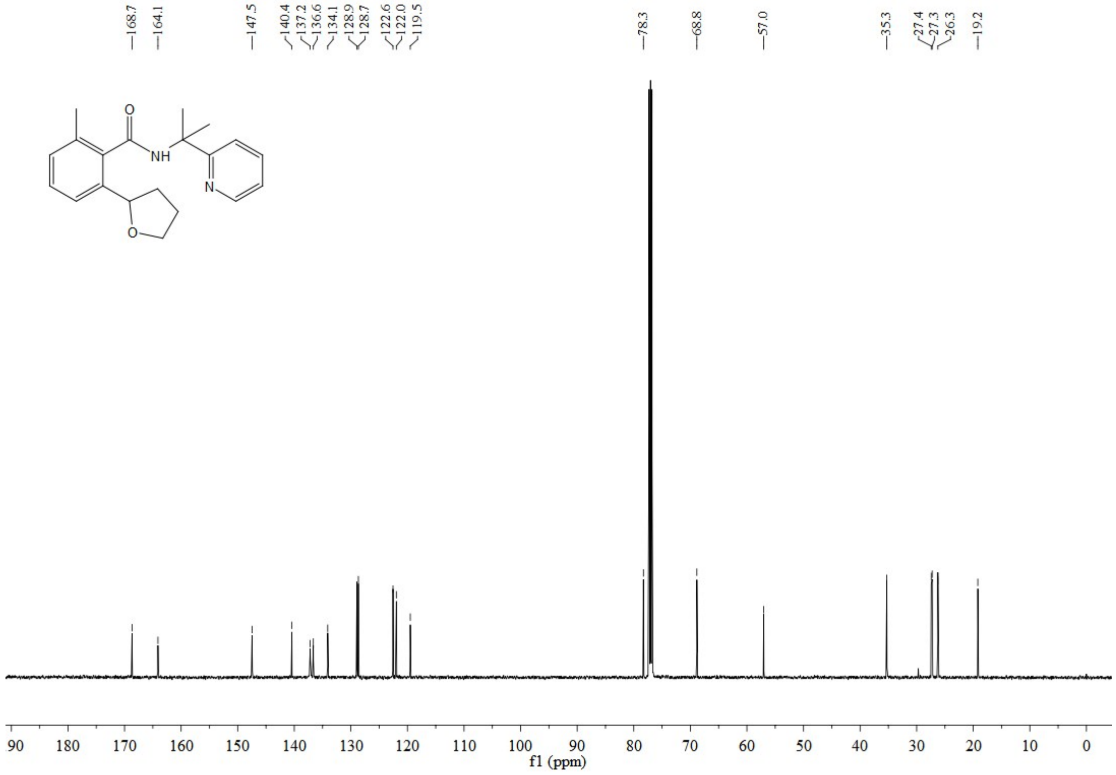

^{13}C NMR spectrum of compound **3ag**

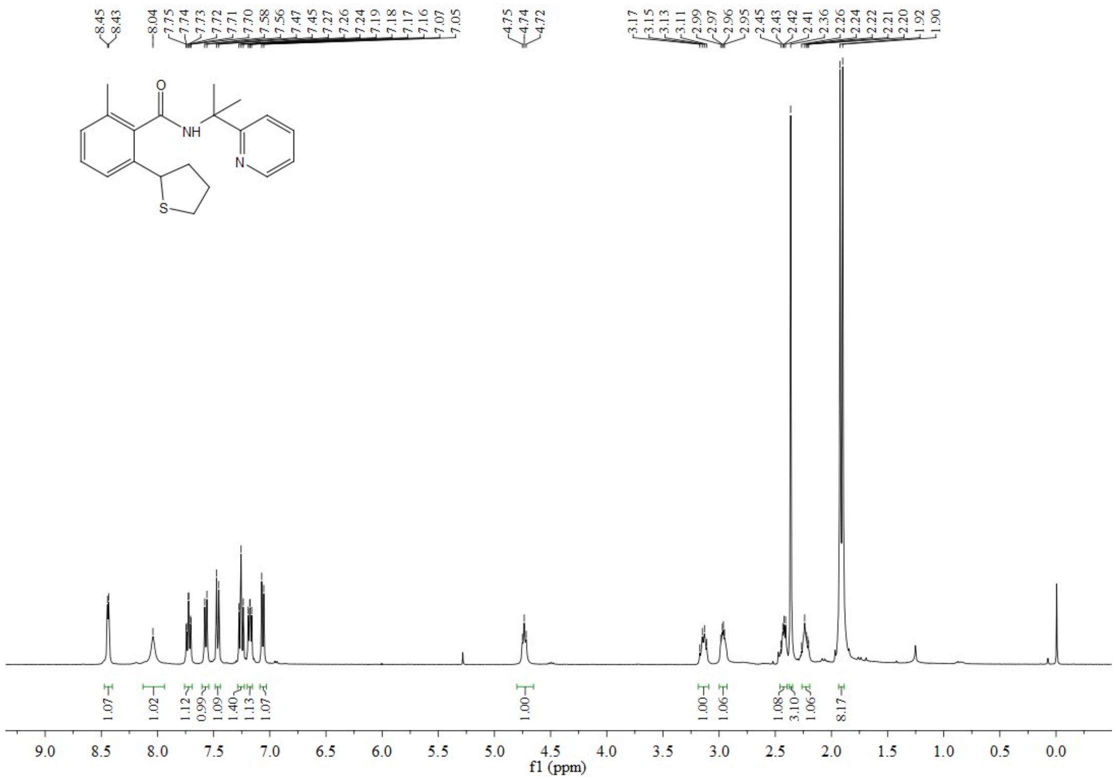

^{1}H NMR spectrum of compound **3ah**

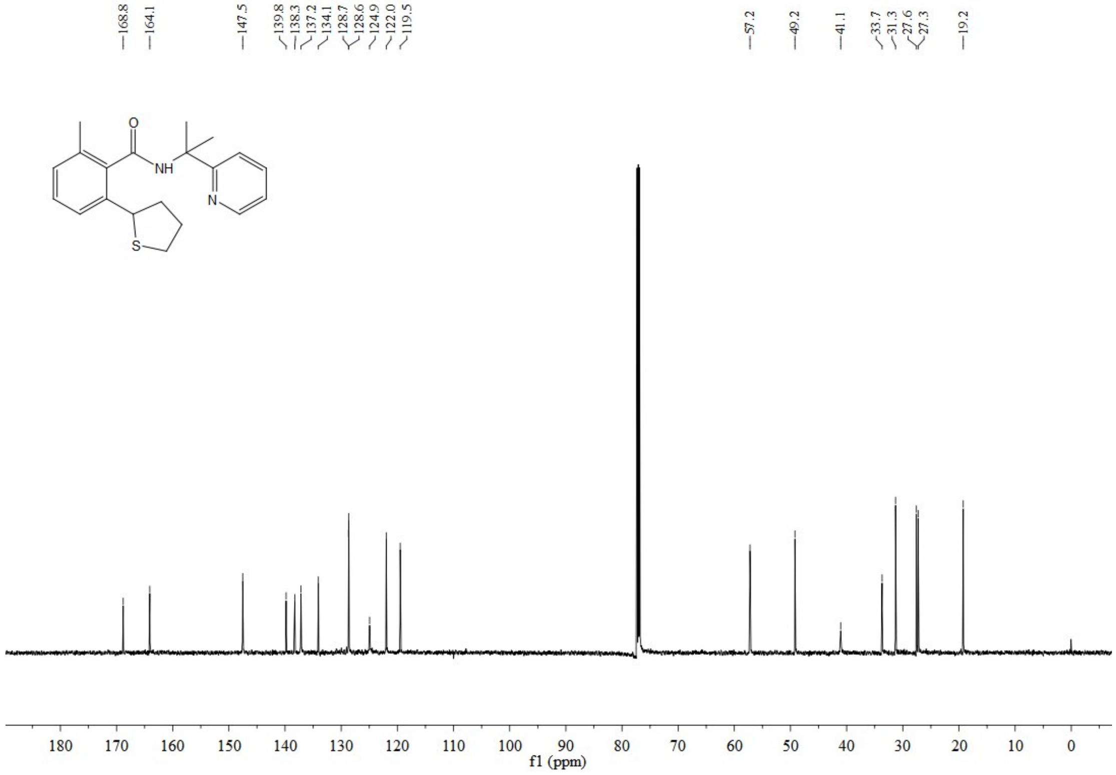

^{13}C NMR spectrum of compound **3ah**

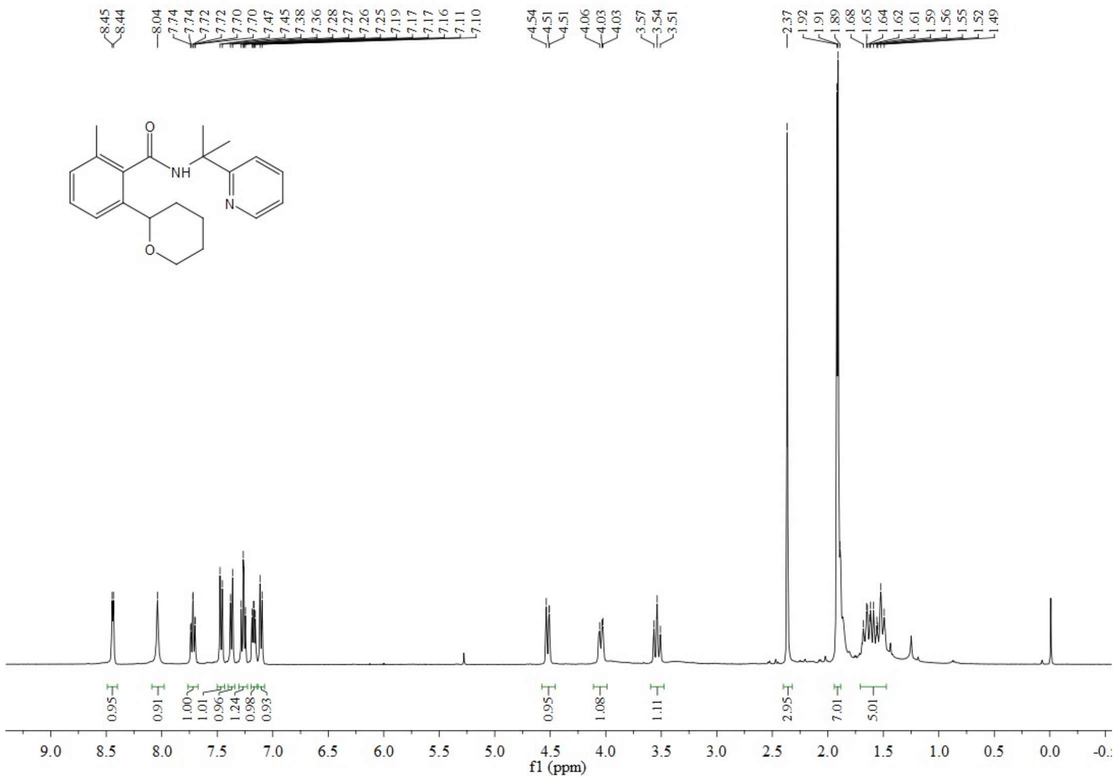

^{1}H NMR spectrum of compound **3ai**

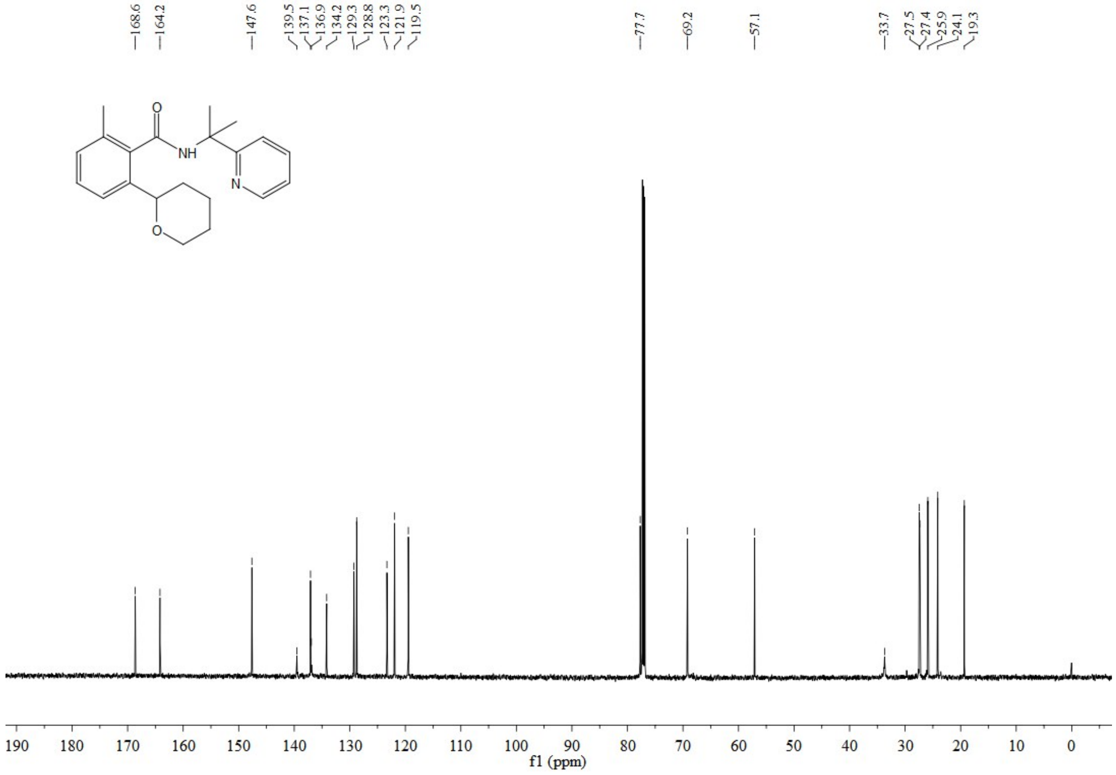

^{13}C NMR spectrum of compound **3ai**

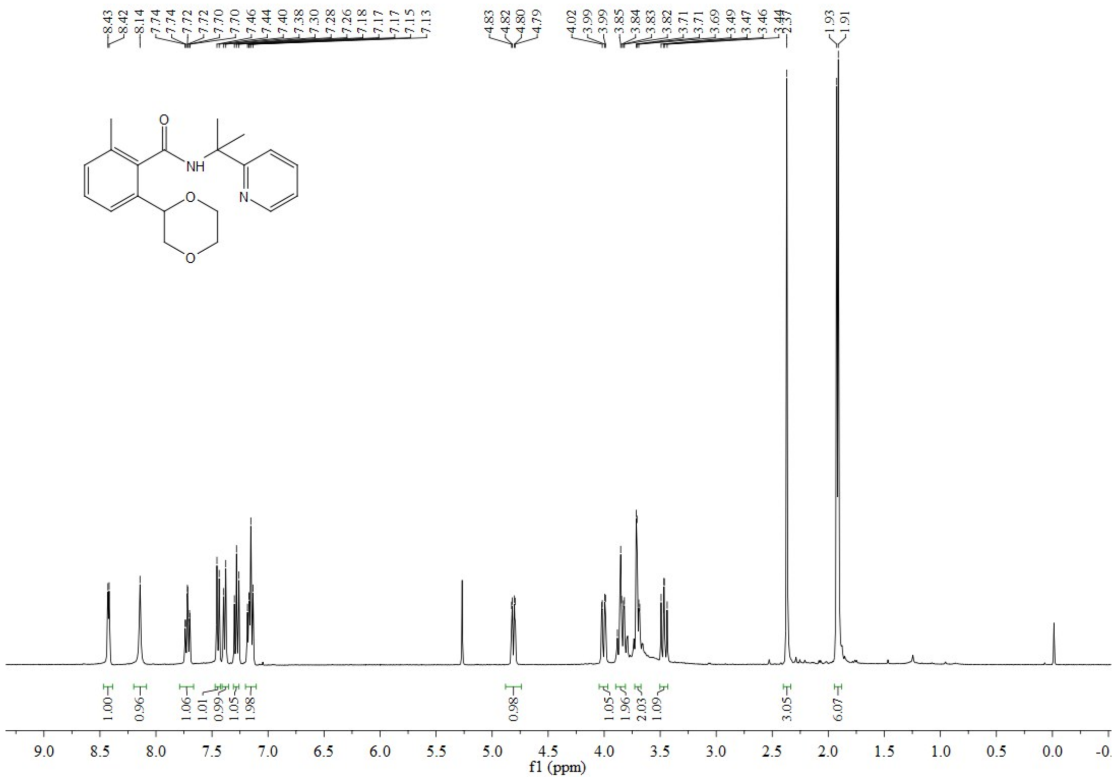

^{1}H NMR spectrum of compound **3aj**

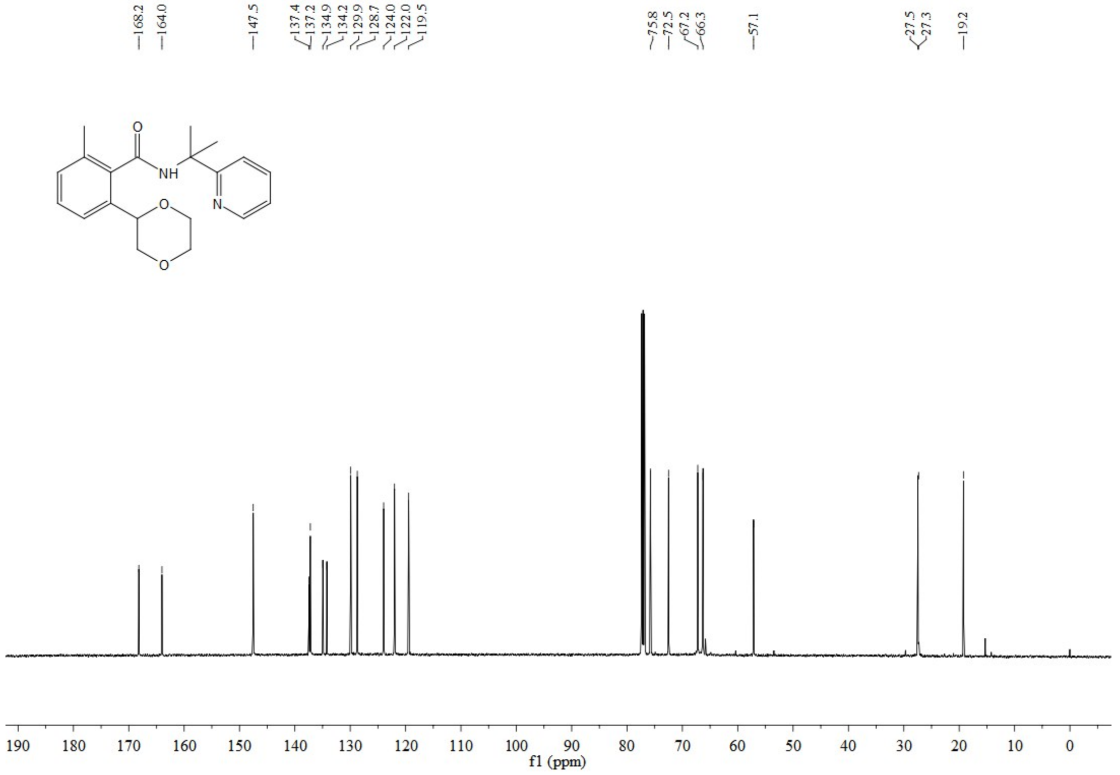

^{13}C NMR spectrum of compound **3aj**

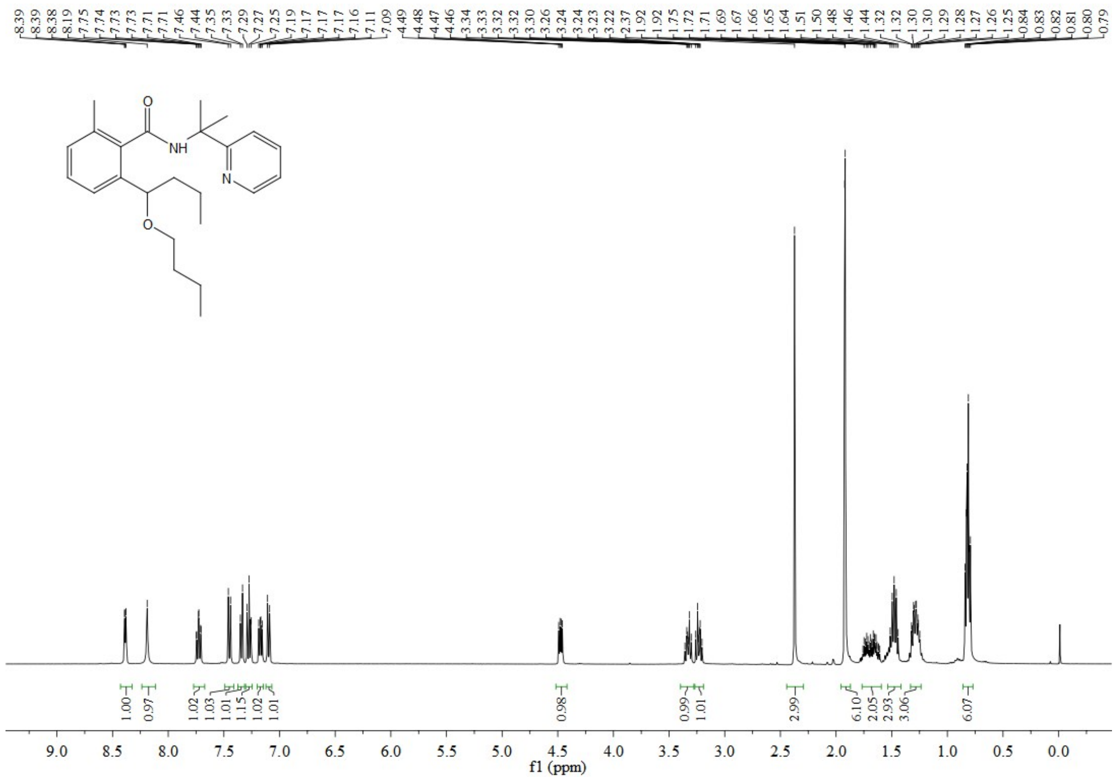

^{1}H NMR spectrum of compound **3ak**

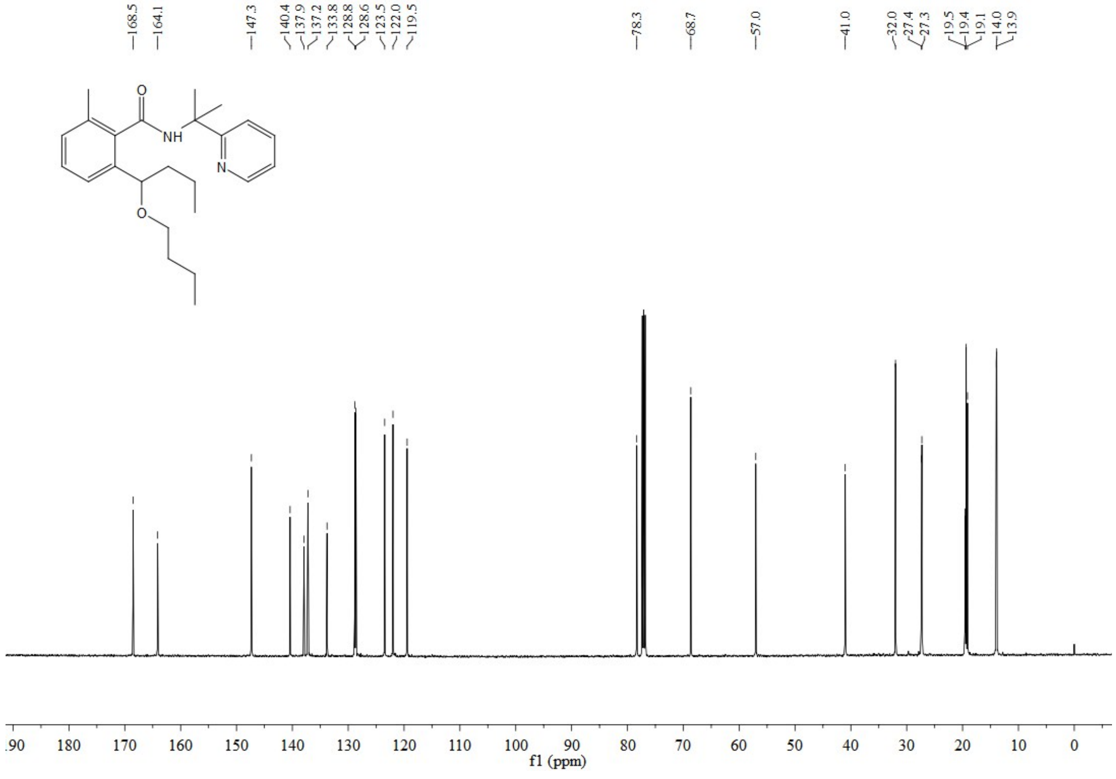

^{13}C NMR spectrum of compound **3ak**

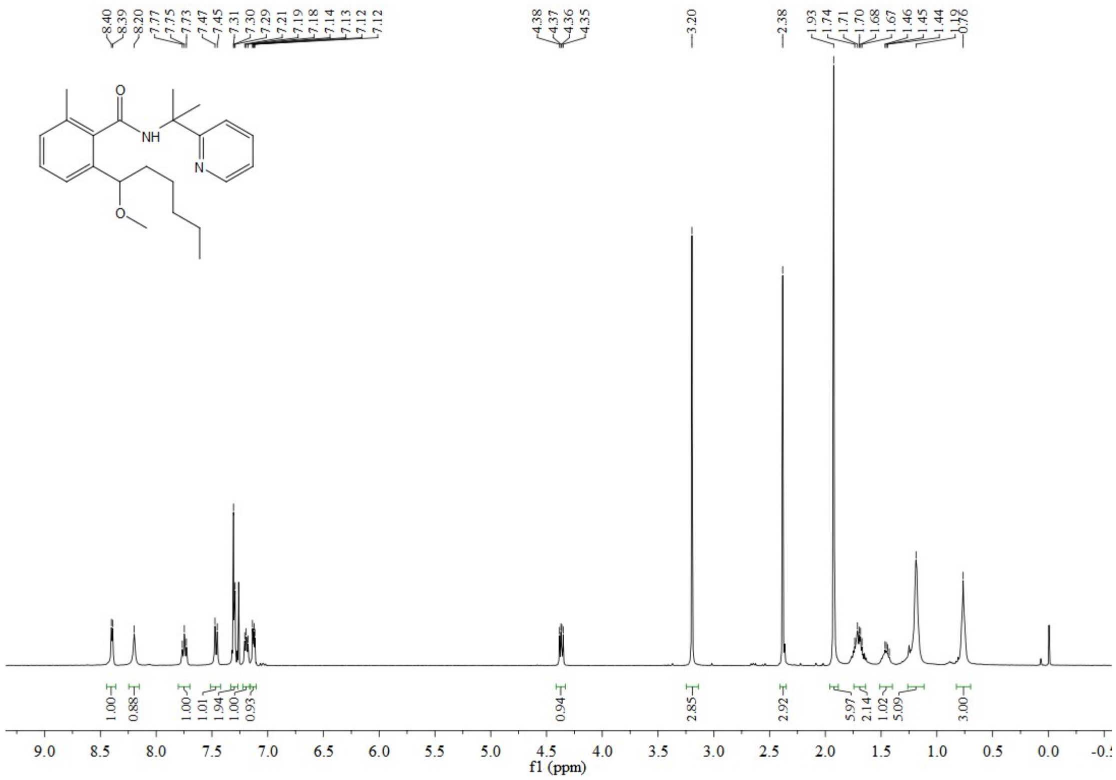

^{1}H NMR spectrum of compound **3al**

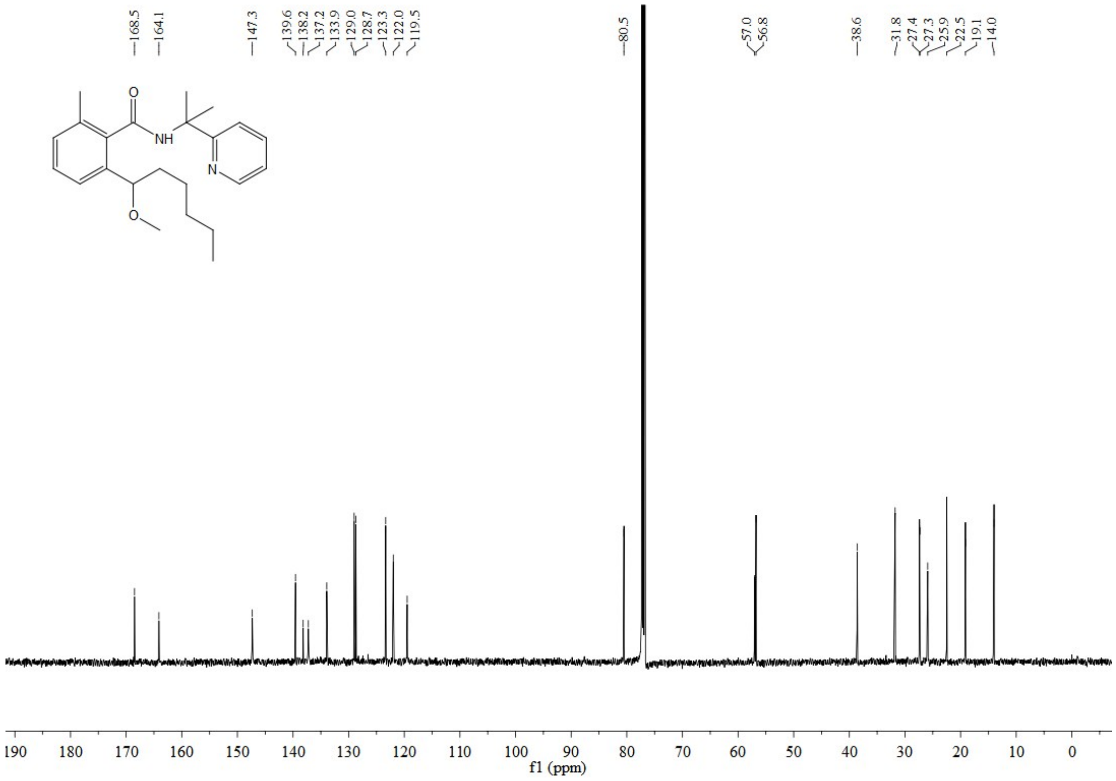

^{13}C NMR spectrum of compound **3al**

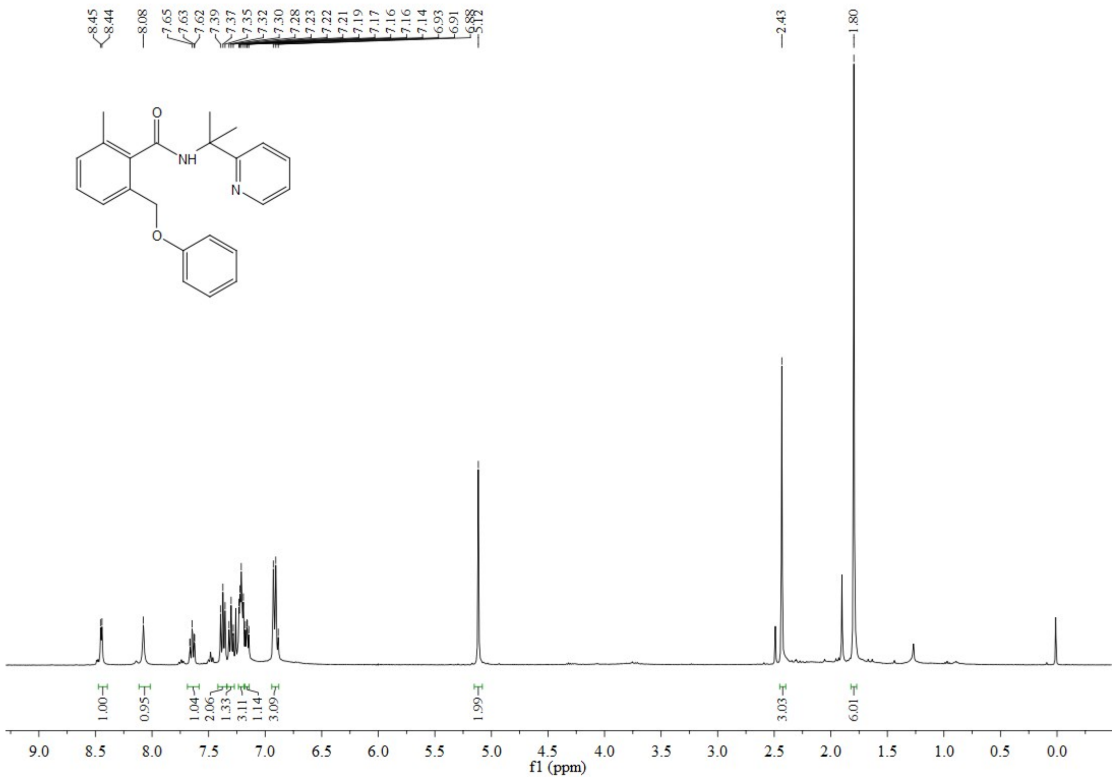

^{1}H NMR spectrum of compound **3am**

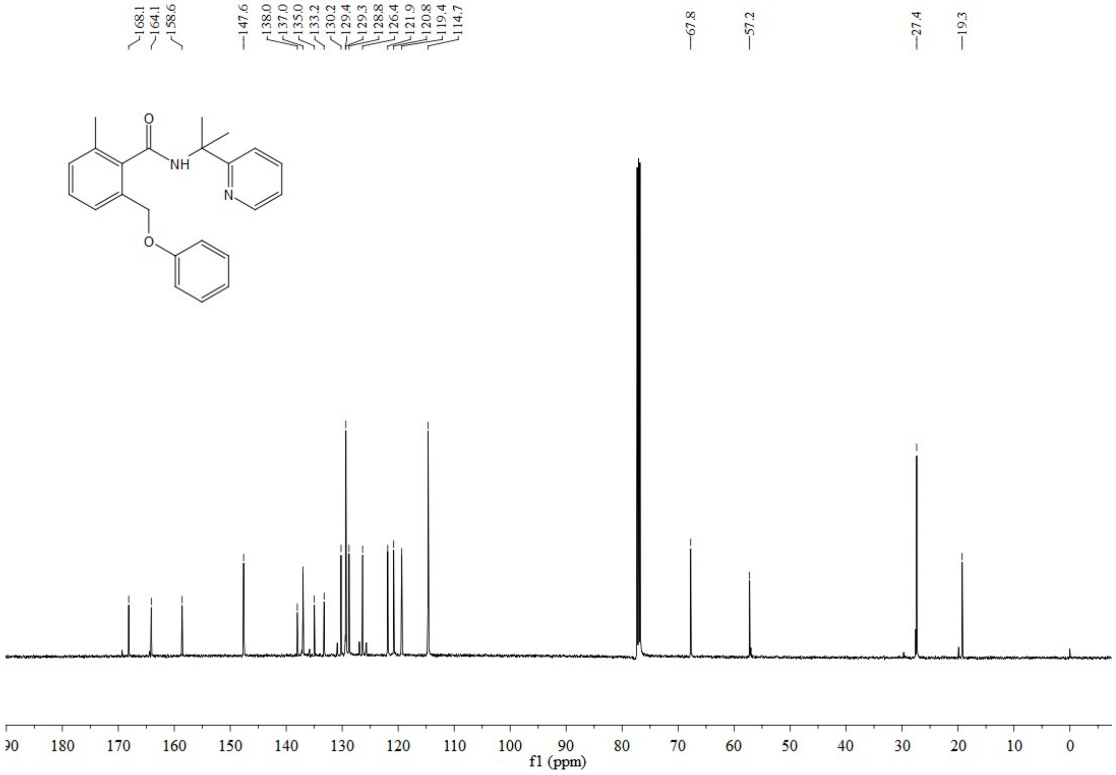

^{13}C NMR spectrum of compound **3am**

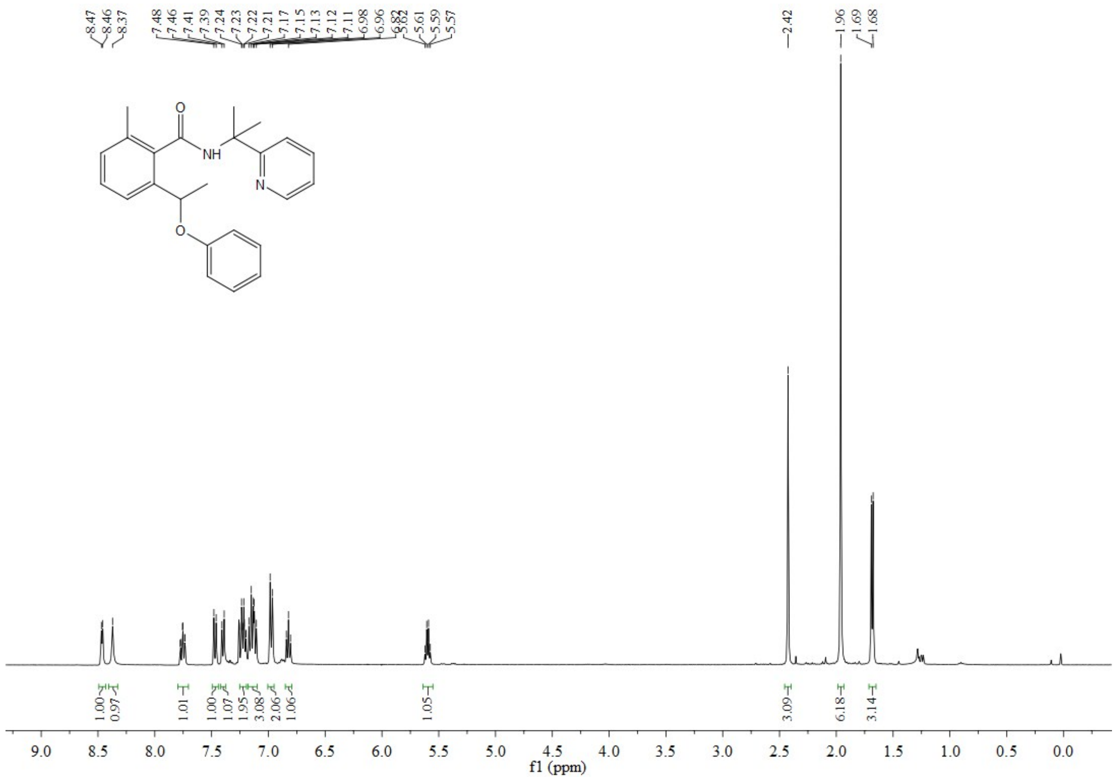

^{1}H NMR spectrum of compound **3an**

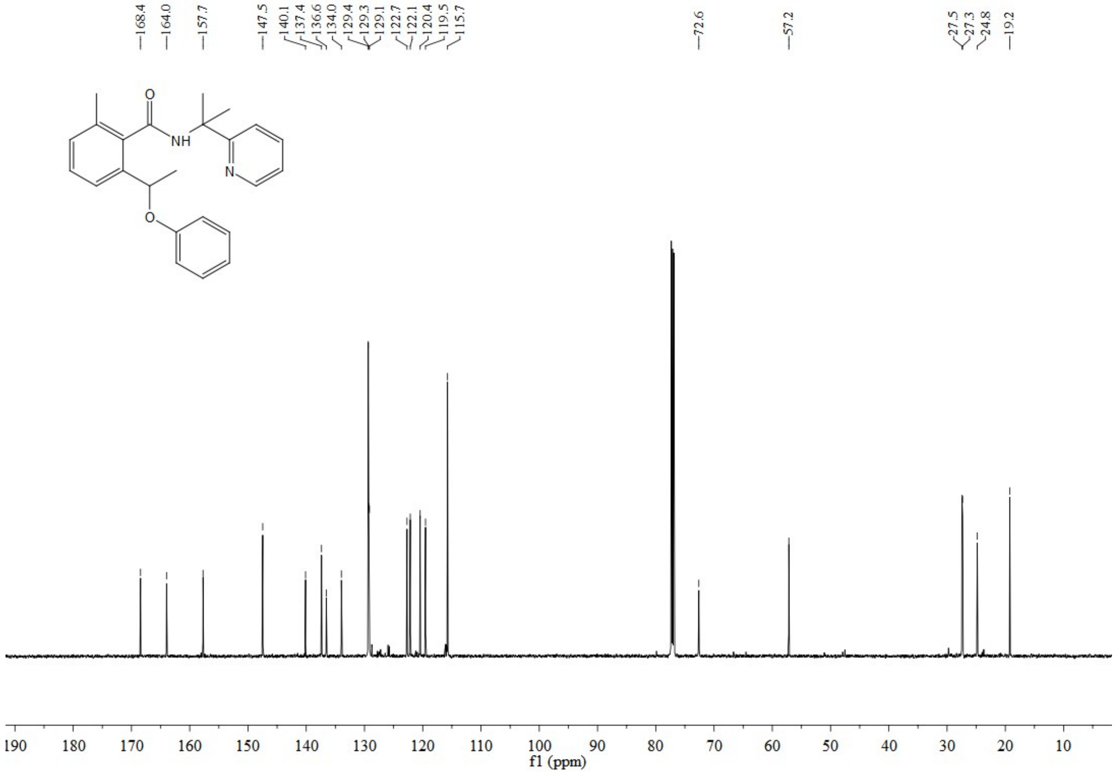

^{13}C NMR spectrum of compound **3an**

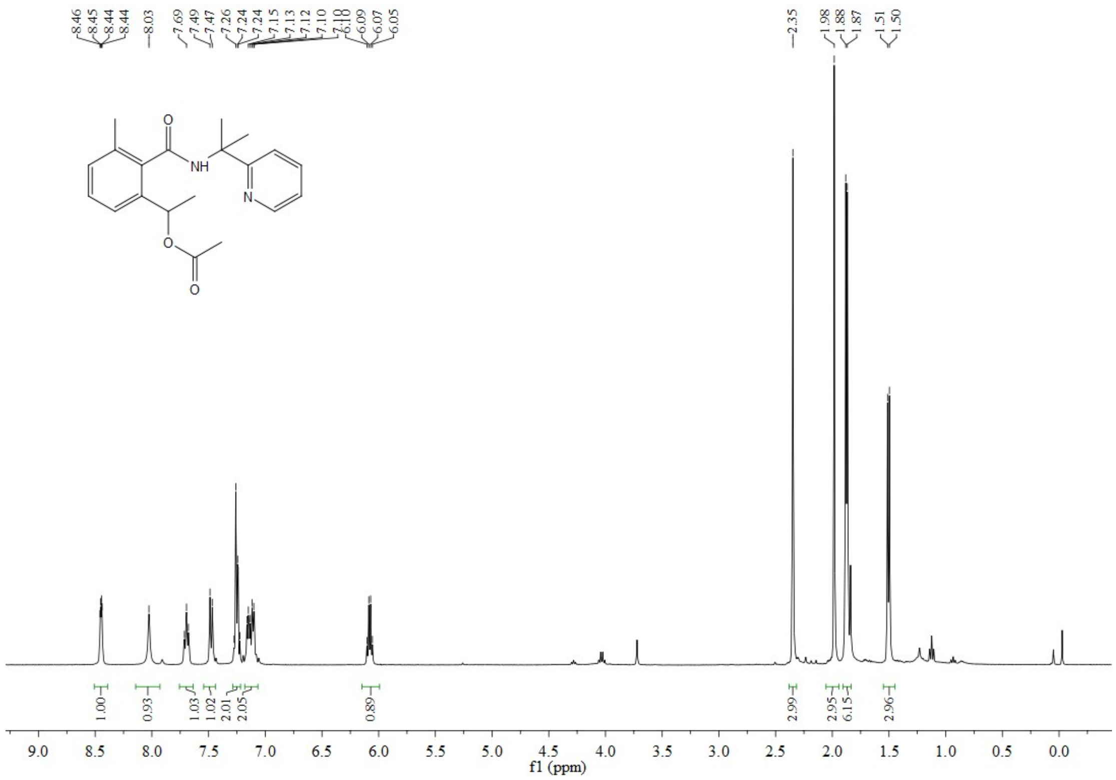

^{1}H NMR spectrum of compound **3ao**

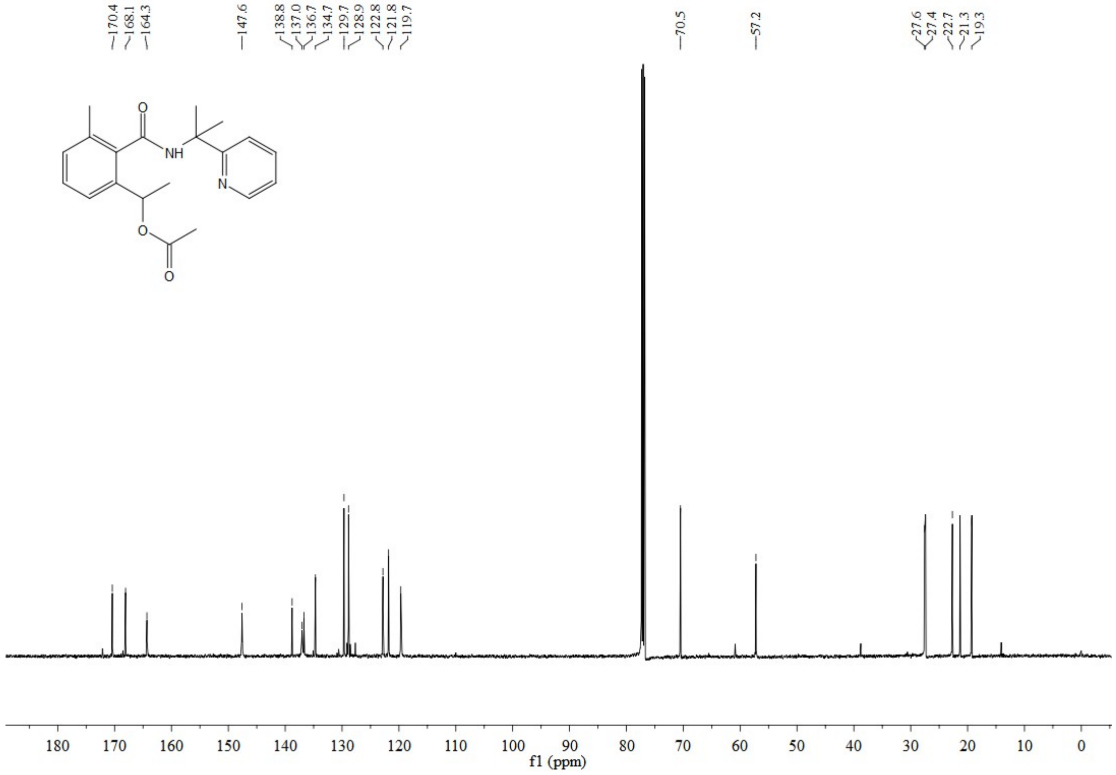

^{13}C NMR spectrum of compound **3ao**

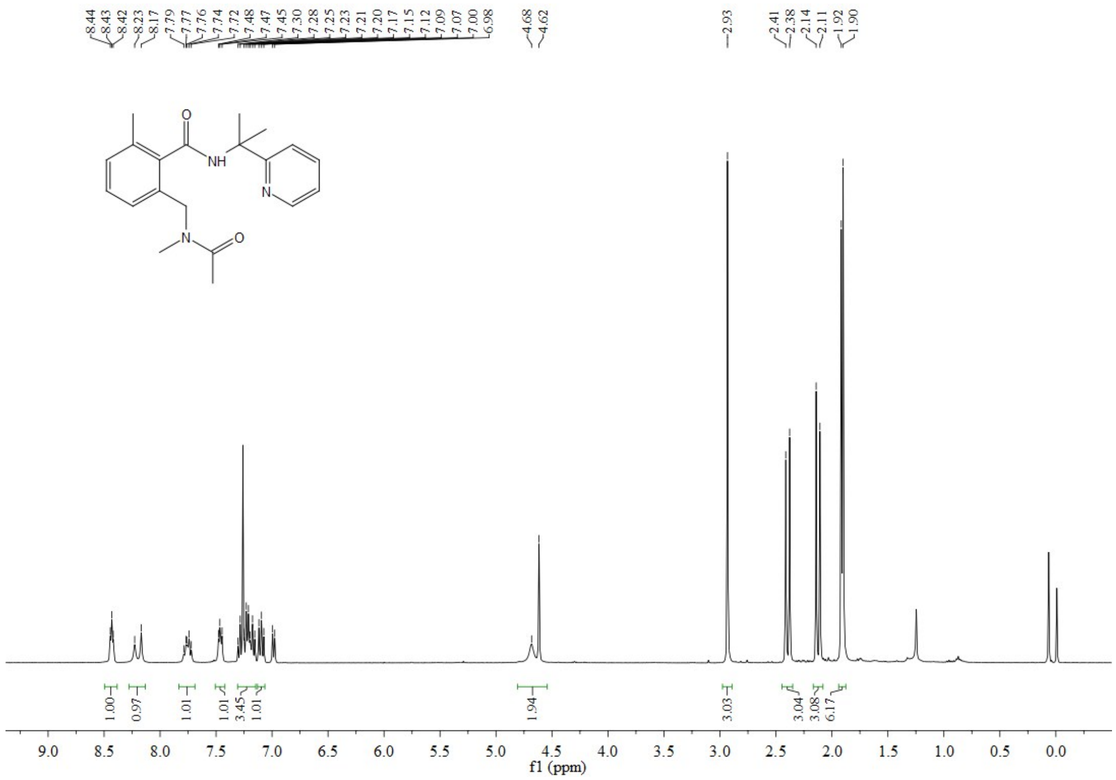

^{1}H NMR spectrum of compound **3ap**

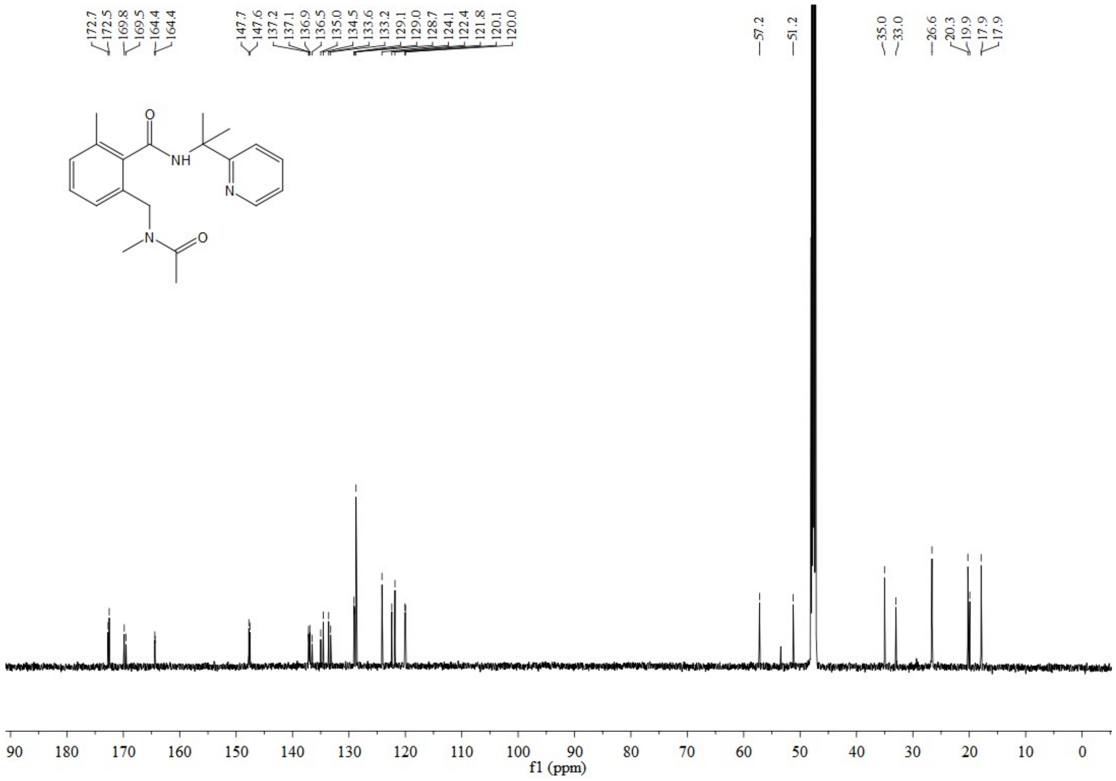

^{13}C NMR spectrum of compound **3ap**

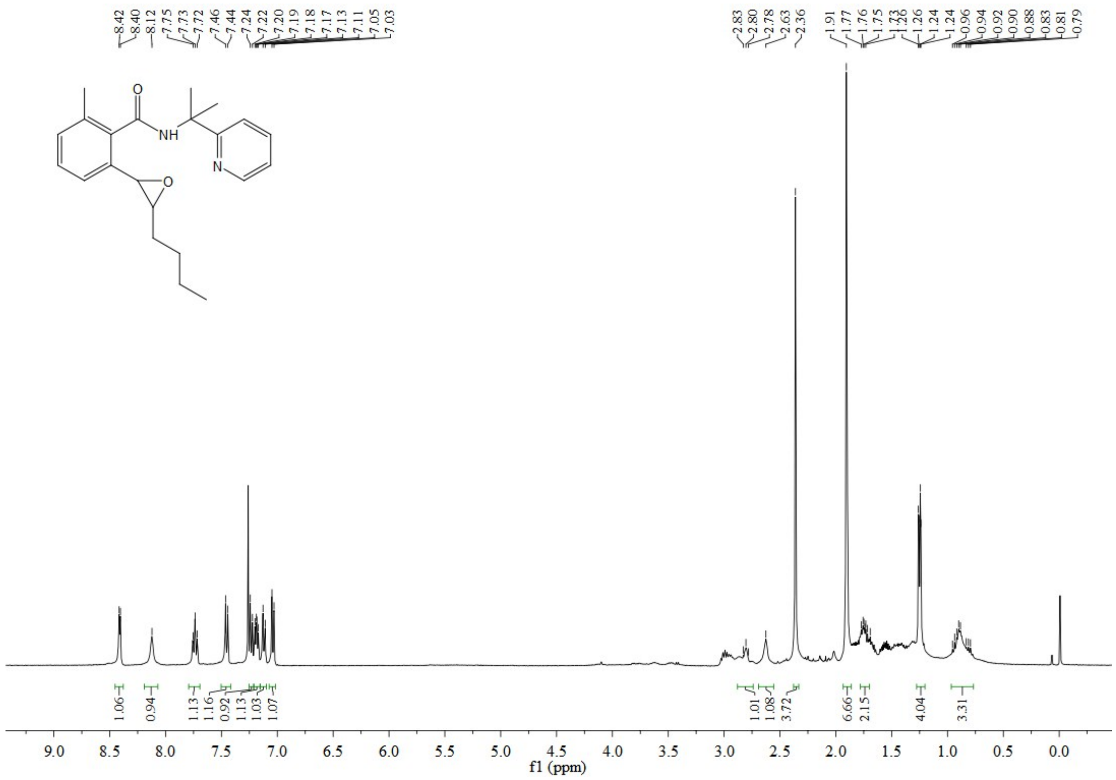

^1H NMR spectrum of compound **3aq**

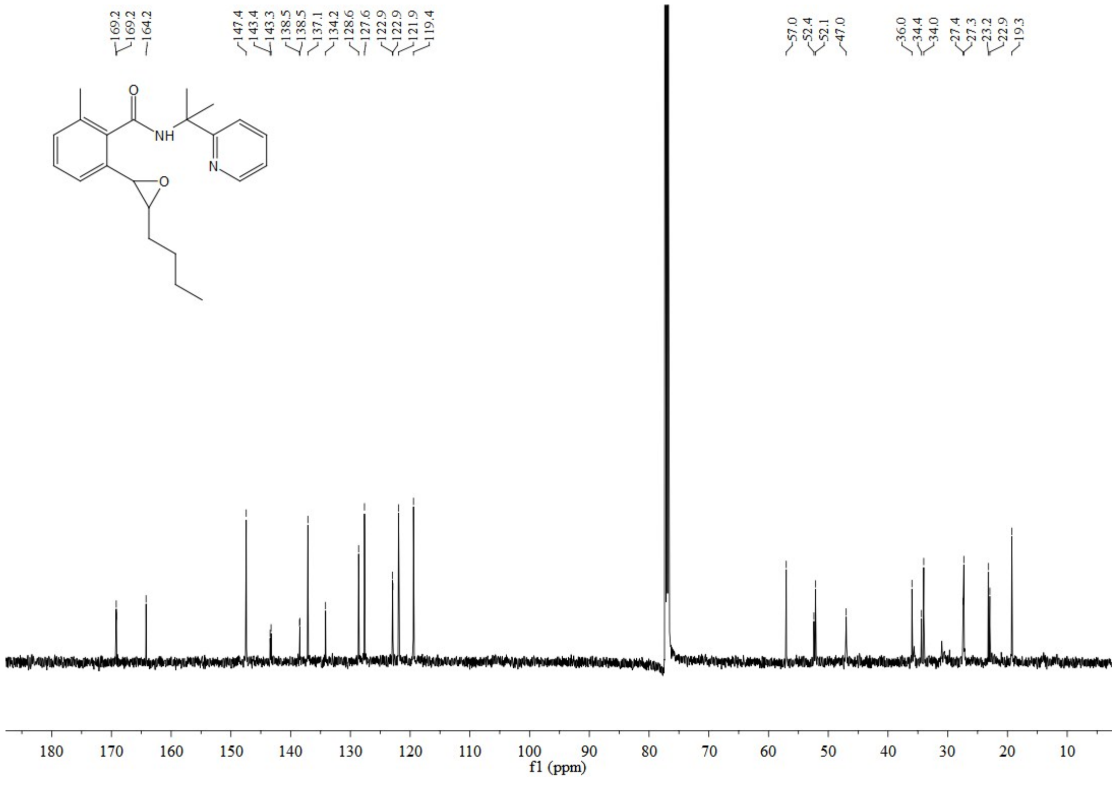

^{13}C NMR spectrum of compound **3aq**

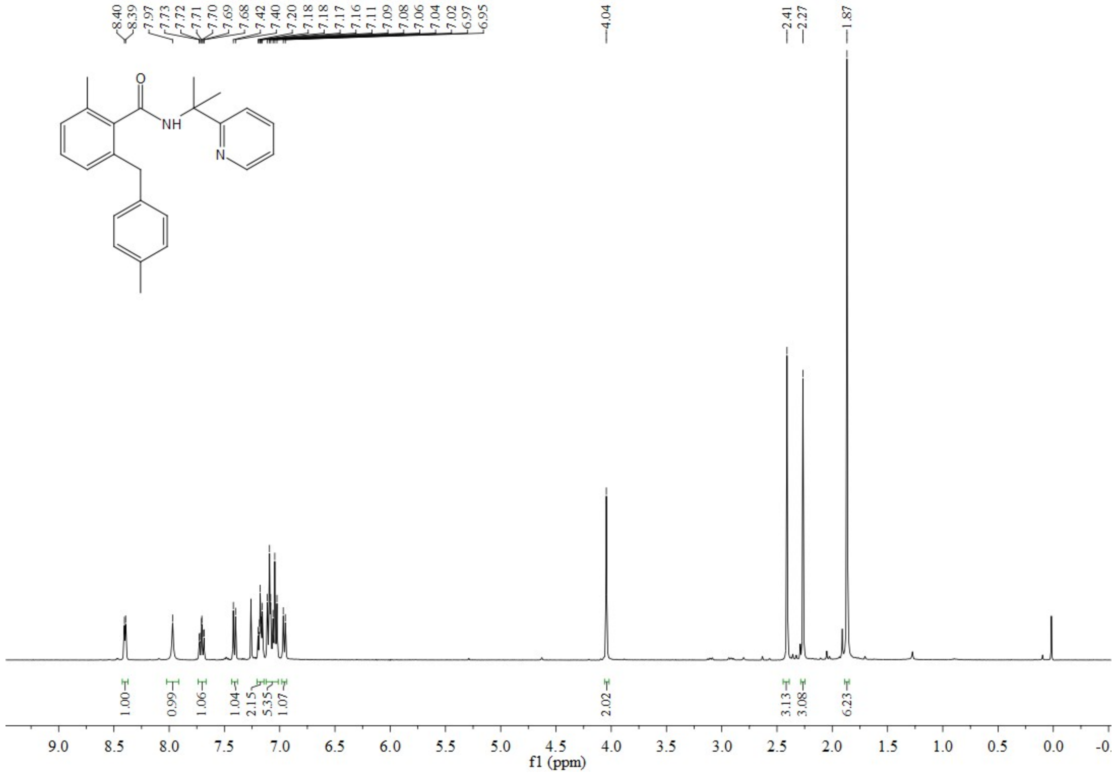

^1H NMR spectrum of compound **3ar**

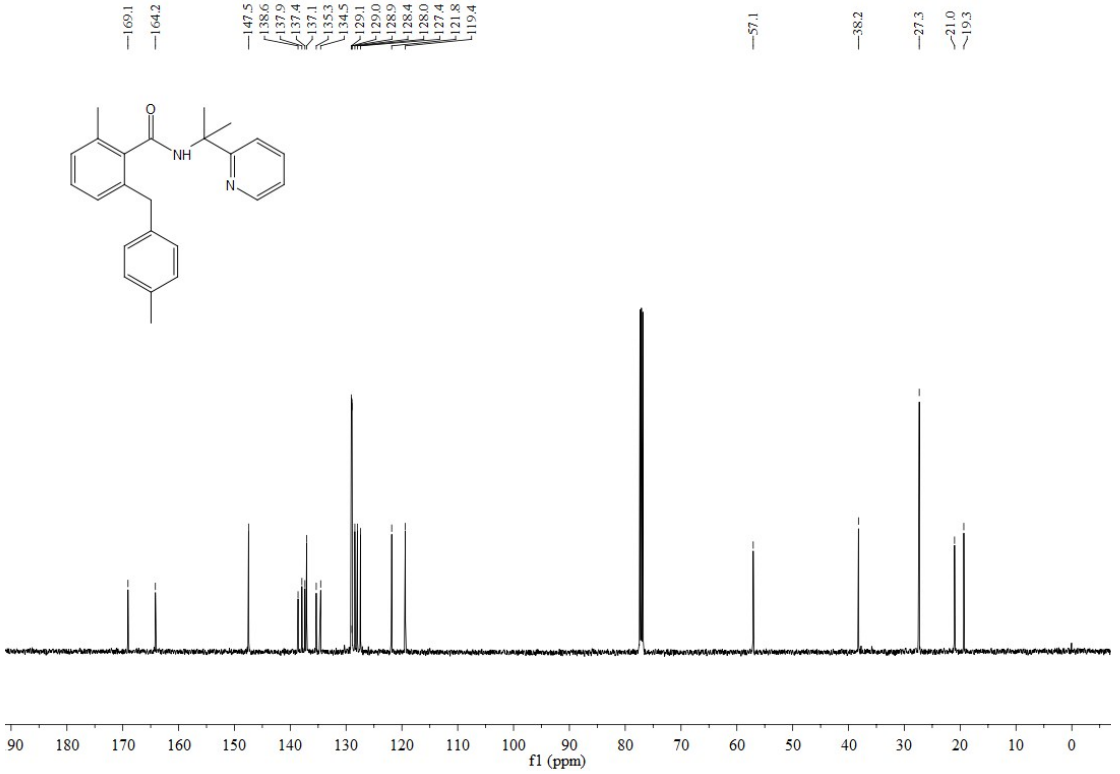

^{13}C NMR spectrum of compound **3ar**

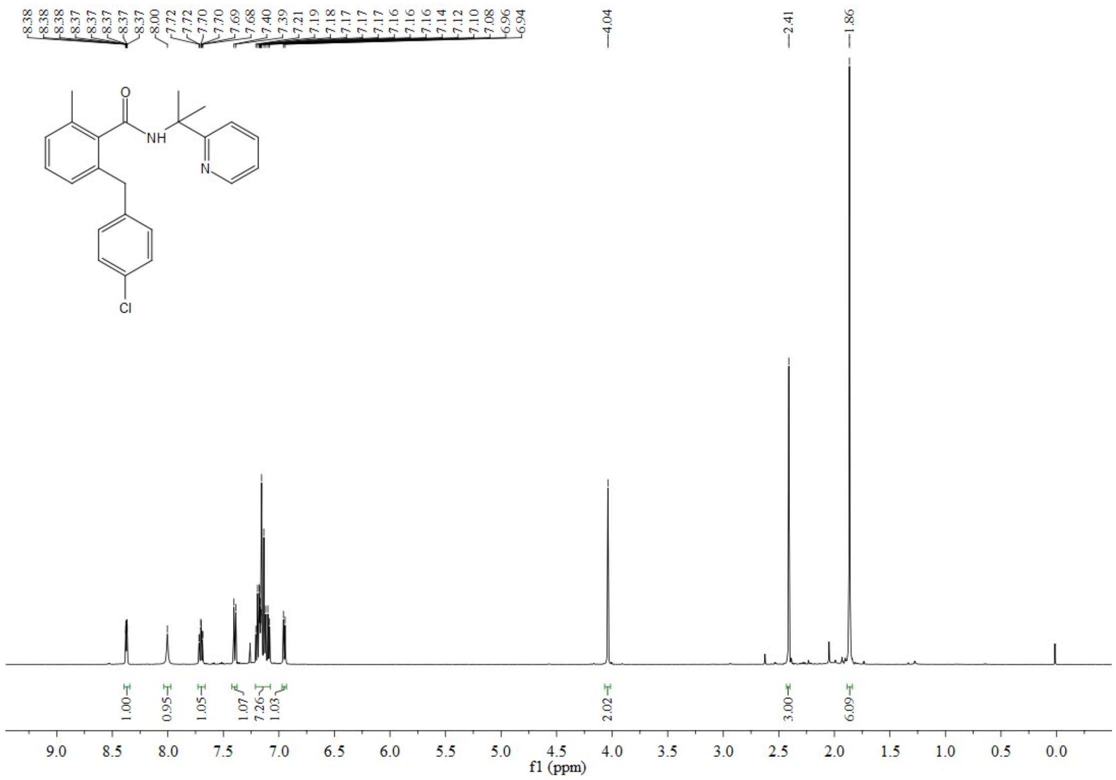

^{1}H NMR spectrum of compound **3as**

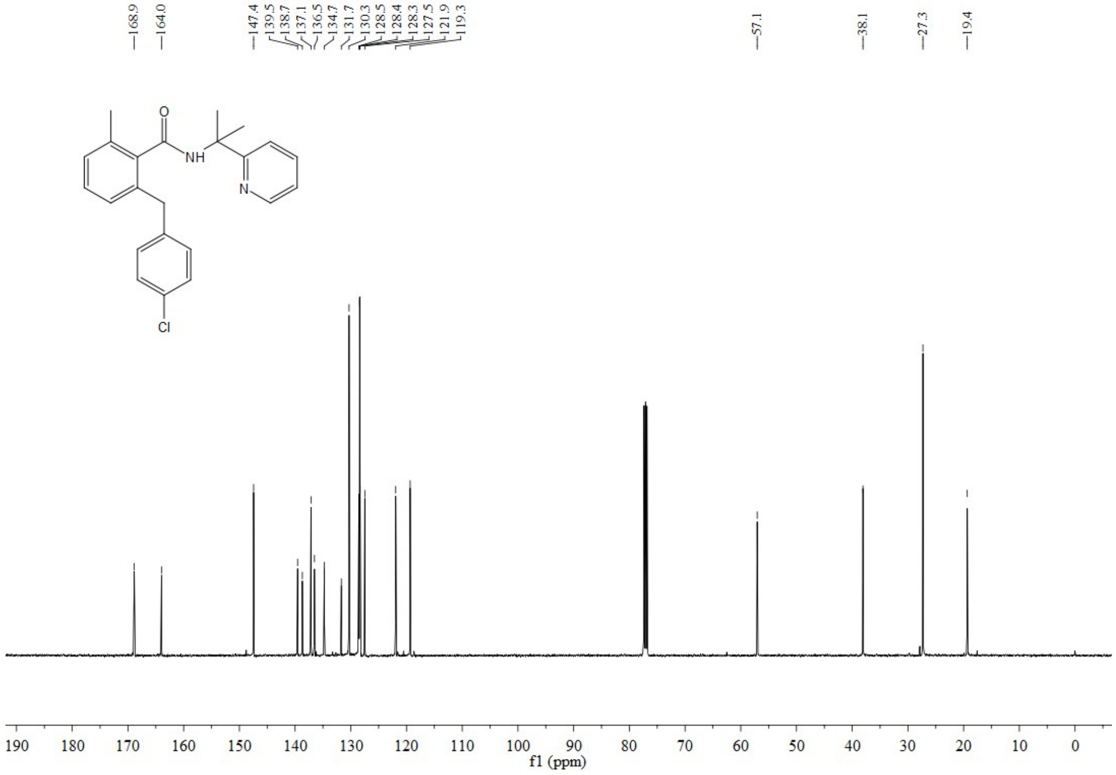

^{13}C NMR spectrum of compound **3as**

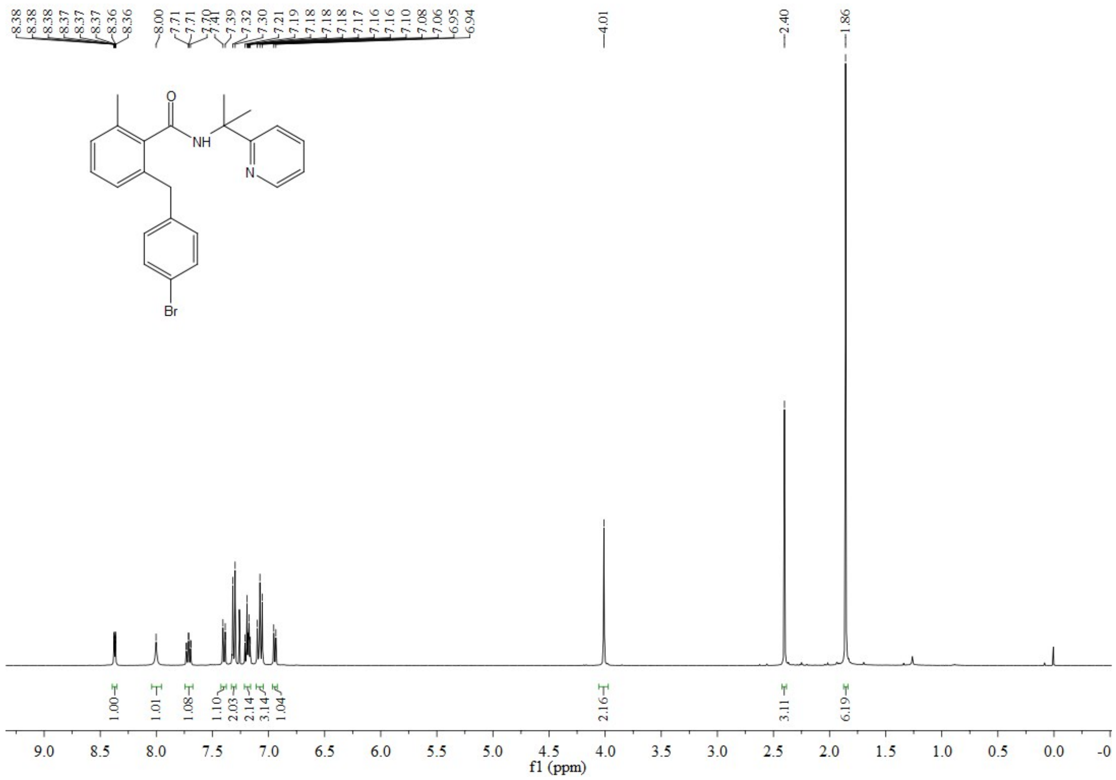

^{1}H NMR spectrum of compound **3at**

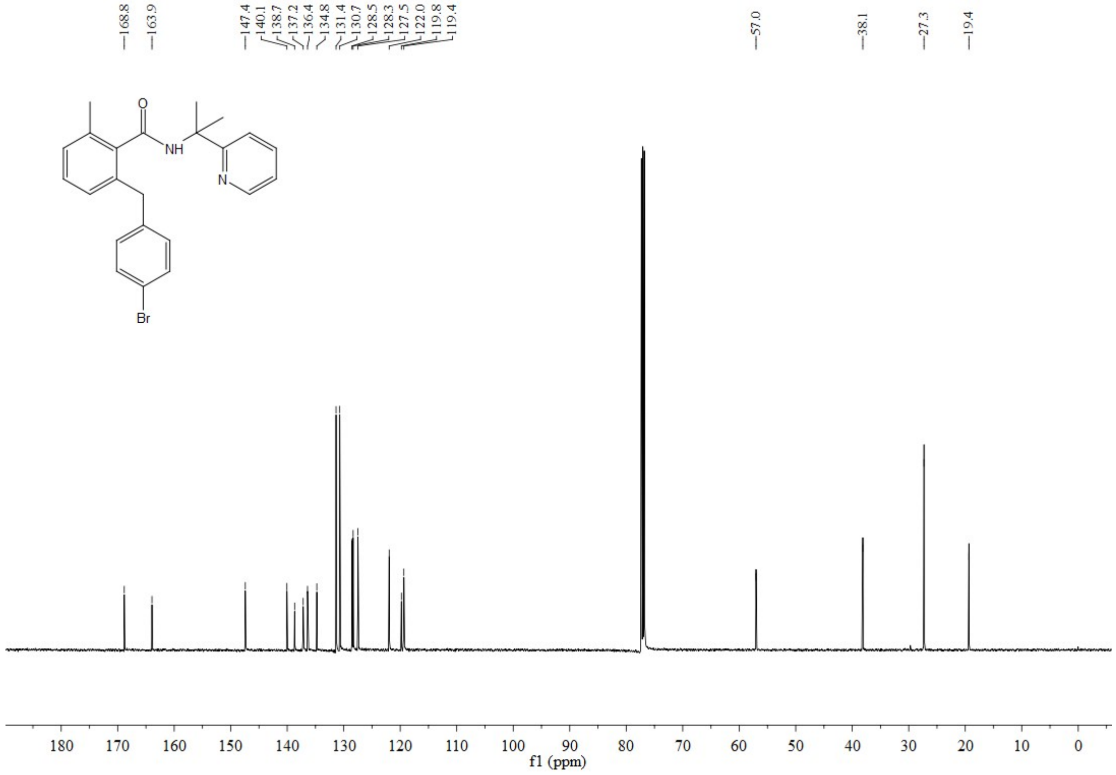

^{13}C NMR spectrum of compound **3at**

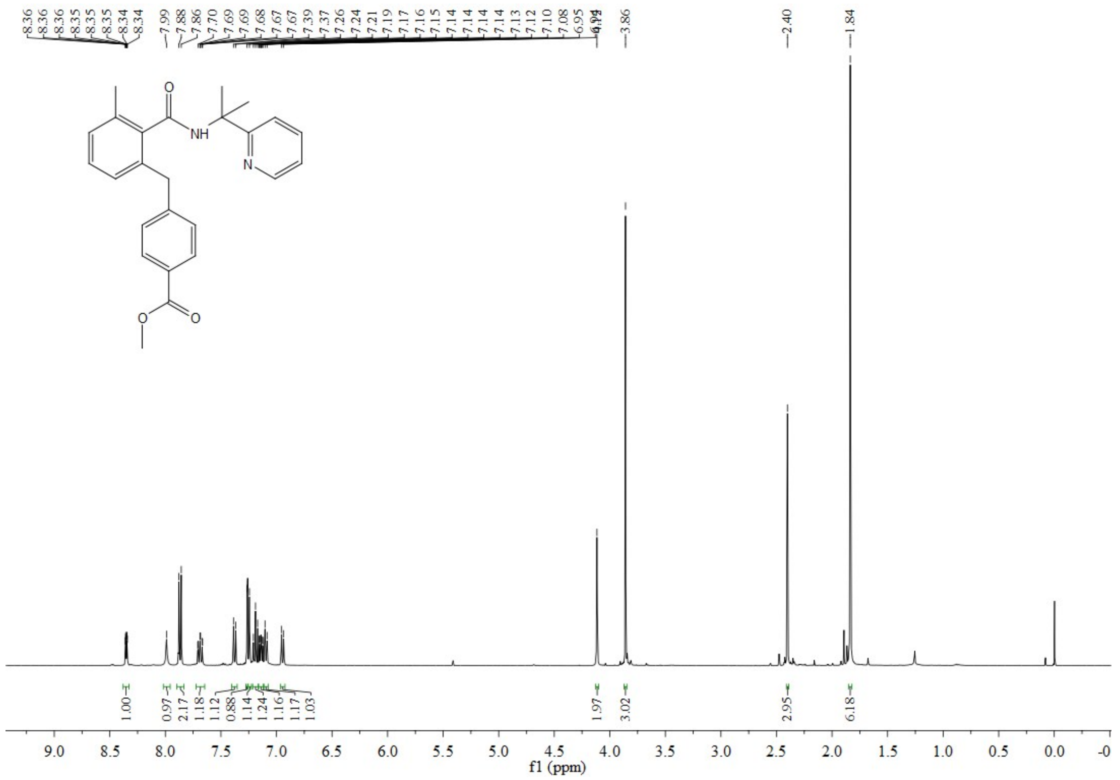

^{1}H NMR spectrum of compound **3au**

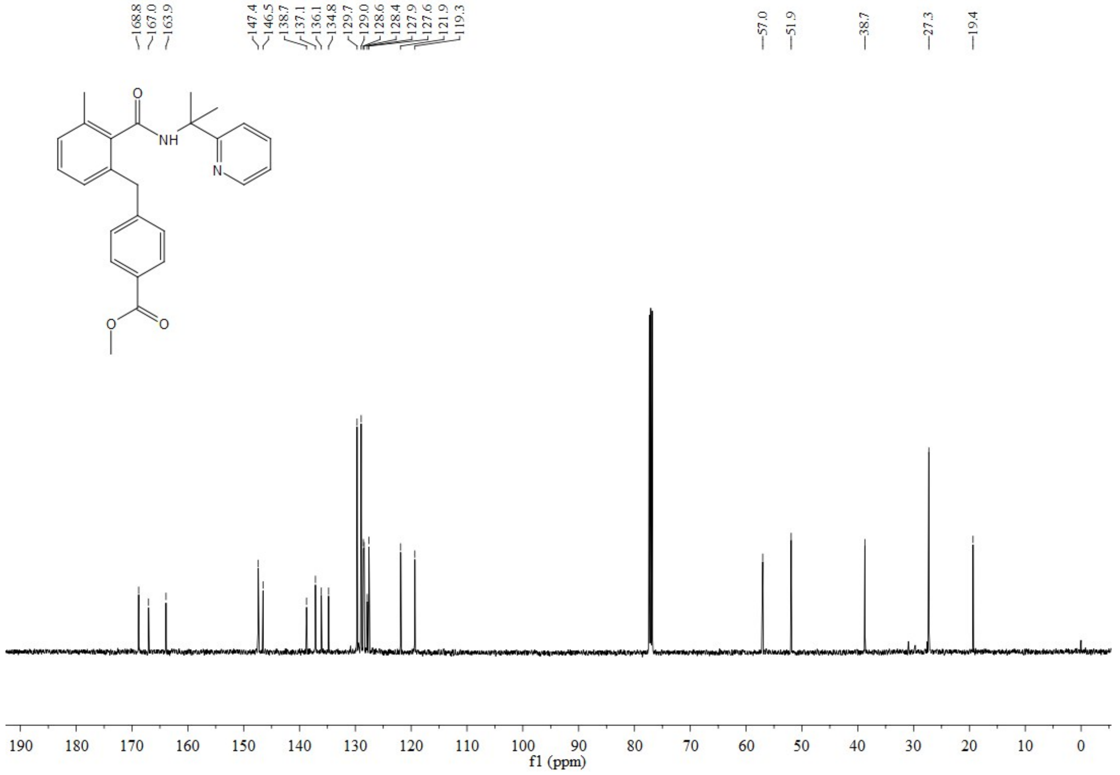

^{13}C NMR spectrum of compound **3au**

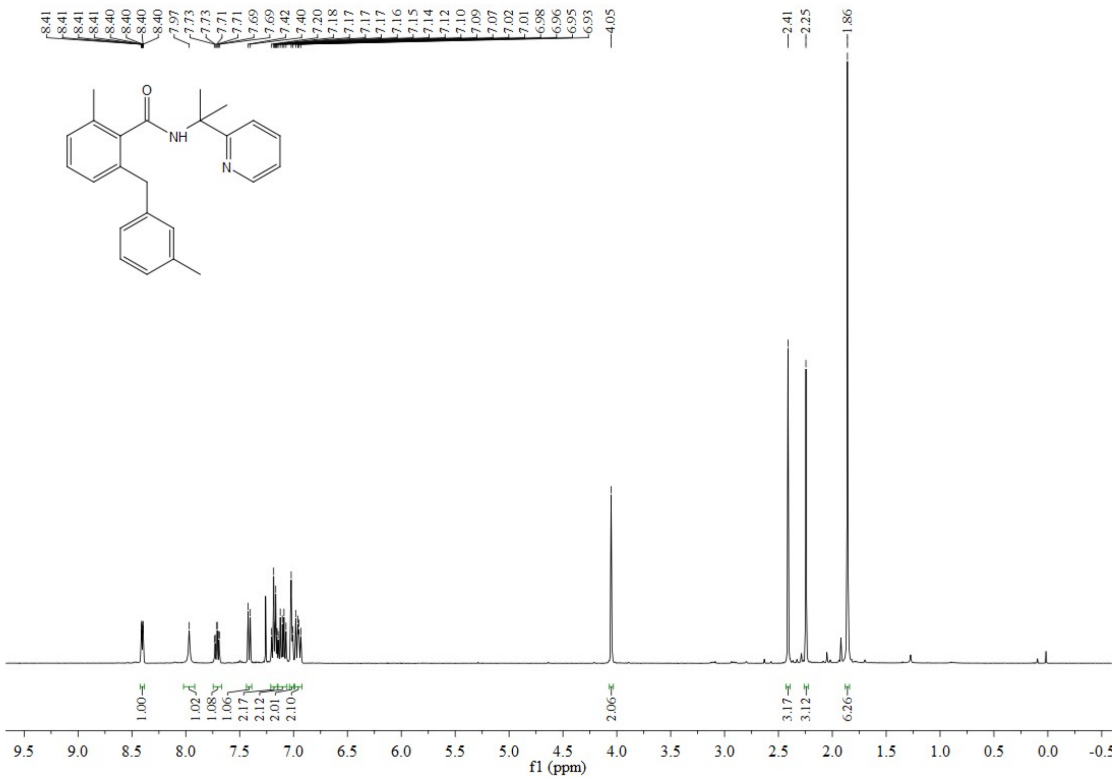

^{1}H NMR spectrum of compound **3av**

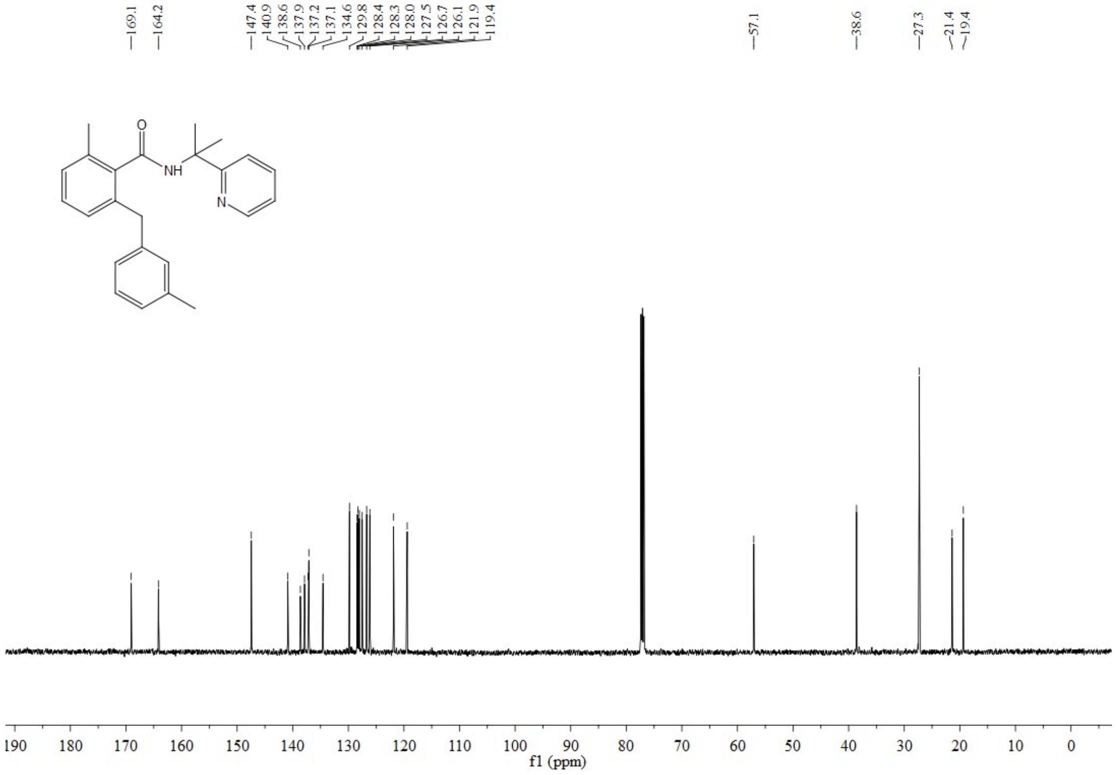

^{13}C NMR spectrum of compound **3av**

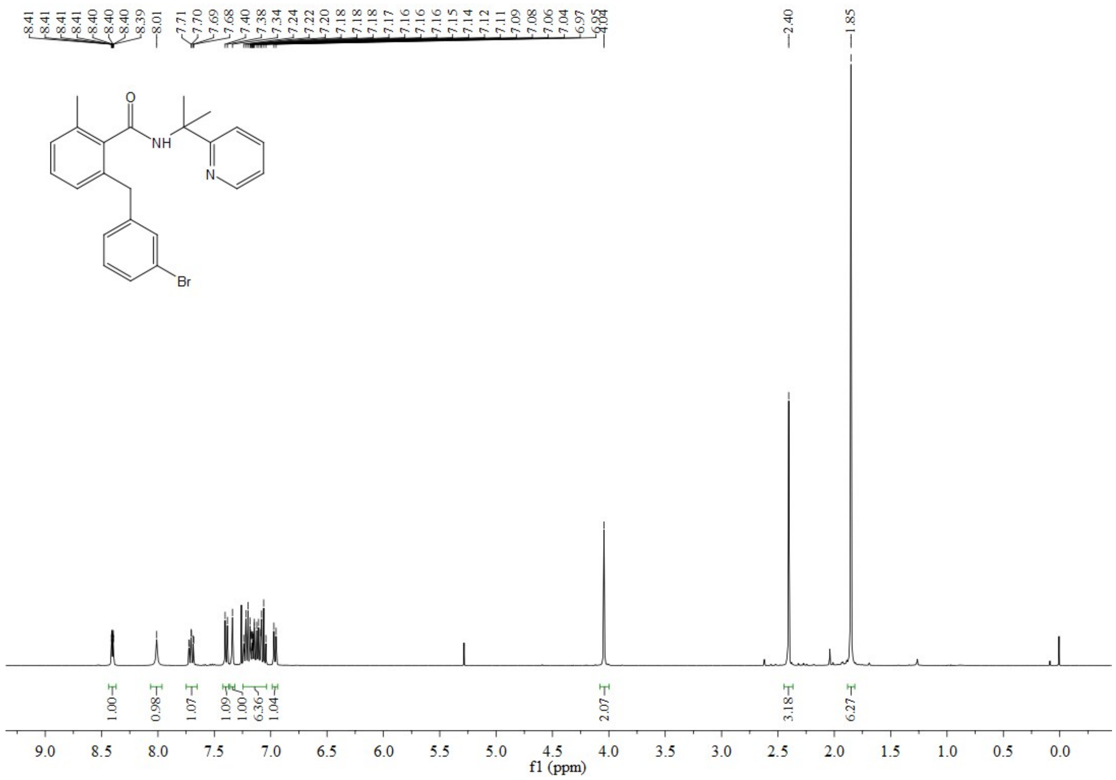

^{1}H NMR spectrum of compound **3aw**

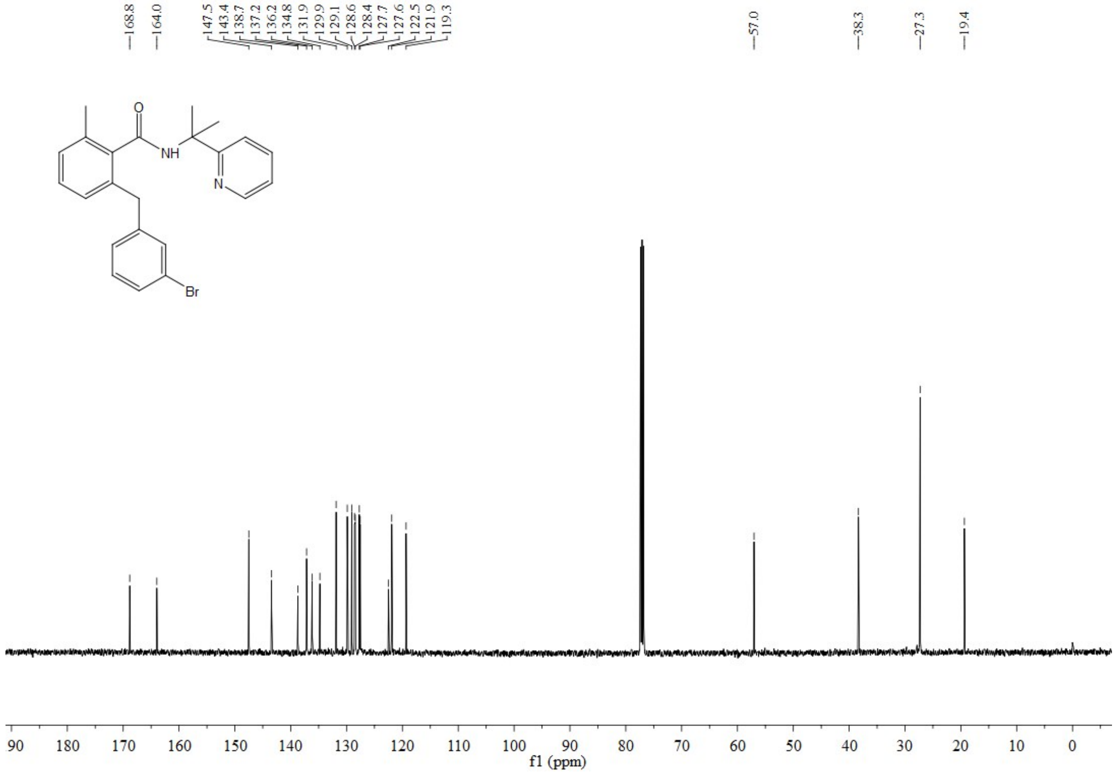

^{13}C NMR spectrum of compound **3aw**

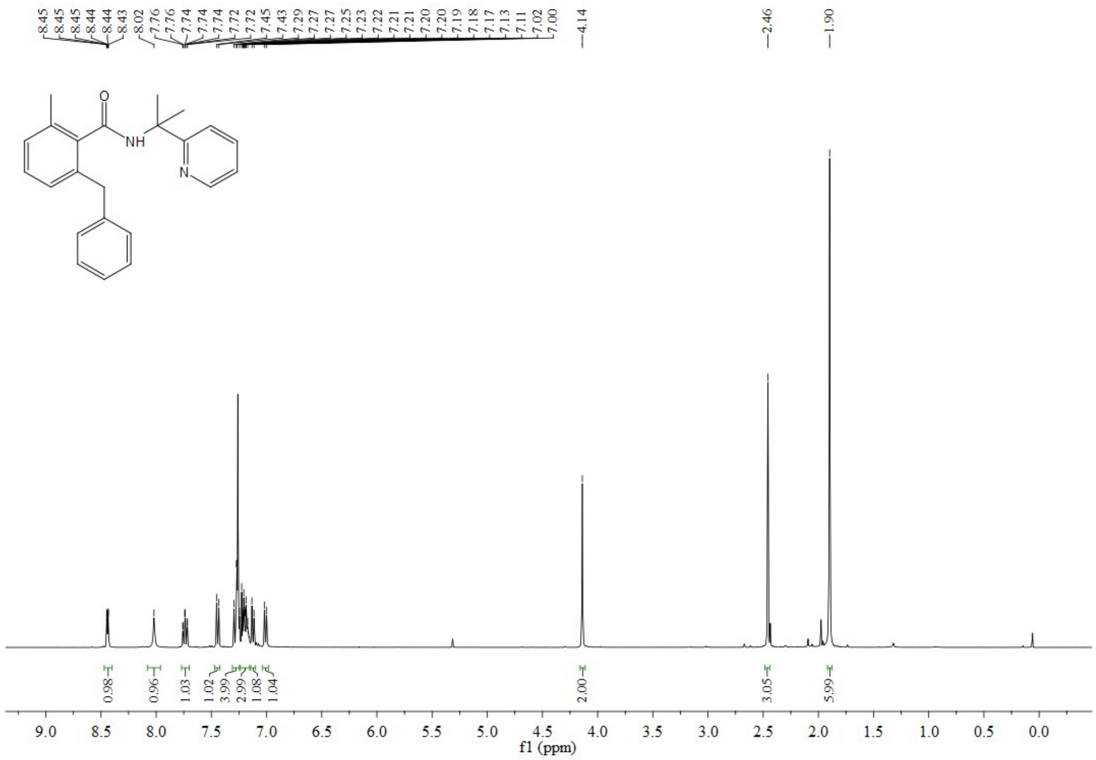

^{1}H NMR spectrum of compound **3ax**

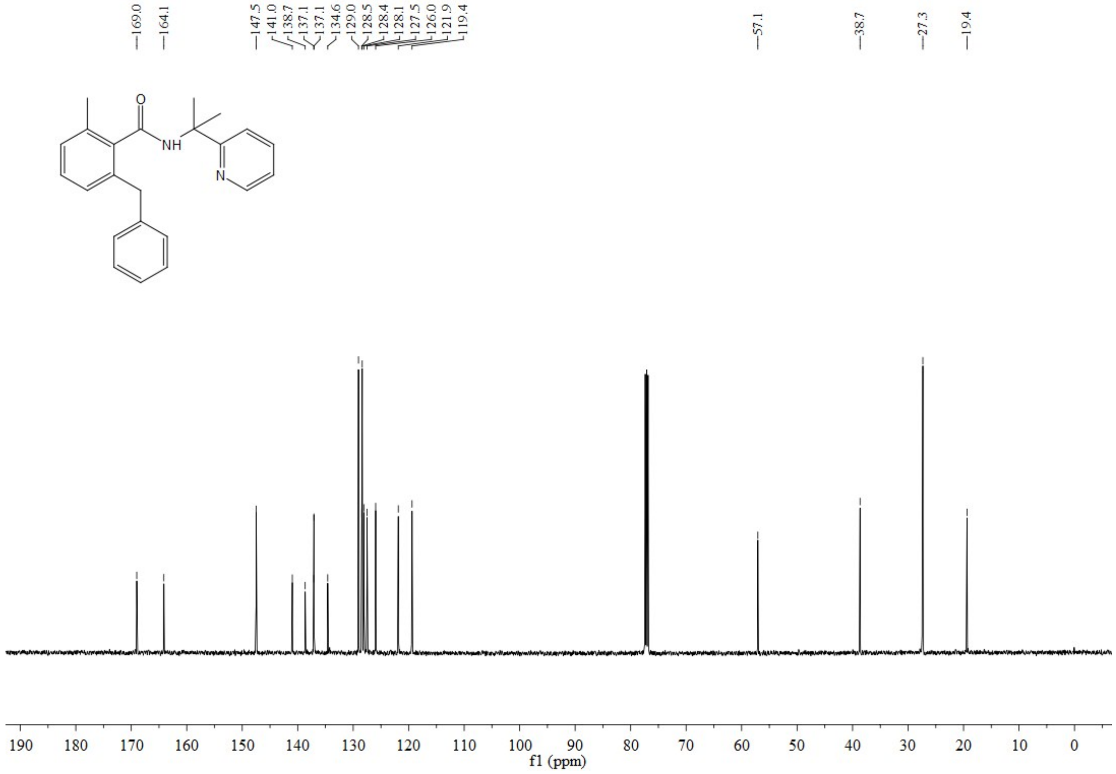

^{13}C NMR spectrum of compound **3ax**

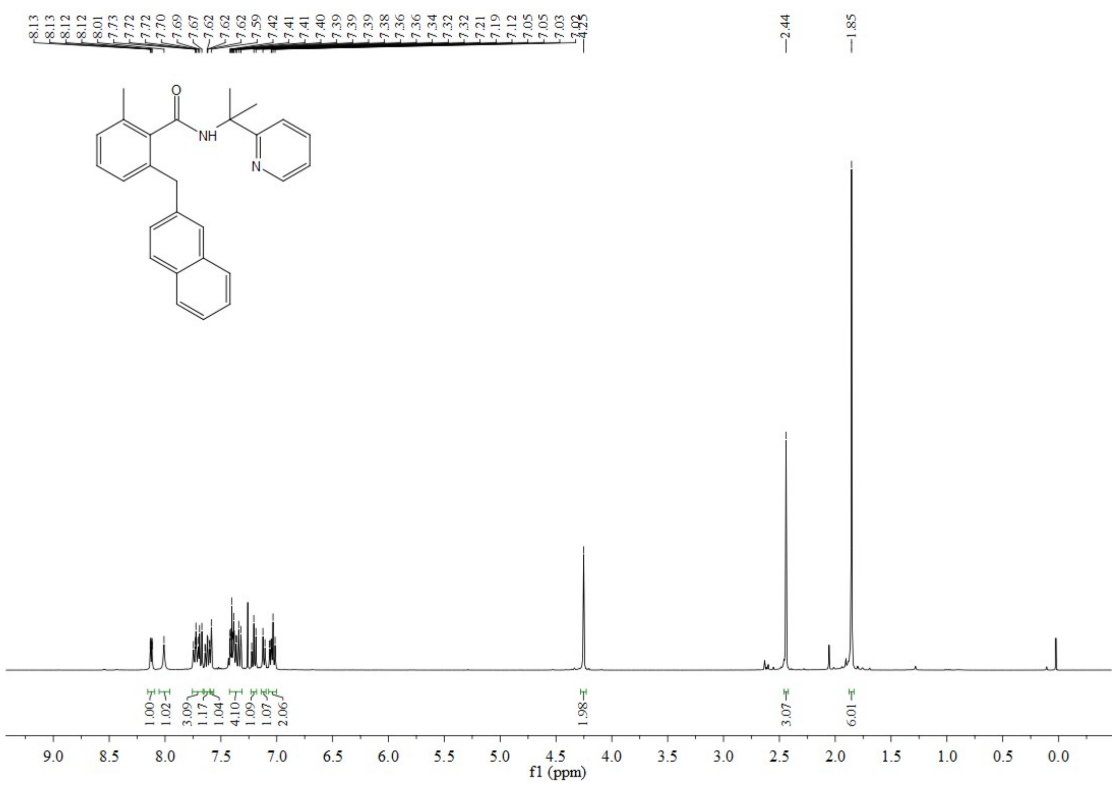

^{1}H NMR spectrum of compound **3ay**

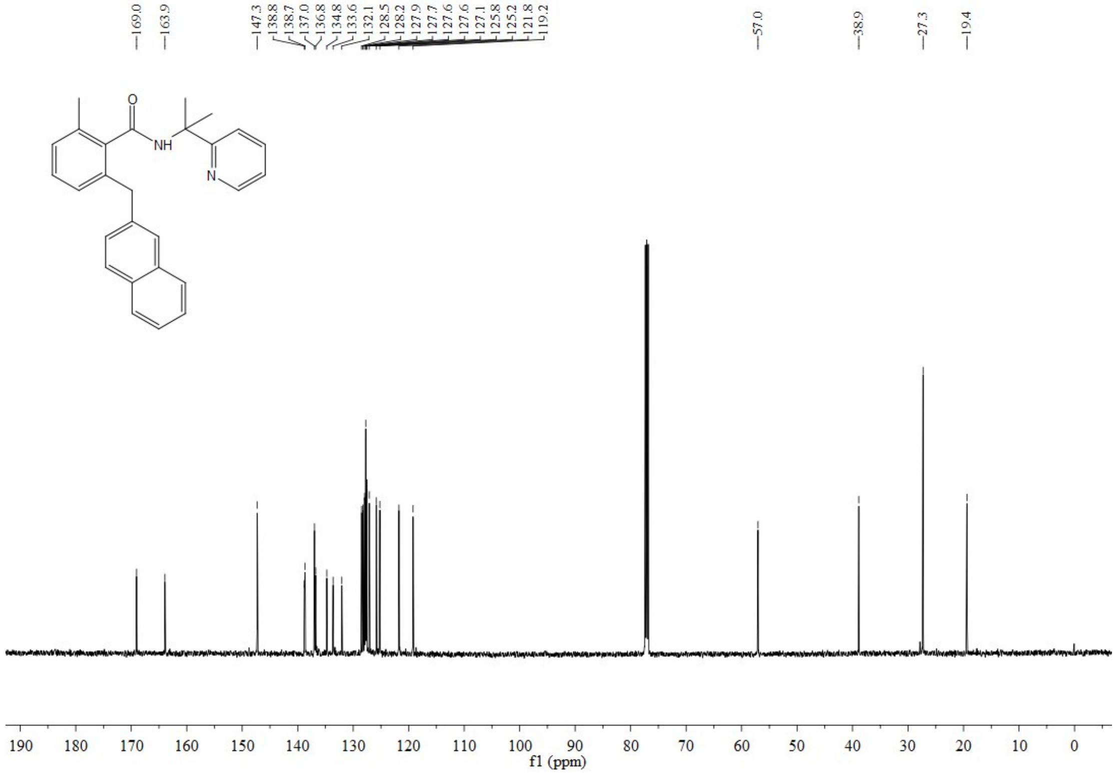

^{13}C NMR spectrum of compound **3ay**

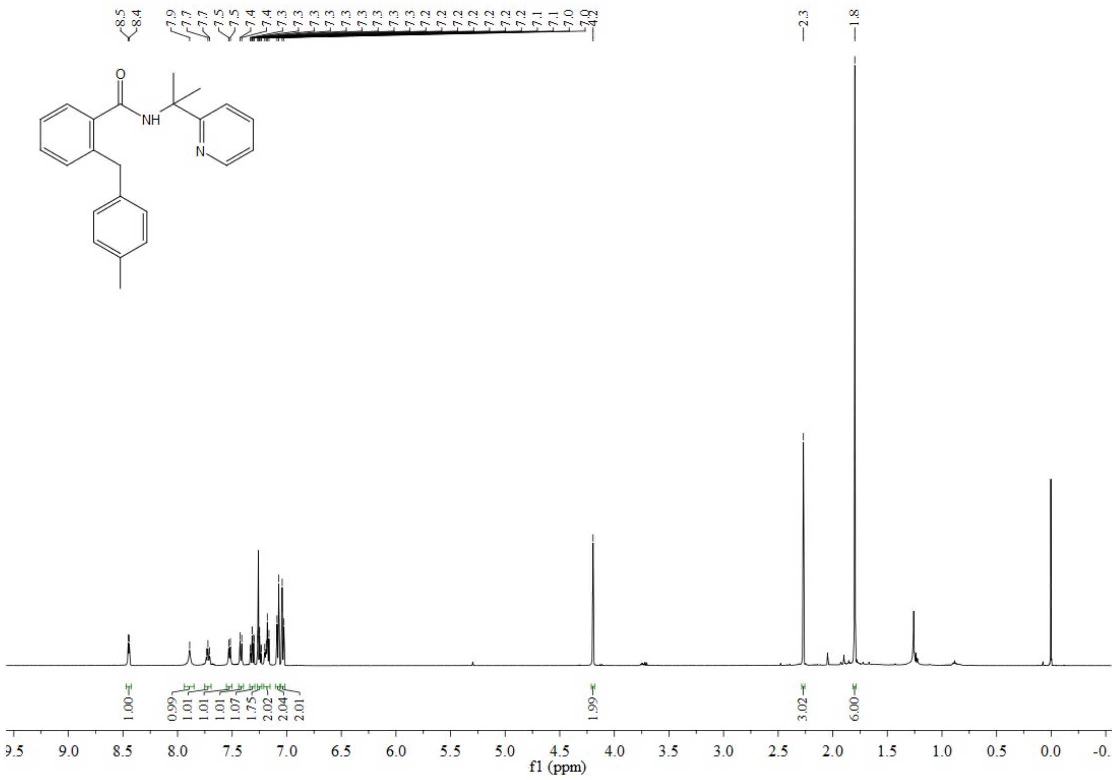

^{1}H NMR spectrum of compound **3fr**

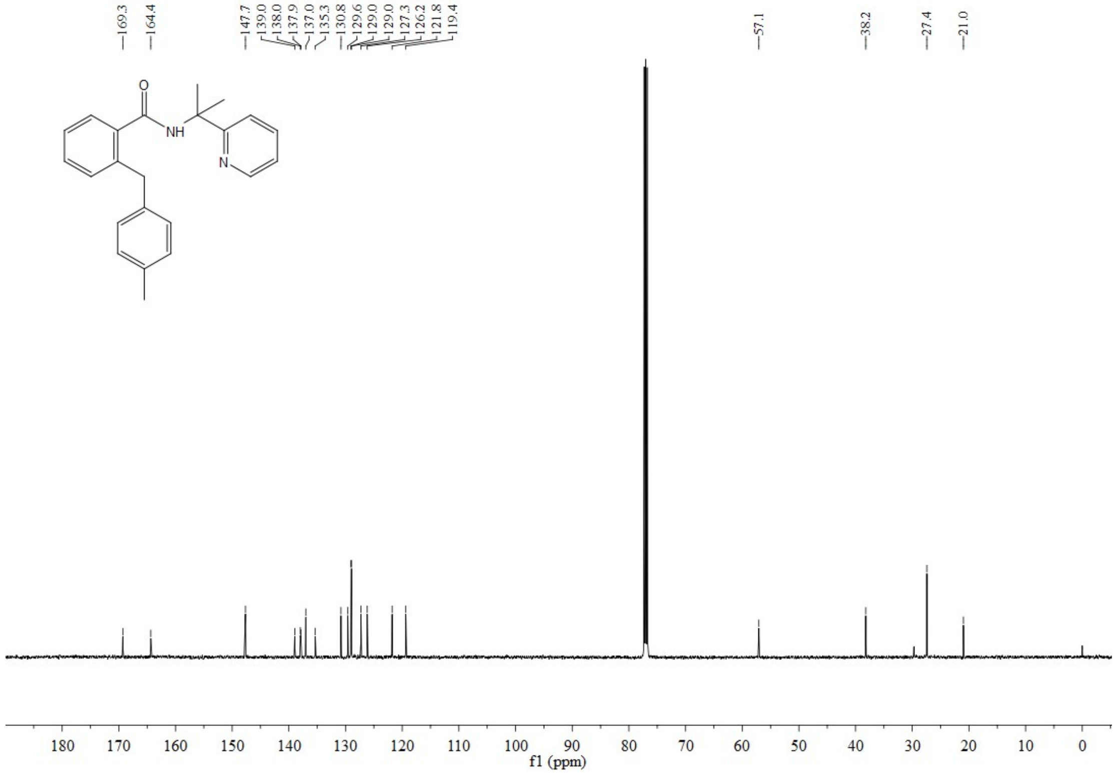

^{13}C NMR spectrum of compound **3fr**

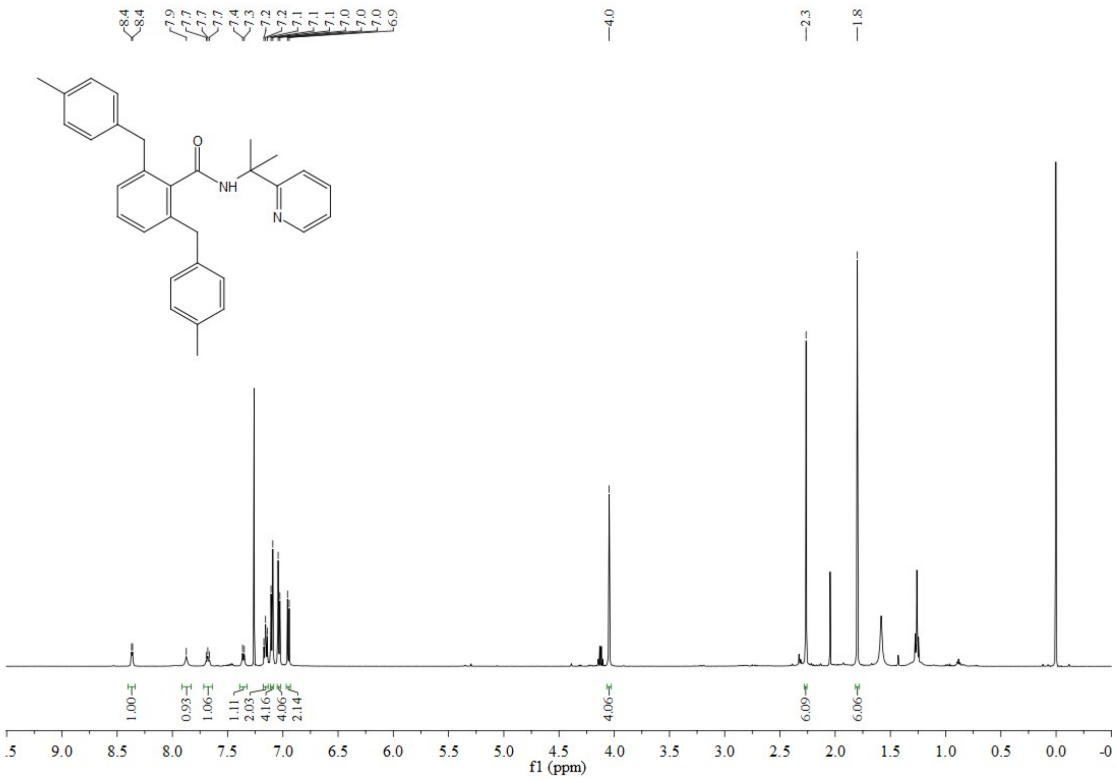

^{1}H NMR spectrum of compound **3fr'**

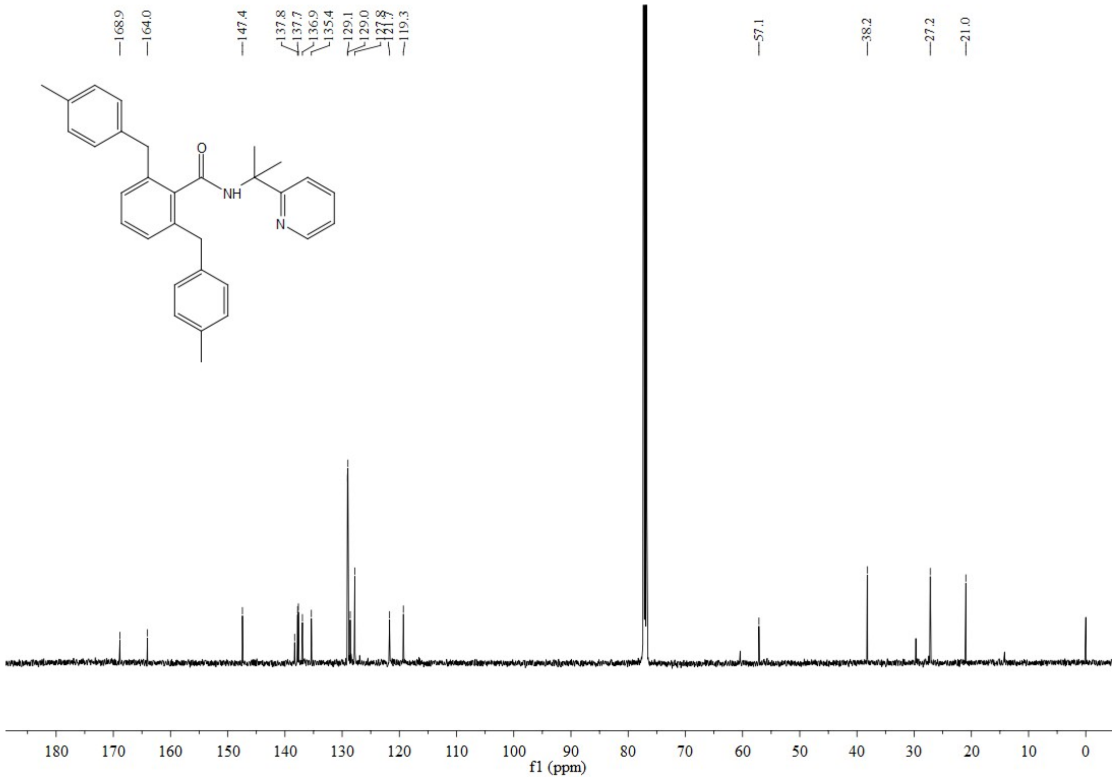

^{13}C NMR spectrum of compound **3fr'**

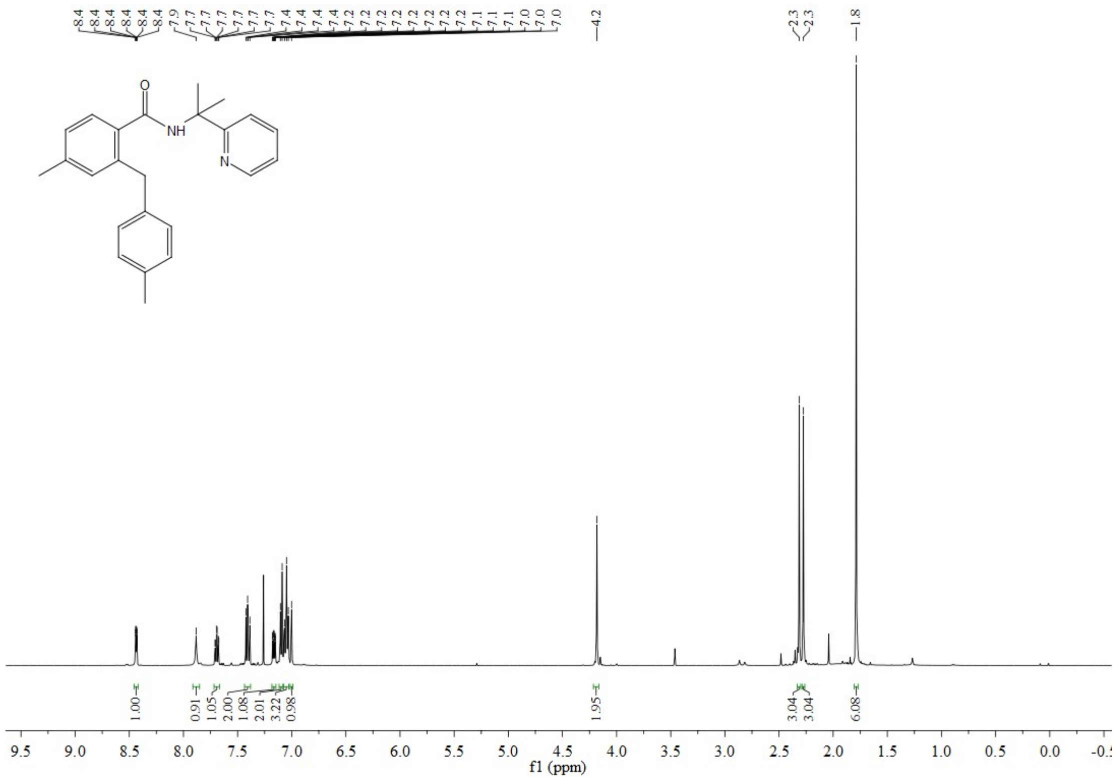

^{1}H NMR spectrum of compound **3kr**

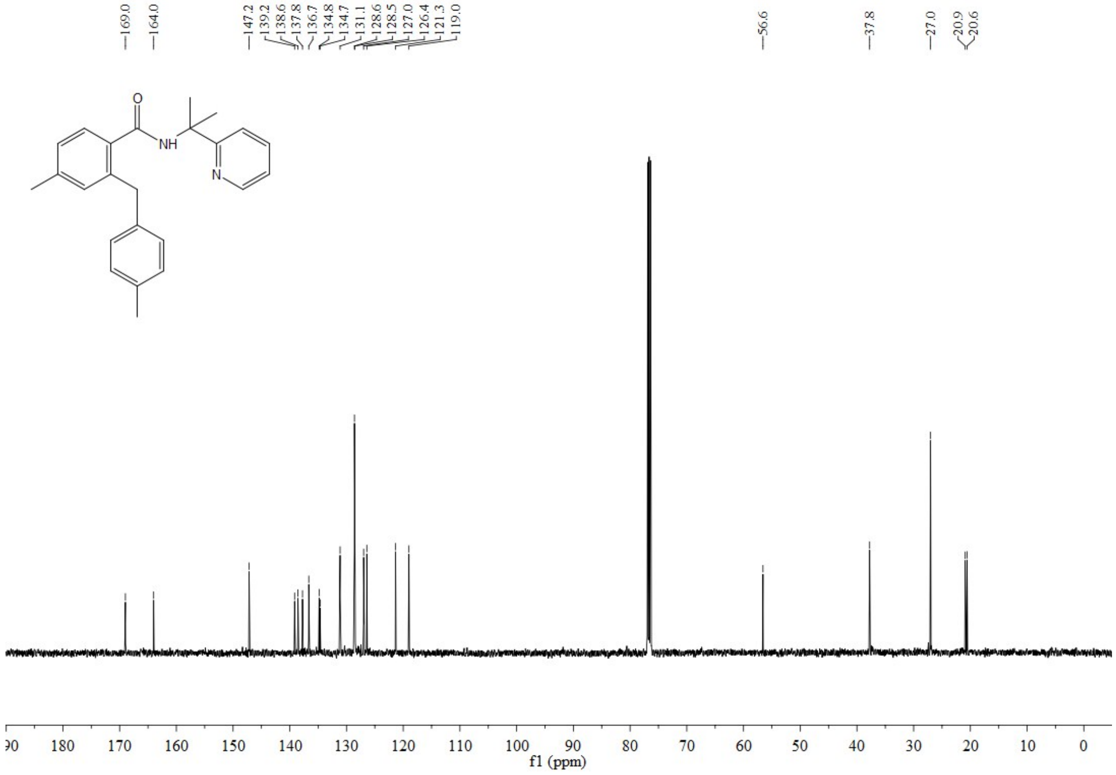

^{13}C NMR spectrum of compound **3kr**

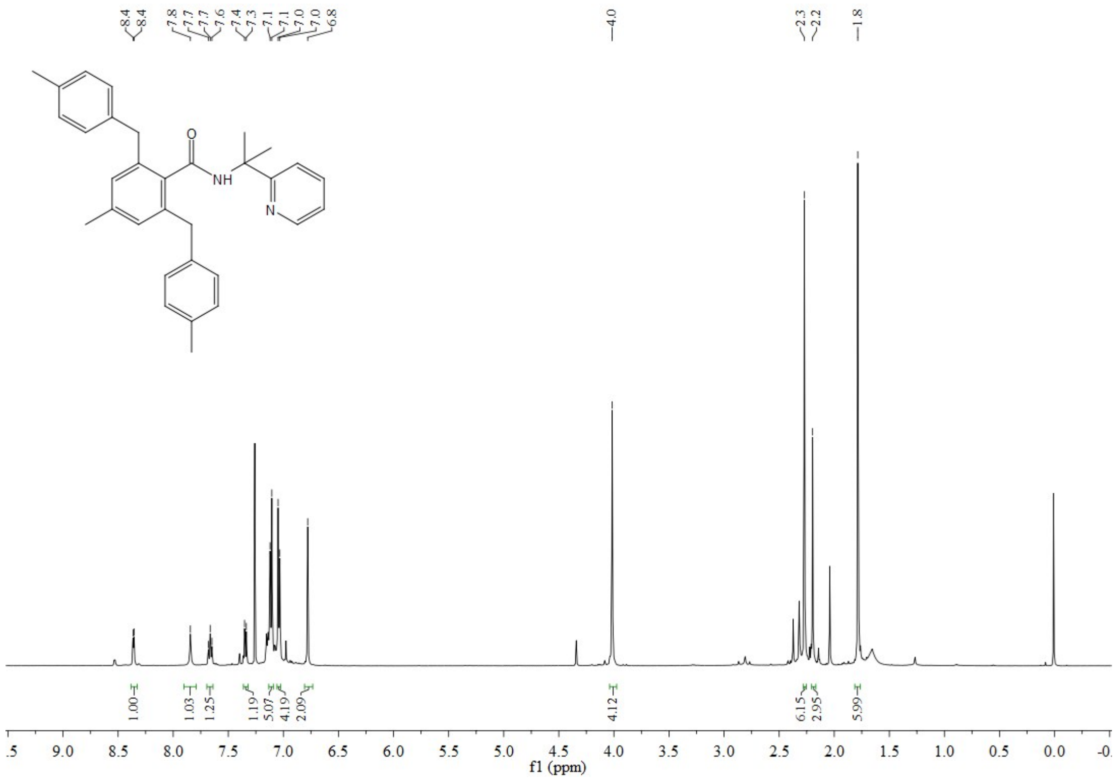

^{1}H NMR spectrum of compound **3kr'**

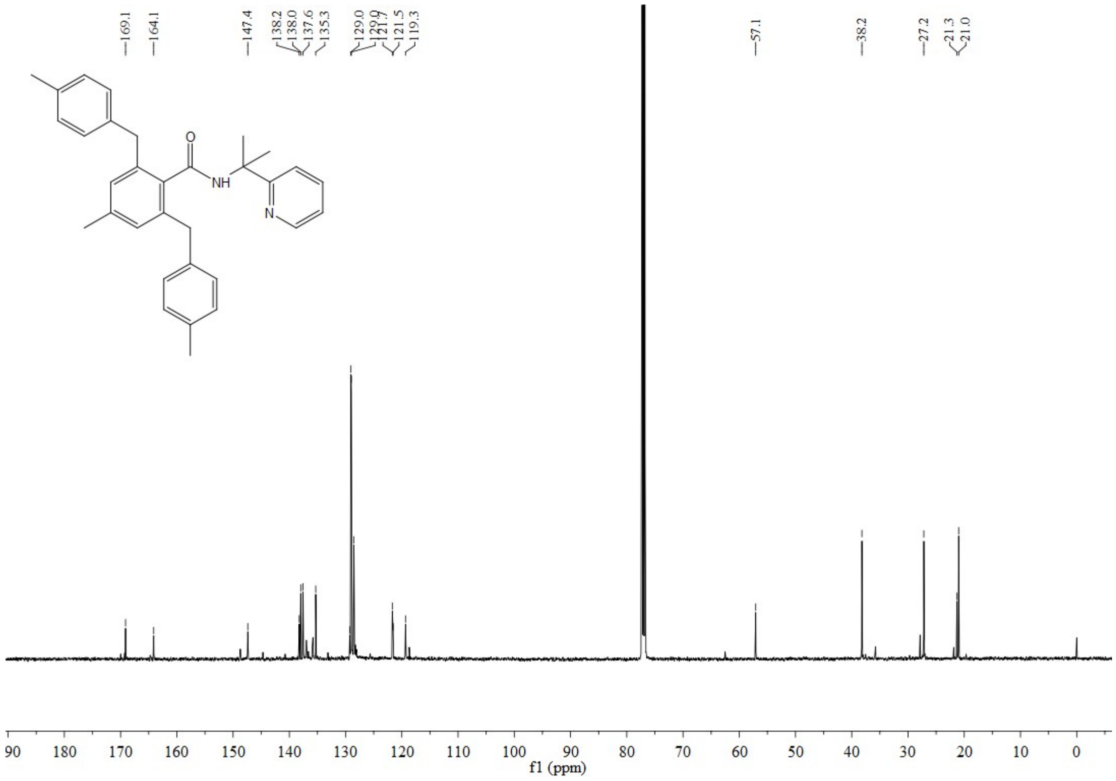

^{13}C NMR spectrum of compound **3kr'**

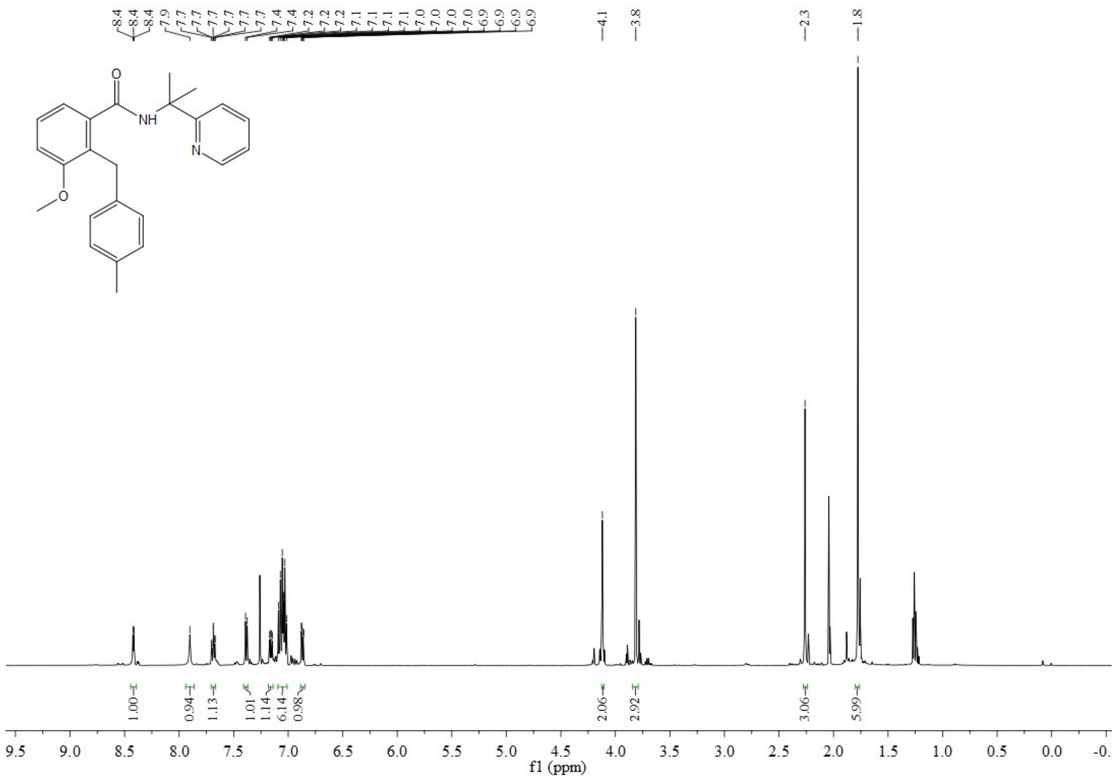

^{1}H NMR spectrum of compound **3or**

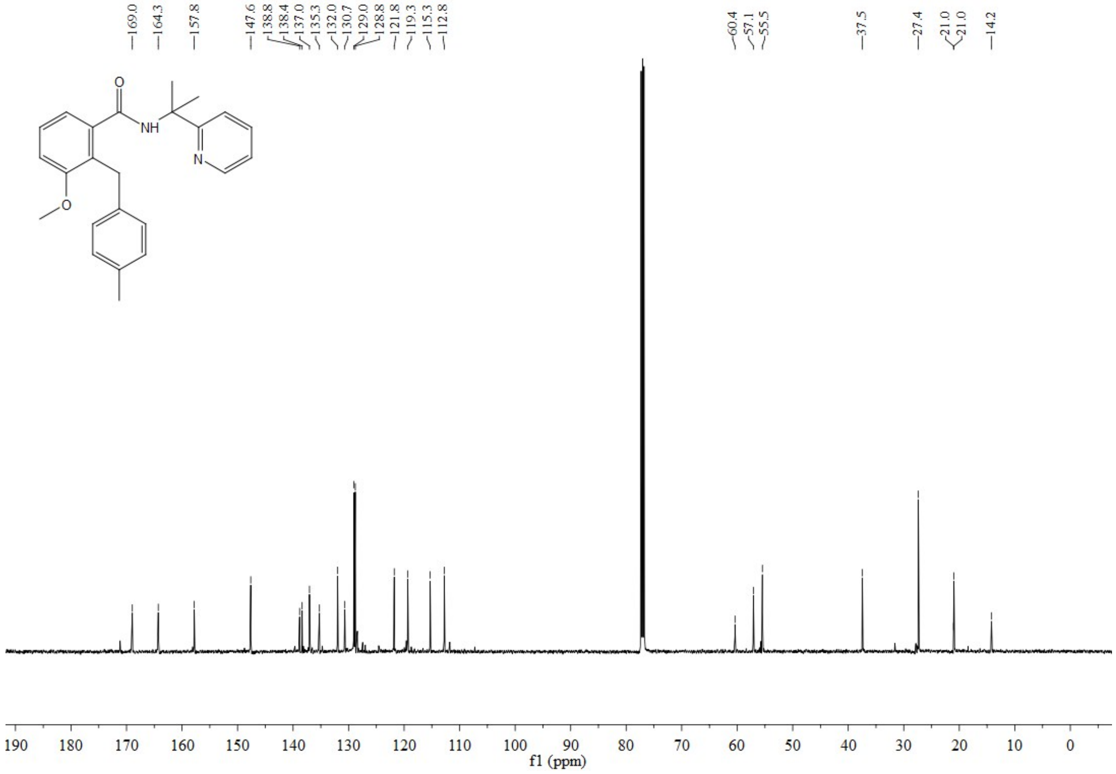

^{13}C NMR spectrum of compound **3or**

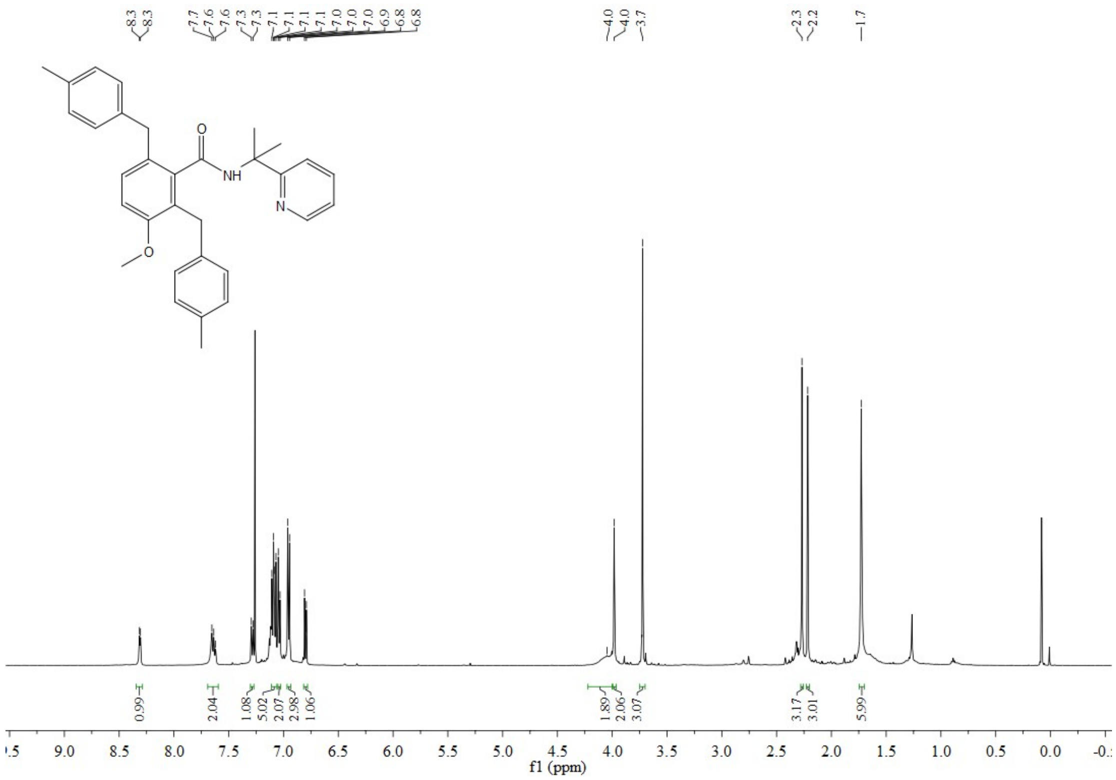

^{1}H NMR spectrum of compound **3or'**

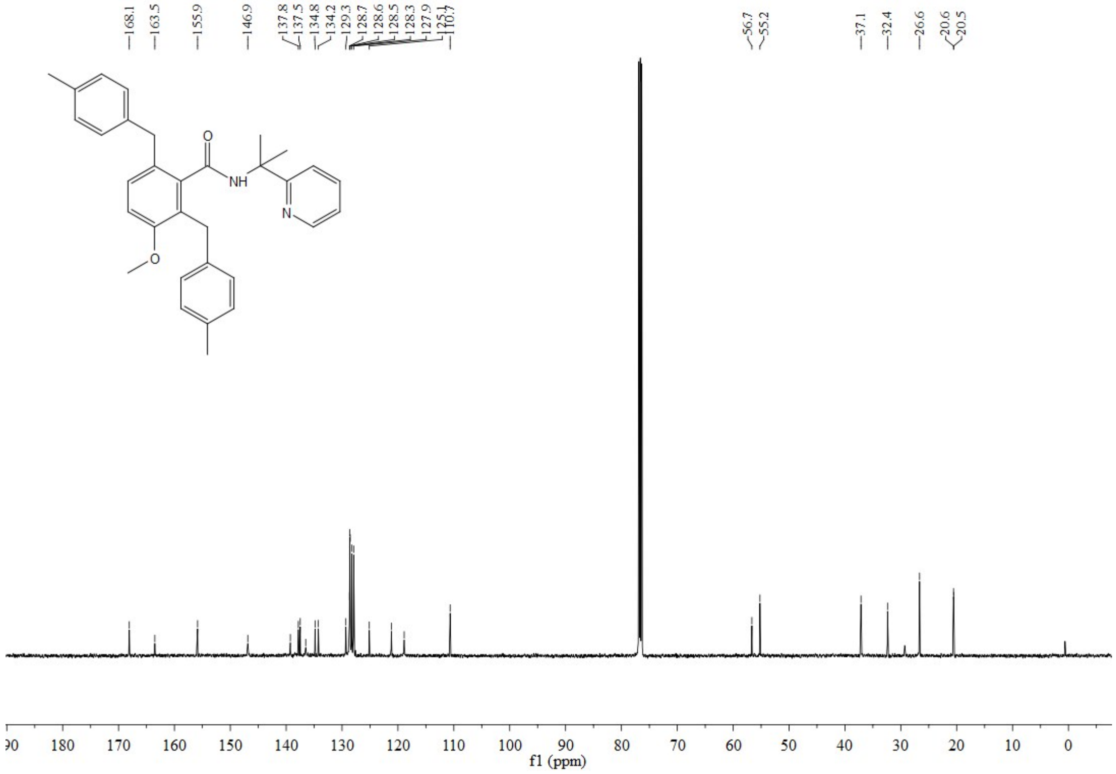

^{13}C NMR spectrum of compound **3or'**

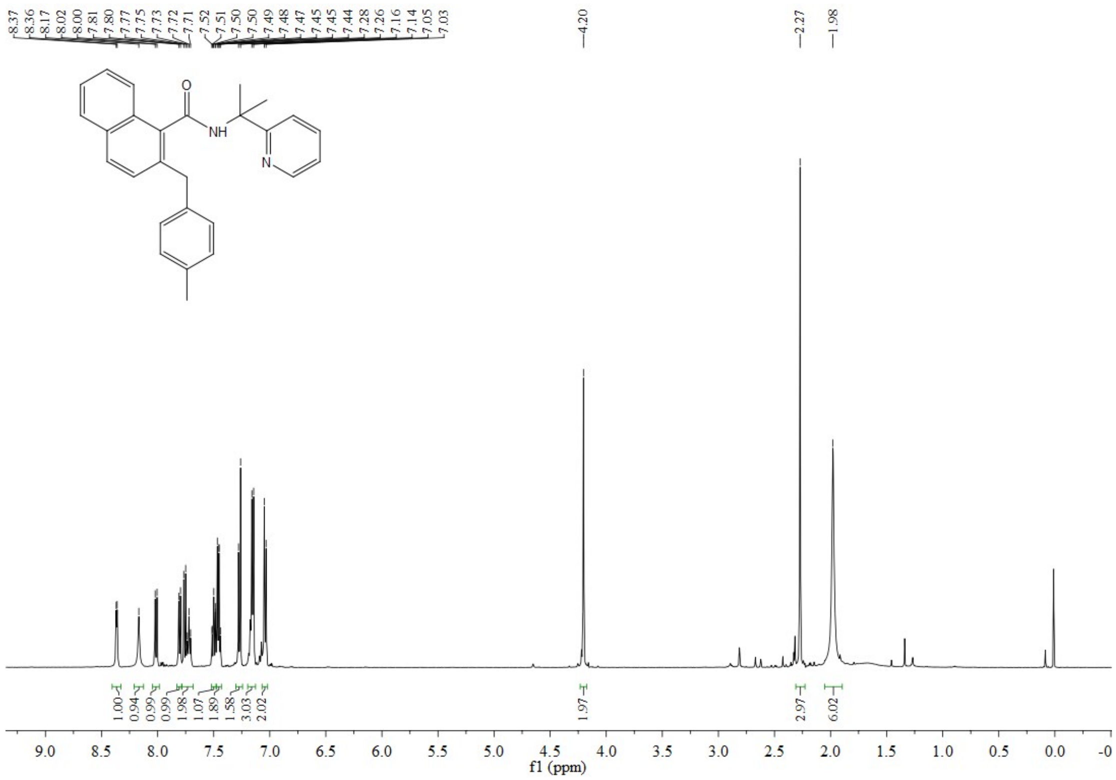

^{1}H NMR spectrum of compound **3mr**

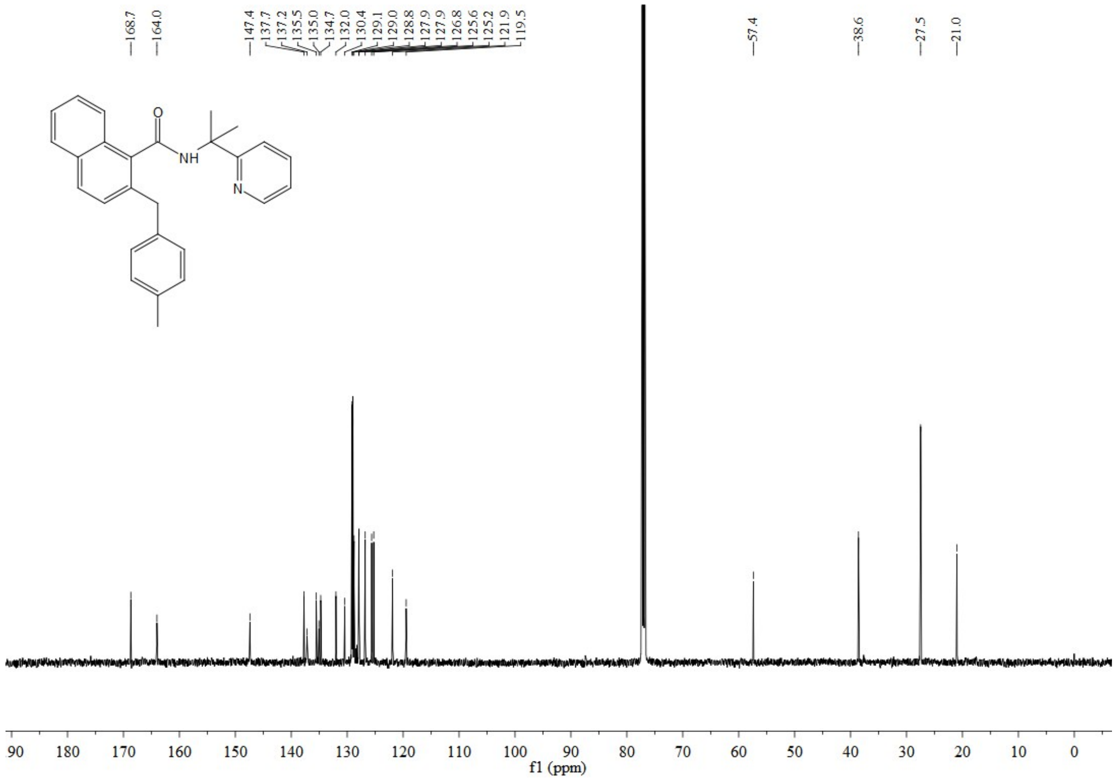

^{13}C NMR spectrum of compound **3mr**

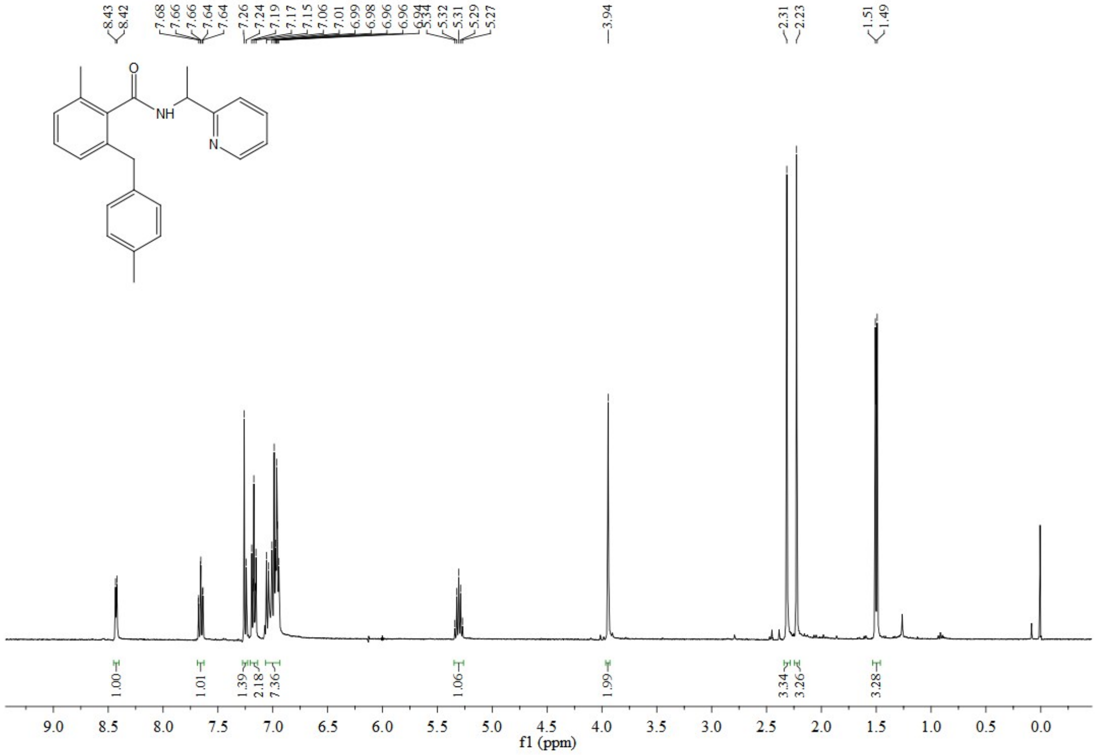

^{1}H NMR spectrum of compound **3qr**

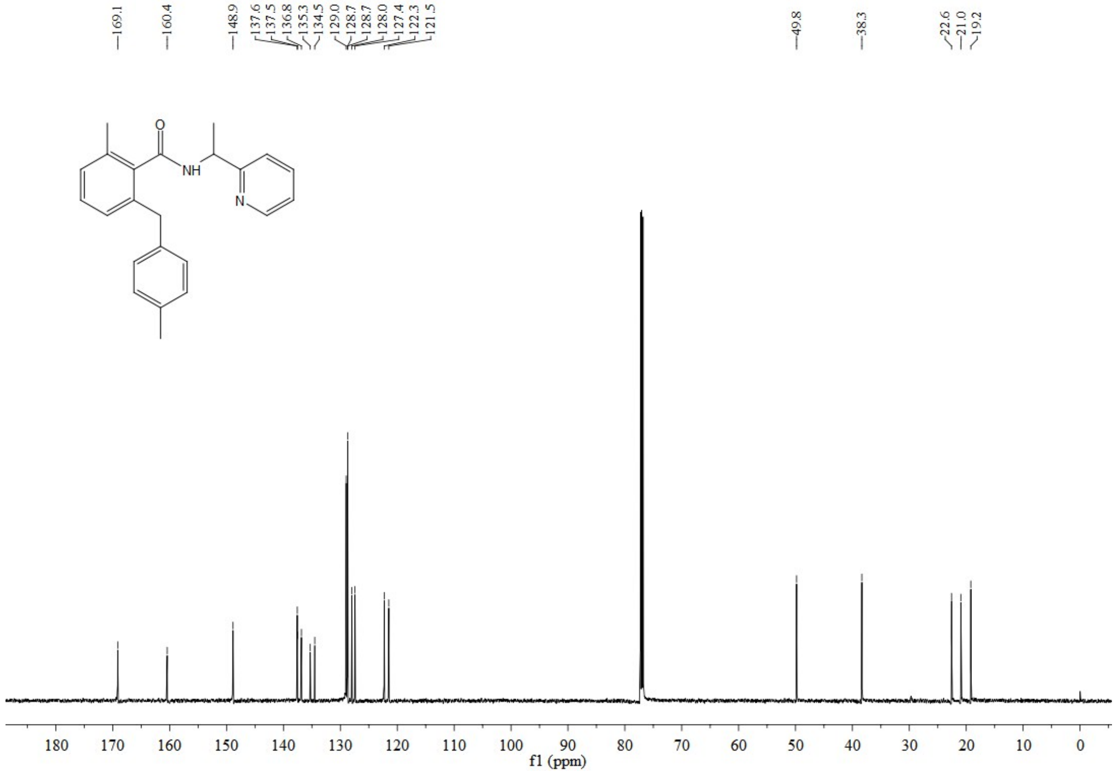

^{13}C NMR spectrum of compound **3qr**

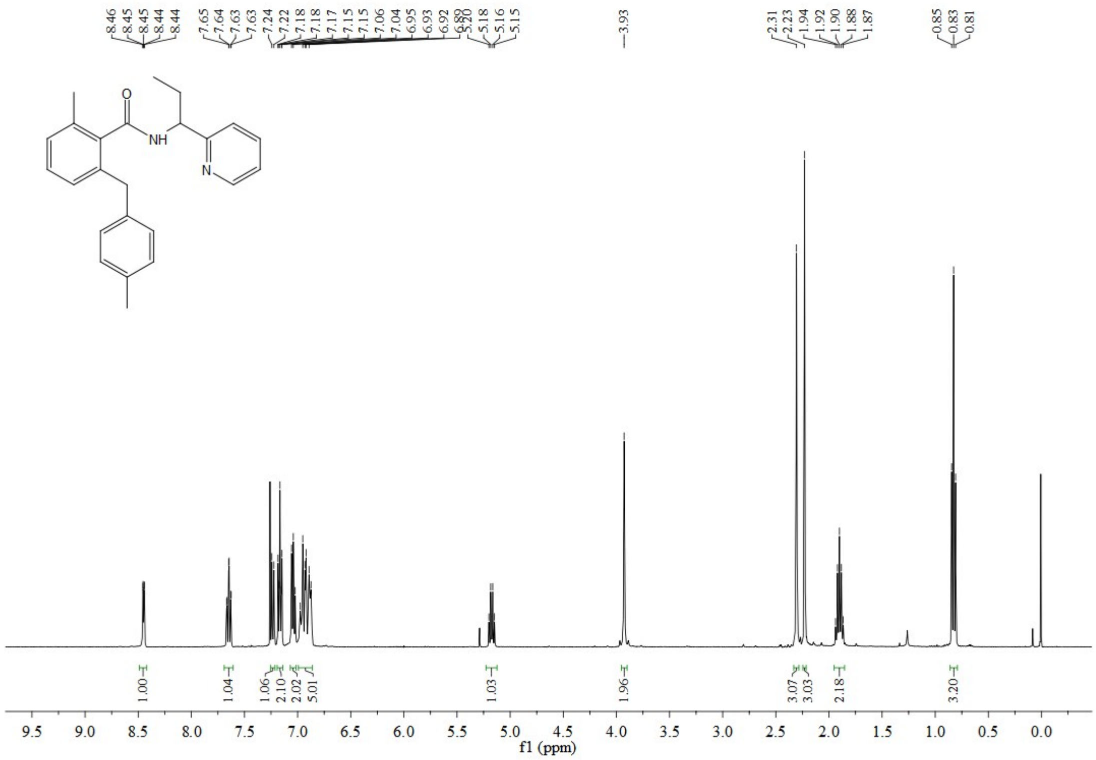

^{1}H NMR spectrum of compound **3rr**

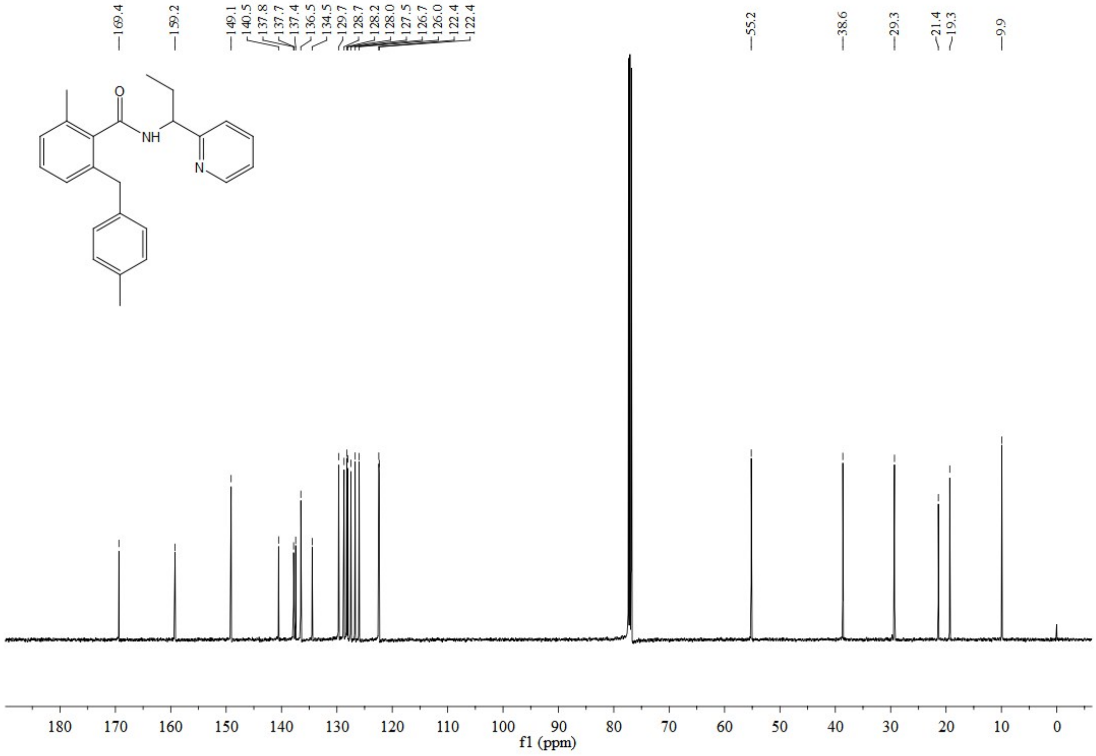

^{13}C NMR spectrum of compound **3rr**

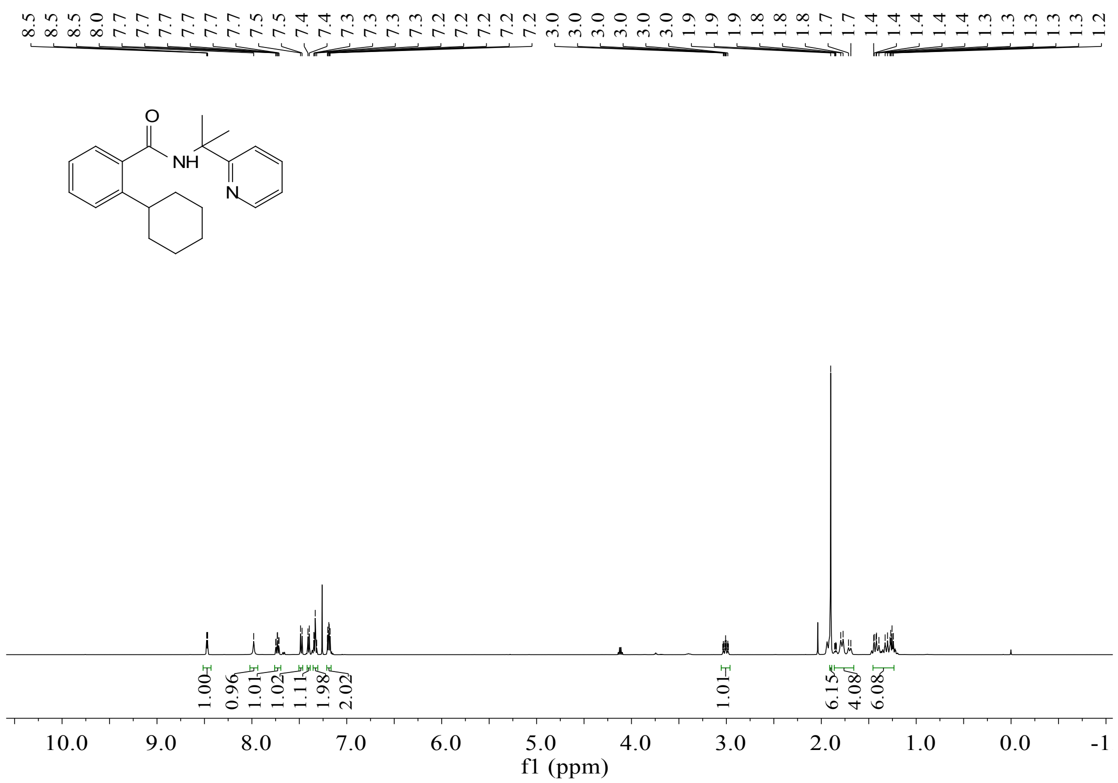

^{1}H NMR spectrum of compound **3eb**

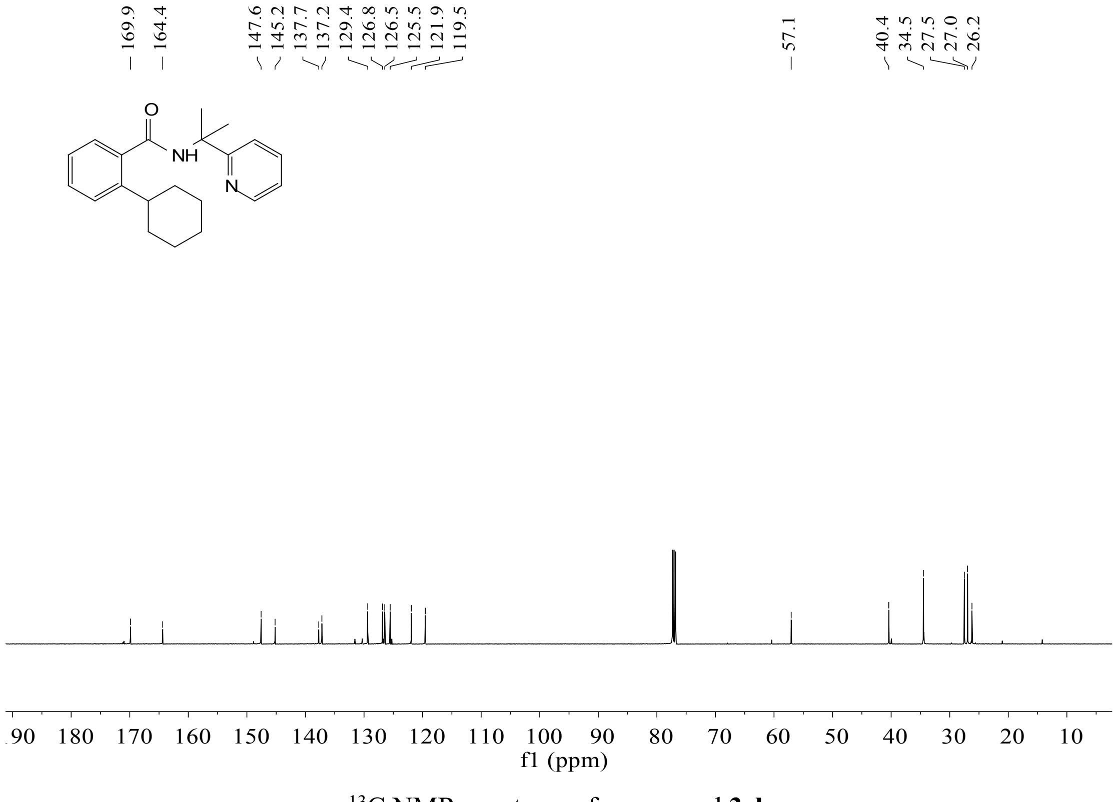

^{13}C NMR spectrum of compound **3eb**

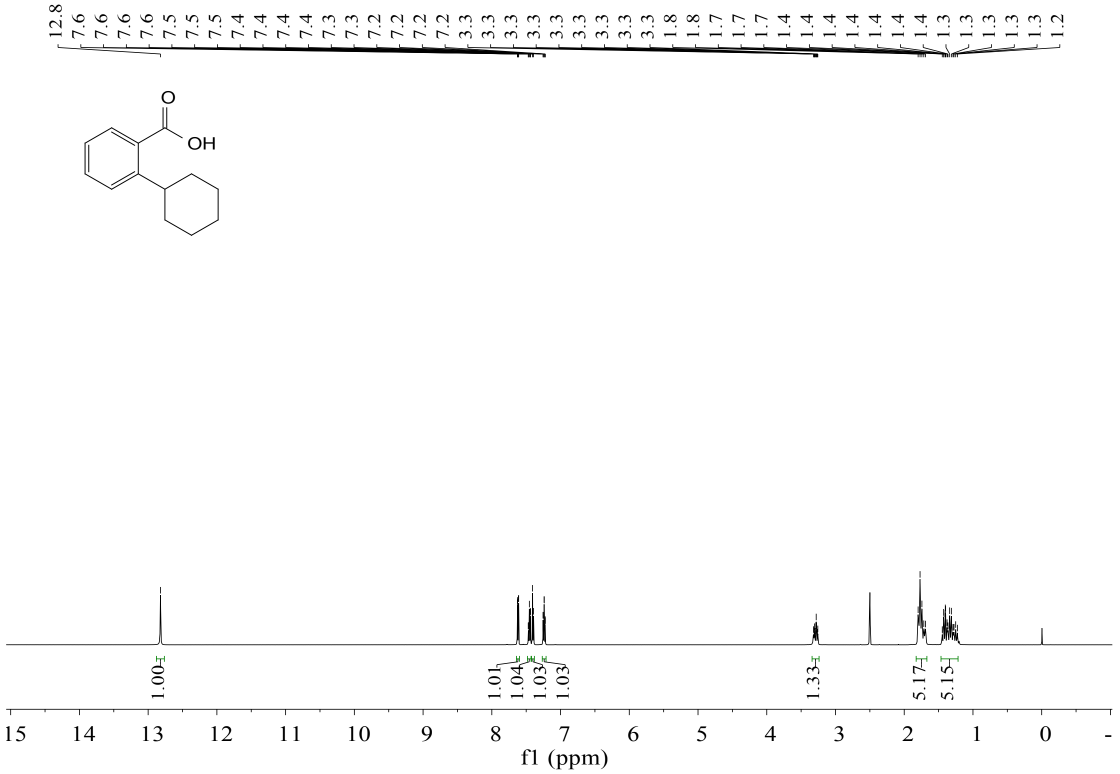

^{1}H NMR spectrum of compound **4**

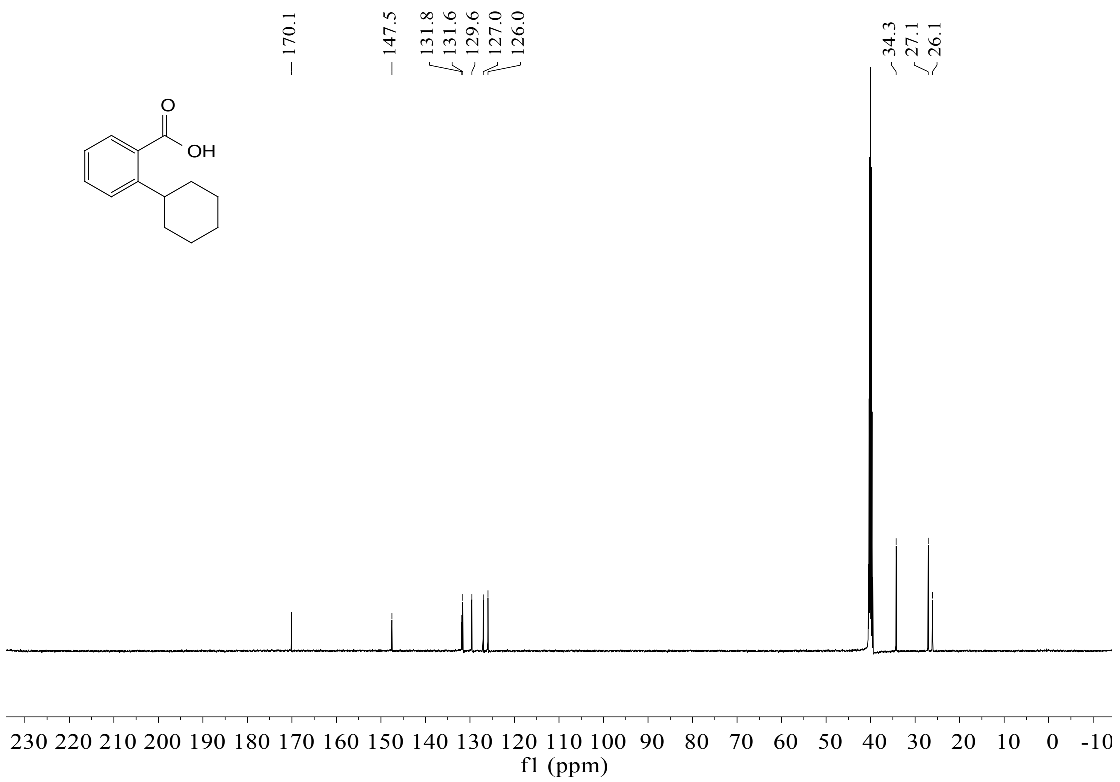

^{13}C NMR spectrum of compound **4**

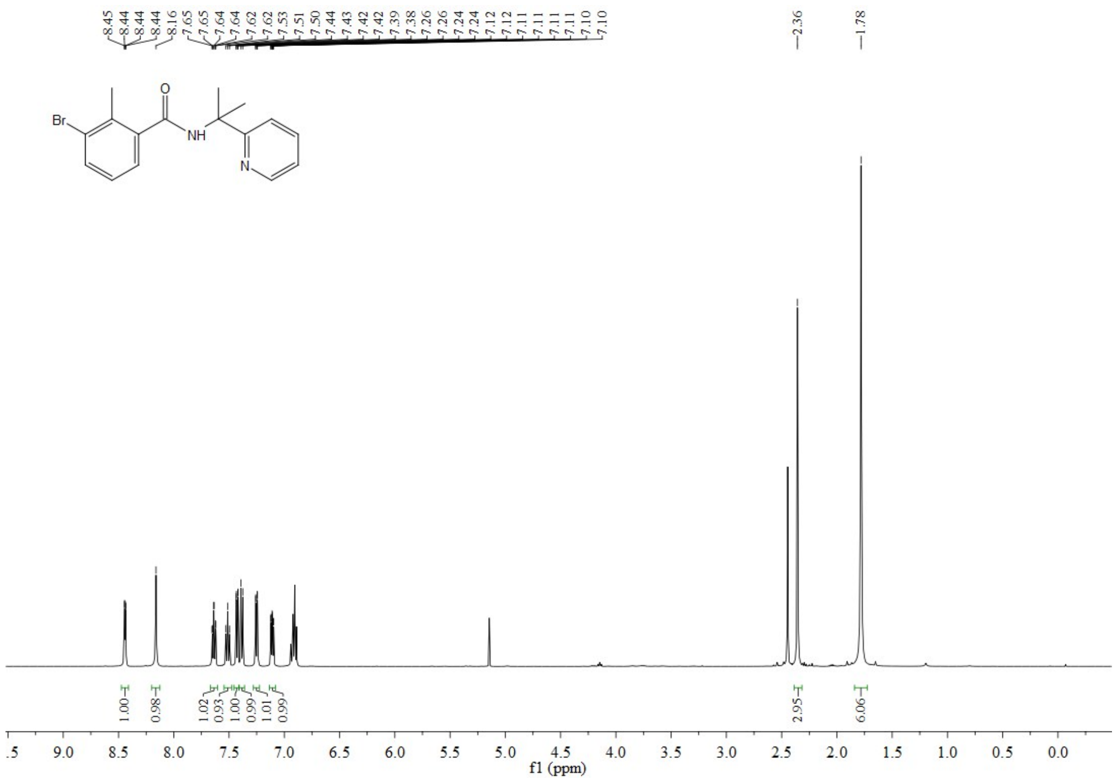

^{1}H NMR spectrum of compound **1g**

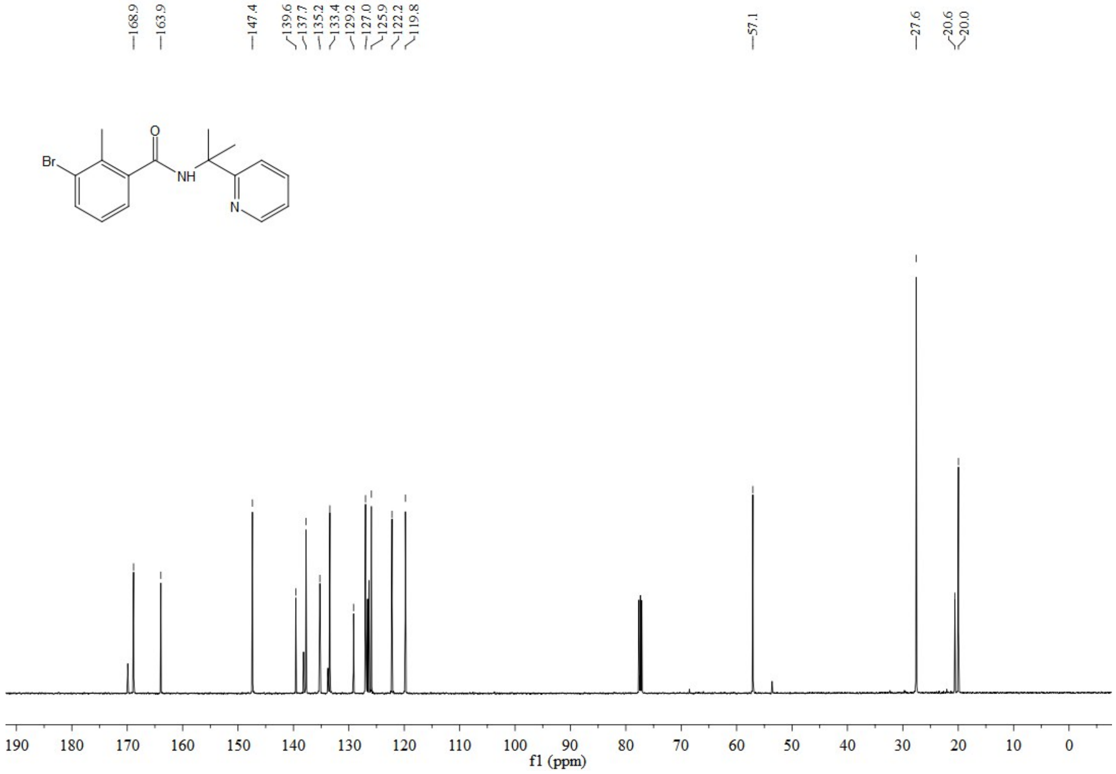

^{13}C NMR spectrum of compound **1g**

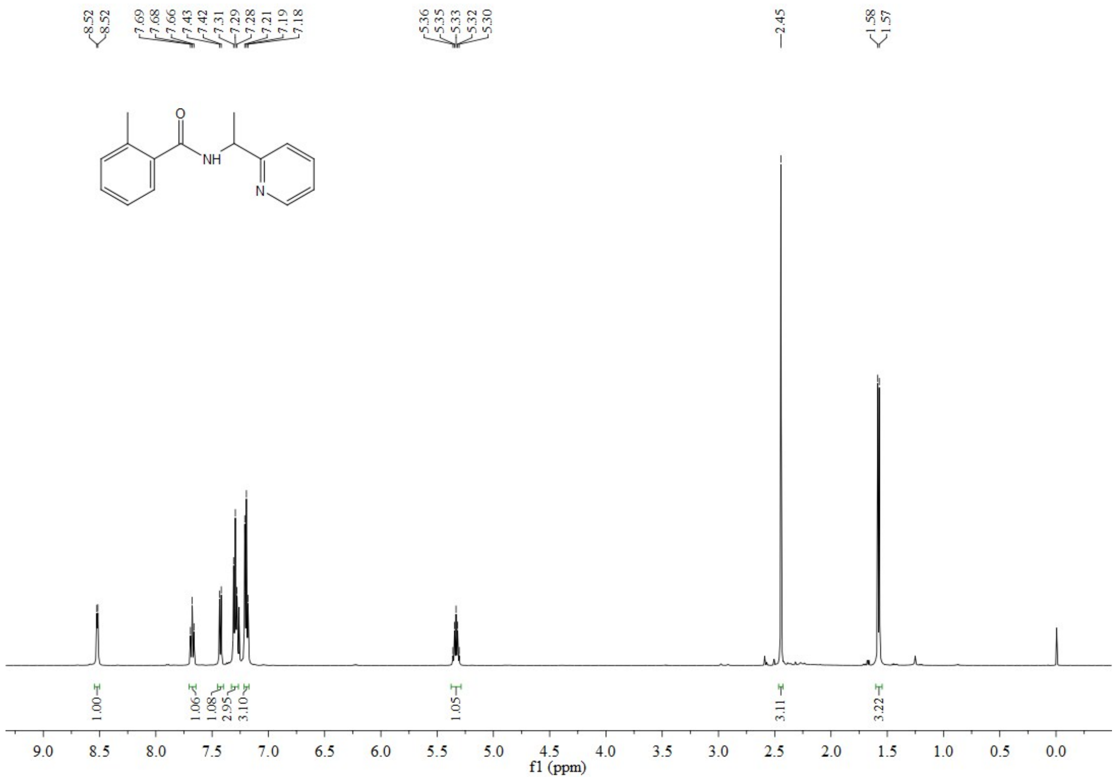

^{1}H NMR spectrum of compound **1q**

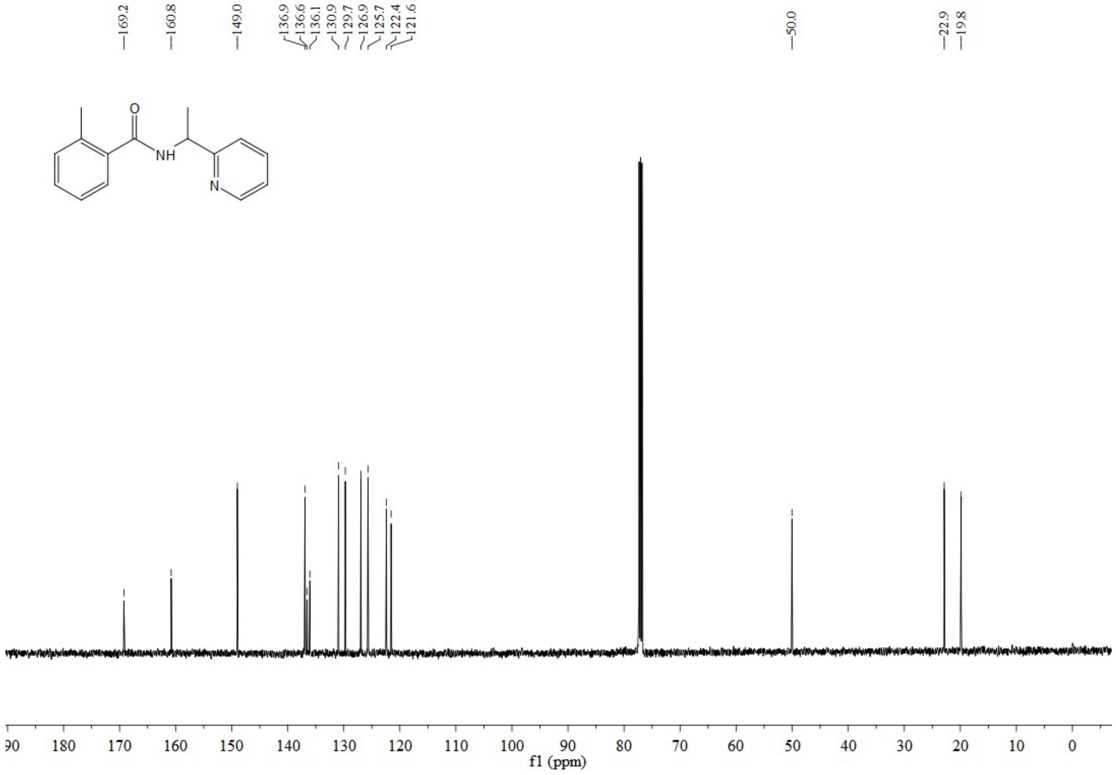

^{13}C NMR spectrum of compound **1q**

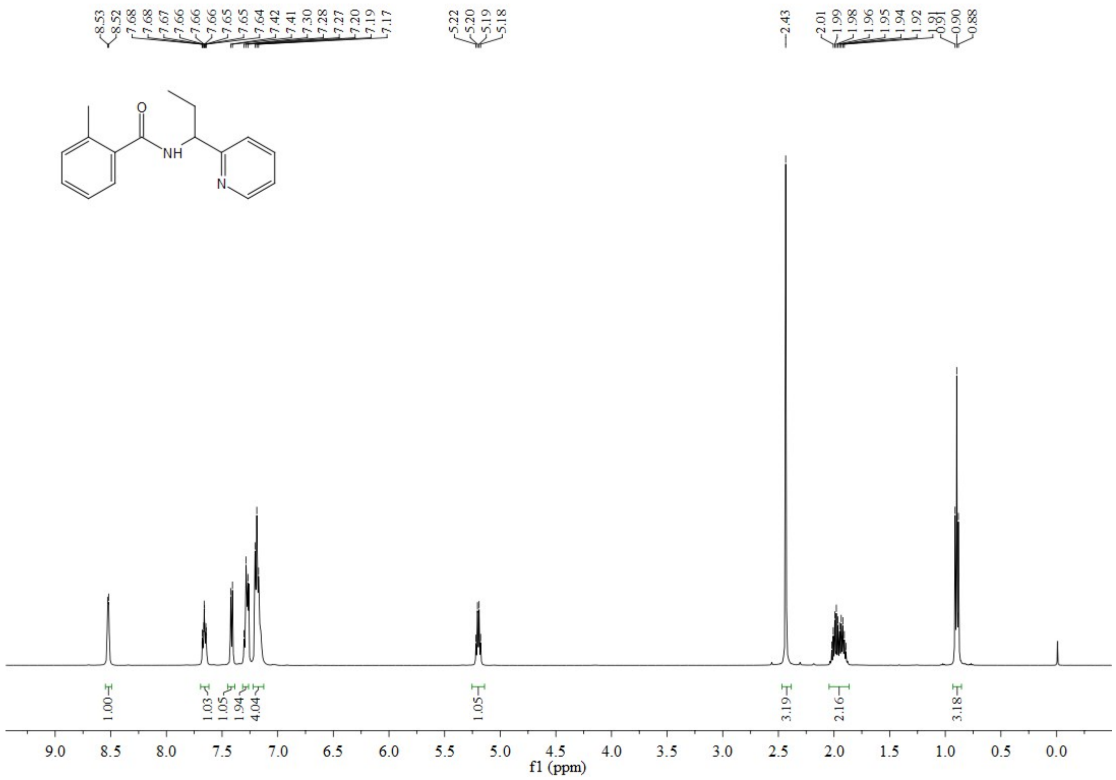

^{1}H NMR spectrum of compound **1r**

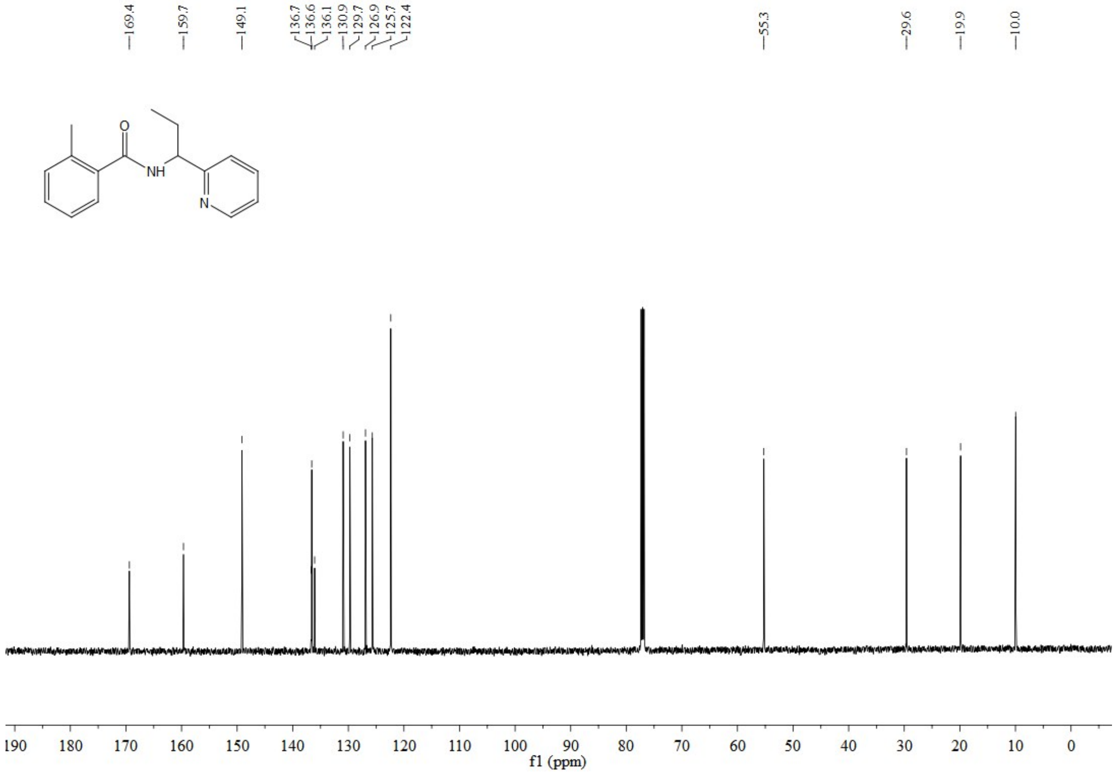

^{13}C NMR spectrum of compound **1r**

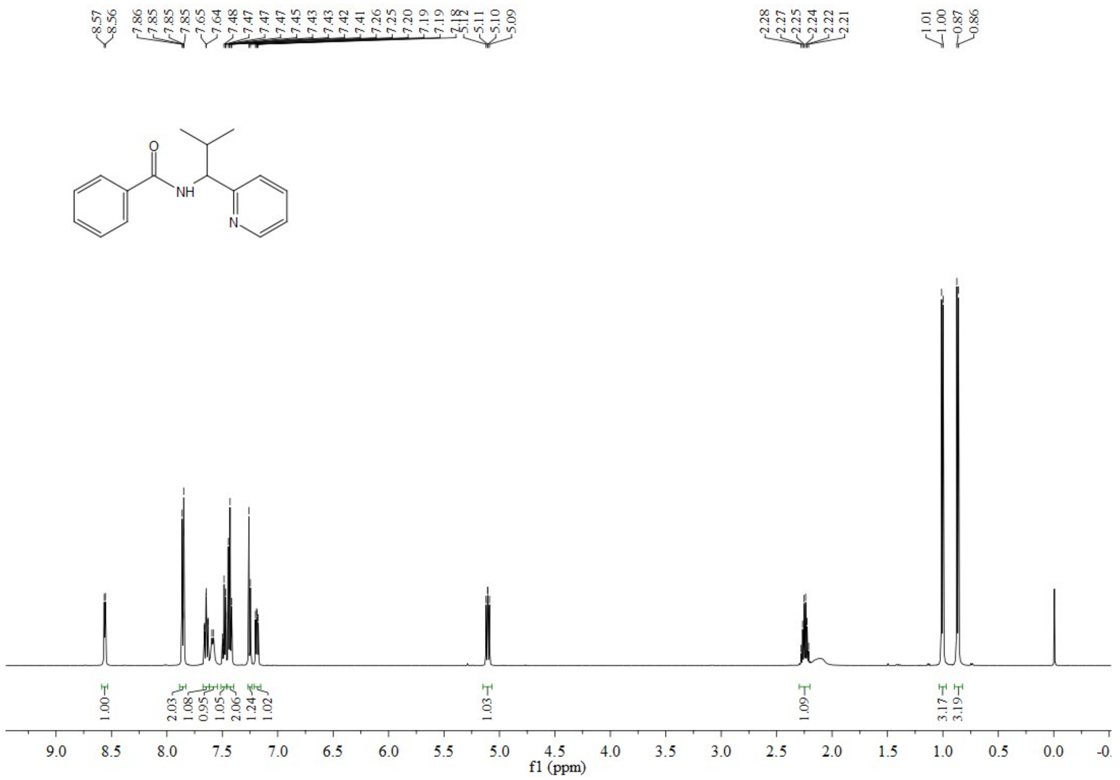

^{1}H NMR spectrum of compound **1s**

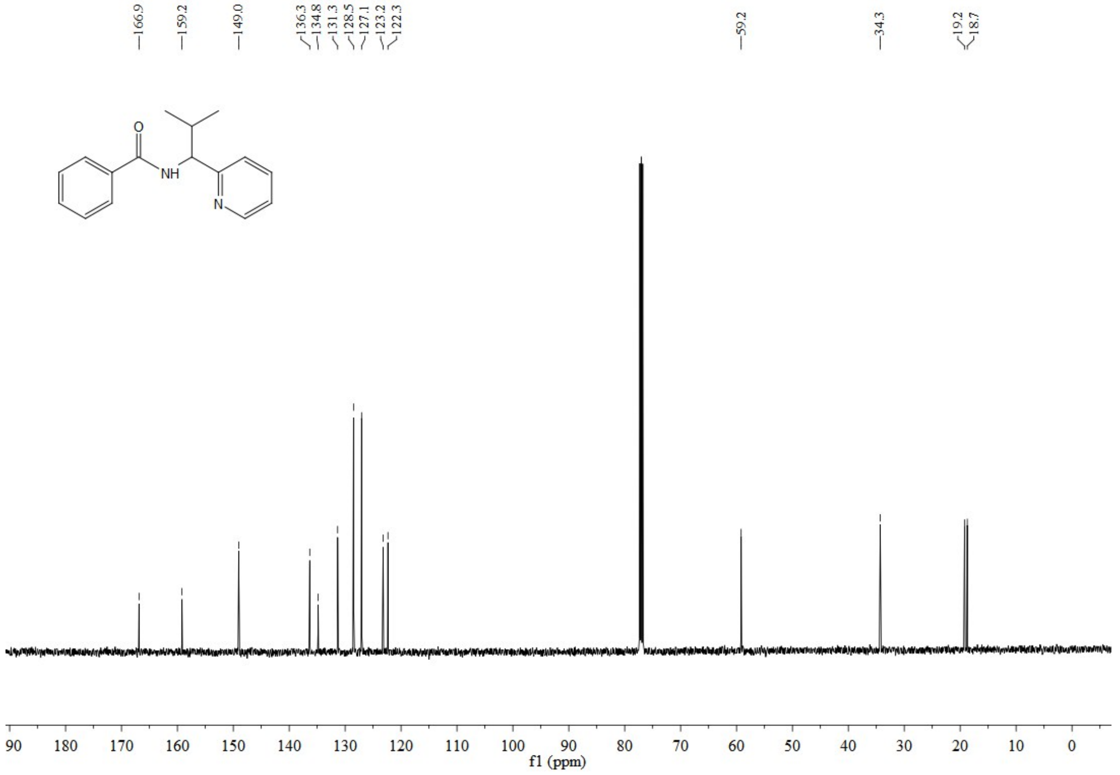

^{13}C NMR spectrum of compound **1s**

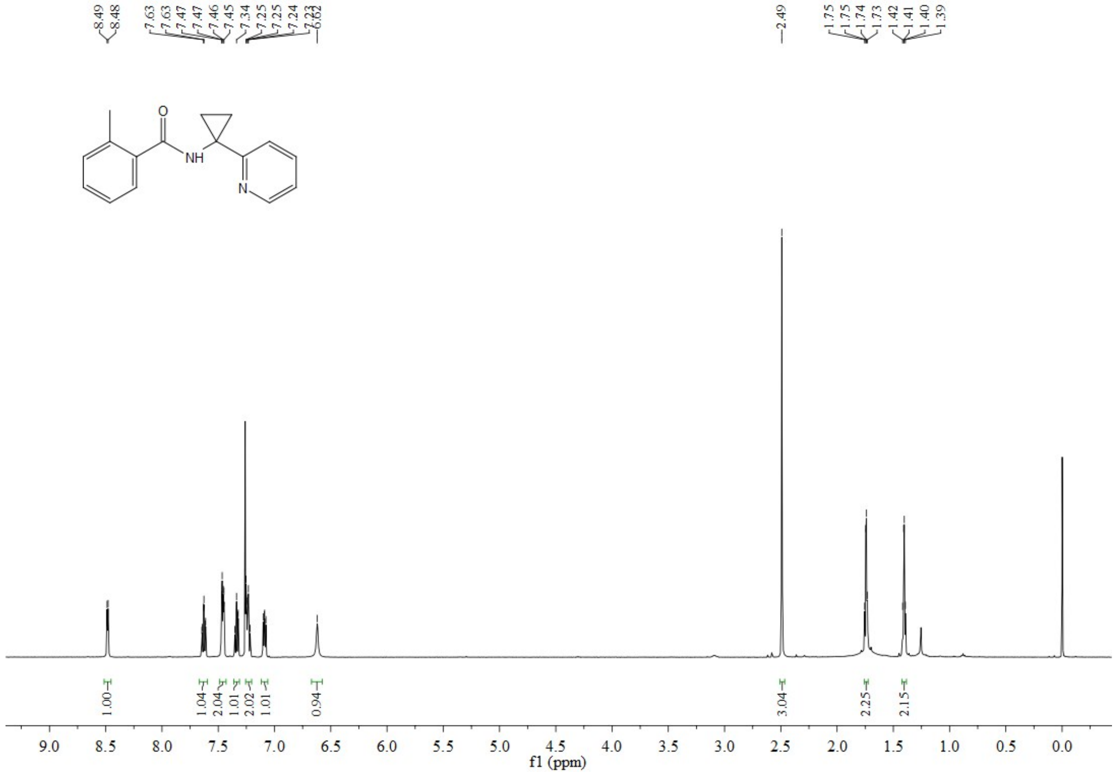

^{1}H NMR spectrum of compound **1t**

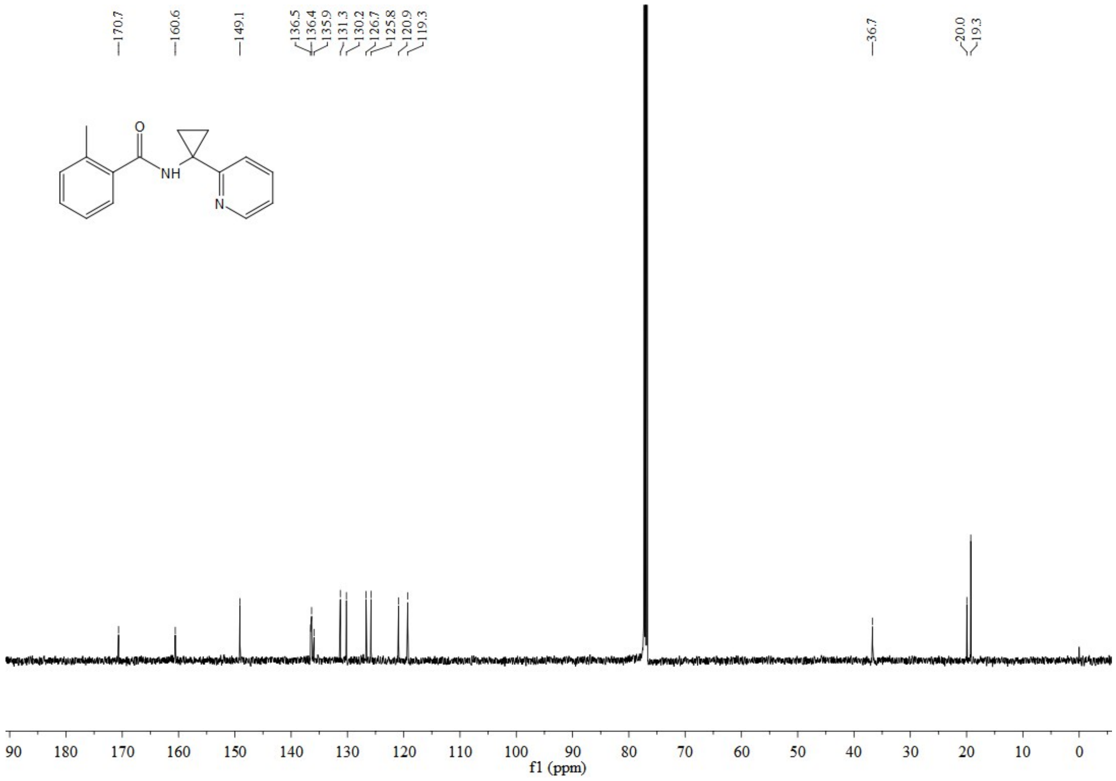

^{13}C NMR spectrum of compound **1t**

(VIII) References

[1] F-J. Chen, S. Zhao, F. Hu, K. Chen, Q. Zhang, S-Q. Zhang and B-F. Shi, *Chem. Sci.*, 2013, **4**, 4187.
